# Recent Progress in the Design of Fused-Ring Non-Fullerene Acceptors—Relations between Molecular Structure and Optical, Electronic, and Photovoltaic Properties

**DOI:** 10.1021/acsaem.1c01737

**Published:** 2021-10-26

**Authors:** Bettina Schweda, Matiss Reinfelds, Petra Hofstadler, Gregor Trimmel, Thomas Rath

**Affiliations:** Institute for Chemistry and Technology of Materials, NAWI Graz, Graz University of Technology, Stremayrgasse 9, 8010Graz, Austria

**Keywords:** organic photovoltaics, organic solar cells, NFA, small molecule acceptors, ladder type

## Abstract

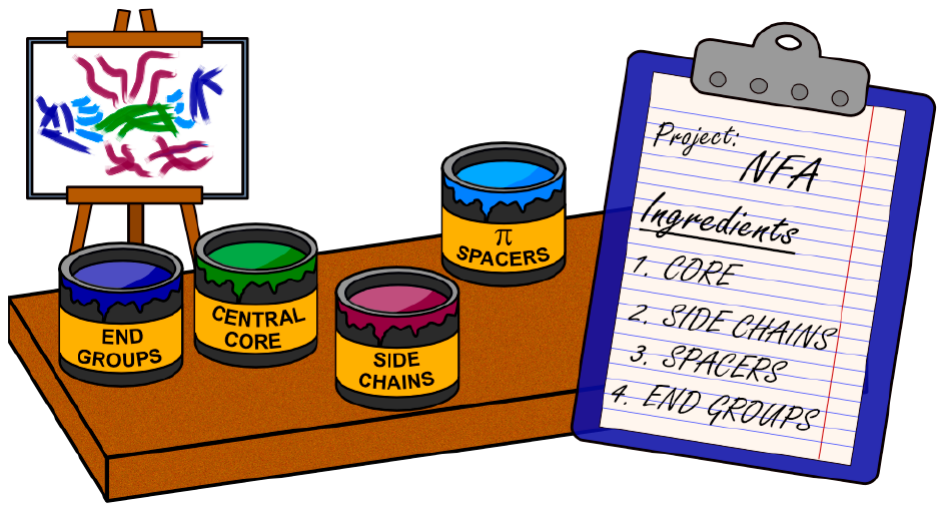

Organic solar cells are on the dawn of the next era. The change of focus toward non-fullerene acceptors has introduced an enormous amount of organic n-type materials and has drastically increased the power conversion efficiencies of organic photovoltaics, now exceeding 18%, a value that was believed to be unreachable some years ago. In this Review, we summarize the recent progress in the design of ladder-type fused-ring non-fullerene acceptors in the years 2018–2020. We thereby concentrate on single layer heterojunction solar cells and omit tandem architectures as well as ternary solar cells. By analyzing more than 700 structures, we highlight the basic design principles and their influence on the optical and electrical structure of the acceptor molecules and review their photovoltaic performance obtained so far. This Review should give an extensive overview of the plenitude of acceptor motifs but will also help to understand which structures and strategies are beneficial for designing materials for highly efficient non-fullerene organic solar cells.

## 1 Introduction

Photovoltaics is a major pillar in tackling climate change, one of the biggest current threats to mankind. Aiming at a resource- and cost-efficient production combined with scalability and a low carbon footprint, organic solar cells (OSCs) are a third-generation photovoltaic technology, which could well meet these targets. OSCs have been the objective of intensive research for several decades, and thanks to continuous advancements in the properties of the absorber materials and in particular due to the introduction of non-fullerene acceptors (NFAs), power conversion efficiencies (PCEs) have very recently surpassed 18%,^1^ thus being in terms of efficiency already highly competitive with other established and emerging thin film technologies.^2,3^

**Figure 1:**
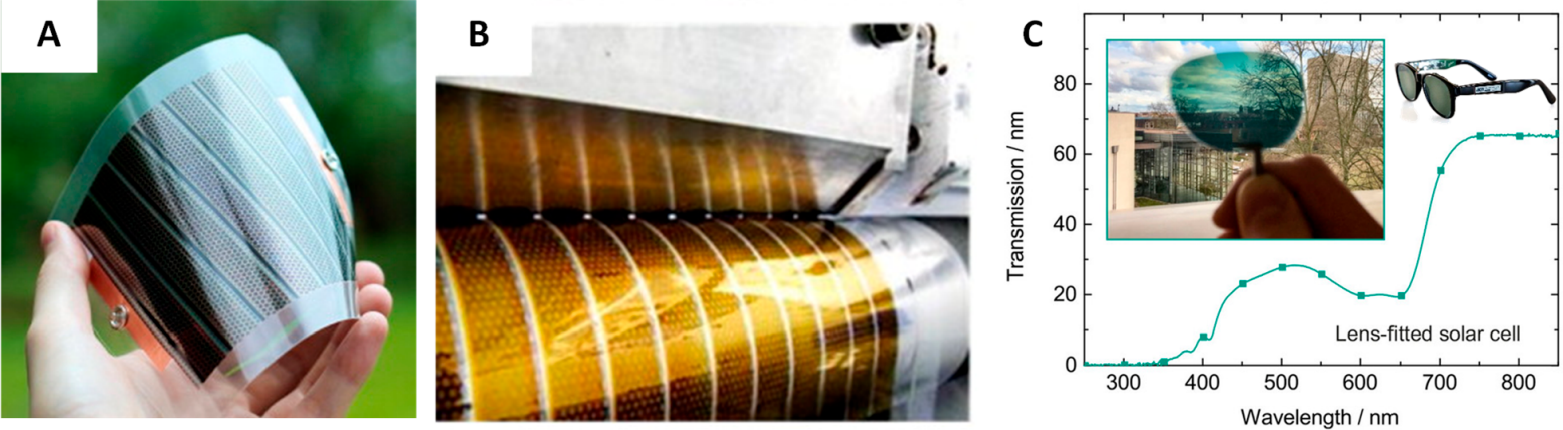
(A) Example of a flexible organic solar cell and (B) roll-to-roll printing of the active layer. Reprinted with permission from ref (24). Copyright 2013 Elsevier. (C) Example of a semitransparent organic solar cell integrated into self-powered sunglasses. Reprinted with permission from ref (25). Copyright 2017 Wiley-VCH.

In addition to these very promising power conversion efficiencies, organic solar cells possess unique properties making them attractive for a variety of appliations.^4^ The absorber in organic solar cells consists of a very thin layer comprising at least two different organic semiconductors, e.g., a conjugated polymer and a small molecule, with high absorption coefficients. Thus, lightweight and flexible solar cells can be realized (Figure 1A).^5,6^ In addition, OSCs can be processed from solution via coating and printing techniques without the need for high temperature treatments,^7^ and the usage of flexible substrates makes large area, high throughput roll-to-roll processing highly feasible (Figure 1B).^8−10^ The possibility to tune the absorption range of the active layer components by modifying their chemical structure allows the realization of colored and semitransparent devices (Figure 1C).^11−13^ These properties enable interesting new applications, such as their integration into glass facades and windows of buildings in an urban environment, into greenhouses in the agricultural sector,^14^ or into wearables and self-powered devices, where they can be used also for indoor light energy recycling.^15−18^ However, even though impressive PCEs are obtained, the long-term stability of OSCs is still a challenging factor in terms of their usage in a broad range of applications.^19,20^ Recent reviews address the status-quo and the current challenges and progress regarding the stability.^21−23^

As already briefly mentioned above, the significant increase of PCEs observed in the past years has been made possible to a great extent by the concerted effort of the whole research community working in this field leading to continuous improvement as well as the introduction of new materials. In the first organic solar cells reported by Tang et al. in 1986, a perylene-based acceptor was used.^26^ Only a few years after, Sariciftci et al. published a seminal paper on photoinduced electron transfer from a conducting polymer to fullerenes.^27^ The outstanding electronic properties of fullerenes and their derivatives soon established them as dominant acceptor materials in the first two decades of OSC research. Thus, a lot of our current understanding of how organic solar cells function is coming from this era.^28^ While with organic solar cells based on polymer/PCBM absorber layers maximum PCEs slightly above 11% have been reported,^29−32^ these values can be exceeded with modern n-type small molecular acceptors, also referred to as non-fullerene acceptors.^1,33^ In the middle of the last decade, first very efficient NFAs (e.g., ITIC, IDIC, O-IDTBR, IDT-2BR, or IEIC)^34−39^ revealing high performance in organic solar cells competitive or higher compared to similar devices based on fullerenes as acceptors were found. Up to now, several hundred new NFA structures have been reported and applied in organic solar cells in combination with a large variety of donor materials.

### 1.1 Device Configuration and Working Principle

**Figure 2:**
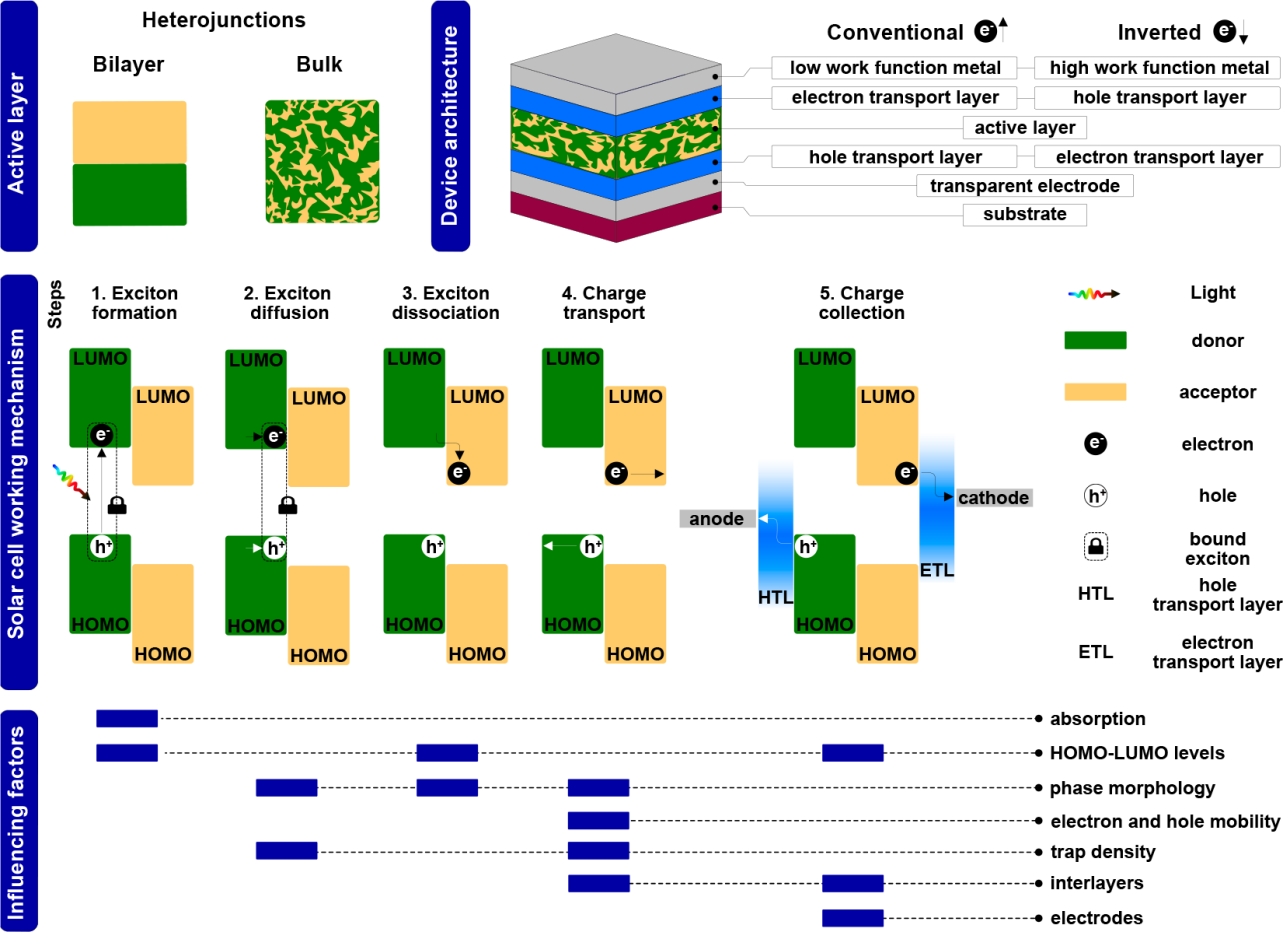
Device architecture and working mechanism of non-fullerene organic solar cells.

The absorber layer of OSCs typically consists of a combination of at least two organic semiconductors, a so-called donor (an electron-donating semiconductor, mainly a conjugated polymer) and an acceptor (electron-accepting semiconductor—another polymer, fullerene derivatives, or non-fullerene acceptors) which are arranged either in a bilayer heterojunction or in a bulk heterojunction, in which both form an interpenetrating, bicontinuous network (Figure 2).^40^ This absorber layer is typically embedded between one transparent (e.g., indium tin oxide - ITO) and one metal (e.g., Ag, Ca/Al) electrode. In addition, selective electron and hole transport layers are used between the electrodes and the active layer in order to facilitate charge extraction.^41,42^ Often used electron transport layers are metal oxides such as ZnO or organic polyelectrolytes (e.g., PFN-Br, poly(9,9-bis(3′-(*N*,*N*-dimethyl)-*N*-ethylammoinium-propyl-2,7-fluorene)-*alt*-(2,7-(9,9-dioctylfluorene))dibromide), while typically applied hole transport layers are PEDOT:PSS or MoO_3_. For a comprehensive summary on interlayers, a recent review and references therein are suggested.^43^ Moreover, organic solar cells can be prepared either in the conventional or the inverted device architecture, which are illustrated schematically in Figure 2. In the conventional architecture, a glass/ITO substrate is typically coated with a hole transport layer followed by the absorber layer, an electron transport layer, and a metal electrode. In the inverted architecture, the layers are stacked in the following sequence on the glass/ITO substrates: electron transport layer (e.g., ZnO)/absorber layer/interlayer (e.g., MoO_3_)/metal electrode, leading to an inverted flow of the charge carriers in the device compared to the conventional architecture.

Figure 2 schematically outlines the charge generation mechanism in the absorber layer of OSCs. First, absorption of an incident photon excites an electron, forming a bound electron–hole pair, the exciton (1). Next, the exciton diffuses to the donor–acceptor interface (2). Due to a higher electron affinity of the acceptor, exciton dissociation occurs (3), leading to free electrons in the lowest unoccupied molecular orbital (LUMO) of the acceptor and free holes in the highest occupied molecular orbital (HOMO) of the donor. Once these free charge carriers are generated, they are transported to the respective electrodes (4), where they are collected (5). In Figure 2, the exciton formation, diffusion, and dissociation are shown based on light absorption in the donor; however, in non-fullerene organic solar cells, also in the acceptor phase, efficient light absorption takes place, leading to an exciton formation in the non-fullerene acceptor phase, followed by a diffusion to the interface to the donor, where the exciton dissociates into free charge carriers.

To achieve high PCE values, it is essential that each of these steps starting from light absorption and the exciton formation to the charge collection at the electrodes takes place efficiently.^44^ The knowledge generated in more than two decades of OSC research allows breaking down important parameters for each of the successive steps.

First, the optoelectronic properties of the active material, e.g., the HOMO–LUMO levels of both the donor and acceptor, and their relative difference in energy are important parameters. The HOMO–LUMO gap has a direct impact on the absorption, and thus, the photoresponse of the solar cell can be maximized by selecting active layer materials with complementary band gaps (typically small band gap NFAs and medium/wide band gap conjugated polymers are used in the most efficient solar cells). On the other hand, for the realization of semitransparent solar cells, material combinations which allow the passing of certain wavelengths in the visible light spectrum can be chosen.^45^ Regarding the exciton dissociation at the donor–acceptor interface, the donor material needs to have higher HOMO and LUMO energies than the acceptor in order to enable this process, wherein typically first a charge transfer (CT) state is formed at the interface, which is subsequently converted into the free charge carriers, an electron, and a hole. Thereby, the energy difference between the LUMO of the donor and the LUMO of the acceptor has to be as high as the energy needed in order to overcome the exciton binding energy (which originates from Coulomb interactions of an electron and a hole). Regarding the hole transfer to the donor when the exciton is formed in the acceptor, similar considerations apply for the energy difference of the HOMO levels of the donor and the acceptor. Additionally, the difference between the HOMO level of the donor and the LUMO of the acceptor correlates to the maximal open circuit voltage (*V*_OC_) the device can theoretically deliver.

The second important issue is the spatial distribution of the donor and acceptor phase within the active layer, i.e., the phase separation also called phase morphology. As stated before, there are two basic concepts, the bilayer heterojunction and the bulk heterojunction approach. Whereas the first has a well-defined interface, the latter is comprised of a mixture of both with phase separation on the nanoscale (few tens of nanometers) in order to enhance the charge separation.^46,47^ This phase morphology, i.e., the domain sizes of the donor and the acceptor phase as well as their local distribution and purity, is one of the crucial parameters to obtain efficient OSCs, as it affects the transport related phenomena in the absorber layer such as exciton diffusion and therefore also their probability for dissociation as well as the transport of the separated charges to the respective electrodes.^48,49^ Since the diffusion length of an exciton in organic materials is limited within the range of a few ten nanometers,^50^ the donor–acceptor interface should be located within this distance from the place the exciton was generated in order to be dissociated into free charge carriers. Furthermore, also trapping of excitons by defects in the films or at interfaces can result in exciton quenching (non-productive recombination) and thereby a reduction of the charge generation yield. In addition, free charge carriers are prone to recombination.^51^ This happens mainly at the interfaces between the donor and the acceptor domains or due to traps within both phases. However, the distances free charge carriers can travel are significantly larger than those of excitons, due to the electric field applied to the device. A certain amount of traps is an intrinsic feature of organic semiconductors, introduced, e.g., during the film formation or by outer forces (such as oxygen or UV-light), while additional traps can also result from interactions with other phases. A detailed analysis of various traps is described by Haneef et al.^51^ Regarding the charge transport, balanced charge carrier mobilities in the donor and the acceptor phase are beneficial. Moreover, pristine layers of donor and acceptor would be ideal as realized in the bilayer concept, but here the yield of excitons reaching the interface is limited. In contrast to the bilayer concept, the bulk heterojunction is a compromise between a high interface for charge separation and domain sizes, which are large enough to provide good pathways for charge transport.^52−55^ Therefore, morphology control of the active layer has attracted much research attention.^47,56,57^

At present, the most often used active layers contain one donor and one acceptor in a bulk heterojunction. However, a single component active layer is also possible. In this case, the donor and acceptor moieties are a part of the same molecule (or oligomer, or polymer). The efficiencies of these solar cells currently reach values up to around 11%.^58−60^ At the same time, if a third component is added to the active layer, ternary solar cells are obtained.^61,62^ The combination of three materials allows for a more efficient harvesting of the solar light and can also have advantages regarding reduced trap states and device performance and stability;^63^ however, the morphological control becomes more challenging. Another way to broaden the solar light absorption is by use of two (tandem)^64−66^ or multiple junction cells.^67^

### 1.2 The Scope of This Work

The imaginativeness of chemists has created a variety of new non-fullerene acceptors, and the amount of new literature focusing solely on NFA design is overwhelming. In light of the large amount of published data, it is desirable to summarize and to find ways to organize and generalize the latest findings. It is not always necessary to strictly separate the donors from acceptors, as often common improvement strategies (such as chlorination^68^ and fluorination^69,70^), design strategies (ladder-type compounds^71^), or substance classes (such as diketopyrrolopyrroles^72^) are used for both. Also, computational methods are very useful to guide the development of both substance classes or even the entire OSC.^73,74^

The fast moving field of non-fullerene organic solar cells has been the objective of several recent perspectives and reviews covering small molecule NFAs and the corresponding solar cells in general (e.g., refs (33, 49, and 75−77)) or specific compound classes, for example, rylene dyes,^78−81^ Y-type^82−84^ and IDIC/ITIC-type acceptors,^85^ or polymeric acceptors.^86,87^ Also, design strategies have been reviewed, such as A–D–A-structure-type^88,89^ and fused-ring molecules^90,91^ as well as isomeric,^92^ star shaped,^93^ and asymmetric compounds.^94^

In this Review, we aim at giving an overview of recently introduced NFA structures based mainly on ladder-type fused-ring systems investigated in the years 2018–2020. Due to the large number of studies reporting on the synthesis and characterization of NFAs and their application in solar cells, we only focus on data of solar cells containing one polymer donor and one small molecule acceptor material in the absorber layer and ternary solar cell data are not included in our discussion to be able to give a better comparability between the photovoltaic performances of the different NFAs. The following chapters will give general ideas on the acceptor design of fused-ring systems. As a basis for categorizing, we have chosen the size of the central fused-ring core. The most efficient solar cells to date are based on seven-ring structures; thus, they will be covered first, followed by NFAs containing five, six, eight, nine, and more fused-ring cores. Moreover, we analyzed and compared the photovoltaic properties of these over 700 NFA structures reported within the last three years and discuss obtained correlations at the end of this Review.

## 2 Non-Fullerene Acceptor Design

**Figure 3:**
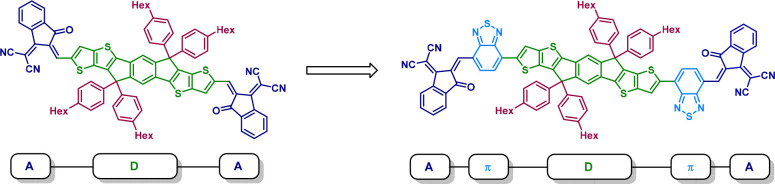
Possible design strategies for non-fullerene acceptors. Left, illustration of an A–D–A-type acceptor (ITIC, **7-1**); right, example of an A-π-D-π-A-type acceptor based on ITIC.

The earliest (but still most common) design strategy for non-fullerene acceptors is the combination of a weak electron-donating core (D) and two strong electron-withdrawing groups (A) as peripheral units, also referred to as the acceptor–donor–acceptor (A–D–A) structure.^95,96^ This framework profits from π-electron push–pull effects, which is not only good for light absorption but also good for charge transfer. A prominent example of this structural motif is ITIC (**7-1**), which was first reported in 2015.^34^ Since then, ITIC and its derivatives are among the most popular non-fullerene acceptors. It consists of a planar, rigid, ladder-type core unit containing a fused aromatic ring system, namely, indacenodithieno[3,2-*b*]thiophene (IDTT or IT) referred to as the donor unit. This unit is decorated with strong electron-withdrawing 2-(3-oxo-2,3-dihydroinden-1-ylidene)malononitrile (INCN or IC) end groups on each side of the central donor unit; thus, they are referred to as the acceptor units (see Figure 3, left).^34,97^

An important modification of the A–D–A structural motif was the introduction of an electron-poor ring into the central donor unit leading to the A–DA′D–A structure type. The most prominent examples are the Y-series acceptors, which currently also hold the record PCEs.^98^ Another strategy is to include π-spacers, whereby compounds with the general structure A−π–D−π–A (Figure 3, right) are obtained.^99^ By selecting the appropriate type of π-spacers, the quinoid character of the conjugated backbone can be increased.^100^ Good results can also be achieved if asymmetric molecules are designed, for example, A–D–A′ or A–D−π–A (as well as combinations thereof).^94^ Such molecules often have larger dipole moments and improve packing in the solid state, which can reduce energy loss and improve the fill factor (FF) in solar cells.

An overall coplanar structure is advantageous for light absorption and charge mobility in the solid state. In the ladder-type central core structures, this is achieved by a covalent ring locking of neighboring heterocycles. In order to keep the π-spacers and end groups coplanar to the central core, non-covalent interactions, i.e., O···S, N···S, H···S, and X···S (X = Cl, Br, F) are utilized. Nevertheless, too much planarity can cause poor solubility if no side chains of sufficient length are used. Side chains not only ensure solution processability but can also have an influence on the optical band gap and the energy levels of the molecule despite not being a part of the electronic conjugation (π-system). Due to their steric bulkiness, they can also prevent too strong self-aggregation in film. Usually, side chains are attached on the central core and/or the π-spacer. This allows the end groups to form intermolecular interactions, crucial to the electron transport in the solid state. Since the end groups are a part of the conjugated π-system, already small modifications can lead to relatively large changes of the optical band gaps and the energy levels of the entire molecule. A very common way to modify the end groups is the introduction of halogens. For example, fluorine atoms lead to downshifted energy levels and reduced optical band gaps.^101,102^

All of the above-mentioned design principles can be found among the large set of new seven-ring acceptors published over the last three years. For this central core size, we have elucidated them in detail. However, a more exhaustive analysis of π-spacers is outlined in the five-ring chapter. This is the logical consequence of the smaller conjugation length of the five-ring central core, which leads to higher optical band gaps. Using π-spacers can shift the band gap values to energies which are more similar to those of the seven-ring central cores. Asymmetric central cores are most often found in six-ring and larger central cores.

**Figure 4:**
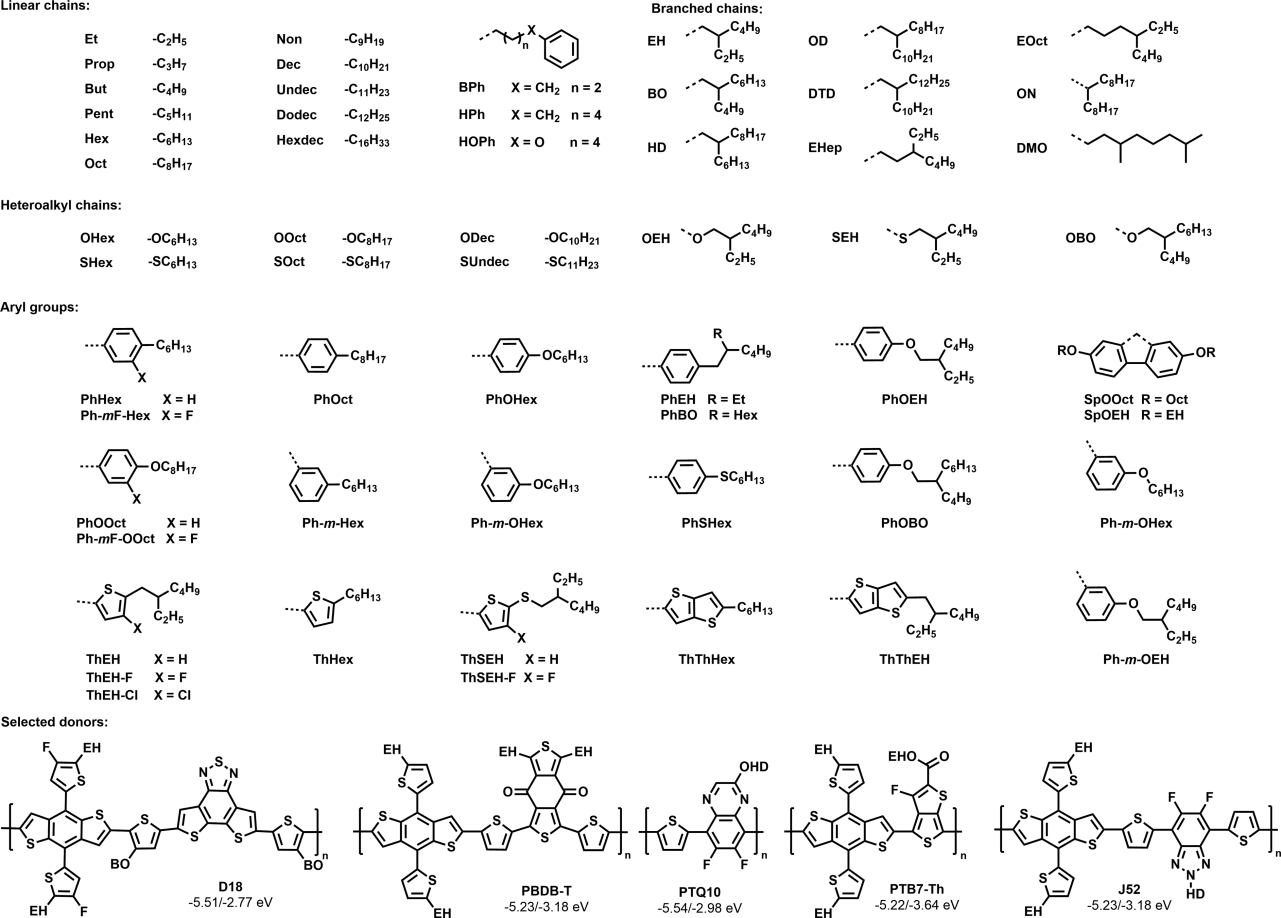
Summary of all side chains used in this Review with their respective abbreviations and selected donor molecules.

Figure 4 contains a summary of all side chains and their abbreviations as well as selected donor materials used in the most efficient non-fullerene organic solar cells to date. For a complete summary of state-of-the-art donor materials, the reader is referred to recent reviews and references therein.^103−105^

## 3 Seven Fused Aromatic Ring Systems

### 3.1 Impact of the Acceptor Units

**Figure 5:**
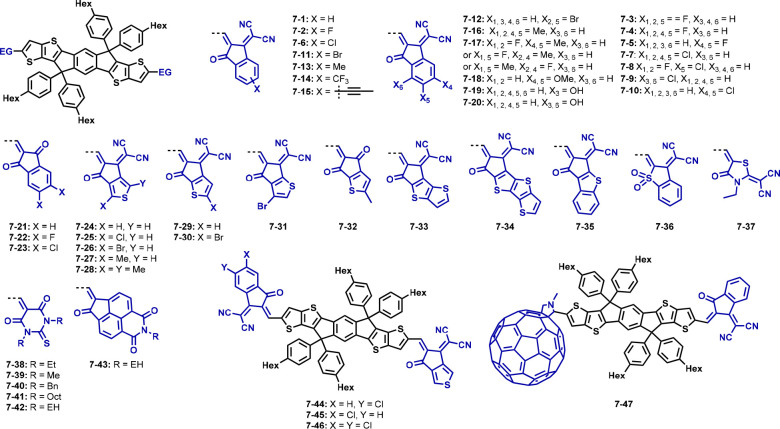
Structures of non-fullerene acceptors with the same core unit bearing hexylphenyl side chains and different acceptor units.

IDTT, the core unit in ITIC (**7-1**), is the most frequently used central donor unit for NFAs in the years 2018–2020, which is why we decided to discuss the influence of different acceptor units based on this donor core. The discussed structures are depicted in Figure 5, and the material and photovoltaic properties are summarized in Table 1. The ITIC acceptor has an INCN unit as an end group and possesses a band gap of 1.59 eV as well as strong and broad absorption in the region 500–750 nm.^34^ Being the first highly efficient NFA, many research groups investigated ITIC itself and its behavior in OSCs with different polymer donors, e.g., PBDB-T,^106^ PTB7-Th,^107^ PM6,^108^ etc. Before 2020, Bin et al. achieved the highest PCE (11.4%) of ITIC-based single-junction OSCs with J71 as a donor.^109^ In 2020, this PCE was topped by Li et al. with 13.5%, where the new polymer donor PBTA-PS-F was used. The device showed in comparison a higher electron mobility of 3.80 × 10^–4^ cm^2^ V^–1^ s^–1^, *V*_OC_ of 0.97 V, *J*_SC_ of 18.5 mA cm^–2^, and FF of 75%.^110^

**Table 1:** Optical, Electrical, and Photovoltaic Properties of Non-Fullerene Acceptors **7-1**–**7-47**

NFA	original name	HOMO^a^ (eV)	LUMO^a^ (eV)	*E*_g_^opt^ (eV)	donor	D:A ratio	*V*_OC_ (V)	*J*_SC_(mA cm^–2^)	FF (%)	PCE (%)	μ_e_^c^(cm^2^ V^–1^ s^–1^)	ref.
**7-1**	ITIC	–5.50	–3.89		PBDS-T	1.2:1	0.98	18.2	60	10.7	-/3.6 × 10^–4^	(135)
	ITIC	–5.50	–3.85	1.61	PM6		1.01	15.6	66	10.3	1.7 × 10^–4^/1.1 × 10^–4^	(108)
	ITIC	–5.68	–4.03	1.58	PBDB-T		0.89	16.5	71	10.4	6.4 × 10^–4^/3.1 × 10^–4^	(106)
	ITIC				PBTA-PS-F	1:1	0.97	18.5	75	13.5	-/3.8 × 10^–4^	(110)
**7-2**	IT-2F	–5.63	–4.06^b^		PM6		0.92	19.3	70	12.7	8.4 × 10^–5^/-	(111)
**7-3**	IT-3F	–5.67	–4.09^b^		PM6		0.90	20.0	73	13.8	1.0 × 10^–4^/-	(111)
**7-4**	IT-4F	–5.68	–4.14^b^		PM6		0.86	20.8	74	13.6	9.1 × 10^–5^/-	(111)
	IT-4F	–5.66	–4.14		D18	1:1.6	0.86	22.3	75	14.9		(136)
**7-5**	*a*-IT-2F	–5.67	–4.07	1.56	PBDB-T	1:1	0.78	19.1	69	10.3	-/4.0 × 10^–5^	(114)
**7-6**	ITIC-2Cl	–5.68	–3.99	1.55	PM6	1:1	0.92	19.1	75	13.2		(115)
**7-7**	ITIC-4Cl	–5.75	–4.09	1.48	PM6	1:1	0.79	22.7	75	13.5		(115)
**7-8**	ITIC-γCl-2F	–5.52	–3.88		PM6	1:1.2	0.85	19.6	72	12.0	-/1.2 × 10^–4^	(116)
**7-9**	ITIC-2Cl-β	–5.30	–3.71		PM6	1:1	0.94	18.5	65	11.2	-/1.1 × 10^–4^	(117)
**7-10**	α-ITIC-2Cl	–5.29	–3.77		PM6	1:1	0.88	18.9	74	12.2	-/2.9 × 10^–4^	(117)
**7-11**	ITIC-2Br-*m*	–5.53	–3.90	1.53	PM6	1:1	0.87	18.0	70	10.9	-/7.6 × 10^–4^	(118)
**7-12**	ITIC-2Br-γ	–5.54	–3.90	1.53	PM6	1:1	0.89	19.0	71	12.1	-/8.3 × 10^–4^	(118)
**7-13**	IT-M	–5.58	–3.98		PDTF-TZNT	1:1	0.80	17.3	73	10.1	-/2.2 × 10^–4^	(119)
	IT-M	–5.51	–3.80		PBDFP-Bz	1:1	1.02	18.3	69	12.9		(137)
**7-14**	IT-CF_3_	–5.71	–3.97	1.49	PM6	1:1	0.84	20.9	76	13.3	4.7 × 10^–4^/2.8 × 10^–4^	(122)
**7-15**	ITEN	–5.63	–3.90	1.58	PM6		0.99	16.5	67	10.9	8.8 × 10^–4^/3.6 × 10^–4^	(108)
**7-16**	IT-DM	–5.58	–3.82	1.63	J71	1:1	1.02	16.7	71	12.1	7.0 × 10^–4^/4.1 × 10^–4^	(112)
**7-17**	ITCF	–5.59	–3.95	1.57	J71	1:1	0.91	18.5	79	13.3	7.4 × 10^–4^/5.3 × 10^–4^	(112)
**7-18**	*a*-IT-2OM	–5.61	–3.92	1.63	PBDB-T	1:1	0.93	18.1	72	12.1	-/3.9 × 10^–5^	(114)
**7-19**	IT-OH	–5.57	–3.92	1.54	PM6	1:1	0.92	17.4	70	11.2		(123)
**7-20**	IT-DOH	–5.58	–3.93	1.53	PM6	1:1	0.96	17.8	73	12.5		(123)
**7-21**	IO-4H	–5.61	–3.65	1.88	PBDB-T	1:1.5	1.12	5.95	43	2.86	-/6.5 × 10^–7^	(125)
**7-22**	IO-4F	–5.65	–3.83	1.85	PBDB-T	1:1.5	1.05	12.2	63	8.06	-/4.5 × 10^–7^	(125)
**7-23**	IO-4Cl	–5.72	–3.83	1.80	PM6	1:1.5	1.24	11.6	68	9.80		(124)
**7-24**	ITCPTC	–5.62	–3.96	1.58	PM6	1:1	0.97	17.1	74	12.3	6.5 × 10^–4^/3.6 × 10^–4^	(127)
**7-25**	ITC-2Cl	–5.58	–4.01	1.58	PM6	1:1	0.91	20.1	74	13.6	7.8 × 10^–4^/4.2 × 10^–4^	(128)
**7-26**	ITC-2Br2	–5.59	–4.02	1.59	PM6	1:1	0.90	19.8	74	13.1	7.8 × 10^–4^/4.1 × 10^–4^	(129)
**7-27**	MeIC	–5.57	–3.92	1.58	PM6	1:1	0.99	18.5	71	13.0	8.5 × 10^–4^/4.8 × 10^–4^	(127)
**7-28**	ITCT-DM	–5.48	–3.90	1.58	PBDB-T	1:1	0.90	17.4	65	10.6	-/6.7 × 10^–4^	(130)
**7-29**	IDTC	–5.47	–3.76	1.68	PBDB-T	1:1	1.00	16.1	69	11.1	-/2.5 × 10^–4^	(131)
**7-30**	ITC-2Br	–5.73	–3.93	1.73	PM6	1:1	1.03	15.4	69	10.9	6.6 × 10^–4^/3.0 × 10^–4^	(129)
**7-31**	ITC-2Br1	–5.70	–3.95	1.70	PM6	1:1	1.01	16.6	71	11.9	6.9 × 10^–4^/3.4 × 10^–4^	(129)
**7-32**	ITCCM-O	–5.67	–3.26	2.00	J52		1.34	9.2	44	5.50	-/1.6 × 10^–5^	(133)
**7-33**	IDTTC	–5.40	–3.72	1.58	PBDB-T	1:1	1.01	18.3	73	13.5	-/5.4 × 10^–4^	(131)
**7-34**	IDTTTC	–5.34	–3.69	1.55	PBDB-T	1:1	1.03	14.5	43	6.46	-/3.8 × 10^–4^	(131)
**7-35**	ITBC	–5.49	–3.90	1.59	PBDB-T	1:1.2	0.94	19.9	65	12.1	-/5.8 × 10^–5^	(134)
**7-36**	ITBC	–5.61	–4.13	1.53	FTAZ	1:1	0.72	11.9	49	4.17		(138)
	ITTBC	–5.61	–4.13	1.53	PM6	1:1	0.86	12.5	63	6.83	-/6.7 × 10^–5^	(139)
**7-37**	IDTT-R	–5.32	–3.49	1.84	P3HT	1:1	0.78	0.83	48	0.43		(140)
**7-38**	IDTT-T	–5.51	–3.51		PTB7-Th	1:2	1.02	18.0	65	11.8	-/4.0 × 10^–3^	(107)
	IDTT-TBTA (b)	–5.51	–3.72	1.89	PTB7-Th	1:1.5	1.00	13.7	64	8.77	-/3.1 × 10^–4^	(141)
**7-39**	IDTT-TBTA (a)	–5.46	–3.72	1.89	PTB7-Th	1:1.5	1.00	10.2	53	5.44	-/4.4 × 10^–5^	(141)
**7-40**	IDTT-TBTA (c)	–5.45	–3.67	1.87	PTB7-Th	1:1.5	0.97	12.0	63	7.41	-/2.2 × 10^–4^	(141)
**7-41**	IDTT-TBTA (d)	–5.42	–3.59	1.89	PTB7-Th	1:1.5	1.01	9.10	51	4.69		(141)
**7-42**	IDTT-TBTA (e)	–5.48	–3.65	1.89	PTB7-Th	1:1.5	0.92	6.72	41	2.52		(141)
**7-43**	NIDT	–5.34	–3.58	1.85	PBDB-T	1.2:1	1.14	13.3	66	10.0	4.7 × 10^–5^/2.1 × 10^–5^	(142)
**7-44**	ITIC-Cl-δ-Th	–5.31	–3.70		PM6	1:1	0.89	17.3	73	11.1	-/1.2 × 10^–4^	(143)
**7-45**	ITIC-Cl-γ-Th	–5.30	–3.66		PM6	1:1	0.91	18.3	73	12.3	-/2.1 × 10^–4^	(143)
**7-46**	ITIC-2Cl-Th	–5.31	–3.74		PM6	1:1	0.86	18.6	72	11.5	-/8.3 × 10^–4^	(143)
**7-47**	A2	–5.37	–3.67	1.61	J71	1:1	0.98	11.6	40	4.52	9.9 × 10^–5^/5.7 × 10^–5^	(144)

a Obtained from the oxidation/reduction potential of the cyclic voltammetry (CV) measurement if not otherwise stated.

b Other method or method not defined.

c Determined via the space-charge limited current (SCLC) technique from the neat acceptor/donor:acceptor blend films if not otherwise stated.

The simplest and most frequent modification of the end groups includes the addition of substituents, such as halogens or methyl groups, on the phenyl ring of the INCN unit. Upon the introduction of two (**7-2**, IT-2F)^111^ (one on each side: INCN-F) or four (**7-4**, IT-4F)^111^ (two on each side: INCN-2F) fluorines on the molecule, the band gap is narrowed compared to ITIC and the HOMO/LUMO energy levels are shifted downward.^112,113^ Gao et al. reported the introduction of three (**7-3**, IT-3F)^111^ fluorine atoms on INCN groups, which has the same effect on the energy levels as for **7-2** and **7-4**. Regarding the photovoltaic parameters, the *J*_SC_, FF, and PCE are increased significantly, whereas the *V*_OC_ is decreased, compared to ITIC in combination with PM6.^108,111^ In contrast, the asymmetric molecule **7-5** (*a*-IT-2F),^114^ where fluorine is only attached at one end group, shows a similar PCE, lower *V*_OC_ and FF, as well as higher *J*_SC_.^106,114^ When one chlorine (INCN-Cl) or two chlorines (INCN-2Cl) are attached to INCN (**7-6**, ITIC-2Cl and **7-7**, ITIC-4Cl, respectively),^115^ it has a similar effect on the photovoltaic parameters and the energy levels as the addition of fluorine.^108,115^ Lai et al. investigated an asymmetric acceptor unit substitution with fluorine and/or chlorine atoms. Upon the introduction of two fluorines on one and one chlorine on the other INCN (**7-8**, ITIC-γCl-2F),^116^ the energy levels stayed in the same range as ITIC and are therefore higher than those for the fluorinated compounds **7-2**–**7-4**. Solar cells with PM6 as a donor polymer are reaching 12%.

When two chlorines are attached only on one side as in **7-10** (α-ITIC-2Cl),^117^ the HOMO level is upshifted compared to ITIC or compared to the fluorinated counterpart **7-5**. With the polymer donor PM6, solar cells reach a higher efficiency (12%) than **7-5** with PBDB-T (10%).^114,117^ In **7-9** (ITIC-2Cl-β),^117^ one chlorine is attached on each side on a specific position on the phenyl ring. Comparing with molecule **7-6**, where the chlorine atom can be present anywhere on the phenyl ring, the HOMO/LUMO energy levels lie higher. With PM6 as the polymer donor and the same solvent additive, **7-6** possesses a higher efficiency of 13.2%, due to a much higher FF of 75% than **7-9**.^115,117^ Qu et al. showed that the photovoltaic parameters differ when bromine atoms are substituted at different positions on the phenyl ring. The group synthesized two compounds, **7-11** (ITIC-2Br-m) and **7-12** (ITIC-2Br-γ),^118^ where **7-12** has the bromine located on one position on the INCN phenyl ring and **7-11** consists of isomers, where the bromine atom can be located on different positions (INCN-Br). Comparing these materials to each other, **7-11** has a significantly lower PCE of 10.9% than **7-12** with 12.1%.^118^ The reason for the great difference in efficiency could be the higher absorption coefficient and consequently the higher EQE, which increased the *J*_SC_ value. In addition, the charge dissociation and recombination for both molecules in blend film were measured, implying a better dissociation and less recombination for **7-12**. When halogens are added to the INCN group, the absorption maximum in film as well as in solution is red-shifted. In the case of bromine substituents, the shift is notably increased in film but about the same in solution.

A methyl group on the INCN acceptor unit (**7-13**, IT-M)^119^ shifts the energy levels upward but gives a similar band gap as ITIC.^119−121^ Like in **7-13**, methyl groups are added to INCN in **7-16** (IT-DM),^112^ with the difference that two groups instead of one group are substituted on each side. The additional methyl groups affect the LUMO energy level by shifting it upward, consequently extending the band gap. When the acceptor **7-13** is combined with the polymer PDTF-TZNT, the photovoltaic parameters are generally lower compared to ITIC, except for the FF, which reveals a value of 73%.^119^**7-16** with the polymer J71 reveals a higher *V*_OC_ and PCE but lower *J*_SC_ and FF compared to **7-13**.^112^ Yao et al. introduced a trifluoromethyl group on the end groups (**7-14**, IT-CF_3_)^122^ with the effect that the LUMO energy level is downshifted, leading to a smaller band gap. The addition of trifluoromethyl groups also leads to a higher red-shift than simple fluorine atoms; here observed in film and solution.^112^ The solar cell parameters, compared to the fluorinated compounds (**7-2**–**7-4**), showed a lower *V*_OC_, higher *J*_SC_, higher FF, and comparable PCE.^111,122^**7-15** (ITEN)^108^ was designed and synthesized by Yu et al. through adding ethynyl groups to extend the π-conjugation of the acceptors. The molecule possesses similar energy levels and a similar band gap as ITIC, but combined with PM6, it shows a better performance in solar cells (PCE 10.9%).^108^

Hao et al. strived to combine the advantages of the addition of methyl groups and fluorine on the end group, resulting in a molecule with two methyl groups and two fluorines on different positions of the terminal groups (**7-17**, ITCF).^112^ An electron-withdrawing group, like fluorine, should narrow the optical band gap of the resulting acceptor, whereas the electron-donating group, like a methyl group, should heighten the LUMO energy level. Compared to ITIC, the HOMO/LUMO energy levels are downshifted equally and the photovoltaic parameters are enhanced compared to the ITIC/J71 blend.^112^**7-17** achieved a similar PCE of 13.3% as **7-14**, which lies between the efficiencies of **7-2** and **7-4** (see Table 1). The replacement of fluorine substituents in the asymmetric structure of **7-5** with electron-donating methoxy groups leads to the structure **7-18** (*a*-IT-2OM).^114^ In contrast to **7-5**, **7-18** has higher energy levels and a higher band gap. Solar cells with PBDB-T as a donor gave an improved *V*_OC_ of 0.93 V and FF of 72% but a lower *J*_SC_ of 18.1 mA cm^–2^. However, this leads to PCE values of 12.1%, which are higher than those of devices with **7-5** as well as with ITIC (**7-1**) and the same donor.^114^

The NFAs **7-19** (IT-OH)^123^ and **7-20** (IT-DOH)^123^ contain hydroxy groups on one and two INCN units, respectively. Compared to ITIC, the HOMO/LUMO energy levels are not affected by the additional group. Moreover, the absorption spectra in solution show the same maxima, but in thin films, the maxima are red-shifted for **7-19** and **7-20**. Photovoltaic parameters reveal the same (**7-19**, 10.4%) or higher (**7-20**, 11.0%) efficiency than ITIC when combined with the same donor PBDB-T. When the NFAs are blended with PM6, the efficiency increases for **7-19** to 11.2% and for **7-20** to 12.5%.^106,123^

The substitution of the dicyanomethylene group of the INCN end group with an oxo group leads to the structures **7-21**–**7-23**.^124,125^ In addition, fluorine and chlorine were introduced on the phenyl ring. Comparing the molecules with their INCN counterparts, **7-21** (IO-4H)^125^ without halogenation shows a downshifted HOMO and upshifted LUMO level, widening the band gap to 1.88 eV, while the energy levels of both **7-22** (IO-4F)^125^ and **7-23** (IO-4Cl)^124^ are downshifted further due to their halogenation. Solar cells of PBDB-T/**7-21** obtained a high *V*_OC_ value of 1.12 V but low values of *J*_SC_ and FF, resulting in an overall PCE of 2.86%, whereas the efficiency of PBDB-T/**7-22** and PM6/**7-23** was higher (8.06 and 9.80%, respectively). All three acceptors show higher *V*_OC_’s compared to their INCN-based counterparts combined with the polymer PBDB-T;^124−126^ however, **7-21** and **7-22** show very poor electron mobilities in the blend film, which may be the reason for the lower performance compared to their parent compounds.

An alternative strategy to modify the INCN acceptor group, is changing the aromatic ring system from benzene to thiophene, resulting in 2-(6-oxo-5,6-dihydro-4*H*-cyclopenta[*c*]thiophen-4-ylidene)malononitrile (CPTCN)-based end groups. Thereby, the sulfur can be oriented in different directions depending on the orientation of the thiophene ring. In the case of molecule **7-24** (ITCPTC)^127^ without any substituents on their acceptor units, the change in the structure leads to a similar optical band gap but lowered energy levels compared to ITIC (**7-1**). Solar cells with **7-24** lead to PCEs up to 12.3%; also, the *J*_SC_ and FF are increased compared with ITIC-based devices.^127^ Based on the structure of **7-24,** different substituents such as chlorine (**7-25**, ITC-2Cl),^128^ bromine (**7-26**, ITC-2Br2),^129^ or methyl (**7-27**, MeIC)^127^ were introduced on the thiophene ring. As expected, the attachment of halogens generally lowers the energy levels compared to **7-24**, while the methyl group has the opposite effect. Blended with PM6, the PCE of both acceptors (**7-25** and **7-26**) exceeded 13% and showed a *V*_OC_ slightly lower than 1 V.^128,129^**7-27** was also combined with PM6 and gave a *V*_OC_ approaching 1 V and a PCE of 13.0%.^127^ The attachment of two methyl groups on the acceptor unit leads to molecule **7-28** (ITCT-DM).^130^ This adjustment upshifts the HOMO energy level further but leads to a similar optical band gap as in **7-27**. Combined with PBDB-T, **7-28** shows lower PV parameters than **7-27** with a PCE of 10.6%.^127,130^ Molecule **7-29** (IDTC)^131^ contains similar acceptor units as **7-24**, with the difference in the position of the S in the thiophene ring. This change in the position affects the energy levels by shifting them upward and widening the optical band gap to 1.68 eV. PV parameters show, compared to **7-24** with the same polymer donor PBDB-T, a higher *V*_OC_ and otherwise slightly lower values of *J*_SC_, FF, and PCE.^131,132^ Luo et al. investigated three isomeric structures containing a bromine substituent on the thiophene ring in the end group (**7-26**, **7-30**, **7-31**).^129^ If the end group is asymmetric regarding the sulfur in the thiophene ring, the LUMO levels are slightly higher and the HOMO levels slightly lower than in the symmetric molecule. Following this, the band gap is widened (i.e., 1.59 eV for **7-26** and 1.73 eV for **7-30**, ITC-2Br). The *V*_OC_ is reaching 1 V with lower *J*_SC_ and FF; the PCE is also lower and settled between 10.9 and 11.9%. The better PCE is achieved with **7-31** (ITC-2Br1), where the bromine is on position 3 in the 5-membered ring.^129^**7-27** with a methyl group on position 3 and sulfur on position 2 possesses a smaller band gap and better photovoltaic parameters than **7-32** (ITCCM-O)^133^ with methyl on position 2 and sulfur in position 3, with the exception of the *V*_OC_. The difference between these two molecules is not only the position of the sulfur and the methyl group but also that the malononitrile group was substituted with an oxygen atom. This leads to very poor photovoltaic parameters except for the *V*_OC_, which reached an outstanding 1.34 V.^127,133^ These results are consistent with **7-21**–**7-23**, where the band gap is also enlarged and the solar cells achieved a *V*_OC_ of over 1 V.

Deng et al. investigated the extension of the acceptor units in **7-29** by one (**7-33**, IDTTC)^131^ or two (**7-34**, IDTTTC)^131^ thiophene units. The extension heightens the HOMO/LUMO energy levels while reducing the optical band gap to 1.58 and 1.55 eV, respectively. However, the introduction of one more thiophene unit as in **7-33** leads to a higher *J*_SC_, FF, and PCE of 18.3 mA cm^–2^, 73%, and 13.5%, respectively, compared to **7-29**, whereas two additional thiophenes (**7-34**) decrease the PCE to 6.46%. The reason for that lies in the coarser morphology of these donor/acceptor blend films.^131^ The substitution of the outer thiophene ring in **7-34** with a phenyl ring leads to structure **7-35** (ITBC)^134^ and affects the energy levels by lowering them again. Compared to **7-34**, the PV parameters of **7-35** with PBDB-T are improved to a *J*_SC_ of 19.9 mA cm^–2^, a FF of 65%, a PCE of 12.1%, and a decreased *V*_OC_ of 0.94 V.^131,134^

In acceptor **7-36** (ITBC),^138^ the oxo group of INCN was replaced with a SO_2_ functionality. This downshifted the energy levels but maintained a similar band gap as ITIC. Combined with the polymer FTAZ, it leads to a quite low *V*_OC_, *J*_SC_, and FF with a PCE of 4.17%.^138^ In molecule **7-37** (IDTT-R),^140^ a 2-(1,1-dicyanomethylene)rhodanine (RCN) acceptor unit is introduced, which heightens the HOMO/LUMO energy levels compared to ITIC. Due to a blue-shift in the absorption of **7-37**, the optical band gap is widened to 1.84 eV. In solar cell devices, the acceptor was combined with P3HT, which leads to poor PV parameters with the highest efficiency being 0.43%.

He et al. added a common acceptor group, a diethyl thiobarbituric acid (TBA), to the IDTT core unit. This has a pronounced influence on the LUMO energy level of **7-38** (IDTT-T),^107^ which is shifted upward. The PV parameters are improved compared to ITIC in combination with the same polymer PTB7-Th. **7-38** and ITIC show *V*_OC_s of 1.02 and 0.83 V, *J*_SC_s of 18.0 and 14.4 mA cm^–2^, similar FFs of 65 and 66%, as well as PCEs of 11.8 and 7.80%, respectively. **7-38** also has an outstanding electron mobility when blended with PTB7-Th.^107^ Xiao et al. designed similar molecules to **7-38**, with differences in the *N*-annulated side chains of the TBA group (**7-39**–**7-42**). Besides the ethyl chains in **7-38**, they introduced methyl (**7-39**, IDTT-TBA (a)),^141^ benzyl (**7-40**, IDTT-TBA (c)),^141^ octyl (**7-41**, IDTT-TBA (d)),^141^ and ethylhexyl (**7-42**, IDTT-TBA (e))^141^ chains. All five molecules (**7-38**–**7-42**) have comparable HOMO energy levels ranging from −5.42 to −5.51 eV and optical band gaps between 1.87 and 1.89 eV. Photovoltaic devices were built with PTB7-Th as a donor material which gave very different results on each acceptor. The acceptor with branched ethylhexyl chains (**7-42**) shows the lowest performance with an efficiency of 2.52%. While the PCE increased to 4.69% upon the introduction of octyl chains (**7-41**) and to 5.44% with methyl groups (**7-39**), a significant change in efficiency is observed when benzyl (**7-40**) and ethyl groups (**7-38**) are used, enhancing the PCE to 7.41 and 8.77%, respectively. The reason for these differences lies in the molecular packing in combination with the polymer donor and may be enhanced under the use of other donor materials.^141^

Naphthalene monoimide (NMI) was used as a terminal group in structure **7-43** (NIDT),^142^ which shifts the energy levels up, resulting in an optical band gap of 1.85 eV. Combined with PBDB-T, this molecule gives over 1 V in OSCs but a smaller *J*_SC_, FF, and PCE than ITIC with the same donor material.^142^ Lai et al. synthesized a series of asymmetric molecules (**7-44**–**7-46**) with an INCN on one side and a CPTCN end group on the other. They differ in the substitution of the number and position of chlorine atoms on their INCN unit. The molecules **7-44** (ITIC-Cl-δ-Th)^143^ and **7-45** (ITIC-Cl-γ-Th)^143^ possess one chlorine atom in the δ-position and the γ-position, respectively, whereas **7-46** (ITIC-2Cl-Th)^143^ contains two chlorines on the acceptor unit. These three compounds show similar HOMO energy levels, which are, compared to ITIC, heightened but differ in their LUMO levels being −3.70 eV for **7-44**, −3.66 eV for **7-45**, and −3.74 eV for **7-46**. In solar cells blended with PM6 as a donor polymer, these molecules have a *V*_OC_ approaching 0.90 V, a FF of 72–73%, and efficiencies over 11%. The highest PCE was reached by **7-44** with 12.3%, which shows that the position of the halogen on the end group is crucial for the device performance.^143^ The acceptor **7-47** (A_2_)^144^ is also asymmetric, having an INCN end group on one side and a fullerene (C_60_) on the other. The introduction of a fullerene raised the HOMO/LUMO levels while retaining a similar band gap. In contrast, the photovoltaic parameters are low except for the *V*_OC_ and they show a moderate PCE of 4.52%.^144^

### 3.2 Impact of Side Chains

**Figure 6:**
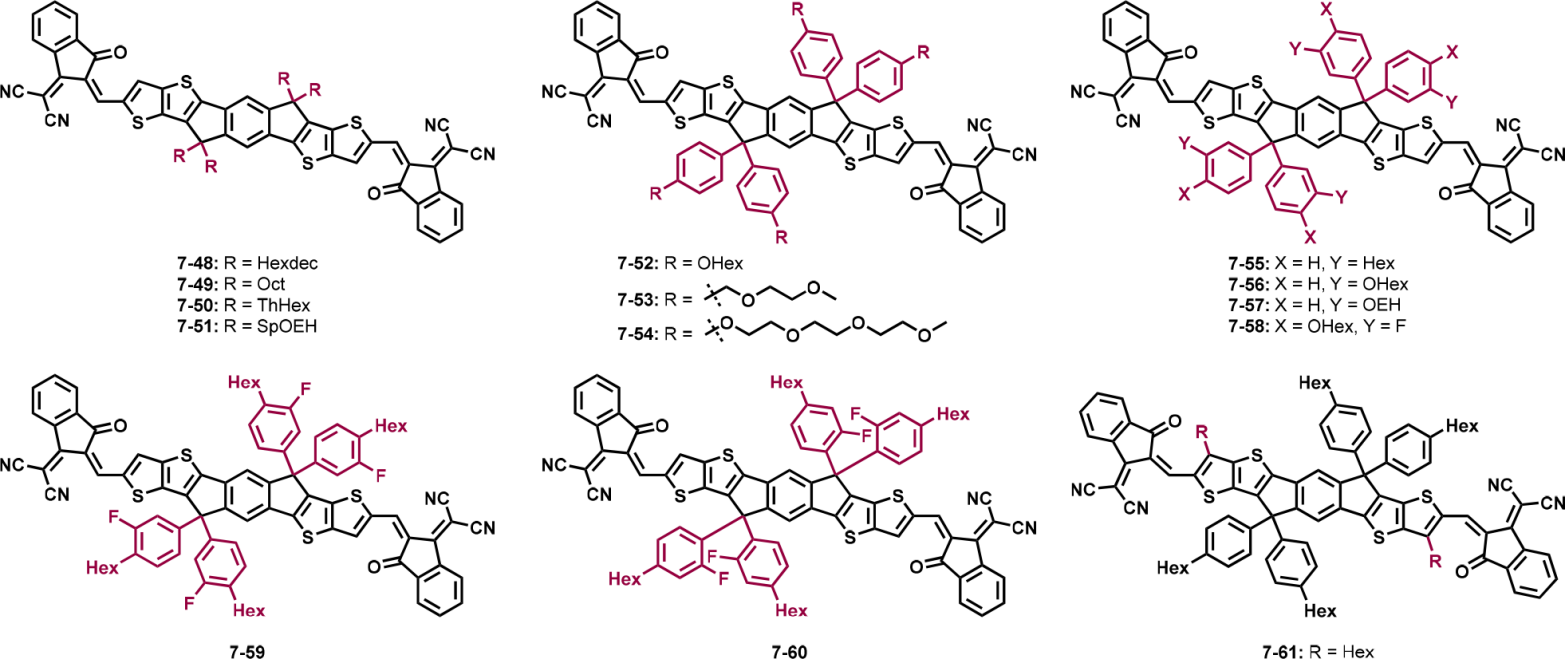
Structures of non-fullerene acceptors with the same donor backbone and accepting units and different side chains.

Side chain engineering is used to ensure the solubility of a compound, to improve molecular packing and the film morphology, and thus has an immense effect on the photovoltaic properties. The replacement of the *p*-hexylphenyl side chains in ITIC with alternatives leads to the structures **7-48**–**7-61** (see Figure 6 and Table 2). In general, the overall influence on the HOMO/LUMO energy levels is small and their values are similar or slightly downshifted compared to ITIC; consequently, also the optical band gap is in the same range.

**Table 2:** Optical, Electrical, and Photovoltaic Properties of Non-Fullerene Acceptors **7-48**–**7-61**

NFA	original name	HOMO^a^ (eV)	LUMO^a^ (eV)	*E*_g_^opt^ (eV)	donor	D:A ratio	*V*_OC_ (V)	*J*_SC_(mA cm^–2^)	FF (%)	PCE (%)	μ_e_^c^(cm^2^ V^–1^ s^–1^)	ref.
**7-48**	IDTTIC	–5.68	–3.84	1.51	PBDB-T	1:1	0.92	17.3	70	11.2	-/1.2 × 10^–4^	(145)
**7-49**	C8-ITIC	–5.63	–3.91	1.55	PFBDB-T	1:1.25	0.94	19.6	72	13.2	-/6.9 × 10^–4^	(146)
**7-50**	ITIC-Th	–5.66	–3.93		PTFB-O	1:1.5	0.92	16.7	68	10.9	-/4.5 × 10^–4^	(147)
	ITIC-Th	–5.65	–4.05^b^	1.60	P(Cl)	1:1.25	0.90	18.6	68	11.4	-/5.1 × 10^–4^	(157)
**7-51**	sp-mOEh-ITIC	–5.67	–4.06^b^	1.61	PBDB-T	1:1	0.87	12.1	61	6.44		(148)
**7-52**	POIT-IC	–5.57	–3.92	1.58	PM6	1:1	1.04	16.1	60	10.1	1.8 × 10^–4^/6.4 × 10^–4^	(149)
**7-53**	ITIC-OE	–5.67	–4.03	1.57	PBDB-T	1:1	0.85	14.8	67	8.50	4.8 × 10^–4^/1.2 × 10^–5^	(106)
**7-54**	ITIC-OEG	–5.39	–3.99	1.54	PPDT2FBT	1:1	0.90	3.56	49	1.58	1.9 × 10^–5^/-	(150)
**7-55**	*m*-ITIC	–5.68	–3.95	1.59	PBDS-T	1:1.5	0.99	16.8	62	10.3		(151)
**7-56**	mO-ITIC	–5.50	–3.74	1.63	PTZ-DO	1:1	0.92	15.2	66	9.28		(152)
	*m*-ITIC-O-H	–5.25	–3.65^b^	1.60	PBDB-T	1:1	0.85	16.0	70	9.55	-/9.4 × 10^–6^	(153)
**7-57**	*m*-ITIC-O-EH	–5.25	–3.63^b^	1.62	PBDB-T	1:1	0.88	15.9	68	9.77	-/7.1 × 10^–6^	(153)
**7-58**	FpO-ITIC	–5.61	–3.72	1.64	PTZ-DO	1:1	0.88	12.6	61	6.69		(152)
**7-59**	*o*F-ITIC	–5.73	–3.93	1.63	PBTIBDTT	1:1.2	0.94	13.5	71	9.01	3.6 × 10^–4^/3.8 × 10^–4^	(154)
	*m*-F-ITIC	–5.69	–3.96	1.62	PBDB-T	1.3:1	0.88	15.8	64	8.90	9.4 × 10^–5^/3.7 × 10^–5^	(155)
**7-60**	*m*F-ITIC	–5.66	–3.88	1.60	PBTIBDTT	1:1	0.96	14.8	67	9.55	3.0 × 10^–4^/3.0 × 10^–4^	(154)
	*o*-F-ITIC	–5.66	–3.94	1.58	PBDB-T	1.3:1	0.92	18.1	67	11.1	4.8 × 10^–4^/4.1 × 10^–4^	(155)
**7-61**	ITC6-IC	–5.72	–3.78	1.60	PBDB-T	1:1	0.94	16.2	71	10.9	-/6.6 × 10^–4^	(156)

a Obtained from the oxidation/reduction potential of the CV measurement if not otherwise stated.

b HOMO/LUMO energy levels obtained from the LUMO/HOMO levels determined by CV and the optical band gap.

c Determined via the SCLC technique from the neat acceptor/donor:acceptor blend films if not otherwise stated.

Structures **7-48** (IDTTIC)^145^ and **7-49** (C8-ITIC)^146^ are the only acceptors with pure alkyl chains (C16 and C8, respectively) which reduce the band gaps significantly to 1.51 and 1.55 eV. The C16 chains give **7-48** a high crystallinity; however, in the blend film with PBDB-T, this crystallinity is largely suppressed. Compared to ITIC, solar cells based on **7-48** and PBDB-T show an improved PCE of 11.2% and enhanced *V*_OC_ and *J*_SC_.^145^ When the alkyl chains only contain eight C atoms (**7-49**), the PCE of solar cells with PBDB-T is increased to 11.9%, and in comparison to **7-48**, the *V*_OC_, *J*_SC_, and FF decreased slightly.^145,146^ Another prominent compound is ITIC-Th (**7-50**)^147^ in which the phenyl ring is substituted by a thiophene. Compared to ITIC, the energy levels are downshifted to −5.66 and −3.93 eV. Different polymers were combined with **7-50**, showing that they greatly influence the crystallinity within the blend film. The molecular ordering is stronger in the PTFB-O/ITIC-Th blend film, which is also consistent with the PV parameters. PTFB-O/ITIC-Th blended active layers give a PCE over 10%, while all other tested polymers barely reached over 8%.^147^

Sung et al. added fluorene units with alkyl chains to the seven-ring backbone, giving **7-51** (sp-mOEh-ITIC)^148^ with two spirobifluorenes implemented in the acceptor. The solar cell parameters are better with chlorobenzene than with *o*-xylene; the highest PCE reached was 6.44%. GIWAXS measurements were done with the neat film of the acceptor, giving a wide halo pattern with a mixture of face-on and edge-on orientations.^148^

The introduction of an alkoxy side chain on the phenyl group (**7-52**, POIT-IC)^149^ leads to good PV parameters with a *V*_OC_ over 1 V and a PCE above 10%. Upon measuring the crystallinity in pure films, the acceptor shows mixed face-on and edge-on orientations. Combined with PM6, the film favors the face-on orientation resulting from the arrangement of the polymer. PM6 also suppresses the crystallinity of **7-52** due to their good miscibility.^149^ Oligoethylene glycol side chains were introduced in acceptors **7-53** (ITIC-OE)^106^ and **7-54** (ITIC-OEG).^150^ They show a lower PCE than ITIC when blended with PBDB-T and PPDT2FBT, respectively. Thereby, **7-54** only reached PCEs of approx. 1.5%, which may be due to the usage of the polymer PPDT2FBT, as **7-53** led to an efficiency above 8%. Another reason could be the chain length of the oligoethylene glycol unit. The acceptors and polymers as well as ITIC itself were investigated with GIWAXS. ITIC, PPDT2FBT, and PBDB-T show strong lamellar stacking peaks in the in-plane and π–π stacking peaks in the out-of-plane direction. Both, **7-53** and **7-54** exhibit π–π stacking peaks in the out-of-plane direction, whereas the peak of **7-53** is much weaker compared to ITIC, which indicates less crystallinity. In contrast, **7-54** has a sharply resolved π–π stacking peak, suggesting a tighter stacking and the coherence length of **7-54** implying a promotion of the intermolecular packing. The same is present in the blend films. Acceptor **7-54** suffers from poor electron mobility in neat film, and **7-53** shows a low mobility in the blend film.^106,150^

Chen et al. investigated the influence of the hexyl chain position on the phenyl ring of the side chain (**7-55**, *m*-ITIC).^151^ They added the alkyl chain at the meta position which leads, combined with PBDS-T, to rather good photovoltaic properties in the same range as ITIC.^151^ Exchanging the alkyl side chain in the meta position with an alkoxy chain (**7-56**, mO-ITIC)^164^ leads to slightly blue-shifted absorption maxima and similar energy levels as ITIC. However, also the PV parameters are slightly lower. Compared to **7-52**, where the alkoxy side chains are located in the para position, the energy levels are slightly upshifted and the band gap widened. The absorption maxima are also slightly blue-shifted. The PCE achieved for **7-56** is 9.3% and that for **7-52** is 10.1%; however, another conjugated polymer was used. Under the same conditions and the use of the same polymer, the acceptor **7-52** reached only 9.0% efficiency. Both acceptors were investigated using GIWAXS, giving an idea about the crystallinity, which is higher for **7-56** than for **7-52**.^149,152^ Lee et al. investigated molecule **7-56** and reported another acceptor, where the hexyloxy chains are replaced with ethylhexyloxy chains (**7-57**, *m*-ITIC-O-EH). Blended with PBDB-T as a donor, solar cells show comparable parameters with **7-56** and **7-57**. The higher PCE was achieved by **7-57** with 9.77% compared to **7-56** with 9.55%. However, the higher electron mobility in the blend film was reached by **7-56**.^153^ Upon the introduction of a fluorine atom in the meta-position (**7-58**, FpO-ITIC),^152^ the device parameters decreased further, reaching only 6.69% efficiency. When comparing the acceptor to **7-52**, the introduction of fluorine blue-shifts the absorption maxima of the NFA and downshifts the energy levels, as was expected for halogens.^149,152^ The neat film of **7-58** has a broad lamellar stacking peak indicating weak side chain packing; the absence of π–π stacking peaks in both directions suggests a weak crystallinity compared to ITIC, just like the *para*-substituted **7-52**. Acceptor **7-56** shows a slightly higher crystallinity in pure film than **7-58**.^152^ The additional fluorine in the side chain has no effect on the crystallinity of the NFA.

Finally, the influence of fluorine on the phenyl ring of the phenylhexyl side chain was investigated by introducing fluorine in either the meta (**7-59**, *o*F-ITIC/*m*-F-ITIC)^154,155^ or the ortho (**7-60**, *m*F-ITIC/*o*-F-ITIC)^154,155^ position to the backbone. Both molecules show similar energy levels and optical band gaps (1.58–1.63 eV); however, the efficiency of **7-60** is increased to 11.1% compared to **7-59** (PCE: 8.9%) when PBDB-T is used as a donor.^155^ Compared to ITIC, the absorption maxima of both acceptors are slightly blue-shifted, leading to a slightly higher band gap compared to ITIC, and the electron mobility in neat film is higher for ITIC than for **7-59** and **7-60**.^154,155^**7-61** (ITC6-IC)^156^ has a backbone and side chains similar to ITIC but additional hexyl chains on the outer thiophene rings of the donor unit. Compared to ITIC, the HOMO energy level is downshifted and the LUMO level and optical band gap are similar. Combined with PBDB-T, the solar cells show a *V*_OC_ of 0.94 V, a *J*_SC_ of 16.2 mA cm^–2^, a FF of 71%, and a PCE of 10.9%.^156^

### 3.3 Combined Effects in IDTT-Based Acceptors

**Figure 7:**
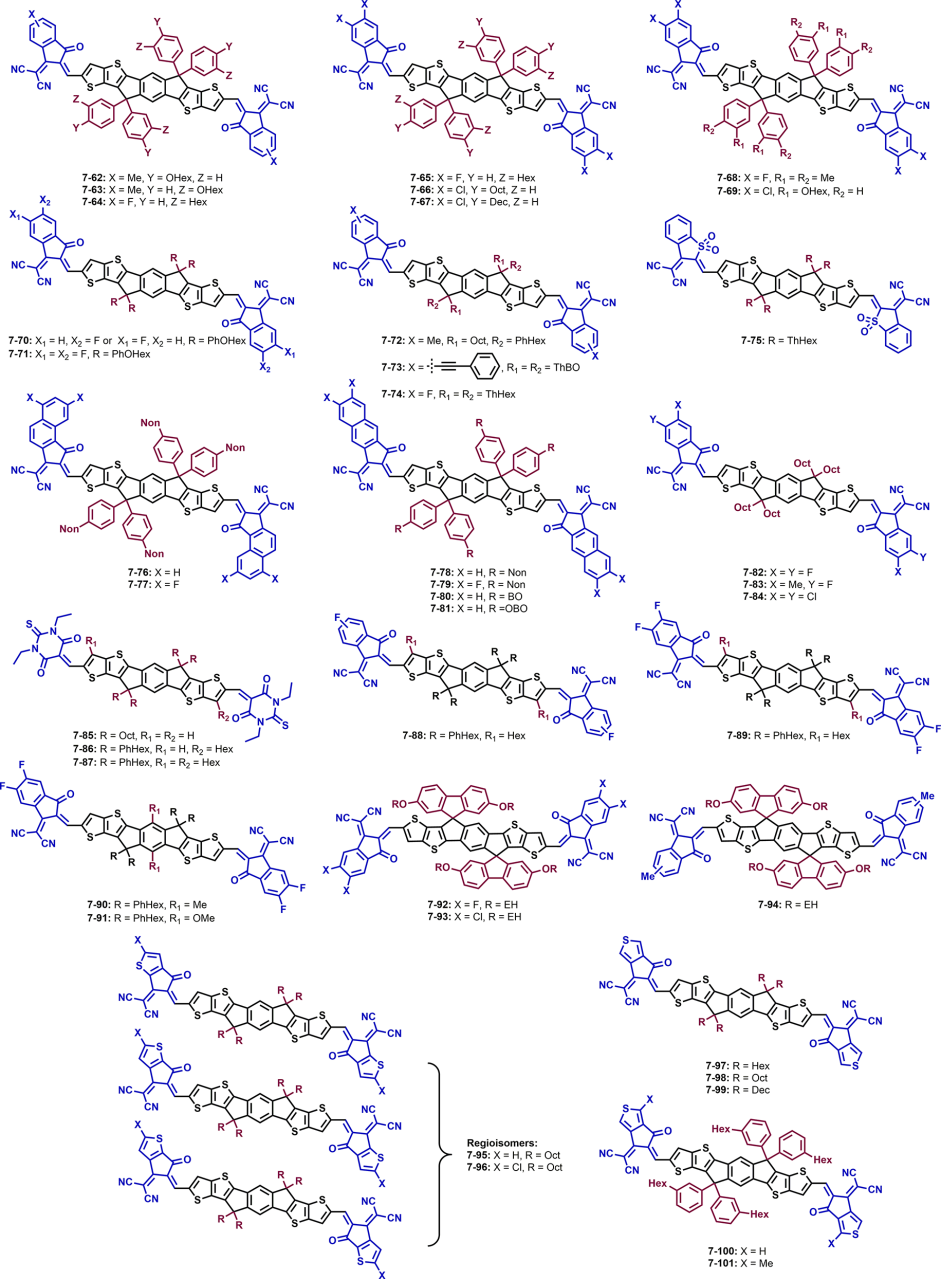
Structures of non-fullerene acceptors with an IDTT core and different side chains and end groups.

The combination of modifying the side chain and variation of the acceptor end groups even further increases the structural diversity of IDTT-based acceptors, as shown in Figure 7. For example, the combination of the structure **7-13**—ITIC methylated on the INCN-acceptor units—with variation in the end groups on the central core—hexyloxy groups in the para or meta position—leads to the structures **7-62** (POIT-M)^120^ and **7-63** (MOIT-M).^120^ As expected, their electrochemical and optical properties are comparable with **7-13**. A comparison of solar cells using these three acceptors and PTZ1 as donor polymers leads to similar *V*_OC_ values of approx. 0.97 V for all three acceptors, but the *J*_SC_ as well as the FF increases from 14.2 mA cm^–2^ and 62% for **7-13** to 15.4 mA cm^–2^ and 65% for **7-62** and to 17.5 mA cm^–2^ and 69% for **7-63** (see also Table 3). Consequently, **7-63**, with the alkoxy group in the meta position, leads to the highest PCE of 11.6% (compared to 9.10% for **7-13** and 9.70% for **7-62**). GIWAXS data of the three acceptors indicate that **7-63** has a stronger intermolecular π–π stacking interaction and thus has a higher crystallinity and more ordered molecular orientation than **7-13** and **7-62**. Moreover, the electron mobility of **7-63** is higher, which is also an indicator of why this acceptor works better than the other two.^120^ In very similar approaches, the influence of side chain variation on the IDTT core on the fluorinated ITIC analogues **7-2** and **7-4** was investigated. This leads to structures **7-64** (*m-*ITIC-2F)^158^ and **7-65** (*m*-ITIC-4F),^158^ where the hexyl side chains were placed in meta position on the phenyl ring. The HOMO and LUMO energy levels for **7-64** and **7-65** are −5.73 and −3.95 eV and −5.73 and −4.02 eV, respectively, which are lower than the values for structures **7-2** and **7-4**. The fluorination leads to a reduction of the optical band gap to 1.56 and 1.53 eV for **7-64** and **7-65** compared to 1.59 eV for the unfluorinated **7-55**. Organic solar cells with PTQ10 as a polymer donor exhibited efficiency values of 12.5%. **7-64** has a higher degree of self-organization and molecular packing than **7-65**, which also agrees with the mobility data of the two materials.^158^

**Table 3:** Optical, Electrical, and Photovoltaic Properties of Non-Fullerene Acceptors **7-62**–**7-101**

NFA	original name	HOMO^a^ (eV)	LUMO^a^ (eV)	*E*_g_^opt^ (eV)	donor	D:A ratio	*V*_OC_ (V)	*J*_SC_(mA cm^–2^)	FF (%)	PCE (%)	μ_e_^d^(cm^2^ V^–1^ s^–1^)	ref.
**7-62**	POIT-M	–5.60	–3.87	1.60	PTZ1	1:1	0.97	15.4	65	9.70	6.2 × 10^–4^/3.7 × 10^–4^	(120)
**7-63**	MOIT-M	–5.61	–3.89	1.60	PTZ1	1:1	0.96	17.5	69	11.6	8.3 × 10^–4^/6.0 × 10^–4^	(120)
**7-64**	*m*-ITIC-2F	–5.73	–3.95	1.56	PTQ10	1:1	0.96	19.0	69	12.5	3.3 × 10^–4^/4.0 × 10^–4^	(158)
**7-65**	*m*-ITIC-4F	–5.73	–4.02	1.53	PTQ10	1:1	0.90	19.8	70	12.5	2.9 × 10^–4^/5.1 × 10^–4^	(158)
**7-66**	IT-4Cl-C8	–5.94	–4.10		PM6	1:1	0.83	20.4	76	12.9		(159)
**7-67**	IT-4Cl-C10	–5.95	–4.08		PM6	1:1	0.87	20.3	77	13.5		(159)
**7-68**	IDMIC-4F	–5.46	–3.83		PM6	1:1.2	0.89	16.6	61	9.40		(160)
**7-69**	*m*-ITIC-OR-4Cl	–5.78	–4.05	1.50	PTQ10	1:1.5	0.87	21.8	67	12.7	-/3.4 × 10^–4^	(161)
**7-70**	POIT-IC2F	–5.60	–3.98	1.55	PM6	1:1	0.97	18.6	69	12.4	2.2 × 10^–4^/6.6 × 10^–4^	(149)
**7-71**	POIT-IC4F	–5.65	–4.07	1.49	PM6	1:1	0.91	20.9	73	13.8	4.2 × 10^–4^/8.0 × 10^–4^	(149)
**7-72**	IDTT-OB	–5.59	–3.88	1.59	PBDB-T	1:1	0.91	16.4	74	11.2	-/6.5 × 10^–4^	(162)
**7-73**	ITPN	–5.69	–3.91	1.60	PM6		0.99	17.5	73	12.6	4.4 × 10^–4^/7.5 × 10^–4^	(108)
**7-74**	ITIC-Th1	–5.70	–4.12		PBDB-T	1:1	0.88	19.6	70	12.0	-/9.8 × 10^–3^	(135)
**7-75**	ITThBC	–5.71	–4.15	1.58	PM6	1:1	0.87	13.4	65	7.59	-/9.4 × 10^–5^	(139)
**7-76**	ITzN-C9	–5.62	–3.78	1.65	PM6	1:1	1.05	14.1	64	9.51	0.3 × 10^–4^/3.5 × 10^–4^	(165)
**7-77**	ITzN-F4	–6.00	–4.20	1.58	PM6	1:1	0.92	17.5	68	10.9	-/3.5 × 10^–4^	(166)
**7-78**	ITN-C9	–5.78	–3.92	1.54	PM6	1:1	0.92	15.7	65	9.33	2.3 × 10^–4^/4.5 × 10^–4^	(165)
**7-79**	ITN-F4	–6.10	–4.30	1.49	PM6	1:0.8	0.82	19.6	67	10.7	-/1.9 × 10^–4^	(166)
**7-80**	IDTT-BH	–5.42	–3.86	1.54	J71	1:1.4	0.90	17.8	69	11.1	-/1.6 × 10^–4^	(164)
**7-81**	IDTT-OBH	–5.41	–3.86	1.57	PBDB-T	1:0.8	0.87	17.5	72	10.9	-/9.8 × 10^–5^	(164)
**7-82**	C8-IT-4F	–5.57	–4.04	1.47	PM7	1:1	0.82	22.7	77	14.3	1.3 × 10^–3^/1.1 × 10^–3^	(167)
**7-83**	MF2	–5.61	–3.93	1.49	PM7	1:1	0.96	19.2	75	13.7	7.7 × 10^–4^/2.6 × 10^–4^	(168)
**7-84**	C8IDTT-4Cl	–5.76	–4.29	1.43	PBDT-TPD	1:1	0.67	19.1	59	7.55	-/3.6 × 10^–4^	(169)
**7-85**	IDTTA	–5.81^c^	–3.97	1.75	PBDB-T	1:1	0.98	15.8	69	10.8	1.8 × 10^–4^/1.0 × 10^–4^	(170)
**7-86**	C_6_-IDTT-T	–5.71	–3.78	1.82	PTB7-Th	1:1.1	1.05	14.4	56	8.51	1.3 × 10^–4^/8.0 × 10^–5^	(171)
**7-87**	2C_6_-IDTT-T	–5.74	–3.72	1.84	PTB7-Th	1:1.1	1.07	13.3	53	7.52	7.3 × 10^–5^/4.2 × 10^–5^	(171)
**7-88**	ITC6-2F	–5.73	–3.81	1.58	PBDB-T	1:1	0.86	17.8	73	11.2	-/1.6 × 10^–5^	(156)
**7-89**	ITC6-4F	–5.74	–3.84	1.54	PBDB-T	1:1	0.78	18.6	73	10.5	-/2.7 × 10^–4^	(156)
**7-90**	IM-4F	–5.69	–4.19	1.46	PM6	1:1	0.88	22.1	73	14.2	-/5.2 × 10^–4^	(172)
**7-91**	IOM-4F	–5.72	–4.27	1.48	PM6	1:1	0.86	21.7	72	13.4	-/4.8 × 10^–4^	(172)
**7-92**	sp-mOEh-ITIC-F	–5.77	–4.23^b^	1.54	PBDB-T	1:1	0.68	13.7	62	5.79		(148)
**7-93**	sp-mOEh-ITIC-Cl	–5.74	–4.22^b^	1.52	PBDB-T	1:1	0.69	14.2	59	5.78		(148)
**7-94**	sp-mOEh-ITIC-M	–5.69	–4.07^b^	1.62	PBDB-T	1:1	0.90	11.4	58	5.96		(148)
**7-95**	C8-ITCC	–5.45	–3.85	1.66	PM6	1:1	1.04	16.1	63	10.8	-/2.7 × 10^–4^	(173)
**7-96**	C8-ITCC-Cl	–5.50	–3.93	1.58	PM6	1:1	0.95	17.9	73	12.7	-/6.7 × 10^–4^	(173)
**7-97**	IDTT-C6-TIC	–5.55	–3.99	1.60	PBT1-C	1:1.3	0.85	17.0	67	10.0	1.2 × 10^–3^/2.0 × 10^–4^	(174)
**7-98**	IDTT-C8-TIC	–5.64	–3.97	1.59	PBT1-C	1:1.3	0.88	20.3	75	13.7	2.2 × 10^–4^/1.2 × 10^–4^	(174)
**7-99**	IDTT-C10-TIC	–5.71	–3.91	1.61	PBT1-C	1:1.3	0.98	18.1	71	12.7	9.8 × 10^–5^/6.7 × 10^–5^	(174)
**7-100**	*m*-ITTC	–5.62	–3.89		J71	1:1.2	0.87	19.3	74	12.4	-/4.4 × 10^–5^	(175)
**7-101**	*m*-MeIC	–5.54	–3.90	1.54	J71		0.92	18.5	69	11.7	-/2.5 × 10^–4^	(176)

a Obtained from the oxidation/reduction potential of the CV measurement if not otherwise stated.

b HOMO/LUMO energy levels obtained from the LUMO/HOMO levels determined by CV and the optical band gap.

c HOMO obtained via photoelectron spectroscopy in air (PESA).

d Determined via SCLC technique from the neat acceptor/donor:acceptor blend films if not otherwise stated.

**7-66** (IT-4Cl-C8)^159^ and **7-67** (IT-4Cl-10)^159^ have chlorine atoms attached on their INCN units and octylphenyl and decylphenyl side chains, respectively. They differ only slightly in their energy levels and optical band gaps; compared to ITIC, the HOMO/LUMO energy levels are downshifted and the optical band gap is widened. In combination with PM6, solar cells with **7-66** and **7-67** show similar *J*_SC_ and FF values, but PM6/**7-66** gives a lower PCE with 12.9% than PM6/**7-67** (13.5%).^159^ The NFA **7-68** (IDMIC-4F)^160^ has again fluorinated acceptor units, but its side chains are dimethylphenyl groups. The combination of the fluorines and the shorter side chains gave HOMO and LUMO energy levels of −5.46 and −3.83 eV. Blended with PM6, solar cells show a good performance with a PCE of 9.40%. Compared with **7-4**, bearing longer side chains, in combination with PM6, the *V*_OC_ is in the same range, but the PCE is exceeding 13%.^111,160^

Molecule **7-69** (*m*-ITIC-OR-4Cl)^161^ contains INCN-2Cl acceptor units and hexyloxyphenyl side chains, where the alkyloxy chain is present in the meta-position to the backbone. The HOMO/LUMO energy levels as well as the optical band gap are in the same range as for the chlorinated ITIC counterpart **7-7**. Photovoltaic parameters of PM6/**7-69** show a *V*_OC_ of 0.83 V, a *J*_SC_ of 21.6 mA cm^–2^, a FF of 70%, and a PCE of 12.9%, while the PM6/**7-7** combination gives a lower *V*_OC_ but a higher *J*_SC_, FF, and PCE.^161^ Replacing the *p*-hexyl chains in **7-2** and **7-4** with hexyloxy side chains leads to the structures **7-70** (POIT-IC2F) and **7-71** (POIT-IC4F).^149^ Compared to the unfluorinated compound **7-62**, the energy levels are lowered, slightly more for **7-71** than **7-70**, and the optical band gap is 1.49, 1.55, and 1.60 eV for **7-71**, **7-70**, and **7-62**, respectively. Solar cells of the fluorinated acceptors with PM6 have high PCEs over 12%, with a maximum of 13.8%.^149^ Compound **7-72** (IDTT-OB)^162^ has INCN end groups with a methyl substituent. On each side of the backbone, two side chains are present, one being an octyl chain and the other being a hexylphenyl chain. The HOMO/LUMO energy levels and the optical band gap are similar to molecule **7-13**, where similar side chains (hexylphenyl) are attached on each side. In combination with PBDB-T, an efficiency of 11.2% can be reached.^119,162^ In **7-73** (ITPN),^108^ the side chains were extended and branched to butyloctyl chains and the phenylene was changed to thiophenes. Compared to **7-72**, the HOMO/LUMO energy levels are lowered; however, the optical band gap remains at about 1.60 eV. The PV parameters in combination with PM6 are enhanced (except the FF) to a *V*_OC_ of 0.99 V and a PCE of 12.6%. The small molecule acceptor **7-74** (ITIC-Th1)^135,163^ has thiophene rings in the side chains instead of phenyl rings, and compared to **7-2**, the energy levels are downshifted further. Solar cells based on **7-2** blended with PBDS-T reveal a *V*_OC_ of 0.90 V, *J*_SC_ of 18.6 mA cm^–2^, FF of 67%, and PCE of 11.2%. However, **7-74** reached 12.0% efficiency, a better FF of 70%, and *J*_SC_ of 19.6 mA cm^–2^ but a slightly lower *V*_OC_ of 0.88 V using the same donor. The non-fluorinated counterpart of **7-74**, ITIC-Th (**7-50**), achieved 10.9% PCE.^135^ NFA **7-75** (ITThBC)^139^ has the same side chains as ITIC-Th (**7-50**) but contains a sulfone group instead of a carbonyl in the acceptor unit. In comparison with **7-50**, **7-75** has downshifted energy levels and a similar optical band gap. Solar cell parameters with PM6 are also lower with the highest efficiency being 7.59% (P(Cl)/**7-50** = 11.4%). This difference may lie in the selection of the polymer donor and the resulting low electron mobility of 9.4 × 10^–5^ cm^2^ V^–1^ s^–1^ of the PM6/**7-75** blend film.^139,157^

The extension of the π-system on the acceptor units by using naphthyl instead of phenyl rings leads in combination with alternative side chains on the IDTT core to molecules **7-76**–**7-81**.^164−166^ Compared to ITIC, it has the effect that the energy levels are slightly upshifted for **7-76** (ITzN-C9)^165^ and downshifted for **7-78** (ITN-C9).^165^ The fluorinated counterparts of **7-76** and **7-78**, **7-77** (ITzN-F4),^166^ and **7-79** (ITN-F4),^166^ respectively, have much lower HOMO/LUMO energy levels (see Table 3). The [*a*]-annulated naphthalene derivative (**7-76**) has an optical band gap of 1.65 eV, while the [*b*]-annulated naphthalene derivative (**7-78**) has a band gap of 1.54 eV, and thereby, both are wider compared to **7-77** and **7-79**. Solar cells of **7-76** and **7-78** with PM6 reveal a similar PCE of approx. 9.5%, with high *V*_OC_ values of up to 1.05 V for **7-76**. As expected, the lower band gap of **7-78** leads to lower *V*_OC_ but higher *J*_SC_ values with similar FFs in both cases (65%).^165^ Compared to that, the fluorine-containing molecules **7-77** and **7-79** combined with PM6 have similar to lower *V*_OC_s but reach PCEs of over 10.5%. The reason therefore may be the higher *J*_SC_ and FF values, although the electron mobility in the blend film is lower.^166^ Structures **7-80** (IDTT-BH) and **7-81** (IDTT-OBH)^164^ differ from **7-78** only in the side chains on the central IDTT core, i.e., using a 2-butyloctyl-side chain in **7-80** and a 2-butyloctyloxy side chain in **7-81**.^164^ As expected, the optical band gaps (1.54 and 1.57 eV) are very similar to the value of **7-78**. By screening different donor polymers, solar cells based on **7-80** reached with J71 the best photovoltaic performance with PCEs up to 11.1%, whereas **7-81** worked best with PBDB-T as a donor, yielding a PCE of 10.9%.^164,165^

Compounds **7-82**–**7-84** have all octyl side chains and INCN acceptor units with different substitution on the phenyl ring. **7-82** (C8-IT-4F)^167^ is the fluorinated counterpart to **7-49**; in comparison, the LUMO of **7-82** is downshifted, whereas the HOMO energy level is similar to the one of **7-49**. Solar cells with PM7/**7-82** revealed an efficiency of 14.3% with a high FF of 77% and high electron mobilities of 1.1 × 10^–3^ cm^2^ V^–1^ s^–1^ in the as-cast blend film. **7-83** (MF2)^168^ differs from **7-82** in the replacement of one fluorine by one methyl group on each acceptor unit. This leads to a heightened LUMO and an unchanged HOMO energy level with an optical band gap of 1.49 eV. Solar cells with PM7 lead to lower PCEs of 13.7%, due to lower FFs and electron mobilities.^168^ Replacing the fluorines in **7-82** with chlorines leads to molecule **7-84** (C8IDTT-4Cl).^169^ In comparison, the energy levels are further downshifted and **7-84** shows an optical band gap of 1.43 eV. Blended with PBDT-TPD, solar cells show an efficiency of 7.55%.^169^

The exchange of the acceptor unit in **7-49** and **7-82**–**7-84** from INCN to diethyl TBA leads to structure **7-85** (IDTTA).^170^ Compared to **7-49**, **7-85** exhibits lower HOMO/LUMO energy levels and a wider optical band gap. Solar cell devices with the polymer PBDB-T gave a high *V*_OC_ of 0.98 V and a PCE of 10.8%. **7-49**/PBDB-T achieved a lower *V*_OC_ of 0.86 V and a higher PCE of 11.9%, even though the electron mobility is higher for this blend film.^146,170^ The replacement of octyl with hexylphenyl side chains and the introduction of additional alkyl chains on one or two outer thiophene rings in the backbone leads to molecules **7-86** (C_6_-IDTT-T)^171^ and **7-87** (2C_6_-IDTT-T),^171^ respectively. These changes lower the energy levels compared to **7-85** and broaden the optical band gap further, thus leading to higher *V*_OC_ values >1 V in solar cells with PTB7-Th. **7-86** with only one additional hexyl chain achieved a higher PCE of 8.51% compared to **7-87** (7.52%), which may be attributed to the higher electron mobility and thus faster charge transport.^171^ The same backbone with two additional hexyl side chains is present in structures **7-88** (ITC6-2F)^156^ and **7-89** (ITC6-4F).^156^ Like their parent compound, **7-61**, the acceptor units are again INCN based with either one or two fluorines on each acceptor unit in **7-88** and **7-89**, respectively. The fluorines shift the LUMO energy levels slightly downward; the optical band gap is narrowed with increasing fluorine content. Solar cells were built with PBDB-T, and the best PCE value (11.2%) of these three acceptors was achieved by **7-88**.^156^

The structures **7-90** (IM-4F)^172^ and **7-91** (IOM-4F)^172^ have fluorines attached on their acceptor units and additional side chains, but unlike **7-88** and **7-89**, these alkyl and alkyloxy units are located on the central phenyl ring of the backbone. Compared to **7-4**, the additional methyl and methoxy groups lower the LUMO energy levels to −4.19 and −4.27 eV for **7-90** and **7-91**, consequently narrowing the band gap to 1.46 and 1.48 eV, respectively. The photovoltaic parameters of PM6/**7-90-**based devices show a *V*_OC_ of 0.88 V, a FF of 73%, and a high PCE of 14.2%, whereas PM6/**7-91** reached a *V*_OC_ of 0.86 V, a FF of 72%, and a PCE of 13.4%. The reason for the higher efficiency in **7-90** is attributed to the improved *J*_SC_ of 22.1 mA cm^–2^ and the increased electron mobility.^172^

Sung et al. investigated the influence of substitution on the INCN acceptor units in spirobifluorene-containing IDTT-based acceptor structures (**7-51**, **7-92**–**7-94**). Whereas the introduction of the methyl group does not significantly change the HOMO/LUMO energy levels and the optical band gap (1.62 eV for **7-94**, sp-mOEh-ITIC-M,^148^ and 1.61 eV for **7-51**), the halogenation shifted both to lower values (1.54 eV for the fluorinated structure **7-92**, sp-mOEh-ITIC-F,^148^ and 1.52 eV for the chlorinated counterpart **7-93**, sp-mOEh-ITIC-Cl).^148^ Overall, solar cells with these acceptors and PBDB-T as a donor lead to similar efficiencies with PCEs in the range of 5.80–6.40%.

Zhang et al. investigated IDTT-based acceptors with octyl side chains on the central core and CPTCN end groups (**7-95**, C8-ITCC) and compared them to **7-96** (C8-ITCC-Cl) with chlorinated CPTCN (CPTCN-Cl).^173^ In both cases, a mixture of structural isomers are obtained due to the different annulation of the thiophene ring. The chlorinated acceptor has lower energy levels and a smaller optical band gap than the non-halogenated counterpart. The photovoltaic parameters are also improved, giving a PCE of 12.7% and a slightly lower *V*_OC_ of 0.95 V. In contrast, **7-95** reached a PCE of 10.8% and a *V*_OC_ of 1.04 V.^173^ Similar structures with CPTCN acceptor units are designed by Ye et al., which differ in the length of their side chains on the central core being 6 (**7-97**, IDTT-C6-TIC),^174^ 8 (**7-98**, IDTT-C8-TIC),^174^ or 10 (**7-99**, IDTT-C10-TIC)^174^ carbon atoms long. Upon the extension of the alkyl chain, the HOMO levels are lowered, whereas the LUMO levels are heightened. In solar cells with PBT1-C, **7-97** showed the lowest efficiency with 10.0%, followed by **7-99** with 12.7% and **7-98** with 13.7%. It shows that in this case the octyl-chain-containing structure yielded the best results, due to a more refined morphology in the blend film and therefore higher charge transport.^174^ Replacing the alkyl chain in **7-97**–**7-99** with hexylphenyl chains with the hexyl residue in the meta position leads to molecules **7-100** (*m*-ITTC)^175^ with HOMO/LUMO energy levels of −5.62 and −3.89 eV, respectively. The introduction of an additional methyl group on the CPTCN (CPTCN-Me) leads to **7-101** (*m*-MeIC)^176^ with downshifted HOMO/LUMO energies. Both acceptors were blended with J71 and implemented in solar cells, which yielded 12.4% for **7-100** and 11.7% for **7-101**.^175,176^

**Figure 8:**
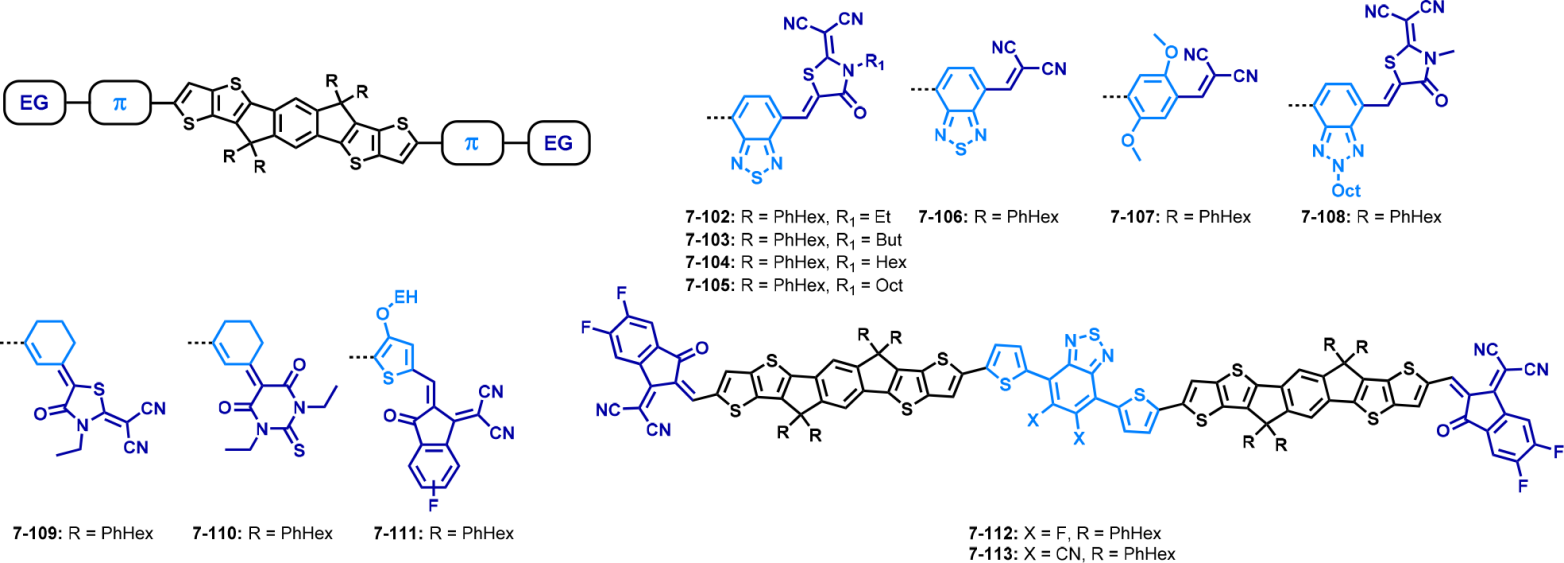
Structures of non-fullerene acceptors with IDTT core units and π-spacer/dimeric compounds.

The IDTT building block was used for the preparation of a variety of other NFAs, summarized in Figure 8 and Table 4. For example, IDTT was coupled with the electron-deficient benzothiadiazole (BT) π-bridge and *N*-alkylated RCN end groups with different alkyl chains leading to the NFAs **7-102**–**7-105** (ITBTR-C2–ITBTR-C8).^177^ All four acceptors have similar HOMO/LUMO energy levels of about −5.30 and −3.70 eV, respectively, which are upshifted in comparison to those of ITIC. The optical band gaps of all four NFAs show similar values around 1.52 eV, as the alkyl chains do not influence the electronic structure here. Solar cells with PBDB-T reveal similar values of all photovoltaic parameters. The PCEs varied slightly between the NFAs, giving 7.04, 7.43, 8.26, and 7.93% for **7-102**, **7-103**, **7-104**, and **7-105**, respectively. According to GIWAXS data of the neat acceptor films, **7-102** tends to crystallize more than the other three, whereas **7-104**, with the highest efficiency, shows more ordered packing compared to **7-103** and **7-105**.^177^

**Table 4:** Optical, Electrical, and Photovoltaic Properties of Non-Fullerene Acceptors **7-102**–**7-113**

NFA	original name	HOMO^a^ (eV)	LUMO^a^ (eV)	*E*_g_^opt^ (eV)	donor	D:A ratio	*V*_OC_ (V)	*J*_SC_(mA cm^–2^)	FF (%)	PCE (%)	μ_e_^c^(cm^2^ V^–1^ s^–1^)	ref.
**7-102**	ITBTR-C2	–5.30	–3.70	1.52	PBDB-T	1:1	0.89	13.5	59	7.04	-/8.6 × 10^–6^	(177)
**7-103**	ITBTR-C4	–5.30	–3.71	1.52	PBDB-T	1:1	0.90	14.9	55	7.43	-/2.4 × 10^–5^	(177)
**7-104**	ITBTR-C6	–5.30	–3.71	1.52	PBDB-T	1:1	0.89	14.9	58	8.26	-/6.6 × 10^–5^	(177)
**7-105**	ITBTR-C8	–5.28	–3.70	1.49	PBDB-T	1:1	0.90	14.9	59	7.93	-/3.4 × 10^–5^	(177)
**7-106**	IDTTBM		–4.03^b^		PTB7-Th	1:1	0.83	15.5	63	8.40		(178)
**7-107**	IDTT-BO-MN	–5.98	–3.81	2.17	P3HT	1:2	0.93	10.2	61	5.75	-/9.1 × 10^–4^	(179)
**7-108**	BTA13	–5.34	–3.62^b^	1.72	J52-F	1:1	1.18	11.6	61	8.36	-/6.2 × 10^–5^	(180)
**7-109**	IDTT-CR	–5.25	–3.38	1.74	P3HT	1:1	0.82	5.61	55	2.52		(140)
**7-110**	IDTT-CT	–5.28	–3.65	1.63	PTB7-Th	1:1	0.93	12.6	51	5.89		(140)
**7-111**	ITOTIC-2F	–5.22	–4.11	1.32	PTB7-Th	1:1.5	0.76	7.00	61	3.70	-/5.1 × 10^–6^	(181)
**7-112**	FDTBT-IDTT-FINCN	–5.46	–3.92	1.51	PBDB-T	1:1	0.86	13.6	62	7.27	-/3.9 × 10^–5^	(182)
**7-113**	CNDTBT-IDTT-FINCN	–5.56	–3.98	1.45	PBDB-T	1:1	0.82	17.1	65	9.13	-/1.2 × 10^–4^	(182)

a Obtained from the oxidation/reduction potential of the CV measurement if not otherwise stated.

b HOMO/LUMO energy levels obtained from the LUMO/HOMO levels determined by CV and the optical band gap.

c Determined via the SCLC technique from the neat acceptor/donor:acceptor blend films if not otherwise stated.

A similar design was used in NFA **7-106** (IDTTBM),^178^ by replacing the RCN groups with methylidene malonitrile groups. Replacing the BT group by a *p*-dimethoxyphenylene group leads to the structure **7-107** (IDTT-BO-MN).^179^ In contrast to ITIC, the HOMO energy level of **7-107** is downshifted, giving a larger band gap of 2.17 eV. Following this, the absorption of **7-107** is blue-shifted by about 200 nm in film and in solution. Solar cells with a combination of **7-106**/PTB7-Th reveal 8.40% efficiency, whereas solar cells of **7-107**/P3HT reach 5.75%, mainly due to the lower *J*_SC_ caused by the limited absorption range of both the NFA **7-107** and P3HT.^178,179^ Exchanging the BT group in **7-102** with a *N*-octyl-benzotriazole unit leads to the NFA **7-108** (BTA13).^180^ This change slightly shifts the HOMO downward, whereas the LUMO energy level is shifted upward, thereby broadening the optical band gap to 1.72 eV. Blended with J52-F, it achieved a remarkable *V*_OC_ value of 1.18 V. However, due to the limited *J*_SC_ of 11.6 mA cm^–2^, an efficiency of only 8.36% was reached.^180^**7-109** (IDTT-CR)^140^ comprises, like **7-102**, RCN-based end groups but a different π-bridge (cyclohexene). The cyclohexene shifts the LUMO level upward, thus broadening the band gap compared to **7-102**. However, in combination with P3HT, **7-109** shows a poor efficiency of 2.52% resulting from its low FF and *J*_SC_ values.^140^ The replacement of the RCN-based end groups in **7-109** with a diethyl TBA group leads to structure **7-110** (IDTT-CT).^140^ The TBA unit lowers again the LUMO level and thereby narrows the optical band gap to 1.63 eV. Here, PTB7-Th was used as donor, giving a *V*_OC_ of 0.93 V, a *J*_SC_ of 12.6 mA cm^–2^, a FF of 51%, and a PCE of 5.89%.^140^

**7-111** (ITOTIC-2F)^181^ features thiophenes with alkyloxy chains as a π-bridge and INCN-F end groups. Compared to **7-2**, which has the same end-capped groups, the HOMO is upshifted and the LUMO level downshifted. In solar cells combined with PBDB-T, **7-111** gave a lower PCE of 3.70% with a *V*_OC_ of 0.76 V, a *J*_SC_ of 7.00 mA cm^–2^, and a FF of 61% (**7-2** had a PCE of 9.3%). A more than 3 times higher efficiency (12.1% PCE) was reached with compound **5-149**, which consists of a five-ring central core and the same π-bridge as **7-111**. The poorer performance of the compound **7-111** despite its larger conjugation length was explained by an unfavorable active layer phase morphology.^181^

The structures **7-112** (FDTBT-IDTT-FINCN) and **7-113** (CNDTBT-IDTT-FINCN)^182^ are combining two IDTT units with INCN-2F end groups via a thieno-benzothiadiazole-thienyl bridge, thereby resulting in a more oligomer-like NFA species. The difference between these two acceptors are the substituents on the BT, which are two fluorine atoms in the molecule **7-112** and two nitrile groups in case of **7-113**. The HOMO/LUMO energy levels of **7-112** and **7-113** are −5.46 and −3.92 eV as well as −5.56 and −3.98 eV, respectively. The optical band gaps are lower compared to ITIC with 1.51 eV for the fluorinated and 1.45 eV for the nitrile-containing counterpart. GIWAXS data showed that **7-113** has a reduced crystallinity in pristine film. The photovoltaic parameters are similar in terms of *V*_OC_ and FF, but **7-113** has a better *J*_SC_ of 17.1 mA cm^–2^ and PCE of 9.13%. On the contrary, **7-111** achieved 7.27% in photovoltaic devices.^182^

### 3.4 Impact of the Central Donor Unit

**Figure 9:**
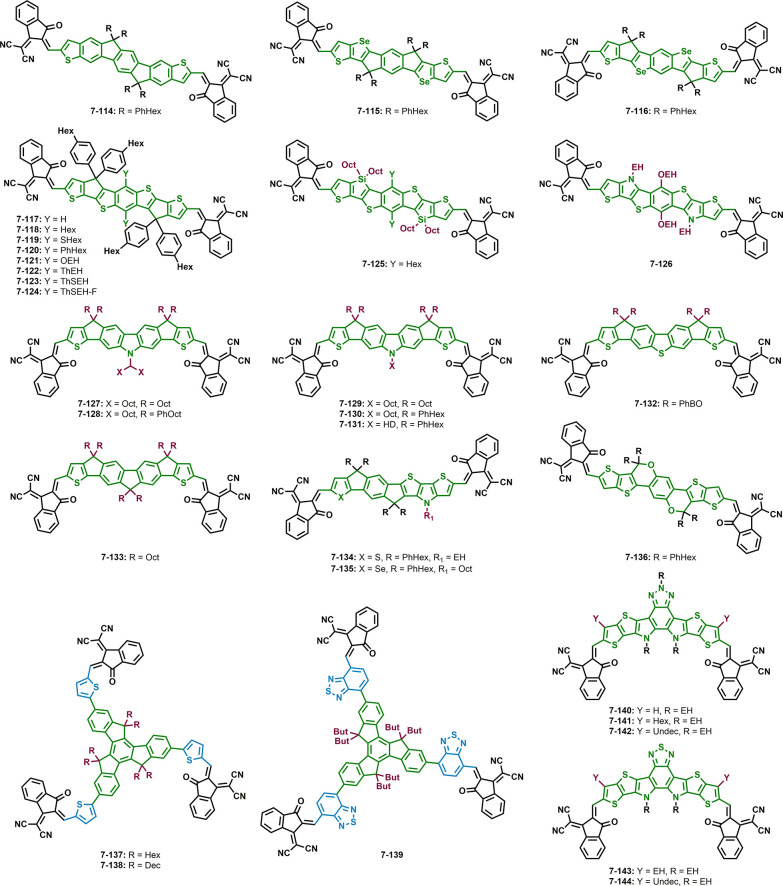
Structures of non-fullerene acceptors with the same end group and different donor units.

The central donor core is of utmost importance for the optical and electronical properties of the NFA. NFAs with INCN as acceptor subunits are the most common structures and thus are very suitable for the comparison of the influence of the central donor subunit (see Figure 9 and Table 5). By replacing the thienothiophene units with benzothiophene subunits in ITIC, the acceptor **7-114** (NIDBT)^183^ is obtained and exhibits a broad optical band gap of 1.84 eV due to the lower HOMO energy level of −5.87 eV compared to ITIC. Solar cells of **7-114** combined with PTB7-Th showed only moderate PCE values of max. 4.45%.^183^ The modification of ITIC by the introduction of selenophenothiophenes instead of the thienothiophene units leads to structure **7-115** (SeTIC).^184^ The selenium in the structure causes a slight reduction of the band gap compared to ITIC. Solar cells of **7-115** with PM6 as a donor gave similar *J*_SC_ values than those with ITIC, but due to slightly lower *V*_OC_ and significant lower FF values, the PCEs only achieved a maximum of 7.46%.^184^ In structure **7-116** (BDSeIC),^185^ the selenophene rings were swapped with the adjacent cyclopentadienyl rings bearing the hexylphenyl side chains. This leads to an even smaller band gap of 1.51 eV due to a slight downshift of the LUMO and an upshift of the HOMO energy level. Solar cells with PM6 gave similar results with maximum PCEs of about 7.10%.^185^ By replacing Se by S in this structure, acceptor **7-117** (BDT-IC)^186,187^ is obtained. NFA **7-117** is similar to ITIC but with exchanged thiophene and (dihexylphenyl)cyclopentadienyl units. The HOMO/LUMO energy levels as well as the optical band gap are comparable to ITIC. Organic solar cells were built with J71 as a polymer donor and revealed a *V*_OC_ of 0.92 V, *J*_SC_ of 17.3 mA cm^–2^, FF of 66%, and PCE of 10.5%. In contrast, solar cells of ITIC/J71 are only showing a PCE of 8.99%.^186,188^ The molecules **7-118**–**7-124** have the same conjugated donor core but have various side chains introduced at the central phenyl ring. Upon the introduction of hexyl side chains (**7-118**, CBT-IC),^186^ the LUMO level is downshifted, leading to a smaller band gap of 1.53 eV. However, hexylthio side chains (**7-119**, SBT-IC)^186^ lower the energy levels, and the band gap increased to 1.57 eV. GIWAXS data of the neat films indicate that the addition of hexyl or hexylthio side chains weakens the π–π stacking in the out-of-plane direction, which may lead to weaker crystallinity of the acceptor. Solar cells of **7-118** and **7-119** show PCEs up to 11.3% with similar *V*_OC_, *J*_SC_, and FF values as those of **7-117**. The electron mobility is rather low for all three acceptors.^186^**7-120** (BPIC)^189^ comprises hexylphenyl chains. Compared to the simple hexyl analogue **7-118**, the LUMO level of **7-120** is heightened, but the HOMO energy level and the optical band gap are unchanged. Solar cells were built with PBDB-T and yielded efficiencies up to 10.7%, which is lower than for **7-118**/J71 devices.^186,189^**7-121** (BT-IC)^190^ features an ethylhexyloxy side chain (see Figure 9). The +M-effect heightens both energy levels, but especially the HOMO, leading to a reduced band gap of 1.43 eV. Blended with PBDB-T, the solar cells show PCE values like the solar cells based on **7-117**.

**Table 5:** Optical, Electrical, and Photovoltaic Properties of Non-Fullerene Acceptors **7-114**–**7-144**

NFA	original name	HOMO^a^ (eV)	LUMO^a^ (eV)	*E*_g_^opt^ (eV)	donor	D/A ratio	*V*_OC_ (V)	*J*_SC_(mA cm^–2^)	FF (%)	PCE (%)	μ_e_^b^(cm^2^ V^–1^ s^–1^)	ref.
**7-114**	NIDBT	–5.87	–3.91	1.84	PTB7-Th	1:1	0.83	11.5	47	4.45	3.5 × 10^–5^/6.9 × 10^–5^	(183)
**7-115**	SeTIC	–5.55	–3.90	1.58	PM6	1:1	0.95	15.5	51	7.46	-/2.9 × 10^–3^	(184)
**7-116**	BDSeIC	–5.53	–3.92	1.51	PM6	1:1	0.97	14.0	52	7.10	1.6 × 10^–4^/1.2 × 10^–4^	(185)
**7-117**	BDT-IC	–5.51	–3.90	1.61	J71		0.92	17.3	66	10.5	-/1.9 × 10^–4^	(186)
**7-118**	CBT-IC	–5.50	–3.97	1.53	J71	0.9:1	0.92	18.1	66	11.0	-/8.6 × 10^–5^	(186)
**7-119**	SBT-IC	–5.61	–4.04	1.57	J71	0.8:1.1	0.89	17.8	71	11.3	-/1.1 × 10^–4^	(186)
**7-120**	BPIC	–5.51	–3.81	1.53	PBDB-T	1:1	0.88	17.9	68	10.7	6.3 × 10^–4^/3.8 × 10^–4^	(189)
**7-121**	BT-IC	–5.39	–3.87	1.43	PBDB-T	1:1	0.85	18.1	70	10.8		(190)
**7-122**	ITIC2	–5.48	–3.84	1.53	PBDB-T-SF	1:1	1.09	15.7	60	10.1	6.4 × 10^–4^/4.2 × 10^–4^	(191)
**7-123**	ITIC-S	–5.50	–3.86	1.55	PBDB-T-SF	1:1	1.06	16.4	67	11.6	7.0 × 10^–4^/4.4 × 10^–4^	(191)
**7-124**	ITIC-SF	–5.57	–3.92	1.58	PBDB-T-SF	1:1	1.04	16.8	69	12.1	5.3 × 10^–4^/4.0 × 10^–4^	(192)
**7-125**	ArSiID	–5.37	–3.86		J51	1:1	0.84	16.2	60	8.30	-/2.4 × 10^–4^	(193)
**7-126**	BDTIC	–5.30	–3.85	1.38	PBDB-T	1:1	0.88	20.0	68	12.1	-/2.3 × 10^–4^	(194)
**7-127**	DTC(4R)-IC	–5.69	–3.63		J71	1:1.3	0.94	16.4	62	9.61		(195)
**7-128**	DTC(4Ph)-IC	–5.75	–3.67		PBDB-T		0.97	14.3	68	9.48		(196)
**7-129**	DTCCIC	–5.72	–3.58	1.62	PFBDB-T	1:2	1.01	12.6	48	6.20	-/2.2 × 10^–6^	(197)
**7-130**	HCN-C8	–5.63	–3.54	1.67	J71	1:1	1.01	6.24	38	2.38	-/9.4 × 10^–7^	(198)
**7-131**	HCN-C16	–5.63	–3.57	1.66	J71	1:1	1.03	12.3	44	5.51	-/1.3 × 10^–6^	(198)
**7-132**	DBTIC	–5.91	–4.00	1.67	PBDB-T	1:1	1.00	15.5	62	9.66	-/2.0 × 10^–6^	(199)
**7-133**	F-H	–5.42^c^	–3.79	1.63	PBDB-T		0.94	15.0	67	9.59		(200)
**7-134**	TPIC	–5.30	–3.85	1.50	PM7	1:1	1.00	18.8	71	13.3	6.7 × 10^–4^/3.7 × 10^–4^	(201)
**7-135**	DTPPSe-IC	–5.38	–3.84	1.46	PBDB-T	1:1.25	0.90	17.3	63	9.88	-/4.5 × 10^–4^	(202)
**7-136**	CO_j_7IC	–5.66	–3.92	1.60	PTB7-Th	1:1.4	0.82	13.1	59	6.36	-/1.4 × 10^–5^	(203)
**7-137**	Tr(Hex)_6_-3IC	–5.92	–3.94	1.90	PTB7-Th	1:1	0.88	3.71	38	1.28	-/5.4 × 10^–6^	(204)
**7-138**	Tr(Dec)_6_-3IC	–5.88	–3.90	1.92	PTB7-Th	1:1	0.74	2.55	28	0.55	-/2.0 × 10^–6^	(204)
**7-139**	TrBTIC	–5.56	–3.62	1.80	P3HT	1:1.2	0.88	13.0	72	8.25	-/3.3 × 10^–4^	(205)
**7-140**	Y1	–5.45	–3.95	1.44	PBDB-T	1:1	0.87	22.4	69	13.4	-/3.0 × 10^–4^	(206)
**7-141**	Y16	–5.25	–3.66		PBDB-T	1:1	0.91	21.3	67	13.0	-/5.0 × 10^–4^	(207)
**7-142**	Y9	–5.59	–3.78	1.36	PBDB-T	1:1	0.90	23.3	63	13.3	6.7 × 10^–4^/4.1 × 10^–4^	(208)
**7-143**	Y5	–5.55	–3.87	1.38	PBDB-T	1:1.5	0.88	22.8	70	14.1	2.1 × 10^–4^/4.0 × 10^–4^	(209)
**7-144**	BTP-IC	–5.58	–3.90	1.42	PM6	1:1.2	0.96	14.7	53	7.54	9.0 × 10^–5^/1.4 × 10^–4^	(210)
	BTP	–5.52	–3.82	1.40	PM6	1:1.2	0.96	16.3	57	8.85	-/9.4 × 10^–4^	(211)

a Obtained from the oxidation/reduction potential of the CV measurement if not otherwise stated.

b Determined via the SCLC technique from the neat acceptor/donor:acceptor blend films if not otherwise stated.

c Obtained via ultraviolet photoelectron spectroscopy (UPS).

**7-122** (ITIC2)^191^ and **7-123** (ITIC-S)^191^ contain 2-ethylhexylthiophene and 2-ethylhexylthiothiophene side chains, respectively. Compared to **7-117**, this does not have much impact on the band gap and energy levels. Regarding the PV parameters, the *V*_OC_ is reaching over 1 V, and for **7-122**, the FF and PCE are lower; for **7-123**, they are higher than for **7-117**. GIWAXS data indicate that the thioether bond reduces the crystallinity of the **7-123** neat film. The effect is also present in the blended films, but it is weakened.^186,191^ When a fluorine atom is added to the thiophene ring in the side chain of **7-123**, it leads to structure **7-124** (ITIC-SF).^192^ The fluorine lowers the energy levels and widens the optical band gap to 1.58 eV. Solar cell devices with PBDB-T-SF as a donor reached a higher efficiency of 12.1% than **7-123** with a *V*_OC_ of over 1 V.^191,192^

A variation of the core structure of **7-118**–**7-124** was introduced by Wang et al., who exchanged the cyclopentadiene rings with dioctylsiloles to give the structure **7-125** (ArSiID),^193^ shifting the HOMO/LUMO energy levels slightly to higher values. Solar cells using J51 as a donor polymer show a PCE of 8.30%, a *V*_OC_ of 0.84 V, a *J*_SC_ of 16.2 mA cm^–2^, and a FF of nearly 60%.^193^ Chen et al. exchanged the silole ring with 2-ethylhexyl pyrrole in the NFA **7-126** (BDTIC).^194^ This change upshifts the HOMO level, leading to a band gap of 1.38 eV. Combined with PBDB-T, solar cells with **7-126** reached PCE values up to 12.1%.^194^

Molecules **7-127**–**7-131** contain a pyrrole unit in the center of the donor unit but differ in the side chains. **7-127** (DTC(4R)-IC) comprise *N*-octylnonyl groups and octyl side chains,^195^**7-128** (DTC(4Ph)-IC) *N*-octylnonyl and octylphenyl,^196^**7-129** (DTCCIC) *N*-octyl and octyl,^197^**7-130** (HCN-C8) *N*-octyl and hexylphenyl,^198^ and **7-131** (HCN-C16) *N*-hexadecyl and hexylphenyl groups.^198^ They have the most upshifted LUMO and downshifted HOMO energy levels compared to ITIC. Solar cells of **7-127** and **7-128** show PCEs of over 9% blended with J71 or PBDB-T, respectively,^195,196^ and with **7-129**—bearing the shortest side chains—they show a *V*_OC_ of 1.01 V but only an efficiency of 6.20%.^197^*V*_OC_ values of over 1 V are also observed with **7-130**/J71 and **7-131**/J71 blends, but the PCE values decrease even further to 5.51 to 2.38%.^198^ Structure **7-132** (DBTIC)^199^ comprises a thiophene as a central unit. This leads to a further downshift of the HOMO level to −5.91 eV. Solar cells using PBDB-T as a donor polymer show comparable parameters to those of **7-128**.^196,199^ Replacing this central thiophene unit in **7-132** with a cyclopentadienyl ring leads to structure **7-133** (F-H).^200^ The frontier orbital energy levels are upshifted, hereby the HOMO more than the LUMO, narrowing the band gap slightly. The PV devices of PBDB-T/**7-133** gave a PCE of 9.59%.^200^

An asymmetric backbone can enhance the π–π stacking, thereby improving charge transport.^212^ The asymmetric **7-134** (TPIC)^201^ and **7-135** (DTPPSe-IC)^202^ feature the same backbone with the difference that **7-135** comprises a selenophene instead of one thiophene ring, leading to a slightly decreased optical band gap. Solar cells of **7-134**/PM7 yield a good efficiency of 13.3% and a *V*_OC_ of 1.00 V.^201^ Devices of **7-135**/PBDB-T reached only PCE values up to 9.88%.^202^ Molecule **7-136** (CO_j_7IC)^203^ contains carbon–oxygen bridges in the core unit. It possesses HOMO and LUMO energy levels of −5.66 and −3.92 eV and an optical band gap of 1.60 eV, which are all in the same range as ITIC. Solar cells were built with PTB7-Th as a donor and achieved a PCE of 6.36%.^203^

In a quite different seven-ring structure, truxene was used as the central core for the acceptors **7-137** (Tr(Hex)_6_-3IC),^204^**7-138** (Tr(Dec)_6_-3IC),^204^ and **7-139** (TrBTIC)^205^ with the difference between **7-137** and **7-138** being only in the length of the side chains. **7-139** differs from the others regarding the side chains and π-bridge. In general, the energy levels of **7-137** and **7-138** are much lower than the ones of ITIC and the band gap is enlarged to values equal or over 1.9 eV. **7-139** shows a higher LUMO level than ITIC, thus enlarging the band gap to 1.80 eV. The solar cells of **7-137** and **7-138** were built with PTB7-Th as a donor material and the devices showed moderate PV parameters, having efficiencies of 1.28% for **7-137** and 0.55% for **7-138**, respectively. Also, the electron mobilities of the active layer films are rather low. However, molecule **7-139** in combination with P3HT revealed a higher PV performance with a PCE of 8.25%.^204,205^

### 3.5 The Y Series and Related NFAs

**Figure 10:**
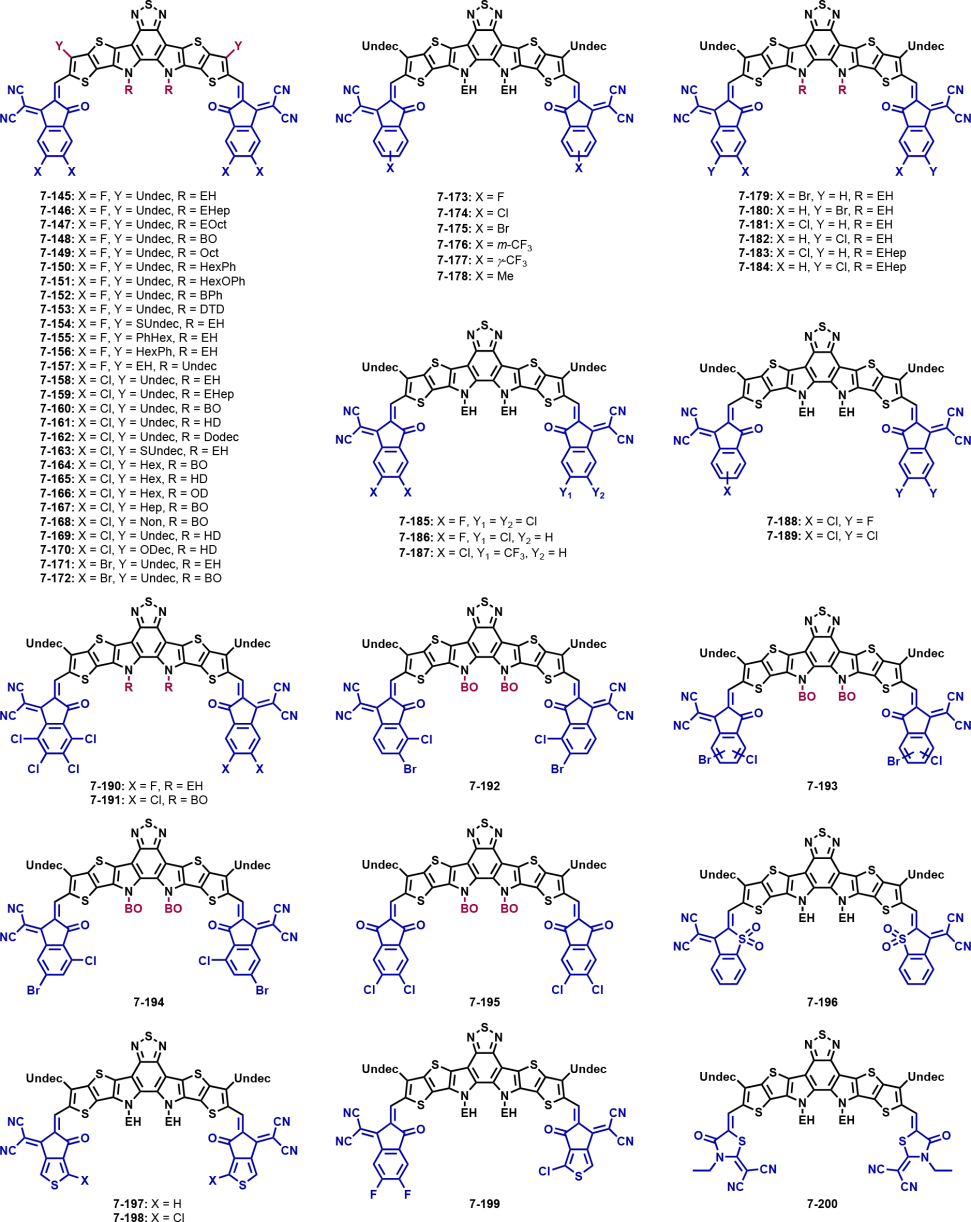
Structures of non-fullerene acceptors with Y backbones containing benzothiadiazole units.

**Figure 11:**
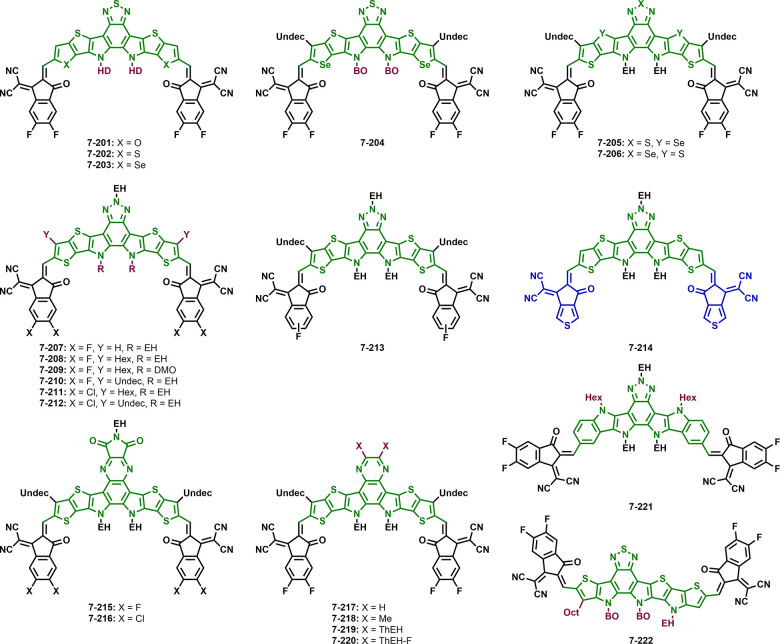
Structures of non-fullerene acceptors with Y backbones containing Se atoms or benzotriazole core units.

A boost in efficiency was experienced by introducing the so-called Y-series by altering the classical A–D–A structural motif. By attaching electron-accepting units in the central core, the donor subunit is split in a DAD structure, resulting in an A–(DA′D)–A motif (see Figures 9–11 and Tables 5–7).^213^ This can be achieved by, e.g., introducing a benzotriazole group as the central core (**7-140**, Y1,^206^**7-141**, Y16,^207^ and **7-142**, Y9,^208^ differing in the side chains on the central core) or a benzothiadiazole unit (**7-143**, Y5,^209^ and **7-144**, BTP,^211^ differing also in the side chains on the central core) flanked by thienothienopyrrole units on both sides. Compared to ITIC, the energy values of the HOMO/LUMO are only slightly different (except **7-141**); however, the optical band gap decreased for all four molecules, red-shifting the absorption by 40–90 nm in solution as well as in thin films (see also Table 5). Combined with PBDB-T, the solar cells of **7-140**–**7-143** achieved efficiencies over 13% (for **7-142**, even more than 14% due to the increased *J*_SC_ values above 22 mA cm^–2^), good FFs between 63 and 70%, and simultaneous high *V*_OC_ values between 0.87 and 0.91 V.^206−209^ Only **7-144**/PBDB-T gave, compared to the others, lower PCEs of 8.80%, even though the electron mobility in the blend is quite high with a value of 9.4 × 10^–4^ cm^2^ V^–1^ s^–1^.^211^ These last three structures have been shown to belong to the most efficient acceptor materials currently known and are in the center of today’s OPV research.^214^ Based on the above described A–(DA′D)–A motifs, this new class of materials can be divided into three main groups according to their central A′ unit, i.e., either (i) a benzothiadiazole unit, **7-145**–**7-205** and **7-222** (see Figures 10 and 11), (ii) a benzotriazole core, **7-207**–**7-214** and **7-221**, or (iii) a quinoxaline unit (1,4 benzopyrazine) **7-215**–**7-220**.

**Table 6:** Optical, Electrical, and Photovoltaic Properties of Non-Fullerene Acceptors **7-145**–**7-200**

NFA	original name	HOMO^a^ (eV)	LUMO^a^ (eV)	*E*_g_^opt^ (eV)	donor	D:A ratio	*V*_OC_ (V)	*J*_SC_(mA cm^–2^)	FF (%)	PCE (%)	μ_e_^d^(cm^2^ V^–1^ s^–1^)	ref.
**7-145**	Y6	–5.66	–4.08	1.33	PM6	1:1.2	0.82	25.2	76	15.7		(247)
	BTP-4F	–5.65	–4.02		PM6	1:1.2	0.83	24.9	75	15.6	-/7.3 × 10^–5^	(225)
	Y6	–5.72	–4.00		PM6	1:1.2	0.85	25.5	75	16.2	5.7 × 10^–4^/3.4 × 10^–4^	(222)
	Y6	–5.65	–4.10		D18	1:1.6	0.86	27.7	77	18.2	-/1.4 × 10^–4^	(1)
**7-146**	Y6-C2	–5.65	–4.09		PM6-Ir1.5	1:1.2	0.84	26.1	78	17.1	-/4.3 × 10^–4^	(215)
**7-147**	Y6-C3	–5.68	–4.07	1.41	PM6	1:1.2	0.83	24.1	67	13.8	2.5 × 10^–4^/5.1 × 10^–5^	(216)
**7-148**	BTP-4F-12	–5.68	–4.06		PM6	1:1.2	0.86	25.3	76	16.4	7.4 × 10^–4^/-e	(217)
**7-149**	Y6-nC8	–5.71	–4.08	1.41	PM6	1:1.5	0.86	19.1	63	10.4	2.3 × 10^–4^/2.4 × 10^–4^	(218)
**7-150**	C6	–5.58	–4.06	1.37	PM6	1:1.2	0.84	23.8	73	14.5		(219)
**7-151**	Y6-phOC6	–5.75	–4.05	1.43	PM6	1:1.5	0.84	21.3	62	11.1	7.1 × 10^–4^/4.7 × 10^–5^	(218)
**7-152**	C4	–5.60	–4.05	1.39	PM6	1:1.2	0.71	16.4	68	7.92		(219)
**7-153**	DTY6	–5.67	–4.04	1.40	PM6	1:1.2	0.86	25.3	75	16.3	4.7 × 10^–4^/6.8 × 10^–4^	(220)
**7-154**	BTPS-4F	–5.73	–3.91	1.38	PM6	1:1.2	0.82	24.8	76	16.2	-/2.1 × 10^–4^	(221)
**7-155**	BTP-PhC6	–5.58	–3.85	1.36	PM6	1:1.2	0.87	25.0	77	16.7	-/7.2 × 10^–4^	(223)
**7-156**	BTP-C6Ph	–5.60	–3.94	1.35	PM6	1:1.2	0.84	24.3	76	15.5	-/6.1 × 10^–4^	(223)
**7-157**	N-C11				PM6	1:1.2	0.85	21.5	71	12.9	-/1.1 × 10^–4^	(224)
**7-158**	BTP-4Cl	–5.68	–4.12^c^		PM6	1:1	0.87	25.4	75	16.5	-/1.6 × 10^–4^	(225)
**7-159**	N3-4Cl	–5.63	–3.98	1.35	PM6	1:1.2	0.85	25.9	75	16.5	-/4.5 × 10^–4^	(226)
**7-160**	BTP-4Cl-12	–5.66	–4.09	1.39	PM6	1:1.2	0.86	25.6	78	17.0		(227)
	BTP-BO-4Cl				PM6	1:1.2	0.85	26.1	78	17.3	-/3.9 × 10^–4^	(229)
**7-161**	BTP-4Cl-16	–5.68	–4.09	1.40	PM6	1:1.2	0.86	24.2	75	15.6		(227)
**7-162**	BTIC-C12-4Cl	–5.56	–4.16	1.39	PM6		0.84	19.8	68	11.4	-/8.7 × 10^–5^	(228)
**7-163**	BTPS-4Cl	–5.65	–3.93	1.36	PM6	1:1.2	0.80	24.0	68	13.5	-/1.9 × 10^–4^	(221)
**7-164**	BT_6_IC-BO-4Cl	–5.54	–4.05	1.35	PM6	1:1.2	0.84	23.6	73	14.4	-/4.8 × 10^–5^	(230)
**7-165**	BT_6_IC-HD-4Cl	–5.57	–4.10	1.35	PM6	1:1.2	0.88	23.4	73	14.9	-/6.4 × 10^–5^	(230)
**7-166**	BT_6_IC-OD-4Cl	–5.55	–4.11	1.35	PM6	1:1.2	0.89	18.6	58	9.60	-/5.6 × 10^–6^	(230)
**7-167**	BTP-eC7	–5.62	–4.03	1.40	PM6	1:1.2	0.84	24.1	74	14.9	1.3 × 10^–4^/-	(231)
**7-168**	BTP-eC9	–5.64	–4.05	1.40	PM6	1:1.2	0.84	26.2	81	17.8	2.7 × 10^–4^/-	(231)
**7-169**	HD-4Cl	–5.68	–4.09		PM6	1:1.2	0.85	25.4	72	15.7	1.2 × 10^–4^/-	(232)
**7-170**	HDO-4Cl	–5.70	–3.91		PM6	1:1.6	0.94	21.9	76	15.6	1.4 × 10^–4^/-	(232)
**7-171**	BTIC-4Br	–5.57	–4.11		PM6	1:1.2	0.85	20.7	70	12.2	2.3 × 10^–5^/1.1 × 10^–5^	(233)
**7-172**	BTIC-BO-4Br	–5.53	–4.09		PM6	1:1.2	0.86	24.1	68	14.0	8.1 × 10^–5^/4.5 × 10^–5^	(233)
**7-173**	Y8	–5.55	–3.89	1.35	PM6	1:1.2	0.88	22.2	73	14.3	-/8.7 × 10^–4^	(234)
**7-174**	BTIC-Cl-*m*	–5.42	–3.91	1.34	PM6	1:1.2	0.88	21.4	70	13.2	-/3.8 × 10^–4^	(235)
	ZC	–5.69	–4.12	1.38	PM6	1:1	0.90	25.1	65	14.7	-/1.5 × 10^–4^	(248)
**7-175**	BTIC-2Br-*m*	–5.56	–4.07		PM6	1:1.2	0.88	25.0	73	16.1	2.9 × 10^–4^/1.1 × 10^–4^	(233)
	TPT10	–5.52	–3.99	1.36	PTQ11	1:1.2	0.88	24.8	75	16.3		(249)
**7-176**	BTIC-CF_3_-*m*	–5.45	–3.97	1.31	PM6	1:1.2	0.85	24.9	72	15.3	-/6.6 × 10^–5^	(235)
**7-177**	BTIC-CF_3_-γ	–5.45	–3.96	1.30	PM6	1:1.2	0.85	25.2	73	15.6	-/4.5 × 10^–4^	(235)
**7-178**	BTP-M	–5.48	–3.81	1.42	PM6	1:1.2	0.98	8.43	52	4.26	-/5.2 × 10^–4^	(236)
**7-179**	BTPIC-2Br-5	–5.63	–3.91	1.37	PM6	1:1.2	0.90	22.9	68	14.0	-/2.3 × 10^–4^	(237)
**7-180**	BTPIC-2Br-6	–5.60	–3.93	1.34	PM6	1:1.2	0.87	24.1	71	15.0	-/2.7 × 10^–4^	(237)
**7-181**	LY-Cl-1	–5.66	–3.82	1.39	PM6	1:1.1	0.91	24.3	65	14.4	4.6 × 10^–4^/7.3 × 10^–4^	(238)
**7-182**	LY-Cl-2	–5.61	–3.84	1.36	PM6	1:1.1	0.88	24.2	71	15.2	7.1 × 10^–4^/1.0 × 10^–3^	(238)
	BTP-2Cl-δ	–5.57	–3.85	1.36	PM6	1:1.2	0.89	24.3	71	15.4	-/5.8 × 10^–4^	(211)
**7-183**	N3-Cl-1	–5.64	–3.78	1.37	PL1	1:1.5	0.88	23.9	71	15.1	5.8 × 10^–4^/6.2 × 10^–4^	(238)
**7-184**	N3-Cl-2	–5.58	–3.80	1.33	PL1	1:1.5	0.85	25.6	76	16.4	1.5 × 10^–3^/9.2 × 10^–4^	(238)
**7-185**	SY2	–5.67	–3.99		PM6	1:1.2	0.85	25.3	74	16.0	6.1 × 10^–4^/3.6 × 10^–4^	(222)
**7-186**	BTIC-γCl-2F	–5.39	–3.87		PM6	1:1.2	0.86	24.6	73	15.4	-/1.8 × 10^–4^	(116)
**7-187**	BTIC-2Cl-γCF_3_	–5.55	–4.00	1.31	PM6	1:1.2	0.84	25.1	77	16.3	-/1.5 × 10^–4^	(239)
**7-188**	SY1	–5.68	–3.95		PM6	1:1.2	0.87	25.4	76	16.8	5.4 × 10^–4^/3.1 × 10^–4^	(222)
**7-189**	SY3	–5.69	–3.98		PM6	1:1.2	0.86	25.5	74	16.2	6.5 × 10^–4^/3.6 × 10^–4^	(222)
**7-190**	BTP-S1	–5.55	–4.01	1.49	PM6	1:1	0.93	22.4	73	15.2	-/4.2 × 10^–4^	(240)
**7-191**	BTP-S2	–5.65	–4.01	1.48	PM6	1:1.2	0.95	24.1	72	16.4	-/8.3 × 10^–4^	(240)
**7-192**	BTP-ClBr	–5.79	–4.00	1.38	PM6	1:1.2	0.91	23.5	79	16.8	5.2 × 10^–4^/4.1 × 10^–4^	(241)
**7-193**	BTP-ClBr1	–5.79	–4.03	1.33	PM6	1:1.2	0.85	23.7	72	14.6	6.1 × 10^–4^/4.3 × 10^–4^	(241)
**7-194**	BTP-ClBr2	–5.80	–4.04	1.33	PM6	1:1.2	0.85	25.0	74	15.5	5.7 × 10^–4^/4.2 × 10^–4^	(241)
**7-195**	ZY-4Cl	–5.64	–3.67		P3HT	1:1	0.88	16.5	65	9.46	-/3.6 × 10^–5^	(242)
**7-196**	BTP-IS	–5.65	–4.02	1.38	PM6	1:1.2	0.89	22.6	64	12.8	7.7 × 10^–5^/2.0 × 10^–4^	(210)
**7-197**	Y10	–5.56	–3.76	1.35	J11	1:1.2	0.89	21.2	72	13.5	-/4.2 × 10^–4^	(243)
	Y6-T	–5.51	–3.90	1.35	PBDT-ST	1:1.2	0.92	22.6	70	14.4	-/3.8 × 10^–4^	(250)
**7-198**	BTCT-2Cl	–5.56	–3.95	1.37	PM6	1:1	0.88	24.4	70	15.1		(244)
**7-199**	BTP-2F-ThCl	–5.70	–3.99	1.34	PM6	1:1.2	0.87	25.4	77	17.1	5.6 × 10^–4^/3.3 × 10^–4^	(245)
**7-200**	TPBT-RCN	–5.42	–3.71^b^	1.71	P3HT	1:1.5	0.81	10.3	61	5.11	-/5.3 × 10^–4^	(246)

a Obtained from the oxidation/reduction potential of the CV measurement if not otherwise stated.

b HOMO/LUMO energy levels obtained from the LUMO/HOMO levels determined by CV and the optical band gap.

c Other method or method not defined.

d Determined via the SCLC technique from the neat acceptor/donor:acceptor blend films if not otherwise stated.

e Determined by other methods than SCLC.

**Table 7:** Optical, Electrical, and Photovoltaic Properties of the Non-Fullerene Acceptors **7-201**–**7-222**

NFA	original name	HOMO^a^ (eV)	LUMO^a^ (eV)	*E*_g_^opt^ (eV)	donor	D:A ratio	*V*_OC_ (V)	*J*_SC_(mA cm^–2^)	FF (%)	PCE (%)	μ_e_^b^(cm^2^ V^–1^ s^–1^)	ref.
**7-201**	BPF-4F	–5.58	–3.98	1.36	SZ5	1:1.2	0.85	22.1	67	12.6	-/3.2 × 10^–4^	(251)
**7-202**	BPT-4F	–5.59	–4.00	1.36	SZ5	1:1.2	0.85	24.8	79	16.8	-/6.4 × 10^–4^	(251)
**7-203**	BPS-4F	–5.54	–4.00	1.29	SZ5	1:1.2	0.82	25.4	78	16.3	-/4.8 × 10^–4^	(251)
**7-204**	CH1007	–5.59	–3.97	1.30	PM6	1:1.2	0.82	27.0	72	16.0	1.1 × 10^–4^/-	(252)
**7-205**	Y6-2Se	–5.58	–3.84	1.34	PM6	1:1.2	0.83	24.3	70	14.6	-/3.3 × 10^–4^	(253)
**7-206**	Y6Se	–5.70	–4.15	1.32	D18	1:1.6	0.84	28.0	75	17.7	2.7 × 10^–4^/-	(254)
**7-207**	Y3	–5.56	–4.03	1.32	PM6	1:1.5	0.81	24.1	66	13.2	-/7.5 × 10^–5^	(255)
	Y1-4F	–5.56	–4.11	1.31	PM6	1:1	0.83	25.2	69	14.8	-/3.0 × 10^–4^	(256)
**7-208**	Y18	–5.58	–3.91	1.31	PM6	1:1.5	0.84	25.7	77	16.5	-/3.5 × 10^–4^	(247)
**7-209**	Y18-DMO	–5.63	–3.90	1.37	PBDB-T	1:1.5	0.81	24.0	73	14.2	-/3.5 × 10^–4^	(260)
**7-210**	Y11	–5.61	–3.92	1.32	PM6	1:1.5	0.84	24.6	73	15.3	-/6.2 × 10^–4^	(255)
	Y11	–5.69	–3.87	1.31	PM6		0.83	26.7	74	16.5		(259)
**7-211**	Y19	–5.68	–3.95	1.31	PM6	1:1	0.84	22.4	68	12.8	-/6.5 × 10^–4^	(257)
**7-212**	Y15	–5.56	–3.93	1.30	PM6	1:1.8	0.88	23.8	69	14.1	-/5.2 × 10^–4^	(258)
**7-213**	Y14	–5.56	–4.01	1.30	PBDB-T		0.80	26.2	72	14.9	-/1.0 × 10^–4^	(261)
**7-214**	Y2	–5.43	–4.04	1.40	PBDB-T	1:1	0.82	23.6	69	13.4	-/2.1 × 10^–4^	(206)
**7-215**	QIP-4F	–5.75	–3.86	1.54	P2F-EHp	1:1	0.94	18.3	71	12.1	-/4.7 × 10^–4^	(262)
**7-216**	QIP-4Cl	–5.77	–3.89	1.48	P2F-EHp	1:1	0.94	19.6	72	13.3	-/5.3 × 10^–4^	(262)
**7-217**	AQx-2	–5.62	–3.88	1.35	PM6	1:1.2	0.86	25.4	76	16.6	-/2.9 × 10^–4^	(263)
**7-218**	AQx-1	–5.59	–3.85	1.35	PM6	1:1.2	0.89	22.2	67	13.3	-/3.7 × 10^–4^	(263)
**7-219**	TPQx-4F	–5.57	–3.74	1.41	PM6	1:1.2	0.94	15.0	54	7.72	-/4.3 × 10^–4^	(264)
**7-220**	TPQx-6F	–5.61	–3.78	1.43	PM6	1:1.2	0.92	21.8	71	14.3	-/4.6 × 10^–4^	(264)
**7-221**	Y18-ID	–5.43	–3.75	1.28	P	1:1.5	0.84	24.5	74	15.3	-/4.2 × 10^–4^	(265)
**7-222**	BDTP-4F	–5.61	–3.90	1.36	PM6	1:1.2	0.90	22.5	76	15.2	7.2 × 10^–4^/4.1 × 10^–4^	(266)

a Obtained from the oxidation/reduction potential of the CV measurement if not otherwise stated.

b Determined via the SCLC technique from the neat acceptor/donor:acceptor blend films if not otherwise stated.

The most common backbone type is based on BT as DA′D units, as already discussed with Y5 (**7-143**). This structural motif was systematically modified by introducing halogens on the accepting units, by changing the side chains, etc. Introducing INCN-2F units leads to the structure Y6 (**7-145**). As discussed above for ITIC derivatives, this modification leads to a lowering of the HOMO/LUMO energy levels and additionally to a slightly decreased optical band gap (1.33 eV) compared to the parent structure Y5 (1.38 eV) but also to the corresponding ITIC-4F (**7-4**). Solar cells based on a blend of **7-145**/PM6 give high PCE values of 15.7% compared to 13.6% revealed by similar devices with **7-4**/PM6. Although slightly lowering the *V*_OC_, the lower band gap allows efficient light harvesting above 900 nm and enables consequently high *J*_SC_ values of 25 mA cm^–2^. Liu et al. investigated a new polymer donor, D18, and applied it in solar cells with Y6, leading to a PCE of 18.2%, which is the highest efficiency for single junction organic solar cells by now. This PCE is accompanied by a high *J*_SC_ of 27.7 mA cm^–2^ and a FF of 77%. The *V*_OC_ is limited by the narrow band gap and reached therefore not more than 0.86 V.^1^

The exchange of the ethylhexyl groups of the central *N*-alkyl side chains in **7-145** leads to the molecules **7-146**–**7-153**. The substitution with ethylheptyl (**7-146**, Y6-C2),^215^ ethyloctyl (**7-147**, Y6-C3),^216^ or butyloctyl (**7-148**, BTP-4F-12)^217^ chains does not significantly affect the energy levels or the optical band gap but the crystallinity of the materials as well as their molecular packing order. **7-145**, **7-146**, and **7-148** show favorable face-on packing in neat film, whereas **7-147** has edge-on orientations, which is worse for vertical charge transport. **7-146** also shows a stronger π–π stacking and crystallization tendency. These results are consistent with the PV parameters; **7-146** gives the highest PCE of 16.3%, followed by **7-145** with 15.7% and **7-147** with 13.8%.^216^ NFA **7-146** was also tested using a modification of PM6 (addition of an iridium complex into the polymer backbone), leading to an enhanced efficiency of 17.1%. Also, the FF reached outstanding 78%, while the *V*_OC_ is at 0.84 V.^215^ When the ethyloctyl side chain is changed to a butyloctyl, extending the alkyl chain length (**7-148**), the efficiency of the solar cells is also enhanced to 16.4%.^217^ The replacement of the butyloctyl chain to an unbranched octyl group leads to structure **7-149** (Y6-nC8).^218^ Again, the energy levels and the band gap are, compared to Y6, not affected by this change. However, the PV parameters of PM6/**7-149**-based solar cells are lower than those for **7-145**–**7-148** with a PCE of 10.4%. Upon the introduction of phenyl rings at the end of the alkyl side chains as in **7-150** (Y6-phC6)^218^ and **7-151** (Y6-phOC6),^218^ the energy levels and band gaps stay similar to **7-149**. The PCEs of both acceptors with PM6 are enhanced to 11.8% for **7-150** and 11.1% for **7-151**, whereas the additional oxygen in the **7-151** side chains lead to the lower efficiency, as well as lower *J*_SC_ and FF. GIWAXS data of the neat acceptor films reveal a preferred “slant orientation” of **7-150**, which is neither edge-on nor face-on. A similar characteristic, but not as distinctive, was observed in NFA **7-149**.^218^ Han et al. investigated **7-150** (C6) and compared it with the counterpart **7-152** (C4)^219^ with shorter side chains on the nitrogen of the pyrrole units. As before, the energy levels and band gap are not affected, but the PV performance drops from 14.5% for **7-150** to 7.92% for **7-152** in PM6/NFA-based devices. Reasons therefore may lie in the lower absorption of **7-152** compared to **7-150**, as well as in the more pronounced face-on orientation of the PM6/**7-150** blend film which is beneficial for charge transport.^219^ In **7-153** (DTY6),^220^ 2-decyltetradecyl is implemented as side chains on the pyrroles. The longer branched alkyl chains lead to a good device performance with PM6 reaching an efficiency of 16.3% when processed in chloroform. The same blend was processed in xylene too, giving a PCE as high as 16.1%. GIWAXS data also revealed a similar pattern of the chloroform and xylene processed solar cell devices with favored face-on orientation and alike line-cut profiles.^220^

In molecules **7-154**–**7-156**, the alkyl chains on the pyrroles from Y6 remained while the outer alkyl chains were adjusted. Replacing the undecyl chains with undecylthio chains leads to structure **7-154** (BTPS-4F).^221^ The incorporation of sulfur upshifts the LUMO energy level, thus widening the band gap to 1.38 eV compared to Y6 with 1.33 eV. Solar cells with PM6 gave similar PCE values of 16.2% as Y6.^221,222^ Chai et al. used hexylphenyl (**7-155**, BTP-PhC6) or phenylhexyl (**7-156**, BTP-C6Ph) chains as outer side chains. Compared to Y6, the energy levels of both acceptors are heightened, and the optical band gap is slightly broadened. The PM6/**7-155** blend film shows a higher electron mobility than the PM6/**7-156** films. In addition, TEM images of PM6/**7-155** show a smaller phase separation and clearer interpenetrating network nanostructures than PM6/**7-156**. These investigations as well as the GIWAXS pattern give rise to the higher PCE of solar cells with PM6/**7-155** reaching values up to 16.7% compared to those of **7-156** with a PCE of 15.5%.^223^ Switching ethylhexyl and undecyl side chains in Y6 (**7-145**) leads to the structure **7-157** (N-C11)^224^ with an inverse substitution pattern. However, this affects the packing in a negative way, so that solar cells with PM6 showed decreased PCE values reaching 12.9%.^217,224^

Substitution of the fluorides in **7-145** (Y6) by chlorides leads to structure **7-158** (BTP-4Cl),^225^ and in analogy, variations on the *N*-alkyl side chains lead to molecules **7-159**–**7-162**. Cui et al. investigated the difference between **7-158** and **7-145** in detail. The absorption spectra measured in neat film show a red-shift of **7-158** and a narrower absorption. In general, the chlorine attachment increases the PCE by about 1% under optimal conditions with enhanced *V*_OC_, *J*_SC_, and FF.^225^ Extending the alkyl chain like in **7-159** (N3-4Cl)^226^ does not significantly influence the PV parameters, resulting in identical PCE values of 16.5% for solar cells based on **7-158** or **7-159** and PM6.^226^

The structures **7-160** (BTP-4Cl-12),^227^**7-161** (BTP-4Cl-16),^227^ and **7-162** (BTIC-C12-4Cl)^228^ have even more extended side chains, i.e., butyloctyl, hexyldecyl, and dodecyl chains, respectively. Solar cells of **7-160** showed increased PV parameters with outstanding efficiencies of 17.0–17.3%, whereas those with **7-161** and **7-162** have significantly lower PCEs of 15.6 and 11.4%, respectively (PM6 is used as a donor in all three cases).^227,229^ In the chlorinated NFAs **7-163**–**7-170**, the outer alkyl chain was changed from undecyl to undecylthio or other alkyl substituents with different chain lengths. In **7-163** (BTPS-4Cl),^221^ undecylthio side chains were introduced, like in the fluorinated counterpart **7-154**. As in **7-154**, the LUMO energy level of **7-163** is upshifted compared to Y6, and thereby slightly broadening the band gap to 1.36 eV. Solar cells with PM6 exhibit lower PV parameters compared to **7-154**, reaching a PCE of 13.5%. Mo et al. designed and synthesized the molecules **7-164**–**7-166**, which contain hexyl chains on the outer thiophene ring and alkyl chains differing in their chain length on the nitrogen atom of the pyrrole units. Compared to Y6, the HOMO energy level of all three acceptors is heightened slightly and the optical band gap is similar at 1.35 eV for all three compounds. In solar cells with the polymer PM6, **7-164** (BT_6_IC-BO-4Cl)^230^ showed a PCE of 14.4%, **7-165** (BT_6_IC-HD-4Cl)^230^ reached 14.9%, and **7-166** (BT_6_IC-OD-4Cl)^230^ only 9.60%. One reason for the lower efficiency in PM6/**7-166** cells is the by a factor of 10 lower electron mobility of the blend film, as well as the low *J*_SC_ and FF values. Interestingly, **7-166** possesses the highest extinction coefficient followed by **7-164** and **7-165**.^230^ Cui et al. used butyloctyl *N*-alkyl chains in **7-167** (BTP-eC7)^231^ and **7-168** (BTP-eC9)^231^ and changed the outer substituents from a heptyl to a nonyl chain, respectively. The energy levels of both acceptors are similar to Y6, but the PV parameters with the donor PM6 deviate from each other. **7-167** and **7-168** both show a *V*_OC_ of 0.84 V and *J*_SC_s of over 24 mA cm^–2^. However, the FF of **7-168** based solar cells is with a value of 81% much higher, which leads to a PCE of 17.8% compared to **7-167** revealing a FF of 74% and a PCE of 14.9%. Molecules **7-169** (HD-4Cl)^232^ and **7-170** (HDO-4Cl)^232^ have a hexyldecyl chain on the nitrogen atom as well as decyl and decyloxy chains on the outer donor unit, respectively. **7-170** has a higher LUMO level than **7-169** due to the additional oxygen in the side chain. Blended with PM6, both show good performance with a PCE over 15.6%.^232^

NFAs **7-171** (BTIC-4Br)^233^ and **7-172** (BTIC-BO-4Br)^233^ are brominated structures, with either ethylhexyl or butyloctyl groups as *N*-alkyl side chains. Compared to their chlorinated and fluorinated counterpart, the HOMO energy level is slightly upshifted. Blended with PM6, **7-171** and **7-172** gave *V*_OC_’s of 0.85 and 0.86 V, *J*_SC_’s of 20.7 and 24.1 mA cm^–2^, FFs of 70 and 68%, and PCEs of 12.2 and 14.0%, respectively. Similarly, as found for the chlorinated and fluorinated counterparts, the acceptor with the butyloctyl chains gives a higher efficiency.^233^

The series **7-173**–**7-178** shows the difference of monosubstitution on the peripheral INCN units with either fluorine **7-173** (Y8),^234^ chlorine **7-174** (BTIC-Cl-*m*),^235^ bromine **7-175** (BTIC-2Br-*m*),^233^ trifluoromethyl, where **7-176** (BTIC-CF_3_-*m*)^235^ is an isomer mixture of γ- and δ-substituted acceptors and **7-177** (BTIC-CF_3_-γ)^235^ is the pure γ-isomer, and a methyl group **7-178** (BTP-M).^236^ The side chains of these NFAs are the same as in **7-143** (Y5) with undecyl and ethylhexyl chains. All acceptors exhibit downshifted LUMO levels in the order from −Me (−3.81 eV), −F (−3.89 eV), −Cl (−3.91 eV), −CF_3_ (−3.97 eV), and −Br (−3.97 eV) compared to **7-143** (−3.78 eV). The HOMO levels show similar values of around −5.45 eV for **7-174** and **7-176**–**7-178**.^234−236^ For **7-173**, conflicting values of −5.55 eV are reported,^234^ which are in the range as for **7-143** and **7-175**. All six acceptors were blended with PM6 and investigated in PV devices, which are showing PCE values above 13% except for **7-178** (4.26%). Their *V*_OC_ and FF values resemble each other (again except for **7-178**), with *V*_OC_ values between 0.85 and 0.88 V and FFs above 70%.^235,236^

**7-173** shows a PCE of 14.3%, which is lower than the corresponding acceptor containing two fluorines on each side.^234^ The highest PCE of these five acceptors was reached by solar cells of the brominated **7-175**. The obtained efficiency was 16.1% correlating with excellent *J*_SC_ values of 25 mA cm^–2^, which is also enhanced compared to the other brominated acceptors **7-171** and **7-172**.^233^ Molecules **7-176** and **7-177**, having a trifluoromethyl group attached on the INCN phenyl ring, show the same high *J*_SC_ values, but due to lower *V*_OC_s, the solar cells exhibit up to 15.6% efficiency.^235^

**7-179**–**7-184** are a group of acceptors with one halogen on each end group but on different specific positions and with deviations in the *N*-alkyl chains. **7-179** (BTPIC-2Br-5)^237^ and **7-180** (BTPIC-2Br-6)^237^ share the same ethylhexyl chains on the nitrogen atom and have a bromine on each end group in position X and Y, respectively. They have similar optical band gaps and HOMO/LUMO energy levels, where the LUMO levels are upshifted compared to Y6. Solar cells were built with PM6; the devices yield similar *V*_OC_s, but PM6/**7-179** has higher *J*_SC_, FF, and PCE values. **7-179** also shows a higher electron mobility in the blend film as well as weaker trap-assisted recombination in the devices.^237^ Molecules **7-181** (LY-Cl-1)^238^ and **7-182** (LY-Cl-2)^238^ are the counterparts of **7-179** and **7-180**, respectively, with chlorine atoms attached on the end group. Compared to **7-179** and **7-180**, the HOMO levels are slightly lowered and the LUMO levels heightened, which leads to higher optical band gaps of 1.39 and 1.36 eV for **7-181** and **7-182**, respectively. Photovoltaic parameters with PM6 show again higher FF and PCE values for **7-182**, as well as a higher electron mobility, while **7-181** reveals a slightly higher *V*_OC_ and *J*_SC_. Li et al. also investigated the structures **7-183** (N3-Cl-1)^238^ and **7-184** (N3-Cl-2),^238^ which resemble **7-181** and **7-182** with longer *N*-alkyl chains. With ethylheptyl chains, the energy levels are slightly upshifted and the band gap narrowed. However, in solar cells with PL1, a similar trend is observed as for **7-181** and **7-182** with the difference that the PCEs are higher for both compounds.^238^

NFAs **7-185**–**7-188** have similar side chains but are asymmetric, concerning their acceptor units, as they have different atoms attached on each side. **7-185** (SY2)^222^ and **7-186** (BTIC-γCl-2F)^116^ share two fluorines on one end group and have two or one chlorine on the other (**7-185** and **7-186**), respectively. The results of **7-185** and **7-186** are difficult to compare, as completely different device structures were used for very similar molecules. However, in combination with PM6, **7-185** and **7-186** reached high FFs and PCEs of over 70% and over 15%, respectively.^116,222^ In **7-187** (BTIC-2Cl-γCF_3_),^239^ the fluorines from **7-186** were exchanged with chlorine atoms and the chlorine from **7-186** was replaced with a trifluoromethyl group. Photovoltaic cells based on PM6/**7-187** achieved a good efficiency of 16.3% with a high FF of 77%.^239^ Liu et al. investigated three different molecules, **7-185**, **7-188** (SY1),^222^ and **7-189** (SY3),^222^ where **7-188** and **7-189** contain one chlorine atom on one end group (on no specific position) and on the other side they have two fluorines or chlorines, respectively. Their HOMO/LUMO energy levels are in the same range, and the solar cells were built with PM6 as the donor. The photovoltaic parameters of all three combinations have *V*_OC_s over 0.85 V, *J*_SC_s over 25 mA cm^–2^, FFs over 74%, and PCEs over 16%. With 16.8%, **7-188** reached, compared to **7-185** and **7-189**, the highest efficiency.^222^ Other asymmetric electron acceptors were introduced by Li et al. where four chlorines are attached on one INCN unit and two fluorines or chlorines on the other (**7-190**, BTP-S1,^240^ and **7-191**, BTP-S2,^240^ respectively). In addition, the two molecules have different *N*-alkyl chain lengths. The HOMO energy level of **7-190** is heightened compared to **7-191**, and the LUMO energy levels of both acceptors are higher than in Y6. This is also the reason for the higher *V*_OC_s in combination with PM6, which are between 0.93 and 0.95 V for **7-190**- and **7-191**-based solar cells. The devices show also similar FFs, but the *J*_SC_ and PCE are 14.1 mA cm^–2^ and 16.4% for **7-191**, which is elevated compared to **7-190**.^240^

Luo et al. designed and synthesized three molecules **7-192**–**7-194** resembling **7-191** in their side chains and differing in the halogens attached on the INCN units. All three acceptors have one bromine and one chlorine atom on each end group on different positions on the phenyl ring. In **7-192** (BTP-ClBr),^241^ the bromine and chlorine atoms occupy positions next to each other, in **7-193** (BTP-ClBr1),^241^ they are randomly attached, whereas, in **7-194** (BTP-ClBr2),^241^ the halogens are attached in *meta*-substitution. The HOMO/LUMO energy levels are similar for all three acceptors, but the optical band gap has a higher value of 1.38 eV in **7-192** than for **7-193** and **7-194**. The difference in the optical band gap indicates a stronger π–π stacking of **7-193** and **7-194**, which is also consistent with the obtained GIWAXS data. Solar cells of PM6/**7-192** reached a *V*_OC_ of 0.91 V, an outstanding FF of 79%, and an efficiency of 16.8%. **7-193** and **7-194** have higher values for the *J*_SC_ but lower ones for *V*_OC_, FF, and PCE.^241^

Replacing the INCN units with other electron-accepting groups leads to the structures **7-195**–**7-200**, which all contain undecyl side chains and either ethylhexyl or butyloctyl *N*-alkyl chains. In acceptor **7-195** (ZY-4Cl),^242^ the malononitrile group in **7-160** was exchanged with a carbonyl unit, which heightens the LUMO level, whereas the HOMO energy level is similar in both **7-195** and **7-160**. Due to the use of a different polymer donor (P3HT in **7-195** and PM6 in **7-160**), the photovoltaic parameters are not easy to compare. However, P3HT/**7-195**-based devices possess a similar *V*_OC_ as solar cells with PM6/**7-160** absorber layers, but all other parameters are much lower with an efficiency of 9.46%. Exchanging the carbonyl unit at the INCN end groups of **7-144** with a sulfone group leads to **7-196** (BTP-IS).^210^ In comparison, the sulfone lowers both energy levels and gives a slightly lower band gap than **7-144**. Combined with PM6, the **7-196**-based device shows lower *V*_OC_ but higher *J*_SC_, FF, and PCE values, enhancing the efficiency from 7.54 to 12.8%. A reason therefore can be the much higher electron mobility in the blend film of **7-196** compared to **7-144**, as well as the more effective exciton dissociation and charge collection.^210^ The exchange of the benzene unit of the INCN group with a [*c*]-thiophene ring leads to the structures **7-197** (Y10)^243^ and **7-198** (BTCT-2Cl)^244^ with the latter having the same undecyl and ethylhexyl side chains as in **7-143** and **7-198** having additional chlorines on the acceptor units. The band gaps for **7-197** and **7-198** are 1.35–1.37 eV; their HOMO/LUMO energy levels are −5.56 eV/–3.76 eV and −5.56 eV/–3.95 eV, respectively. Solar cells were built with different polymers, namely, J11 and PM6, but in both cases, very similar *V*_OC_ and FF values were reached; the PCE was higher for **7-198** (15.1% vs 13.5%) due to the higher *J*_SC_.^243,244^ The end groups in structure **7-199** (BTP-2F-ThCl)^245^ are a combination of the acceptor units of Y6 (**7-145**) and **7-198**, having an INCN-2F unit on one side and a CPTCN-Cl end group on the other side. The energy levels as well as the optical band gap and absorption maxima of **7-199** lie between the values from **7-145** and **7-198**. However, when it comes to the PV parameters in combination with PM6, **7-199** outperforms both **7-145** and **7-198** regarding the *J*_SC_, FF, and PCE, giving an efficiency of over 17% (**7-145** with 16.4% and **7-198** with 14.5%). This study shows that asymmetric end group engineering may be an efficient way for enhancing the PCE.^245^ Yang et al. introduced RCN end groups on the Y backbone structure (**7-200**, TPBT-RCN),^246^ which heightens both energy levels compared to Y6 and thereby enlarges the band gap to 1.71 eV. In solar cells, **7-200** was blended with P3HT, which yielded 5.11% efficiency. Compared to the P3HT/Y6 blend, the electron mobility and PCE were enhanced.^246^

Chai et al. developed the molecules **7-201**–**7-203**, which again have INCN-2F acceptor units but lack the outer alkyl chains usually present in the Y backbone structure (see Figure 11 and Table 7). Moreover, the thiophene rings of the base structure **7-202** (BPT-4F)^251^ were replaced with either furans (**7-201**, BPF-4F)^251^ or selenophenes (**7-203**, BPS-4F).^251^ All three molecules show similar LUMO energy levels, whereas the HOMO energy level of **7-203** is shifted upward, thus narrowing the optical band gap. The efficiency of **7-201**/SZ5-based solar cells is lower (12.6%) than that for **7-202**/SZ5- and **7-203**/SZ5-based devices (16.8 and 16.3%, respectively). This is attributed to the lower electron mobility and worse blend film morphology, due to a less coplanar geometry of **7-201**. Differences in **7-202** and **7-203** lie in lower EQE values for **7-203** due to inefficient charge transport compared to **7-202**.^251^ Structure **7-204** (CH1007)^252^ is the selenium analogue of **7-148**, with undecylselenophene outer rings and *N*-BO side chains. The HOMO/LUMO energy levels as well as the optical band gap are similar to those of **7-203**. Solar cells based on **7-204** and PM6 exhibit a lower *V*_OC_ and FF but a higher *J*_SC_ (27.0 mA cm^–2^), yielding only a slightly lower overall PCE value than devices of PM6/**7-148**.^252^ Yu et al. introduced the selenium either by exchanging the inner thiophene of the Y-backbone with selenophene leading to structure **7-205** (Y6-2Se)^253^ or by changing the central benzothiadiazole to a benzoselenadiazole leading to structure **7-206** (Y6-Se).^253^ These particular molecules have, as the other selenium-containing compounds, slightly upshifted energy levels but similar optical band gaps as Y6. In combination with PM6, both reach a similar *V*_OC_, but **7-206** shows a higher *J*_SC_, FF, and PCE of 25.5 mA cm^–2^, 75, and 15.8%, respectively. Furthermore, the group of Zhang et al. combined **7-206** with D18 and achieved even higher PCEs up to 17.7%.^254^

The influence of halogenation as well as side chain variation was also investigated on benzotriazole-based acceptors of this type, leading to the structures **7-207**–**7-213**. In all cases (except **7-209**), an ethylhexyl side chain is attached on the central nitrogen atom. Their HOMO energy levels lie for all halogenated acceptors between −5.56 and −5.69 eV, and their LUMO energy levels between −3.87 and −4.11 eV. Compared to the parent non-halogenated structures **7-140** and **7-142**, all of them exhibit low and similar band gaps of about 1.30 eV (except **7-209** with 1.37 eV). All acceptors, except **7-209** and **7-213**, were blended with PM6 for the preparation of solar cells. **7-207** (Y3)^255,256^ derives from **7-140** with no alkyl chains on the outer thiophene rings but with INCN-2F units. Overall, the fluorination increased the PCE in this case from 13.4% for **7-140** to 14.8% for **7-207**.^206,256^ The lower band gap leads to an increased photocurrent due to the higher absorption range but to lower *V*_OC_ values.

Starting from the parental structure **7-142**, the structures **7-210** (Y11),^255^**7-211** (Y19),^257^ and **7-212** (Y15)^258^ contain INCN-2F or INCN-2Cl units and differ in their alkyl chain lengths. In contrast to **7-207**, halogenation leads in both **7-210** and **7-212** to enhanced photovoltaic properties and PCE values of 16.5 and 14.1%, respectively.^255,258,259^**7-211** with shorter alkyl chains yielded 12.8%. The chlorinated structures **7-211** and **7-212** reveal higher *V*_OC_s but lower *J*_SC_s and FFs. By exchanging the undecyl groups in **7-210** with shorter hexyl groups, the structure **7-208** (Y18)^247^ is obtained; however, the difference in the PV parameters of **7-208** and **7-210** is only marginal.^247^**7-209** (Y18-DMO)^260^ differs from **7-208** by longer *N*-alkyl chains, which leads, in combination with PBDB-T, to overall lower PV parameters than PM6/**7-209**-based devices.^260^**7-213** (Y14)^261^ is the monofluorinated counterpart of **7-210**. Solar cells of this acceptor with PBDB-T showed a *V*_OC_ of 0.80 V, a high *J*_SC_ of 26.2 mA cm^–2^, a FF of 72%, and a PCE of 14.9%, which are, except for the *J*_SC_, all slightly lower compared to **7-210**.^261^

Yuan et al. developed the molecule **7-214** (Y2),^206^ which resembled **7-197**, with a benzotriazole instead of a BT unit and without the outer undecyl chains. Compared to **7-197**, the HOMO/LUMO energy levels of **7-214** are further heightened and the optical band gap is widened to 1.40 eV. Solar cell devices were built with PBDB-T**/7-214**, which yielded an efficiency of 13.4%.^206^

Finally, as an alternative A′ unit in the (DA′D) core, quinoxaline (1,4-benzopyrazine) was introduced instead of benzotriazole or benzothiadiazole, leading to the structures **7-215**–**7-220**. All six of them exhibit INCN-2X units and differ from each other in their backbone. **7-215** (QIP-4F)^262^ and **7-216** (QIP-4Cl)^262^ possess *N*-ethylhexyl quinoxaline-2,3-dicarboxylic acid amide as a central unit and either fluorines or chlorines on the INCN unit. This new backbone design leads to downshifted HOMO and upshifted LUMO energy levels compared to similar acceptors (**7-145**, **7-158**, **7-210**, **7-212**), which also increases the optical band gap of the materials. In organic solar cells, the acceptors were blended with a new polymer called P2F-EHp. The devices give a high *V*_OC_ of 0.94 V, a FF of about 70%, and PCEs of 12.1% for the fluorinated acceptor and 13.3% for the chlorinated acceptor.^262^**7-217** (AQx-2),^263^**7-218** (AQx-1),^263^**7-219** (TPQx-4F),^264^ and **7-220** (TPQx-6F)^264^ have a quinoxaline unit in the center with or without two methyl groups on the pyrazine ring (**7-217** and **7-218**) or ethylhexylthienyl as well as fluorinated ethylhexylthienyl groups (**7-219** and **7-220**). These acceptors have about the same HOMO levels as the other Y backbone structures but have upshifted LUMO levels. The optical band gaps lie at about 1.40 eV for all four materials. The PV parameters of devices with PM6 as a donor deviate highly from each other, except for the *V*_OC_, which lies between 0.86 and 0.89 V for **7-217** and **7-218** and between 0.92 and 0.94 V for **7-219** and **7-220**.^263,264^ Devices with **7-217** have the highest *J*_SC_, FF, and PCE and revealed 16.6% efficiency, while those with **7-218** only reached 13.3%. GIXD data revealed unbalanced crystallization properties of the donor/acceptor blend film with **7-218**, which leads to reduced *J*_SC_ and FF values. In addition, TEM images shows excessive aggregation of **7-218**, which leads to worse exciton diffusion and charge transport compared to **7-217**.^263^ Devices with **7-219** show a rather low efficiency of 7.72%, whereas the additional fluorines (in **7-220**) enhance the PCE to 14.3%, due to more homogenous phase separation as well as higher exciton dissociation efficiency.^264^ Chen et al. developed a new DA′D backbone (**7-221**, Y18-ID)^265^ by exchanging the thienothiophene rings of **7-208** with indole groups. This leads to heightened energy levels and a smaller optical band gap of 1.28 eV. Solar cells with the polymer donor P reach a *V*_OC_ of 0.84 V, a *J*_SC_ of 24.5 mA cm^–2^, a FF of 74%, and a PCE of 15.3%, which is lower compared to PM6/**7-208**.^265^ Luo et al. introduced an asymmetric DA′D-backbone acceptor, **7-222** (BDTP-4F),^266^ with a BT core, INCN-2F acceptor units, and alkyl side chains of different length. Compared to Y6, the HOMO/LUMO energy levels of **7-222** are upshifted accompanied by a slightly broadened optical band gap. Solar cells with PM6 as a donor gave a high *V*_OC_ of 0.90 V due to the broader band gap and a good PCE of 15.2%.

### 3.6 Other Seven-Ring Acceptors

**Figure 12:**
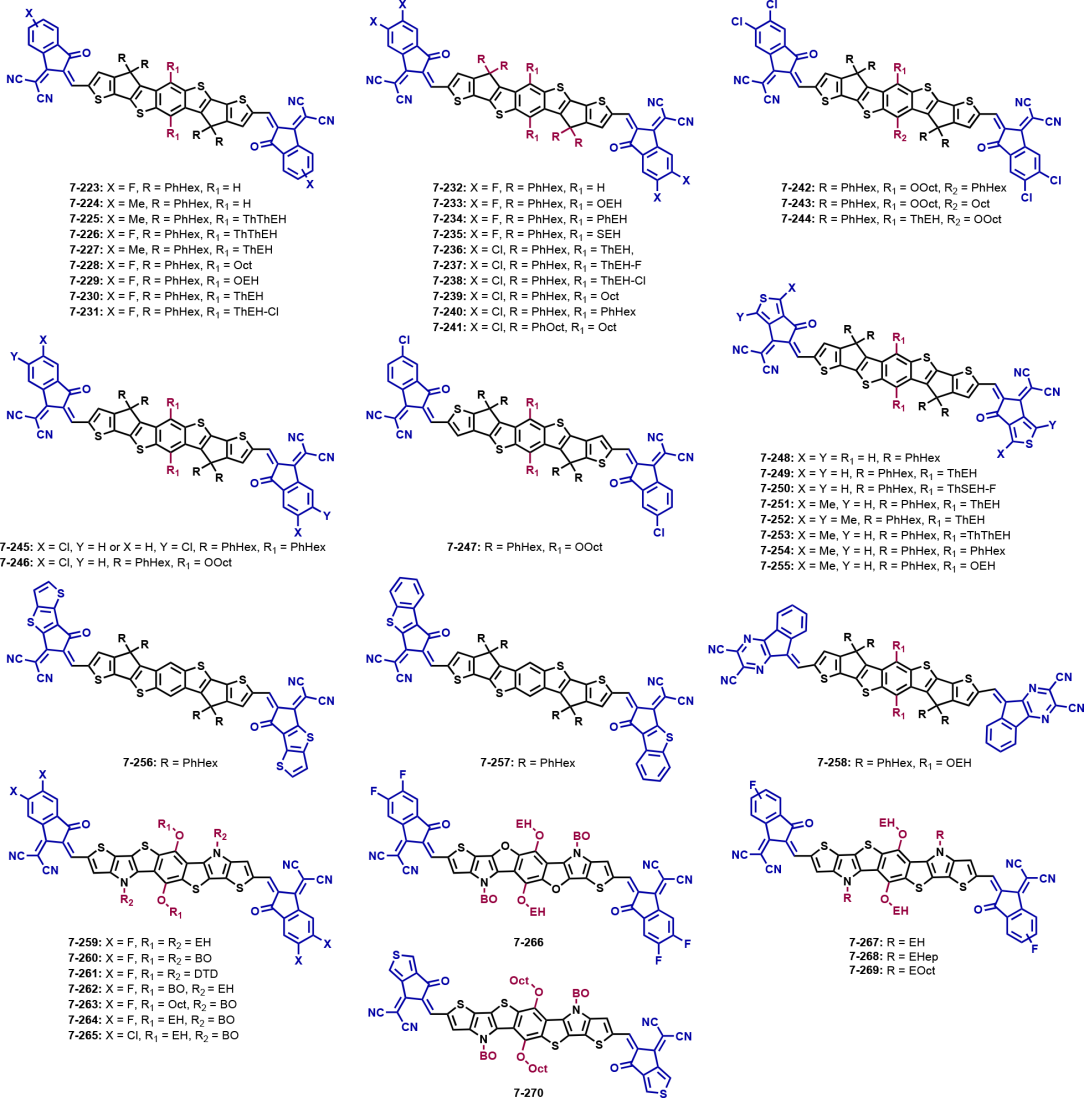
Structures of non-fullerene acceptors based on benzodi(cyclopentadithiophene) and related structures.

As discussed in the section “Impact of the Central Donor Unit”, a promising group of acceptors are based on the benzodi(cyclopentadithiophene) structure as a donor unit (see above **7-117**–**7-124**). Many structural variations of this donor motif have been presented in the last three years, as shown in Figure 12. Almost all benzodi(cyclopentadithiophene)-based NFAs depicted in Figure 12 comprise *p*-hexylphenyl side chains on the sp^3^ hybridized carbon on the cyclopentadiene ring. The structures differ in their substituents on the central phenyl ring and variations on the acceptor unit. The first nine acceptors (**7-223**–**7-231**) have INCN-F or methylated INCN (INCN-Me) end groups. Comparing structures **7-223** (NFBDT-F)^267^ and **7-224** (BDTIT-M),^268^ both exhibit similar HOMO/LUMO energy levels but the INCN-Me groups lead to a slightly larger band gap with a value of 1.55 eV in **7-224** compared to 1.50 eV in **7-223** (see also Table 8). Solar cells in combination with PBDB-T gave higher *V*_OC_ (0.90 V) but lower *J*_SC_ values (17.6 mA cm^–2^) for **7-224** compared to **7-223**. With a slightly better FF, solar cells of **7-224** show better PCEs with a maximum of 11.3% (10.6% for **7-223**). The introduction of 2-(2-ethylhexyl)thieno[3,2-*b*]thiophene side chains on the central benzene ring leads to the structure **7-225** (BTT-MIC)^269^ in the combination with INCN-F and to **7-226** (BTT-FIC)^269^ in combination with INCN-Me acceptor groups. The HOMO levels are downshifted for both structures and are exhibiting similar optical band gaps as **7-223** and **7-224**. Solar cells based on both acceptors were built with PM6 showing high *V*_OC_ values. Also, in this case, the methylated acceptor has a higher *V*_OC_ of 1.03 V compared to 0.95 V for the fluorinated acceptor, but due to higher *J*_SC_s and higher FFs, the fluorinated acceptor **7-226** outperforms the methylated one, giving PCEs of 12.6% compared to 10.0% for **7-225**.

**Table 8:** Optical, Electrical, and Photovoltaic Properties of the Non-Fullerene Acceptors **7-223**–**7-270**

NFA	original name	HOMO^a^ (eV)	LUMO^a^ (eV)	*E*_g_^opt^ (eV)	donor	D:A ratio	*V*_OC_ (V)	*J*_SC_(mA cm^–2^)	FF (%)	PCE (%)	μ_e_^c^(cm^2^ V^–1^ s^–1^)	ref.
**7-223**	NFBDT-F	–5.43	–3.88	1.50	PBDB-T	1:1	0.79	19.3	70	10.6	-/1.3 × 10^–4^	(267)
**7-224**	BDTIT-M	–5.40^b^	–3.85	1.55	PBDB-T	1:1	0.90	17.6	71	11.3	1.9 × 10^–3^/3.0 × 10^–4^	(268)
**7-225**	BTT-MIC	–5.55	–3.76	1.53	PM6	1:1	1.03	15.9	62	10.0	1.6 × 10^–3^/5.2 × 10^–4^	(269)
**7-226**	BTT-FIC	–5.64	–3.87	1.47	PM6	1:1	0.95	18.5	72	12.7	1.9 × 10^–3^/7.6 × 10^–4^	(269)
**7-227**	BDTThIT-M	–5.35^b^	–3.82	1.53	PBDB-T	1:1	0.94	18.0	71	12.1	2.1 × 10^–3^/3.9 × 10^–4^	(268)
**7-228**	NCBDT	–5.36	–3.89	1.45	PBDB-T	1:0.8	0.84	20.3	71	12.1	2.0 × 10^–4^/1.6 × 10^–4^	(270)
**7-229**	BT-SFIC	–5.46	–4.04	1.44	PTB7-Th	1:2.5	0.77	18.5	67	9.52	3.5 × 10^–4^/3.1 × 10^–4^	(271)
**7-230**	BTH-2F	–5.59	–3.91	1.50	PM6	1:1	0.92	19.5	63	11.3	7.1 × 10^–4^/5.6 × 10^–4^	(272)
**7-231**	BTC-2F	–5.64	–3.91	1.53	PM6	1:1	0.92	20.3	69	12.9	8.6 × 10^–4^/6.5 × 10^–4^	(272)
**7-232**	BDCPDT-FIC	–5.52	–4.00	1.49	PBDB-T	1:1	0.70	19.1	61	8.12		(273)
**7-233**	BT-FIC	–5.48	–4.10	1.39	PTB7-Th	1:1.5	0.73	21.3	65	10.1	3.8 × 10^–4^/2.0 × 10^–4^	(271)
**7-234**	BP-4F	–5.63	–3.90	1.49	PM6	1:1	0.90	21.6	72	13.9	2.1 × 10^–4^/2.8 × 10^–4^	(274)
**7-235**	SBT-FIC	–5.81	–4.15		PTB7-Th	1:2	0.70	18.1	62	7.90		(275)
**7-236**	HBDT-4Cl	–5.67	–3.90	1.43	PM6	1:1	0.90	17.8	65	10.4	-/1.2 × 10^–5^	(276)
**7-237**	FBDT-4Cl	–5.70	–3.91	1.45	PM6	1:1	0.89	19.8	70	12.4	-/5.0 × 10^–5^	(276)
**7-238**	ClBDT-4Cl	–5.72	–3.92	1.44	PM6	1:1	0.88	19.0	70	11.7	-/4.0 × 10^–5^	(276)
**7-239**	NCBDT-4Cl	–5.60	–4.02	1.40	PBDB-T-SF	1:1	0.85	22.4	74	14.1	-/1.9 × 10^–4^	(277)
**7-240**	DPBDT-4Cl	–5.62	–3.94	1.41	PM6	1:1	0.90	19.2	66	11.4	-/2.2 × 10^–4^	(279)
	BPIC-4Cl	–5.60	–4.08	1.43	PBDB-T	1:1	0.74	17.1	63	8.01	5.7 × 10^–4^/3.4 × 10^–4^	(189)
**7-241**	NCBDT-4Cl	–5.60	–4.02	1.40	PM6	1:1	0.83	20.1	72	12.0	-/2.0 × 10^–4^	(278)
**7-242**	POBDT-4Cl	–5.58	–3.97	1.39	PM6	1:1	0.88	21.0	68	12.6	-/3.6 × 10^–4^	(279)
**7-243**	COBDT-4Cl	–5.57	–3.98	1.39	PM6	1:1	0.87	21.8	71	13.5	-/3.7 × 10^–4^	(279)
**7-244**	TOBDT	–5.56	–3.97	1.41	PM6	1:1	0.89	18.7	68	11.3	-/3.1 × 10^–4^	(280)
**7-245**	BPIC-2Cl	–5.57	–3.90	1.47	PBDB-T	1:1	0.81	20.7	72	12.2	7.1 × 10^–4^/4.4 × 10^–4^	(189)
**7-246**	BDT_c_IC-γCl	–5.29	–4.01		PM6	1:1.2	0.89	13.8	62	7.61	-/4.2 × 10^–5^	(281)
**7-247**	BDT_t_IC-γCl	–5.21	–3.89		PM6	1:1.2	0.81	2.26	39	0.71	-/4.6 × 10^–6^	(281)
**7-248**	ITIC5	–5.48	–3.95	1.53	J71	1:1	0.90	18.5	76	12.5	1.2 × 10^–3^/8.3 × 10^–4^	(188)
**7-249**	BTTIC-0M	–5.62	–3.89	1.47	PBDB-T	1:1	0.86	19.0	73	11.9	6.7 × 10^–4^/3.7 × 10^–4^	(282)
**7-250**	BDTSF-IC	–5.58	–3.93	1.56	PM6	1:1	0.90	20.4	72	13.1	1.5 × 10^–4^/6.7 × 10^–4^	(283)
**7-251**	BTTIC-2M	–5.60	–3.86	1.47	PBDB-T	1:1	0.90	19.4	75	13.2	8.2 × 10^–4^/4.6 × 10^–4^	(282)
**7-252**	BTTIC-4M	–5.55	–3.79	1.49	PBDB-T	1:1	0.97	15.7	64	9.60	9.4 × 10^–4^/4.0 × 10^–4^	(282)
**7-253**	BTTIC-TT	–5.54	–3.80	1.47	PBDB-T	1:1	0.92	19.6	74	13.4	9.3 × 10^–4^/5.1 × 10^–4^	(284)
**7-254**	BTTIC-Ph	–5.48	–3.78	1.45	PBDB-T	1:1	0.93	16.5	60	9.14	7.3 × 10^–4^/4.1 × 10^–4^	(284)
**7-255**	BTOIC	–5.42	–3.94	1.39	PBDB-T	1:1	0.86	18.6	68	11.0	9.4 × 10^–4^/4.1 × 10^–4^	(285)
**7-256**	BDCPDT-TTC	–5.38	–3.78	1.58	PBDB-T	1:1	0.94	17.7	62	10.3		(273)
**7-257**	BDCPDT-BC	–5.40	–3.85	1.53	PBDB-T	1:1	0.92	18.6	63	10.8	-/1.4 × 10^–5^	(134)
**7-258**	BTOIPC	–5.47	–3.79	1.45	PBDB-T	1:1	0.88	15.2	70	9.31	-/5.2 × 10^–4^	(190)
**7-259**	M2	–5.60	–3.96	1.39	PM6	1:1	0.88	19.8	64	11.2	-/1.3 × 10^–4^	(286)
**7-260**	M36	–5.62	–3.95	1.39	PM6	1:1	0.90	24.6	72	16.0	-/5.8 × 10^–4^	(286)
**7-261**	M38	–5.65	–3.93	1.47	PM6	1:1	0.87	18.3	56	8.89	-/5.7 × 10^–5^	(286)
**7-262**	M4	–5.61	–3.97	1.38	PM6	1:1	0.88	23.4	72	14.8	-/4.2 × 10^–4^	(287)
**7-263**	BDTN-BF	–5.58	–3.90	1.41	PM6	1:1	0.93	20.2	62	11.5	9.6 × 10^–5^/2.9 × 10^–4^	(288)
**7-264**	BDTBO-4F	–5.52	–3.90	1.43	PM6	1:1	0.86	23.0	75	14.8	-/5.4 × 10^–4^	(289)
	M34	–5.60	–3.91	1.39	PM6	1:1	0.91	23.6	71	15.2	-/2.7 × 10^–4^	(290)
**7-265**	BDTBO-4Cl	–5.54	–3.94	1.39	PM6	1:1	0.83	23.6	71	13.9	-/3.8 × 10^–4^	(289)
**7-266**	M8	–5.49	–3.91	1.28	PM6	1:1	0.83	8.36	61	4.21	-/6.3 × 10^–5^	(290)
**7-267**	SNBDT1-F	–5.30	–3.83	1.41	PBDB-T	1:1	0.86	21.0	70	12.7	4.0 × 10^–5^/3.6 × 10^–5^	(291)
**7-268**	SNBDT2-F	–5.34	–3.82	1.41	PBDB-T	1:1	0.88	13.9	54	6.57	4.4 × 10^–5^/3.0 × 10^–5^	(291)
**7-269**	SNBDT3-F	–5.36	–3.80	1.41	PBDB-T	1:1	0.90	12.5	52	5.87	8.5 × 10^–5^/1.7 × 10^–5^	(291)
**7-270**	BDTN-Th	–5.50	–3.85	1.41	PM6	1:1	0.95	8.20	45	3.53	5.3 × 10^–5^/3.9 × 10^–5^	(288)

a Obtained from the oxidation/reduction potential of the CV measurement if not otherwise stated.

b HOMO/LUMO energy levels obtained from the LUMO/HOMO levels determined by CV and the optical band gap.

c Determined via the SCLC technique from the neat acceptor/donor:acceptor blend films if not otherwise stated.

The introduction of ethylhexylthienyl substituents on the central benzene ring in the methylated structure **7-224** leads to NFA **7-227** (BDTThIT-M),^268^ with a slightly reduced band gap. Solar cells with PBDB-T gave enhanced PV performance mainly due to slightly better *V*_OC_ values of 0.94 V for **7-227** instead of 0.90 V for **7-224** and better *J*_SC_ values (18.0 vs 17.6 mA cm^–2^).^268^ Modification of the fluorinated acceptors (**7-223**, **7-226**) by introduction of either octyl side chains or 2-ethylhexyloxy side chains on the central benzene ring leads to molecules **7-228** (NCBDT)^270^ and **7-229** (BT-SFIC),^271^ respectively, both having similar band gaps (1.45 and 1.44 eV). **7-228** reached quite promising PCEs of 12.1% in solar cells using PBDB-T as a donor polymer. Solar cells with acceptor **7-229** were prepared with PTB7-Th and led to lower PCEs of 9.52%. Ethylhexylthienyl and chlorinated ethylhexylthienyl groups on the central benzene ring result in the structures **7-230** (BTH-2F)^272^ and **7-231** (BTC-2F),^272^ respectively. Compared to **7-223** which has no additional side chains, both HOMO/LUMO energy levels are downshifted for **7-230** and **7-231**, whereas the HOMO energy level of **7-231** is further lowered due to the chlorine atom on the side chains. Solar cells in combination with PM6 show that the additional halogens increase the efficiency by more than 1%, leading to a PCE of 12.9% for **7-231** (11.3% for **7-230**).^272^

The molecule **7-232** (BDCPDT-FIC)^273^ contains INCN-2F units and is related to structure **7-223** (INCN-F groups). The additional fluorines lower both HOMO/LUMO energies. Solar cells with PBDB-T show a lower PCE of 8.12% for **7-232** than **7-223**, due to lower *V*_OC_ and FF values. However, different device architectures were used.^273^**7-233** (BT-FIC)^271^ is the counterpart of **7-229** with INCN-2F units. Once more, the additional fluorine lowers the energy levels and band gap and the efficiency is improved to 10.1%.^271^

**7-234** (BP-4F)^274^ and **7-235** (SBT-FIC)^275^ have ethylhexylphenyl and ethylhexylthio side chains, respectively. Compared to **7-232**, **7-234** shows similar HOMO and slightly higher LUMO energy levels with an optical band gap of 1.49 eV, whereas **7-235** has both HOMO/LUMO levels downshifted. Solar cells based on PM6/**7-234** yielded a high *V*_OC_ of 0.90 V with a FF of 72% and a PCE of 13.9%. The combination of PTB7-Th/**7-235** gave a rather low *V*_OC_ of 0.70 V with an efficiency of 7.90%.^274,275^ Molecules **7-236**–**7-244** have INCN-2Cl acceptor units but different substituents on the central benzene ring of the donor unit. The NFAs **7-236**–**7-238** possess ethylhexylthienyl (**7-236**, HBDT-4Cl)^276^ or the fluorinated and chlorinated thienyl analogue **7-237** (FBDT-4Cl)^276^ and **7-238** (ClBDT-4Cl).^276^ All three molecules have a similar optical band gap of about 1.44 eV and similar HOMO/LUMO energies. Pristine films of the three acceptors were investigated using GIWAXS, showing that **7-236** has an amorphous nature and disordered packing. In the halogenated structures, the packing patterns are changed from amorphous to face-on orientation. Solar cells from blends with PM6 exhibit better PV performances for the halogenated and higher crystalline compounds **7-237** and **7-238** than the parent compound **7-236**, reaching PCE values up to 12.4% (for **7-237**).

**7-239** (NCBDT-4Cl)^277^ contains octyl side chains on the central benzene ring, and solar cells with PBDB-T-SF as the donor polymer achieved a remarkable PCE of 14.1% exhibiting a *V*_OC_ of 0.85 V.^277^**7-241** (NCBDT-4Cl)^278^ resembles **7-239** but differs in the alkyl group in the side chains, being octylphenyl instead of hexylphenyl. The energy levels and optical band gaps of both acceptors are similar. Solar cells of PM6/**7-241** give PCE values up to 12.0%. A study of Kan et al. compares the acceptors **7-240**, **7-242**, and **7-243** with different substituents on the central ring. Whereas **7-240** (DPBDT-4Cl)^279^ comprises two hexylphenyl side chains, **7-242** (POBDT-4Cl)^279^ and **7-243** (COBDT-4Cl)^279^ bear two different side chains, either a hexylphenyl and an octyloxy group in **7-242** or an octyl and octyloxy group in **7-243**. The introduction of the alkyloxy groups together with the more flexible side chains improved the molecular packing, the light absorption, as well as the electron mobility. Consequently, solar cells with PM6 show higher *J*_SC_ and FF values and thus higher PCEs for the octyloxy derivatives. Following, **7-243** possesses a PCE of 13.5%, which is the highest among these three acceptors.^279^**7-244** (TOBDT)^280^ contains one octyloxy and one ethylhexylthienyl side chain on the middle benzene ring, which has no impact on the energy levels or the optical band gap compared to the acceptors **7-242** and **7-243**. With PM6 as the polymer donor, a similar *V*_OC_ was reached as that for **7-242** and **7-243** accompanied by a lower *J*_SC_, similar FF, and also a lower PCE of 11.3%.^280^ Yan et al. compared the hexylphenyl-substituted compounds **7-245** (BPIC-2Cl)^189^ and **7-240** (BPIC-4Cl),^189^ having either INCN-Cl or INCN-2Cl units with their parent compound **7-122** with INCN. The frontier orbital energies are lowered and the band gap narrowed with increasing chlorine substitution. The NFA **7-245** shows the highest electron mobilities and in combination with PBDB-T also the highest performance in solar cells (PCE: 12.2%) for these three acceptors, followed by **7-122** (10.7%) and **7-240** (8.01%).^189^

Lai et al. investigated different orientations of the thiophene rings in the backbone with the same end groups and side chains, leading to structures **7-246** (BDT_c_IC-γCl)^281^ and **7-247** (BDT_t_IC-γCl).^281^ Here, **7-247** has heightened energy levels compared to **7-246** and they show a quite different absorption behavior in solution as well as in thin films. For **7-247**, a broader absorption but also a lower extinction coefficient is obtained. In solar cells with PM6, **7-246** shows a 10 times higher efficiency of 7.61% compared to **7-247**, revealing a PCE of 0.71%, which is accompanied by low EQE values of PM6/**7-247**. Even though the thiophene orientation has little impact on the film morphology determined by AFM and TEM images, GIWAXS measurements indicate a more regular and ordered packing for **7-246** than **7-247**, which is also accompanied by significantly higher electron mobilities of PM6/**7-246** films.^281^

The NFAs **7-248**–**7-255** bear CPTCN end groups and show additional variations of the substituents on the central benzene ring of the donor unit. The parent compound **7-248** (ITIC5)^188^ without substituents reveals HOMO and LUMO energy levels of −5.48 and −3.95 eV, respectively, with an optical band gap of 1.53 eV. Solar cells with J71 as a donor gave a *V*_OC_ of 0.90 eV, a FF of 70%, and an efficiency of 11.0%.^188^ Gao et al. investigated the influence of additional methyl substituents in the CPTCN unit in benzodi(cyclopentadithiophene) cores with ethylhexylthienyl substituents on the central ring by comparing the parent structure with CPTCN (**7-249**, BTTIC-0M),^282^ CPTCN-Me (**7-251**, BTTIC-2M),^282^ and CPTCN-2Me analogues (**7-252**, BTTIC-4M).^282^ The addition of methyl groups increases the energy levels and optical band gaps. Solar cells of these compounds with PBDB-T yield efficiencies of 11.9% (**7-249**), 13.2% (**7-251**), and 9.6% (**7-252**), respectively. GIWAXS data measured from the neat acceptor films show that all three molecules have a preferred face-on orientation, but **7-251** has a higher coherence length and π–π stacking, which is beneficial for charge transport and may explain why the efficiency is higher.^282^ Zhang et al. also investigated **7-249** and compared it with a similar structure **7-250** (BDTSF-IC),^283^ which has additional fluorines and sulfur atoms in the central side chains. Compared to **7-249**, **7-250** comprises a larger band gap and PM6/**7-250**-based solar cells yield PCE values up to 13.1%.^283^ Structures **7-253** (BTTIC-TT),^284^**7-254** (BTTIC-Ph),^284^ and **7-255** (BTOIC)^285^ are similar to **7-251** (CPTCN-Me) but bear ethylhexylthienothienyl, hexylphenyl, and ethylhexyloxy side chains, respectively, on the central ring. Whereas **7-253** and **7-254** exhibit similar optical band gaps as **7-251** with values of approx. 1.47 eV, the alkoxy chains in **7-255** narrow the band gap to 1.39 eV. All acceptors were implemented in solar cells with PBDB-T. Compared to **7-251**, solar cells with **7-253** and **7-254** exhibit slightly higher *V*_OC_ values, but while solar cells of **7-253** even reach slightly higher PCEs of 13.4%, those of **7-254** show only a maximum PCE of 9.14% due to lower *J*_SC_ and FF values. The devices with **7-255** showed expectedly lower *V*_OC_ values but still reached a PCE of 11.0%.

The extension of the aromatic ring system of the acceptor end groups either with a thiophene or phenyl ring leads to structures **7-256** (BDCPDT-TTC)^273^ and **7-257** (BDCPDT-BC),^134^ both without side chains on the central benzene ring. **7-256** has a higher lying LUMO as well as a broader band gap, whereas the HOMO energy levels are similar for **7-256** and **7-257**. Besides, PV parameters (with PBDB-T) are similar with efficiencies of 10.3 and 10.8% for **7-256** and **7-257**, respectively.^134,273^ Finally, the NFA **7-258** (BTOIPC)^190^ has ethylhexyloxy groups on the central group but replaces the INCN groups with ((2,3-dicyano-9*H*-indeno[1,2-*b*]pyrazine-9-ylidene)methyl) acceptor units. Compared to **7-255**, the optical band gap is increased to 1.45 eV, whereas solar cells with the same donor polymer show lower PCEs of 9.31%.^190^

The NFAs **7-259**–**7-270** comprise two *N*-alkyl pyrroles instead of the cyclopentadiene rings in the central donor unit and an alkyloxy-substituted central benzene ring. They differ in their peripheral accepting units (INCN-based and CPTCN-based accepting units) as well as in their *N*-alkyl chains. Within this group of NFAs, some structures have shown promising high efficiencies in solar cells with PCEs up to 16%.

Ma et al. introduced molecules **7-259**–**7-261**, which have INCN-2F groups but differ in the alkyl groups on the central benzene and the pyrrole unit, i.e., ethylhexyloxy and ethylhexyl chains (**7-259**, M2),^286^ butyloctyloxy and butyloctyl chains (**7-260**, M36),^286^ and decyltetradecyloxy and decyltetradecyl chains (**7-261**, M38).^286^ They all have similar HOMO/LUMO energy levels; the highest optical band gap is observed for **7-261** with 1.47 eV due to a blue-shifted absorption. The PV parameters of solar cells using the acceptors **7-259**–**7-261** blended with PM6 deviate highly from each other. The reason lies in the molecular packing of the donor/acceptor blend films. GIWAXS data revealed the smallest π–π stacking distance for **7-260**, resulting in the highest electron mobility and remarkable efficiencies up to 16.0%. In comparison, **7-259** and **7-261** yielded 11.2 and 8.89%, respectively.^286^ Molecule **7-262** (M4) comprises mixed side chains, *N*-ethylhexyl groups (as in **7-259**), and butyloctyloxy substituents on the central benzene ring (as in **7-260**).^287^ This does not affect the HOMO/LUMO energy levels, nor the optical band gap. In combination with PM6, solar cells yielded high PCE values of 14.8%.^287^ NFA **7-263** (BDTN-BF)^288^ has butyloctyl chains on the nitrogen atoms and octyloxy chains on the central benzene ring. Solar cells of **7-263** in blend with PM6 reach efficiencies up to 11.5% with *V*_OC_s up to 0.93 V.^288^ By swapping the butyloctyl and ethylhexyl chains in **7-262**, structure **7-264** (BDTBO-4F)^289^ is obtained; a further change to the INCN-2Cl groups leads to **7-265** (BDTBO-4Cl).^289^ Both acceptors share similar energy levels and optical band gaps, whereas the PV parameters (except the *J*_SC_) are higher for solar cells with **7-264**, achieving an efficiency of 14.8% compared to the PCE of **7-265**-based solar cells of 13.9%, due to better charge transport properties of PM6/**7-264** blends.^289^ Ma et al. investigated structure **7-264** and compared it with the analogue **7-266** (M8),^290^ in which the inner thiophene rings are exchanged with furans. The oxygen leads to a heightened HOMO energy level, thus reducing the band gap to 1.28 eV. Solar cells of **7-264**/PM6 showed much better parameters with a high PCE of 15.2% compared to 4.21% for **7-266**.^290^ Zeng et al. prepared three acceptors with central ethylhexyloxy substituents and INCN-F groups, differing in their *N*-alkyl chains, i.e., ethylhexyl **7-267** (SNBDT1-F),^291^ ethylheptyl **7-268** (SNBDT2-F),^291^ and ethyloctyl **7-269** (SNBDT3-F).^291^ All three acceptors have the same optical band gap and similar HOMO/LUMO energy levels. With increasing side chain length, the PV efficiencies of solar cells with PBDB-T are decreasing from 12.7% (**7-267**) to 6.57% (**7-268**) and then further to 5.87% (**7-269**). The reason for the low PCEs lies in insufficient exciton dissociation of **7-268** and **7-269** due to edge-on orientation in blend films with the polymer PBDB-T.^291^

**7-270** (BDTN-Th)^288^ resembles **7-263** with the difference of having CPTCN end groups instead of INCN-2F units. They are similar in their absorption behavior and both were evaluated in solar cells with PM6, which show a *V*_OC_ of over 0.90 V in both cases; however, **7-270** only reached PCE values of 3.53% (compared to 11.5% for **7-263**) This may be caused by the higher electron mobilities and more balanced electron/hole mobility values of **7-263**.^288^

**Figure 13:**
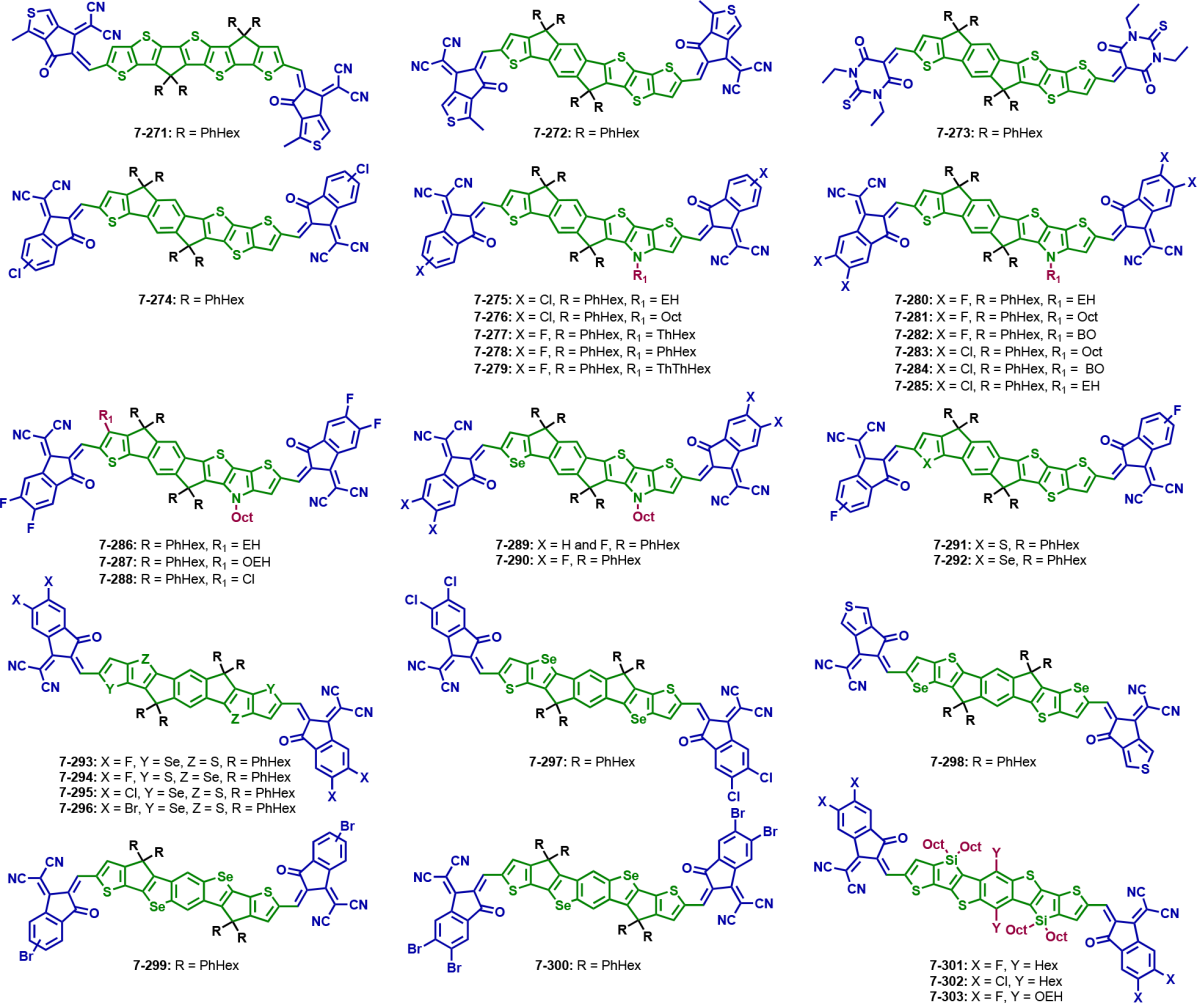
Structures of non-fullerene acceptors with asymmetric backbones and/or additional heteroatoms implemented in the backbone.

Other strategies of designing NFAs are the use of asymmetric donor units and/or the insertion of heteroatoms such as Se or Si in the core unit (**7-271**–**7-303**), as shown in Figure 13. Characteristic properties of these NFAs are summarized in Table 9. The asymmetric molecule **7-271** (T7Me)^292^ contains a backbone consisting of five thiophenes (T) and two cyclopentadienes (Cp) in the following order: T-T-Cp-T-T-Cp-T with CPTCN-Me acceptor units. It has a small optical band gap of 1.36 eV, and the HOMO and LUMO energy levels are −5.45 and −3.93 eV, respectively. Solar cells with PM6 as a donor revealed a PCE of 8.96%.

**Table 9:** Optical, Electrical, and Photovoltaic Properties of Non-Fullerene Acceptors **7-271**–**7-303**

NFA	original name	HOMO^a^ (eV)	LUMO^a^ (eV)	*E*_g_^opt^ (eV)	donor	D:A ratio	*V*_OC_ (V)	*J*_SC_(mA cm^–2^)	FF (%)	PCE (%)	μ_e_^b^(cm^2^ V^–1^ s^–1^)	ref.
**7-271**	T7Me	–5.45	–3.93	1.36	PM6	1:1	0.89	17.1	59	8.96	-/8.4 × 10^–5^	(292)
**7-272**	MeIC1	–5.59	–3.89	1.54	PBDB-T	1:1	0.93	18.3	74	12.6	2.4 × 10^–3^/5.1 × 10^–4^	(212)
**7-273**	TPTTT-T	–5.72	–3.73	1.81	PBT1-C	1:1	1.11	11.1	61	7.42	2.6 × 10^–4^/2.2 × 10^–4^	(293)
**7-274**	a-IT	–5.65	–3.99	1.54	PM6	1:1	0.91	16.6	76	11.5	4.3 × 10^–4^/-	(294)
**7-275**	TPIC-2Cl	–5.36	–3.92	1.45	PM7	1:1	0.94	21.4	72	14.5	7.5 × 10^–4^/4.5 × 10^–4^	(201)
**7-276**	N7IT	–5.47	–3.93	1.42	PM6	1:1	0.93	21.0	71	13.8	7.2 × 10^–4^/7.0 × 10^–4^	(294)
**7-277**	IPT2F-Th	–5.58	–4.03	1.47	PBDB-T	1:1	0.86	20.4	71	12.5	-/5.1 × 10^–4^	(295)
**7-278**	IPT2F-Ph	–5.57	–4.00	1.46	PBDB-T	1:0.8	0.86	21.2	72	13.1	-/5.7 × 10^–4^	(295)
**7-279**	IPT2F-TT	–5.60	–4.00	1.45	PBDB-T	1:1	0.84	22.2	75	14.0	-/6.1 × 10^–4^	(295)
**7-280**	IDTP-4F	–5.54	–3.98		PM7	1:1	0.90	22.5	75	15.2	7.3 × 10^–4^/4.1 × 10^–4^	(296)
**7-281**	IPT-4F	–5.57	–4.08	1.42	PM6	1:1	0.91	22.1	74	15.0	5.8 × 10^–4^/1.3 × 10^–4^	(297)
	IPT-4F	–5.56	–4.05	1.41	PM6	1:1	0.88	22.2	75	14.6		(298)
**7-282**	IPTBO-4F	–5.57	–4.07	1.41	PM6	1:1	0.92	22.1	73	14.7	3.7 × 10^–4^/1.0 × 10^–4^	(297)
**7-283**	IPT-4Cl	–5.58	–4.11	1.39	PM6	1:1	0.88	23.2	70	14.4	7.0 × 10^–4^/1.1 × 10^–4^	(297)
**7-284**	IPTBO-4Cl	–5.64	–4.08	1.39	PM6	1:1	0.89	23.2	73	15.0	5.2 × 10^–4^/1.2 × 10^–4^	(297)
**7-285**	TPIC-4Cl	–5.35	–3.97	1.40	PM7	1:1	0.88	23.0	76	15.3	8.8 × 10^–4^/5.1 × 10^–4^	(201)
**7-286**	IN-4F	–5.59	–3.94	1.45	PM6	1:1	0.92	19.5	70	12.5		(298)
**7-287**	INO-4F	–5.64	–3.93	1.46	PM6	1:1	0.93	20.5	72	13.7		(298)
**7-288**	IPCl-4F	–5.60	–4.08	1.39	PM6	1:1	0.83	21.2	61	10.8		(298)
**7-289**	DTPPSe-2F	–5.52	–4.05	1.40	PBDB-T	1.25:1	0.84	22.2	74	13.8	-/5.1 × 10^–4^	(202)
**7-290**	DTPPSe-4F	–5.53	–4.10	1.39	PBDB-T	1.25:1	0.78	21.2	73	12.0	-/5.0 × 10^–4^	(202)
**7-291**	TPTTT-2F	–5.69	–4.01	1.56	PBT1-C	1:1	0.92	17.6	75	12.0	4.8 × 10^–4^/2.2 × 10^–4^	(299)
**7-292**	SePTTT-2F	–5.66	–3.97	1.50	PBT1-C	1:1.1	0.90	18.0	76	12.2	1.2 × 10^–3^/4.4 × 10^–4^	(300)
**7-293**	SRID-4F	–5.52	–3.90	1.44	PBDB-T	1:1	0.85	20.2	75	13.1	-/2.2 × 10^–4^	(126)
**7-294**	TRID-4F	–5.52	–3.90	1.48	PBDB-T	1:1.5	0.89	18.5	75	12.3	-/2.7 × 10^–4^	(126)
**7-295**	TSeIC-4Cl	–5.75	–3.99	1.44	PM6	1:1	0.74	20.9	72	11.1	7.4 × 10^–4^/3.8 × 10^–4^	(301)
**7-296**	TSeIC-4Br	–5.69	–3.97	1.43	PM6	1:1	0.77	21.3	72	11.9	7.9 × 10^–4^/4.2 × 10^–4^	(301)
**7-297**	SeTIC4Cl	–5.65	–4.08	1.44	PM6	1:1	0.78	22.9	75	13.3	-/3.0 × 10^–3^	(184)
**7-298**	TSeTIC	–5.65	–3.91	1.53	PM6	1:1	0.93	19.4	76	13.7	4.1 × 10^–4^/3.1 × 10^–4^	(302)
**7-299**	BDSeIC2Br	–5.63	–3.99	1.41	PM6	1:1	0.89	20.3	69	12.5	4.5 × 10^–4^/3.8 × 10^–4^	(185)
**7-300**	BDSeIC4Br	–5.65	–4.02	1.39	PM6	1:1	0.85	16.4	69	9.60	4.2 × 10^–4^/2.2 × 10^–4^	(185)
**7-301**	ArSiID-F	–5.42	–3.90		PBDB-T	1:1	0.84	16.8	65	9.40	-/3.1 × 10^–4^	(193)
**7-302**	ArSiID-Cl	–5.45	–4.01		PBDB-T	1:1	0.79	16.4	60	7.90	-/1.1 × 10^–4^	(193)
**7-303**	NFDTSB	–5.56	–3.85	1.40	PTB7-Th	1:1	0.75	20.1	63	9.60	-/9.3 × 10^–5^	(303)

a Obtained from the oxidation/reduction potential of the CV measurement if not otherwise stated.

b Determined via the SCLC technique from the neat acceptor/donor:acceptor blend films if not otherwise stated.

NFAs **7-272**–**7-274** have an asymmetric backbone with six 5-membered rings—4 T and 2 Cp—and one 6-membered ring—a benzene (B)—in the order T-Cp-B-Cp-T-T-T and different acceptor end groups. The molecule **7-272** (MeIC_1_)^212^ shares the same end groups with **7-271**; **7-273** (TPTTT-T)^293^ uses diethyl TBA as an acceptor unit, and **7-274** (a-IT)^294^ possesses INCN-Cl groups. **7-272** and **7-274** have the same optical band gap of 1.54 eV, whereas the TBA in **7-273** increases the band gap to 1.81 eV. The three acceptors were blended with different polymer donors in solar cells, which makes them difficult to compare. However, PBT1-C/**7-273** achieved the highest *V*_OC_ of these three combinations with 1.11 V but also the lowest PCE with 7.42%. PBDB-T/**7-275** and PM6/**7-274** reached *V*_OC_s of over 0.90 V with efficiencies of 12.6 and 11.5%, respectively.^212,293,294^

NFAs **7-275**–**7-288** have similar backbones as **7-272**–**7-274**, but an *N*-alkylated pyrrole (P) substitutes a thiophene in the central donor unit, leading to the following motif: T-Cp-B-Cp-T-P-T. They are different in their accepting units, the *N*-alkyl groups, as well as the substituents on an outer thiophene (**7-286**–**7-288**). **7-275** (TPIC-2Cl)^201^ and **7-276** (N7IT)^294^ share INCN-Cl groups with **7-274**, but the pyrrole ring leads to higher HOMO and lower LUMO energy levels, which reduces the band gaps of **7-275** and **7-276**. Solar cells of PM7/**7-275** and PM6/**7-276** give *V*_OC_s of over 0.90 V, *J*_SC_s of over 21 mA cm^–2^, FFs of over 70%, and efficiencies up to 14.5 and 13.8%, respectively.^201,294^ Cao et al. synthesized a series of acceptors with the same backbone, INCN-F acceptor units, and different substituents on the nitrogen atom (**7-277**–**7-279**).^295^ The side chain has nearly no impact on the energy levels and the optical band gap of the acceptors. Solar cells were built in combination with PBDB-T, and the PCE increases from **7-277** (IPT2F-Th, 12.5%)^295^ over **7-278** (IPT2F-Ph, 13.1%)^295^ to **7-279** (IPT2F-TT, 14.0%)^295^ simultaneously with increasing FF and electron mobility values.^295^**7-280**–**7-282** have INCN-2F units and differ in their *N*-alkyl chains. **7-280** (IDTP-4F)^296^ has HOMO/LUMO energy levels of −5.54 eV/–3.98 eV, respectively. Solar cells with PM7 yielded high efficiencies up to 15.2%.^296^ The two NFAs, **7-281** (IPT-4F)^297^ and **7-282** (IPTBO-4F),^297^ have similar HOMO/LUMO energy levels of −5.57 eV/–4.08 eV and −5.57 eV/–4.07 eV and optical band gaps of 1.42 and 1.41 eV, respectively. Acceptor **7-281** contains an octyl side chain and **7-282** a butyloctyl group. Solar cells with PM6 revealed similar *V*_OC_ and *J*_SC_ and efficiencies of 14.7 and 15.0% for **7-282** and **7-281**, respectively. Devices of the analogous chlorinated NFAs, **7-283** (IPT-4Cl)^297^ and **7-284** (IPTBO-4Cl),^297^ have slightly lower band gaps of 1.39 eV. Solar cells of both acceptors, **7-283** and **7-284**, with PM6 gave PCEs up to 15.0% (**7-284**) and 14.4% (**7-283**).^297^ The molecule **7-285** (TPIC-4Cl)^201^ resembles **7-275**, with the difference of INCN-2Cl instead of INCN-Cl units. The monochlorinated **7-275** exhibits a higher optical band gap (1.45 eV) than **7-285** (1.40 eV) and the other dichlorinated compounds **7-283** and **7-284**. Solar cells with **7-275** and **7-285** and PM7 achieved remarkable efficiencies of 14.5 and 15.3%, respectively. As expected from the band gap, the solar cells based on **7-285** show a lower *V*_OC_ but higher *J*_SC_ of 23.0 mA cm^–2^ and a better FF. The results align with GIWAXS data, as the higher crystallinity of the blend film PM7/**7-285** indicates a better charge transport and thus better performance in OSCs and higher electron mobility.^201^ Zhang et al. studied the difference in the optical and electrochemical behavior when substituents are introduced on an outer thiophene ring of **7-281**. This leads to molecules **7-286**–**7-288**, which contain an additional ethylhexyl group (**7-286**, IN-4F),^298^ ethylhexyloxy group (**7-287**, INO-4F),^298^ or chlorine atom (**7-288**, IPCl-4F).^298^ The alkyl and alkoxy chains are shifting the HOMO down and the LUMO level upward, thus widening the band gap, while the chlorine atom lowers both energy levels, giving a slightly narrower band gap than **7-281**. Photovoltaic devices of **7-286**–**7-288** combined with PM6 lead to lower *J*_SC_, FF, and PCE values than **7-281** but enhanced *V*_OC_s. Here, the highest efficiency was obtained with PM6/**7-287** (13.7%) followed by PM6/**7-286** (12.5%) and PM6/**7-288** (10.8%).^298^

In the structures **7-289** (DTPPSe-2F)^202^ and **7-290** (DTPPSe-4F),^202^ one thiophene is replaced with a selenophene (T(Se)) ring, leading to the structure T(Se)-Cp-B-Cp-T-P-T. **7-290**—comprising INCN-2F units—is thereby the Se analogue to **7-281**, while **7-289** contains INCN-F units. This does not largely affect the positions of the energy levels, but the optical band gaps are slightly lower. The PCEs of PBDB-T/**7-289**- and PBDB-T/**7-290**-based solar cells are 13.8 and 12.0%, respectively; however, the PV parameters are difficult to compare to devices based on PM6/**7-281** due to the different donors.^202^ The thiophene-based asymmetric structure **7-291** (TPTTT-2F)^299^ (T-Cp-B-Cp-T-T-T) can be compared with the selenium counterpart **7-292** (SePTTT-2F)^300^ (T(Se)-Cp-B-Cp-T-T-T). The selenium compound **7-292** provides slightly higher energy levels and a lower band gap (1.50 eV instead of 1.56 eV for **7-291**). However, the PV parameters of solar cells with these molecules and PBT1-C as a donor are very similar. Both exhibit a PCE over 12%, a high *V*_OC_ over 0.90 V, and FFs over 74%.^299,300^

Lin et al. compared the impact of the selenium position using structure **7-293** (SRID-4F)^126^ with T(Se)-T-Cp-B-Cp-T-T(Se) and structure **7-294** (TRID-4F)^126^ with T-T(Se)-Cp-B-Cp-T(Se)-T, both with INCN-2F acceptor units. The position of the selenophene does not affect the energy levels and only slightly the band gap in the case of **7-293** and **7-294**. Combined with PBDB-T, the solar cells showed an efficiency of 13.1 and 12.3% for **7-293** and **7-294**, respectively. These results are consistent with GIWAXS data, where a closer π–π stacking was observed for **7-293**, leading to a better charge transport behavior.^126^ Molecules **7-295** (TSeIC-4Cl)^301^ and **7-296** (TSeIC-4Br)^301^ have a similar backbone to **7-294** but have INCN-2Cl and dibrominated INCN (INCN-2Br) acceptor units, respectively. Both the chlorinated and brominated species show lower HOMO/LUMO energy levels than the fluorinated counterpart, leading to smaller optical band gaps. The characteristic solar cell parameters (except for the *J*_SC_s) of blends with PM6 are lower for **7-295** and **7-296** than for **7-294**; however, still PCEs of over 11% could be reached.^301^

The NFA **7-297** (SeTIC4Cl)^184^ is the INCN-Cl counterpart to **7-294**. The chlorine atoms cause a further downshift of the energy levels and a slightly decreased optical band gap. Devices with PM6 as a donor give a high PCE of 13.3%, due to a high FF and a *J*_SC_ of 22.9 mA cm^–2^.^184^ Molecule **7-298** (TSeTIC)^302^ comprises the same backbone as **7-293**, but CPTCN end groups were used. Comparing them, **7-298** has a downshifted HOMO level and therefore a larger band gap than **7-293**. Solar cells were assembled with PM6 as a donor and give a PCE of 13.7%.^302^**7-299** (BDSeIC2Br)^185^ and **7-300** (BDSeIC4Br)^185^ feature the same backbone structure with two selenophene rings in the donor unit with a T-Cp-T(Se)-B-T(Se)-Cp-T core but either with INCN-Br (**7-299**) or INCN-2Br (**7-300**) acceptor units. The energy levels and the optical band gap resemble each other with a slightly lower band gap of 1.39 eV for the dibrominated molecule. However, the PV parameters of solar cells with PM6 as a donor show that the monobrominated derivative **7-299** yields higher PCE values (12.5%) than **7-300** (9.60%). Besides an expected higher *V*_OC_, also, higher *J*_SC_s and FFs are found. This is also reflected in the enhanced electron mobility in blend film and the more balanced electron/hole mobility.

Silicon can be used to replace carbon in conjugated structures. In **7-301**–**7-303**, two silole (Si) rings substituted with two octyl chains were introduced into the donor unit, leading to the structure T-Si-T-B-T-Si-T. The central benzyl unit contains additional alkyl side chains. Wang et al. studied the difference of INCN-2F (**7-301**, ArSiID-F)^193^ and INCN-2Cl acceptor units (**7-302**, ArSiID-Cl)^193^ in this structure with hexyl chains on the central benzene ring. Both structures possess similar HOMO levels, but the LUMO is upshifted for the fluorinated compound **7-301**. Solar cells with PBDB-T are showing maximum PCEs of 9.40% in the case of **7-301** and 7.90% for **7-302**, caused especially by lower *V*_OC_ and FF values.^193^ The exchange of the octyl chain on the central benzene ring in **7-301** with ethylhexyloxy side chains leads to structure **7-303** (NFDTSB).^303^ In comparison to **7-301**, the HOMO energy level is downshifted and the LUMO upshifted, giving an optical band gap of 1.40 eV. Solar cells with PTB7-Th/**7-303** gave a PCE of 9.6%.^193,303^

**Figure 14:**
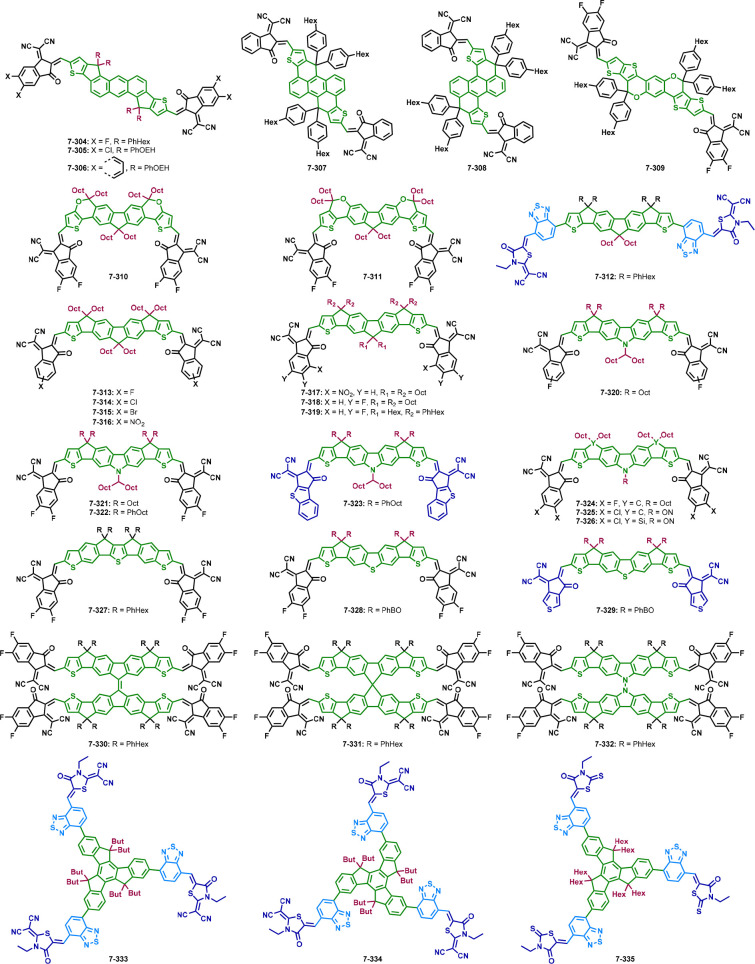
Structures of acceptors with anthracene, pyran-bridged, fluorene, carbazole, dicyclopentathiophene, and dibenzothiophene-based donor units; structures with two seven-ring core units and truxene cores.

Figure 14 shows various other NFA motifs of the last years and Table 10 the corresponding solar cell data. **7-304**–**7-306** exhibit a “kinked” donor unit based on a central anthracene unit with two [*b*]-annulated cyclopenta[*b*]thiophene units on each side which lead to a double-kink structure in the arrangement of the seven rings. Acceptor **7-304** (ANT-4F)^304^ contains INCN-2F groups and hexylphenyl side chains, whereas **7-305** (AT-4Cl)^305^ has INCN-2Cl groups and (2-ethylhexyl)oxyphenyl side chains. **7-304** shows a broader band gap (1.68 eV) and lower LUMO, but their HOMO energies are similar. Solar cells with PM6 give PCEs above 13% with both compounds. Exchanging the dichlorobenzene rings in the INCN units of **7-305** with naphthalene results in structure **7-306** (AT-NC),^305^ which shows higher HOMO/LUMO energy levels as well as a slightly wider band gap. Solar cells (with PBDB-T) revealed, however, a lower performance. More kinked structures are represented by the structural isomers **7-307** (DTA-IC-S) and **7-308** (DTA-IC-M), with hexylphenyl side chains and INCN end groups.^306^**7-307** has a large band gap of 1.67 eV accompanied by a much lower HOMO and higher LUMO energy level. Solar cells with the PBDB-T yielded a PCE of 4.20% (**7-308**) and 6.09% (**7-307**).^306^ The pyrane-bridged acceptor **7-309** (CO_i_7DFIC)^307^ resembles in its double-kink structure the NFA **7-304**, having INCN-2F units too. **7-309** shows HOMO/LUMO energy levels of −5.78 eV/–4.04 eV, respectively, and an optical band gap of 1.55 eV. Solar cells were built with PTB7-Th, giving moderate results with an efficiency of 8.70%.^307^

**Table 10:** Optical, Electrical, and Photovoltaic Properties of Non-Fullerene Acceptors **7-304**–**7-335**

NFA	original name	HOMO^a^ (eV)	LUMO^a^ (eV)	*E*_g_^opt^ (eV)	donor	D:A ratio	*V*_OC_ (V)	*J*_SC_(mA cm^–2^)	FF (%)	PCE (%)	μ_e_^d^(cm^2^ V^–1^ s^–1^)	ref.
**7-304**	ANT-4F	–5.69	–4.01^b^	1.68	PM6		0.93	19.0	74	13.1	-/1.4 × 10^–4^	(304)
**7-305**	AT-4Cl	–5.71	–3.89	1.60	PM6	1:1	0.90	19.5	76	13.3	-/1.4 × 10^–4^	(305)
**7-306**	AT-NC	–5.44	–3.80	1.62	PBDB-T	1:0.8	0.92	17.1	69	10.9	-/6.5 × 10^–5^	(305)
**7-307**	DTA-IC-S	–5.73	–3.83	1.67	PBDB-T	1:1.5	0.94	12.3	53	6.09	3.9 × 10^–6^/3.0 × 10^–5^	(306)
**7-308**	DTA-IC-M	–5.53	–4.05	1.35	PBDB-T	1:1	0.69	13.0	47	4.20	1.1 × 10^–6^/9.4 × 10^–5^	(306)
**7-309**	COi7DFIC	–5.78^c^	–4.04^c^	1.55	PTB7-Th	1:1.5	0.67	18.4	70	8.70		(307)
**7-310**	FCO-2F	–5.37	–3.78		PM6	1:1	0.88	20.9	72	13.4	-/1.4 × 10^–4^	(308)
**7-311**	FO-2F	–5.72	–3.94	1.47	PM6	1:1	0.88	22.3	77	15.1	-/2.4 × 10^–4^	(309)
**7-312**	DTFBR	–5.54	–3.68	1.74	P3HT	1:1	0.71	8.15	62	3.68	2.2 × 10^–4^/-	(310)
**7-313**	F-F	–5.45	–3.77	1.59	PBDB-T	1:1.2	0.88	17.4	71	10.9	-/1.0 × 10^–4^	(200)
**7-314**	F-Cl	–5.46	–3.75	1.58	PBDB-T	1:1	0.87	17.6	75	11.5	-/1.2 × 10^–4^	(200)
**7-315**	F-Br	–5.47	–3.78	1.56	PBDB-T	1:1	0.87	18.2	76	12.1	-/1.5 × 10^–4^	(200)
**7-316**	F-N1	–5.62	–4.16	1.49	PM6	1:1	0.74	20.1	72	10.7	-/1.2 × 10^–4^	(311)
**7-317**	F-N2	–5.61	–4.07	1.53	PM6	1:1	0.94	18.3	69	11.9	-/1.4 × 10^–4^	(311)
**7-318**	F-2F	–5.78	–3.89	1.60	PM6	1:1	0.94	18.5	74	12.9	-/1.5 × 10^–4^	(309)
**7-319**	FXIC-1	–5.78	–3.96	1.67	PTB7-Th	1:1.5	0.79	13.4	67	7.13	5.5 × 10^–4^/3.5 × 10^–4^	(312)
**7-320**	C8-DTC	–5.60	–3.87		PM6	1:1.2	0.95	16.9	75	12.1		(313)
**7-321**	DTC(4R)-4FIC	–5.79	–3.99		J71	1:1.3	0.82	18.9	70	10.9		(195)
**7-322**	DTC(4Ph)-4FIC	–5.80	–3.94		PM6	1:1	0.95	18.3	76	13.2		(196)
**7-323**	DTCC-BC	–5.46	–3.81	1.67	PBDB-T	1:1.5	0.98	17.2	64	10.7	-/1.4 × 10^–5^	(134)
**7-324**	DTCCIC-4F	–5.84	–3.62	1.53	PFBDB-T	1:1.5	0.85	22.1	67	12.6	-/4.3 × 10^–6^	(197)
**7-325**	DTCC-4Cl	–5.71	–4.16	1.50	T1	1:1.4	0.94	20.0	76	14.4	-/1.0 × 10^–5^	(314)
**7-326**	DTSiC-4Cl	–5.73	–4.15	1.55	T1	1:1.4	1.00	19.6	74	14.5	-/1.4 × 10^–5^	(314)
**7-327**	FTBT	–5.67	–3.95^c^	1.59	PM6	1:1	0.96	7.55	48	3.47	3.8 × 10^–5^/3.3 × 10^–5^	(315)
**7-328**	DBTIC-2F	–5.94	–4.08	1.62	PBDB-T	1:1	0.80	15.2	56	6.81	-/1.3 × 10^–6^	(199)
**7-329**	DBTTC	–5.92	–4.03	1.64	PBDB-T	1:1	0.97	17.3	67	11.3	-/3.6 × 10^–5^	(199)
**7-330**	FXIC-2	–5.79	–4.00	1.67	PTB7-Th	1:1.5	0.72	10.7	68	5.22	1.8 × 10^–4^/4.0 × 10^–5^	(312)
**7-331**	FXIC-3	–5.79	–3.98	1.68	PTB7-Th	1:1.5	0.76	12.0	67	6.12	1.8 × 10^–4^/1.2 × 10^–4^	(312)
**7-332**	99CZ-8F	–5.79	–3.88	1.60	PM6	1:1	0.94	11.2	63	6.60		(316)
**7-333**	meta-TrBRCN	–5.95	–3.72	2.10	PTB7-Th	0.8:1	0.94	16.8	65	10.2	-/2.0 × 10^–4^	(317)
**7-334**	para-TrBRCN	–5.98	–3.71	2.19	PTB7-Th	0.8:1	0.95	13.8	64	8.29	-/1.2 × 10^–4^	(317)
**7-335**	Tr(Hex)_6_-3BR	–5.79	–3.74	2.15	PTB7-Th	1:1	1.02	5.92	33	2.10	-/1.5 × 10^–5^	(204)

a Obtained from the oxidation/reduction potential of the CV measurement if not otherwise stated.

b HOMO/LUMO energy levels obtained from the LUMO/HOMO levels determined by CV and the optical band gap.

c Other method or method not defined.

d Determined via the SCLC technique from the neat acceptor/donor:acceptor blend films if not otherwise stated.

The donor unit of structures **7-310**–**7-319** comprises a central fluorene and two thiophene units linked either by a pyrane ring (**7-310**, FCO-2F,^308^ and **7-311**, FO-2F^309^) or a cyclopentadiene unit (**7-312**–**7-319**). Compared to the above-described anthracene-based NFA **7-304**, having the same INCN-2F units, **7-310** has much higher HOMO/LUMO energy levels, whereas **7-311** has slightly lower HOMO and higher LUMO levels, accompanied by a narrower optical band gap. Solar cells with PM6/**7-310** absorber layers reached similar PCEs (13.4%) as PM6/**7-304**-based devices.^308^ PM6/**7-311** blends reveal a similar *V*_OC_ as PM6/**7-310** but higher *J*_SC_ and FF values with an enhanced efficiency of 15.1%.^309^**7-312** comprises a BT π-spacer and RCN end groups. The material has a large optical band gap of 1.74 eV, and devices with P3HT as a polymer donor give an efficiency of 3.68%.^310^ In **7-313**–**7-315**, the central donor unit is directly linked to a INCN-X unit. Wang et al. investigated the fluorinated (**7-313**, F-F),^200^ chlorinated (**7-314**, F-Cl),^200^ and brominated (**7-315**, F-Br)^200^ species, leading to the result that **7-313** possesses the lowest energy levels of these three compounds, whereas **7-314** and **7-315** have similar HOMO/LUMO energy levels. The optical band gap is alike for all of them. Interestingly, solar cells in combination with PBDB-T exhibited enhanced *J*_SC_, FF, PCE, and electron mobility values with increasing halogen atom size, giving the highest efficiency of 12.1% for **7-315**.^200^ Wang et al. introduced molecules with a similar backbone as **7-313**–**7-315** but nitro-substituted end groups, where NO_2_ is situated either anywhere on the INCN phenyl ring (**7-316**, F-N1)^311^ or on a specific position (**7-317**, F-N2).^311^ The nitro substitution does not largely affect the energy levels and optical band gaps when compared to acceptors **7-313**–**7-315**. Solar cells were built with PM6, and the *V*_OC_s of the **7-316**- and **7-317**-based cells deviate by 0.20 V, due to the higher energy loss of 0.85 eV for **7-316** compared to 0.70 eV for **7-317**. The devices based on **7-316** yielded a lower *V*_OC_ but higher *J*_SC_ and FF, leading to an efficiency of 10.7%. The blend with the higher *V*_OC_ of 0.94 V (PM6/**7-317**) reached an overall efficiency of 11.9%. Differences are also observed in the molecular packing of the two acceptors and acceptor/donor blends, indicating a more ordered packing for **7-317** neat and blend films.^311^ Molecule **7-318** (F-2F)^309^ resembles **7-317** in its backbone, but it contains INCN-2F units. **7-318** and the pyran-bridged molecule **7-311** have the same side chains and end groups, and in comparison, **7-318** has a higher LUMO and lower HOMO energy level, which widens the band gap to 1.60 eV. A great deviation is also visible in the PV parameters, as **7-318** gave a much higher *V*_OC_ of 0.94 V, while the other parameters are lower. The highest efficiency reached with **7-318** was 12.9% (15.1% for **7-311**). Absorption spectra show a red-shift of **7-311** and a broader EQE range.^309^

NFA **7-319** (FXIC-1)^312^ possesses a similar structure as **7-318** with the only difference of having other side chains. The LUMO energy level of **7-319** is lower than that for **7-318**; thus, the optical band gap is widened. However, PV parameters with PTB7-Th are much lower for **7-319**, giving an efficiency of 7.13%.^312^

The NFAs **7-320**–**7-325** have a central carbazole moiety in the donor unit with cyclopentathiophenes fused on both sides, and they differ in their end groups and side chains on the donor unit. **7-320** (C8-DTC)^313^ comprises octyl side chains on the cyclopentadienyl rings, 1-octylnonyl chains on the carbazole, and INCN-F units. **7-321** (DTC(4R)-4FIC)^195^ has the same side chains but INCN-2F end groups. **7-322** (DTC(4Ph)-4FIC)^196^ bears the same *N*-alkyl chain as **7-321** but octylphenyl side chains on the cyclopentadiene units. **7-320** and **7-322** were blended with PM6 and **7-321** with J71 for the solar cell preparation and are thus difficult to compare. The best photovoltaic performance is revealed by **7-322**, showing a *V*_OC_ of 0.95 V, a FF of 75%, a *J*_SC_ of 18.3 mA cm^–2^, and a PCE of 13.2%. Also, the other molecules show their potential as NFAs in solar cells with PCEs of 12.1% (**7-320**) and 10.9% (**7-321**).^195,196,313^ Structure **7-323** (DTCC-BC)^134^ shares the backbone and side chains with **7-320** and **7-321** but has extended aromatic acceptor units (cf. also **7-35** and **7-257**), leading to heightened HOMO/LUMO energy levels compared to **7-320** and **7-321**. Solar cells yielded lower PCEs of 10.7%, but different donors were used; however, compared to the NFA with unsubstituted INCNs (**7-128**), the PV parameters are improved using for both the same donor (PBDB-T) (9.25% for **7-128**).^134^ NFAs **7-324**–**7-326** are still carbazole-based but with an octyl chain on the nitrogen atom. **7-324** (DTCCIC-4F)^197^ comprises INCN-2F, whereas **7-325** (DTCC-4Cl)^314^ and **7-326** (DTSiC-4Cl)^314^ bear INCN-2Cl units. In **7-326**, the cyclopentadiene rings are replaced with siloles. Solar cells of **7-324** with PFBDB-T yielded 12.6% PCE, which is much higher than the efficiency of the INCN-containing counterpart **7-129** (6.20%).^197^ The PV parameters of solar cells with **7-325** and **7-326** (with T1 as a donor) are further improved, leading to efficiencies of over 14%.^314^ The energy levels of these two acceptors are quite similar, but the band gap of **7-326** is slightly widened. **7-327** (FTBT)^315^ has the same aromatic rings but the benzyl and cyclopentadienyl units have exchanged positions, leading to the donor motif T-B-Cp-T-Cp-B-T with three 5-membered rings in the middle, shifting the side chains on the cyclopentadiene units closer together. Solar cells with PM6 give a *V*_OC_ of 0.96 V, but all other parameters are significantly decreased, achieving PCEs of only 3.47%.^315^ The backbones of **7-328** (DBTIC-2F)^199^ and **7-329** (DBTTC)^199^ resemble the backbones of **7-320**–**7-325**, but replacing the carbazole by a dibenzo[*b*,*d*]thiophene unit leads to the donor unit motif T-Cp-B-T-B-Cp-T. This widens the optical band gap to 1.6 eV. Blended with PBDB-T, solar cells of **7-328** and **7-329** achieved PCEs of 6.81 and 11.3%, respectively.^199^

Figure 14 also shows some acceptors with two seven-ring donor units (**7-330**–**7-332**) or a truxene donor unit (**7-333**–**7-335**). Molecules **7-330** (FXIC-2)^312^ and **7-331** (FXIC-3)^312^ are based on the backbone of **7-318** with the difference that two of these units are bound together via the central Cp-ring either through a double bond or by sharing one carbon atom (spiro-linkage). Between these three molecules, the variation of the energy levels and optical band gaps is negligible (Table 10). Solar cells were built with PTB7-Th as the donor and yielded quite low efficiencies, being the highest for **7-319** (7.06%), followed by **7-331** (6.12%) and **7-330** (5.22%).^312^ Jiang et al. introduced molecule **7-332** (99CZ-8F).^316^ It is similar to **7-330** and **7-331**, but has central carbazole units, which are linked by a *N*–*N* single bond. It gave a slightly lower LUMO energy level, leading to a narrowed band gap. Solar cells with PM6 reached slightly higher PCE values than **7-330** and **7-332**, achieving the highest efficiency with 6.60% with a much higher *V*_OC_ of 0.94 V.^316^

Truxene-core-based acceptors **7-333**–**7-335** contain the same BT π-bridge and RCN end groups. **7-333** (meta-TrBRCN)^317^ and **7-334** (para-TrBRCN)^317^ are structural isomers with butyl side chains on the cyclopentadienyl units of the truxene. **7-335** (Tr(Hex)_6_-3BR)^204^ differs from the two others by the side chains having hexyl chains instead. All three acceptors are wide band gap materials (over 2.00 eV). All four molecules were tested in solar cells with the low band gap donor polymer PTB7-Th. **7-333** and **7-334** gave good results, achieving *V*_OC_s of 0.94 and 0.95 V and efficiencies of 10.2 and 8.29%, respectively. In contrast, **7-335** showed a *V*_OC_ over 1 V but reached only a PCE of 2.10%.^204,317^

## 4 Five Fused Aromatic Ring Systems

Among the non-fullerene acceptors containing five aromatic rings in their central core, indacenodithiophene (IDT)-based structures have become the most investigated. Its rigid, coplanar, and electron-rich fused-ring structure impedes rotational freedom leading to a lower reorganization energy.^318^ A large variety of different chemical structures are available through the modification of the backbone, the side chains, and/or the end groups.

The side groups of the IDT mainly influence the morphological properties of the molecule. However, by changing the hybridization of the sp^3^ carbon atom or by replacing it with a heteroatom, also the (opto)electrochemical properties can be tuned. Though, variations of the electron-withdrawing end groups show a larger impact on those properties.^37^ The latest examples from research dealing with these aforementioned modifications are summarized in the first part of this section. This is followed by a larger set of compounds based on the IDT core but containing also a π-spacer unit. By elongation of the π-conjugation, such building blocks influence molecular geometry and therefore film morphology and have a significant effect on optical as well as electrochemical properties. Finally, acceptors based on other five-ring systems are summarized in the end of this section.

### 4.1 IDT-Based A–D–A Systems

**Figure 15:**
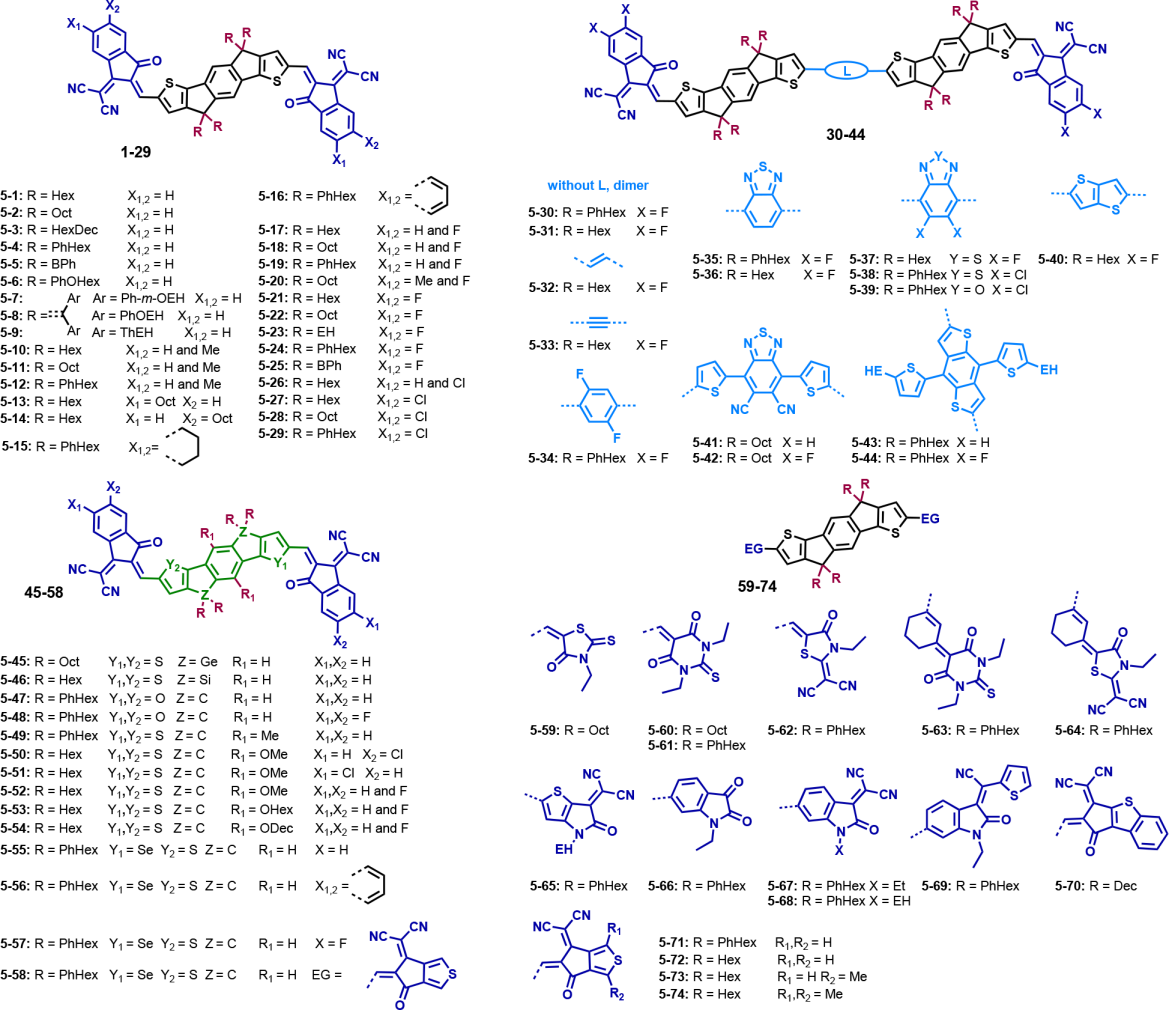
Structures of non-fullerene acceptors with IDT core units. Upper left, variations of side chains and INCN-based end groups; upper right, dimeric IDT-based structures with different linkers; lower left, IDT-based core units with different heteroatoms and/or side chain and end group variations; lower right, IDT core units with varying end groups.

IDIC (**5-1**) is structurally similar to ITIC (vide supra) and was designed and synthesized by Lin et al. in 2016 via a one-step Knoevenagel condensation of C6IDT-CHO with INCN.^37^ It comprises an IDT core decorated with hexyl side chains and INCN end groups (Figure 15).

The molecule has a strong absorption in the range from 500 to 800 nm, which is an important feature of all IDC-based compounds. Solar cells with PTBT-T1 obtain a *V*_OC_ of 0.89 V, a *J*_SC_ of 15.1 mA cm^–2^, a FF of 65%, and a PCE of 8.19%.^37^ Since then, IDIC has been tested in blends with various donors, to name a few—PTZP (PCE: 11.8%),^319^ FTAZ (PCE: 12.5%),^320^ PM6,^321,322^ PBDB-T,^322^ and PTQ10.^323−325^ These devices typically possess good *V*_OC_ and FF; however, *J*_SC_ values rarely reach 20 mA cm^–2^, since the optical band gap of IDIC is relatively high (1.6 eV). Interestingly, also bilayer-junction devices based on PTQ10/IDIC^323^ and PM6/IDIC^326^ blends reach efficiencies which are comparable to those of the BHJ-based devices with the same donors.

#### 4.1.1 Side Chain and End Group Modifications on IDIC-Based NFAs

The role of the side chains in fused five-ring systems is very similar to what has been described in seven-ring systems; however, due to the central core being smaller, effects of the side chains are larger. Small elongation of the side chains on the IDT central core (**5-2**, IDIC-C8, R = octyl)^327^ has a minimal influence on the solar cell performance, while much longer side chains (**5-3**, IDIC16, R = hexadecyl)^328^ have a negative impact, particularly on the fill factor (cf. solar cell parameters with PM6, Table 11). *para*-Hexylphenyl groups (**5-4**, ITIC-2T/IDT-IC) are also often used as side chains on the IDT core.^329,334^ Due to their larger steric hindrance, IDT becomes less crystalline than its hexyl-substituted analogue but delivers a lower PCE (6.14%) than **5-1** in solar cells with PBDB-T.^322^ A lower FF is the main reason for the reduced PCE, while a higher optical band gap leads to a reduced current. Structure **5-5** (IDIC-C4Ph)^322^ comprises phenylbutyl side groups, where the butyl group acts as a spacer between the IDT backbone and the bulky phenyl ring. It combines the good properties of hexyl (as in structure **5-1**) and hexylphenyl (as in **5-4**) side chains and delivers similar or better PCEs with the same polymer donors (PM6, PBDB-T). Analysis of molecular stacking in the films of the three acceptors (**5-1**, **5-4**, and **5-5**) confirms that the acceptor with hexyl side chains (**5-1**) exhibits a face-on stacking orientation; the same is found in compound **5-5** with phenylbutyl side groups present albeit with a weakened π–π stacking/crystallization behavior. Compound **5-4** comprising hexylphenyl side groups, on the other hand, demonstrates the weakest crystallization behavior and has an edge-on orientation.^322^

**Table 11:** Optical, Electrical, and Photovoltaic Properties of Non-Fullerene Acceptors **5-1**–**5-74**

NFA	original name	HOMO^a^ (eV)	LUMO^a^ (eV)	*E*_g_^opt^ (eV)	donor	D:A ratio	*V*_OC_ (V)	*J*_SC_(mA cm^–2^)	FF (%)	PCE (%)	μ_e_^e^(cm^2^ V^–1^ s^–1^)	ref.
**5-1**	IDIC	–5.70	–3.92	1.63	PM6	1:1	0.95	18.2	70	12.0	-/1.9 × 10^–4^	(322)
	IDIC	–5.74^d^	–3.90^d^		PTQ10	1:1	0.96	18.6	74	13.2	-/7.8 × 10^–4^	(324)
**5-2**	IDIC-C8	–5.63	–3.86		PM6		0.97	14.1	75	10.2	-/8.8 × 10^–4^	(327)
**5-3**	IDIC16	–5.82	–3.87	1.64	PM6	1:1	0.99	11.8	51	5.96	1.4 × 10^–4^/5.0 × 10^–5^	(328)
**5-4**	ITIC-2T	–5.61	–3.93^b^	1.68	PBDTsTh-BDD	1:1	0.98	12.3	56	6.7		(329)
**5-5**	IDIC-C4Ph	–5.70	–3.93	1.62	PM6	1:1	0.94	19.1	78	14.0	-/4.5 × 10^–4^	(322)
**5-6**	IDT-PhIC	–5.67	–3.89	1.68	PBDB-T	1:1	0.93	9.99	55	5.11	-/1.8 × 10^–5^	(330)
**5-7**	*m*-IDTV-PhIC	–5.63	–3.78	1.59	PBDB-T	1:1	0.99	11.6	51	5.85	-/2.0 × 10^–5^	(330)
**5-8**	IDTV-PhIC	–5.52	–3.84	1.55	PBDB-T	1:1	0.98	4.93	37	1.77	-/4.3 × 10^–6^	(330)
**5-9**	IDTV-ThIC	–5.43	–3.72	1.52	PBDB-T	1:1	0.91	2.06	33	0.62	-/4.8 × 10^–6^	(330)
**5-10**	IDT-C6	–5.70	–3.98	1.65	PM6	1:1	0.96	17.5	74	12.5	3.9 × 10^–4^/-	(331)
**5-11**	IDIC8-M	–5.51	–3.72	1.66	DRCN5T	1:1	1.00	10.4	61	6.31	-/1.1 × 10^–4^	(332)
**5-12**	IDT-PhC6	–5.69	–3.94	1.74	PM6	1:1	1.00	13.0	43	5.6	1.4 × 10^–5^/-	(331)
**5-13**	IDICO1	–5.69	–3.81	1.65	PBDB-T	1:1	0.92	15.1	68	9.40	4.1 × 10^–5^/4.5 × 10^–5^	(333)
**5-14**	IDICO2	–5.74	–3.86	1.60	PBDB-T	1:1	0.92	14.8	69	9.35	4.8 × 10^–5^/2.4 × 10^–5^	(333)
**5-15**	IDT-HN	–5.92	–3.86	1.68	PBDB-T	1.5:1	0.93	14.4	76	10.2	2.8 × 10^–4^/4.1 × 10^–4^	(334)
**5-16**	TPT-IN	–5.80	–3.97	1.58	PBT1-C	1:1	0.88	13.9	73	8.91	4.7 × 10^–4^/4.0 × 10^–4^	(335)
**5-17**	IDIC-2F	–5.72	–3.99	1.60	PTQ10	1:1	0.90	19.0	76	13.0	1.7 × 10^–3^/9.0 × 10^–4^	(336)
**5-18**	IDIC-2F	–5.75	–3.94	1.59	BDTF-CA	1:0.6	0.94	16.7	58	9.11	-/1.6 × 10^–3^	(337)
**5-19**	TPT-2F	–5.84	–4.06	1.64	PBT1-C	1:1	0.87	13.9	69	8.33	4.2 × 10^–4^/7.1 × 10^–5^	(299)
**5-20**	MF1	–5.73	–3.89	1.54	PM7	1:1	0.94	16.8	78	12.4	1.3 × 10^–3^/5.2 × 10^–4^	(168)
**5-21**	IDIC-4F	–5.78	–4.00	1.60	PTQ10	1:1	0.81	18.6	74	11.1	1.1 × 10^–3^/6.3 × 10^–4^	(336)
**5-22**	IDIC-4F				ZR2-C3	1:0.6	0.78	19.0	70	10.4	-/6.4 × 10^–4^	(338)
**5-23**	ID4F	–5.83	–3.86	1.64	PM6	1:1.2	0.84	13.4	61	6.88	-/3.4 × 10^–5^	(339)
**5-24**	IDIC-4F	–5.79	–4.11	1.63	PM6	1:1	0.84	15.2	72	9.26	-/4.3 × 10^–5^	(340)
**5-25**	C4Ph-IDT-DFIC	–5.76	–4.08	1.54	PM6	1:1	0.80	17.8	74	10.5	-/7.2 × 10^–5^	(340)
**5-26**	BThIND-Cl	–5.64	–3.96	1.49	PM6	1:1.5	0.96	18.1	71	12.4	-/1.2 × 10^–5^	(341)
**5-27**	ID-4Cl	–5.81	–4.01	1.51	PM6	1:1	0.77	17.9	74	10.3	-/4.3 × 10^–5^	(342)
	ID-4Cl	–5.81	–4.01	1.51	PTQ10	1:1.5	0.85	17.3	66	9.5	-/4.7 × 10^–5^	(161)
**5-28**	IDIC-4Cl	–5.88	–3.98	1.59	BSC1	1:0.7	0.86	21.5	70	13.0	-/7.7 × 10^–5^	(343)
**5-29**	IDIC-4Cl	–5.78	–4.02	1.58	PM7	1:1	0.83	16.2	69	9.24	-/2.7 × 10^–4^	(344)
**5-30**	IDT2-DFIC	–5.39	–3.97	1.42	PBDB-T	1:1.2	0.91	16.1	69	10.1	-/4.9 × 10^–4^	(345)
**5-31**	C6-IDT2-DFIC	–5.31	–4.00	1.44	PBDB-T	1:1	0.81	22.6	68	12.3	-/6.6 × 10^–4^	(346)
**5-32**	DIDIC	–5.44	–3.91	1.40	FTAZ	1:1.5	0.82	17.5	65	9.3	2.2 × 10^–3^/1.4 × 10^–4^	(347)
**5-33**	TIDIC	–5.59	–3.88	1.55	FTAZ	1:1.5	0.88	20.2	74	13.1	4.9 × 10^–3^/1.1 × 10^–3^	(347)
**5-34**	DFB-dIDT	–5.53	–3.87	1.64	PBDB-T	1:1	0.86	15.6	50	6.71	-/1.1 × 10^–5^	(348)
**5-35**	BT-dIDT	–5.34	–3.87	1.54	PBDB-T	1:1	0.87	18.6	65	10.5	-/1.2 × 10^–4^	(348)
**5-36**	BTIDIC	–5.48	–3.97	1.47	J71	1:1	0.86	19.8	67	11.5	5.5 × 10^–4^/4.4 × 10^–4^	(349)
**5-37**	BID-4F	–5.69	–3.73	1.53	PM6	1:1.2	0.92	17.8	74	12.3	-/7.0 × 10^–4^	(350)
**5-38**	BT2FIDT-4Cl	–5.70	–3.88	1.56	PM7	1:1	0.97	18.1	72	12.5	-/1.2 × 10^–4^	(351)
**5-39**	BO2FIDT-4Cl	–5.73	–3.89	1.59	PM7	1:1	0.96	16.1	61	10.4	-/3.6 × 10^–5^	(351)
**5-40**	TTIDIC	–5.43	–3.92	1.46	J71	1:1	0.91	10.7	67	6.54	3.7 × 10^–4^/2.2 × 10^–4^	(349)
**5-41**	CNDTBT-C8IDT-INCN	–5.54	–3.82	1.48	PBDB-T	1:1	0.86	19.4	67	11.2	-/9.3 × 10^–5^	(352)
**5-42**	CNDTBT-C8IDT-FINCN	–5.58	–3.92	1.40	PBDB-T	1:1	0.79	22.1	70	12.3	-/4.1 × 10^–4^	(352)
**5-43**	BDT(IDT-IC)_2_	–5.53	–3.96	1.59	PBDB-T	1:1	0.89	11.0	51	4.98	5.5 × 10^–5^/4.8 × 10^–6^	(353)
**5-44**	BDT(IDT-IC-2F)_2_	–5.51	–3.97	1.54	PBDB-T	1:1.5	0.83	13.6	55	6.21	2.2 × 10^–4^/1.1 × 10^–5^	(353)
**5-45**	GDIC-C8	–5.67	–3.86		PM6		1.02	11.4	58	6.76	-/5.3 × 10^–4^	(327)
**5-46**	SiIDT-IC	–5.47^b^	–3.78	1.69	PBDB-T	1:1	0.92	13.5	66	8.16	-/1.0 × 10^–4^	(354)
**5-47**	IDF-IC	–5.75	–4.13	1.62	PM6	1:1	0.91	14.6	59	7.80		(355)
**5-48**	IDF-4F	–5.83	–4.27	1.56	PM6	1:1	0.74	17.5	61	7.81		(355)
**5-49**	ID-MeIC	–5.68	–3.58	1.51	PBDB-T	1:0.8	0.90	14.1	50	6.46	1.0 × 10^–6^/3.4 × 10^–6^	(356)
**5-50**	MO-IDIC-Cl-1	–5.80	–3.91	1.55	PTQ10	1:1.1	0.88	17.7	69	10.8	5.1 × 10^–4^/4.0 × 10^–4^	(238)
**5-51**	MO-IDIC-Cl-2	–5.79	–3.92	1.54	PTQ10	1:1.1	0.88	19.2	74	12.5	7.3 × 10^–4^/7.3 × 10^–4^	(238)
**5-52**	MO-IDIC-2F	–5.80	–3.93	1.55	PM6	1:1	0.84	18.9	77	12.2	8.9 × 10^–4^/6.2 × 10^–4^	(357)
**5-53**	HO-IDIC-2F	–5.81	–3.91	1.55	PM6	1:1	0.86	19.1	76.	12.5	1.1 × 10^–3^/7.2 × 10^–4^	(357)
	HO-IDIC-2F	–5.81	–3.91		PTQ10	1:1	0.92	19.0	71	12.4	-/1.3 × 10^–3^	(358)
**5-54**	DO-IDIC-2F	–5.79	–3.88	1.54	PM6	1:1	0.86	19.6	77	13.0	1.1 × 10^–3^/7.3 × 10^–4^	(357)
**5-55**	T-Se	–5.61	–3.83	1.62	PM6	1:1	0.93	12.8	62	7.44	-/3.8 × 10^–5^	(359)
**5-56**	SePT-IN	–5.77	–4.00	1.54	PBT1-C	1:1	0.85	16.4	73	10.2	7.1 × 10^–4^/6.4 × 10^–4^	(335)
**5-57**	T-Se-4F	–5.62	–3.93	1.58	PM6	1:1	0.78	18.1	67	9.41	-/4.1 × 10^–4^	(359)
**5-58**	T-Se-Th	–5.61	–3.88	1.60	PM6	1:1	0.91	16.9	67	10.3	-/5.1 × 10^–4^	(359)
**5-59**	A1	–5.61	–3.55	1.95	P3HT	1:0.8	0.93	8.69	66	5.39	-/5.8 × 10^–5^	(360)
**5-60**	IDTA	–5.88^c^	–3.90	1.90	PBDB-T	1:1	0.99	12.4	60	7.40	7.6 × 10^–5^/4.3 × 10^–5^	(170)
**5-61**	IDT-TBA	–5.91^c^	–4.00	1.91	PBDB-T	1:1	1.00	11.2	57	6.70	-/5 × 10^–5^	(361)
**5-62**	IDT-R	–5.44	–3.53	1.96	P3HT	1:1	0.73	3.73	51	1.57		(140)
**5-63**	IDT-CT	–5.39	–3.67	1.63	PTB7-Th	1:1.4	0.93	11.2	42	4.62		(140)
**5-64**	IDT-CR	–5.34	–3.44	1.78	P3HT	1:1	0.66	2.62	49	0.99		(140)
**5-65**	TIM-IDT	–5.36	–4.01	1.43	PTB7-Th	1:1	0.59	6.04	48	1.96	-/3.4 × 10^–7^	(362)
**5-66**	IDNO-IDT	–5.45	–3.65	1.95	PBDB-T	1:1	1.03	2.65	35	1.10	-/2.4 × 10^–7^	(363)
**5-67**	IM1-IDT	–5.46	–4.02	1.48	PBDB-T	1:1.5	0.55	5.07	47	1.62	-/8.4 × 10^–7^	(363)
**5-68**	IM-IDT	–5.44	–4.00	1.48	PTB7-Th	1:1	0.58	4.96	49	1.60	-/1.9 × 10^–7^	(362)
**5-69**	ITAN-IDT	–5.42	–3.69	1.68	PBDB-T	1:1.5	1.18	3.71	39	2.00	-/1.0 × 10^–6^	(363)
**5-70**	IBCT	–5.50	–3.70	1.65	L1	1:1.2	1.02	15.1	74	11.3	-/2.5 × 10^–4^	(364)
**5-71**	IDTCN	–5.91	–3.94	1.67	PBT1-EH	1:1	0.93	13.1	70	8.69	-/2.3 × 10^–3^	(365)
**5-72**	IDTPC	–5.84	–3.98	1.52	PTQ10	1:1	0.93	17.5	75	12.2	8.0 × 10^–4^/3.7 × 10^–4^	(366)
**5-73**	IDTPC-Me	–5.62	–3.92	1.55	PTQ10	1:1	0.95	13.8	70	9.2	6.5 × 10^–4^/3.1 × 10^–4^	(367)
**5-74**	IDTPC-DMe	–5.58	–3.85	1.57	PTQ10	1:1	1.02	13.5	68	9.3	5.4 × 10^–4^/2.3 × 10^–4^	(367)

a Obtained from the oxidation/reduction potential of the CV measurement if not otherwise stated.

b HOMO/LUMO energy levels obtained from the LUMO/HOMO levels determined by CV and the optical band gap.

c HOMO obtained via PESA.

d Other method or method not defined.

e Determined via the SCLC technique from the neat acceptor/donor:acceptor blend films if not otherwise stated.

Introducing a hexyloxyphenyl side chain (**5-6**, IDT-PhIC)^330^ has practically no influence on the optical properties. This is not very surprising, as the side chains are attached to a sp^3^ hybridized carbon in the IDT backbone and, thus, they are not a part of π-conjugation. Solar cells with PBDB-T show a lower performance than those with **5-4** (cf. Table 11). In contrast to **5-6**, the optical properties significantly change in structures **5-7**–**5-9**, where one diphenylmethylene or dithienylmethylene group is attached to the IDT core instead of two alkyl or aryl side groups, thereby extending the conjugation, while decreasing the optical band gap. This effect scales with the electron-donating ability of the methylene moieties (**5-7** < **5-8** < **5-9**), leading to optical band gaps from 1.59 eV, over 1.55 eV to 1.52 eV. Solar cells of the *meta*-alkoxyl isomer (**5-7**, *m*-IDTV-PhIC) with PBDB-T exhibit a similar performance to those of **5-6** (with non-conjugated hexyloxyphenyl groups), while the *para*-alkoxyl isomer **5-8** (IDTV-PhIC) and the thiophene derivative **5-9** (IDTV-ThIC) show a drop of PCE.^330^

The influence of substituents on the INCN end group follows the same general characteristics as described in the previous section (Seven Fused Aromatic Ring Systems), e.g., for ITIC-based NFAs. Consequently, the introduction of methyl groups as in compounds **5-10** (IDT-C6), **5-11** (IDIC8-M), and **5-12** (IDT-PhC6) only marginally influences the optical or electrochemical properties, leading to slightly higher optical band gaps (compared to **5-1**, **5-2**, and **5-4**, respectively).^331,332^ Solar cells of **5-10** and PM6 show the highest performance reaching PCE values of up to 12.5% compared to 5.6% for solar cells with **5-12**, which was explained by a better film packing of **5-10** comprising hexyl side chains on the IDT core. A blend of **5-10** with PM6 had a favorable face-on orientation, while **5-12** with hexylphenyl side chains in a blend with PM6 had an amorphous morphology.^331^

Compounds **5-13** (IDICO1) and **5-14** (IDICO2) having octyl chains at different positions on the INCN unit reveal slightly raised LUMO levels compared to the unsubstituted **5-1**. Solar cells of both acceptors with PBDB-T lead to similar PCEs values of approx. 9.4% by keeping the good electron mobility and FF, but increasing the *J*_SC_, compared to **5-1** (PCE 6.96%). The increased current density was attributed to a better light absorption of the octyl derivatives in film as well as improved intermixing with the donor polymer.^333^ Structure **5-15** (IDT-HN), which contains a cyclic alkyl substituent, demonstrated very similar improvements in the same parameters when compared to the unsubstituted compound **5-4**, with nearly 70% increase in PCE (10.2% vs 6.11%) as **5-15** has an improved π–π stacking and a face-on orientation in film.^334^ The naphthalene-based analogue **5-16** (TPT-IN) has a packaging behavior very similar to that of **5-15** but smaller electrochemical and optical band gaps due to the extended aromatic system. Solar cells with PBT1-C reached a PCE of 8.91%.^335^ Halogenation of the INCN groups (compounds **5-17**–**5-29**) has similar effects as described in the seven-ring section and leads to lower HOMO and LUMO energies and lower optical band gaps (in film). Here, fluorination leads only to a slight reduction in the band gap, whereas chlorination leads to molecules with optical band gaps of approx. 1.5 eV. As a result, solar cells with the halogenated derivative and the same donor material have usually decreased *V*_OC_ values but an improved *J*_SC_. The electron mobilities in blends are similar or improved. However, there is up to now no clear trend on the final PCE values, probably also due to a lack of comparative device studies. For example, solar cells with the non-halogenated derivative **5-1** show a better performance with PCE values of 13.2%^324^ using PTQ10 as a donor polymer, compared to similar values of 13.0% for devices based on **5-17** (IDIC-2F) bearing INCN-F units.^336^ However, both are significantly better than those of the acceptor **5-21** (IDIC-4F, 11.1%) with INCN-2F end groups.^336^ Solar cell data for the chlorinated structures **5-26** (BThIND-Cl, INCN-Cl units)^341^ and **5-27** (ID-4Cl, INCN-2Cl units)^342^ with another donor, PM6, also show better PCE values of 12.4% for devices with the dichlorinated NFA **5-26**. However, in the case of the octyl derivatives (**5-2**,^327^**5-18**, IDIC-2F,^332,337^**5-22**, IDIC-4F,^337,338^ and **5-28**, IDIC-4Cl^343^), the best solar cell performances were obtained for solar cells with compound **5-28** bearing INCN-2Cl units (PCE values of 13% with BSC1 as a donor), whereas, for the hexylphenyl series, the best solar cells were reported for the combination of compound **5-24** (IDIC-4F) with PM6 (9.26%),^340^ which is very similar to devices based on **5-29** (IDIC-4CL9) and PM7.^344^ Regarding the phase separation and molecular arrangement, as expected, a face-on orientation is observed for the compounds which have alkyl side chains on the IDT core. Interestingly, for the tetrachloro-substituted compound with hexyl side chains (**5-27**), an edge-on orientation was observed in pristine film,^161^ while the octyl-substituted compound **5-28** had a face-on orientation.^343,368−370^**5-24**, a tetrafluoro-substituted compound with hexylphenyl side chains, showed no preferred orientation in film.^371,372^ A comparison of **5-19** (TPT-2F), which contains INCN-F units and hexylphenyl side chains on the IDT core, with its ring extended analogues TPTT-2F (fused additional thienothiophene, see **6-16**) and TPTTT-2F (fused additional cyclopentadithiophene, see **7-291**) demonstrates how small changes in conjugation length can already be enough to improve the π–π stacking. In the case of a six- and seven-ring central core (compounds **6-16** and **7-291**, respectively), a dominant face-on orientation in film is observed, which is not the case for compound **5-19** with the five-ring central core.^299^ Since the size and shape of domains formed by the active layer components in the BHJ are mutually affected, changes in the acceptor structure can also influence the behavior of the polymer donor. This is nicely illustrated in a study, where the authors prepared blends of PTQ10 with four different NFAs bearing the same end group, **5-27** (ID-4Cl), **7-7**, **7-69**, and **7-158**. The donor in each of the blends had rod-shaped domains but with different radius. When **5-27** was used as an acceptor, the radii of PTQ10 domains were the largest and the smallest in the case when **7-158** (Y7) is used. Consequently, PTQ10/**5-27** had the smallest interfacial area. Thus, despite the high crystallinity of the PTQ10/**5-27** blend, it had the most unbalanced hole and electron mobility, lowest *J*_SC_, FF, and PCE.^161^ This study underlines the importance of obtaining further understanding on how the blend morphology is influenced by relatively small changes in the NFA structure in order to design more efficient acceptors. To that end, the IDT central core is a suitable platform. Since it consists of only five fused rings, it permits the investigation of, for example, various end groups, without imposing too dominant effects itself. IDIC and its halogenated derivatives are often used for the development of new donor polymers^157,373^ or small molecule donors.^370,374−377^

#### 4.1.2 Dimeric IDT Acceptors

The linking of two IDT units either directly or via a conjugated spacer (L) leads to the A–D–(L)–D–A motif. The structure **5-30** (IDT2-DFIC) has an extended absorption spectrum and a reduced optical band gap compared to the monomeric analogue **5-24**. Furthermore, solar cells based on PBDB-T/**5-30** blends reached a nearly doubled PCE of 10.1% and a higher *V*_OC_, *J*_SC_, and FF.^345^ Structure **5-31** with hexyl chains on the IDT core exhibits an improved crystallinity of the acceptor, thus reaching higher electron mobility and *J*_SC_ values. This results in an improved PCE (12.3%) in solar cells using PBDB-T as a donor.^346^ Incorporation of a double bond (**5-32**, DIDIC) between the two IDT units has a minimal effect on the HOMO and LUMO levels and consequently also to the band gap. Insertion of a triple bond (**5-33**, TIDIC) lowers the HOMO level and thus increases the band gap. Solar cells of **5-33** and FTAZ as the donor reach PCE values up to 13.1%.^347^

If electron-withdrawing (A) or -donating (D′) units are incorporated, formal A′–D–A–D–A′- and A–D–D′–D–A-type structures are generated. The addition of electron-withdrawing difluorobenzene leads to structure **5-34** (DFB-dIDT) with a higher optical band gap (1.64 eV) than **5-33** (1.55 eV), whereas the BT-linked NFA **5-35** (BT-dIDT) exhibits a similar value (1.54 eV). Solar cells with blends of these compounds with a PBDB-T donor have nearly identical *V*_OC_s (consistent with the equal LUMO energies), but all other solar cell parameters are better for the benzothiadiazole linked compound **5-35** (PCE 10.5%). The better performance of this compound was assigned to its higher planarity.^348^ Exchanging the hexylphenyl groups on the IDT cores with hexyl side chains gives structure **5-36** (BTIDIC) whose solar cells with the donor J71 give a higher PCE of 11.5%.^349^ If the benzothiazole linker is additionally fluorinated as in compound **5-37** (BID-4F), solar cells with even higher PCE values up to 12.3% were achieved (blend with PM6).^350^ Replacing the fluorine atoms in the INCN group with chlorines leads to structure **5-38** (BT2FIDT-4Cl), and solar cells of this compound with PM7 as a donor give similar PCE values (12.5%).^351^ The substitution of benzothiadiazole with benzoxadiazole gives structure **5-39** (BO2FIDT-4Cl). Solar cells with the donor PM7 gave lower PCE values (10.4%) than the solar cells based on **5-38**. However, this decrease of the PCE cannot be assigned only to the weaker electron-withdrawing strength of benzoxadiazole, as also morphological changes are observed: the benzothiadiazole-linked **5-38** has a face-on orientation (pristine and in blend with PM7), while the pristine film of benzoxadiazole-based **5-39** does not have a clear orientation.^351^ Kim et al. introduced the two NFAs **5-41** (CNDTBT-C8IDT-INCN) and **5-42** (CNDTBT-C8IDT-FINCN) bearing thiophene-flanked dicyanobenzothiazole linkers and either INCN or INCN-2F accepting units. The compounds have relatively low optical band gaps, 1.48 eV for **5-41** and 1.40 eV for **5-42**. Solar cells with PBDB-T gave PCE values up to 11.2 and 12.2%, respectively.^352^ If electron-donating linkers are used, only moderate PCE values are reached. For example, 6.54% with the thienothiophene linked **5-40** (TTIDIC; A-D-D′-D-A-type structure)^349^ and 4.98 and 6.21% with benzodithiophene-based compounds **5-43** (BDT(IDT-IC)_2_) and **5-44** (BDT(IDT-IC-2F)_2_), respectively.^353^

Summarizing these dimerized structures, solar cells with the A′–D–A–D–A′ type of compounds have shown better efficiencies than those with the A–D–D′–D–A type. Fluorinated end groups also have better efficiencies than their non-halogenated counterparts. On average (disregarding the used linker, end groups, or the donor polymer), compounds with alkyl side groups on the IDT core have higher PCE values than those which bear hexylphenyl side groups on the IDT core.

#### 4.1.3 Modification of the IDT Core Unit

Modification of the IDT core can be easily achieved by heterosubstitution or by additional side chains on the central benzene ring. First, replacing the sp^3^ hybridized carbon in the IDT backbone by germanium leads to structure **5-45** (GDIC-C8). The Ge–C bond is longer (1.97 Å) than its C–C analogue (1.53 Å), which makes the molecule more crystalline. This, in return, disrupts the crystallization of PM6 (PCE 6.76%).^327^ In contrast to **5-45**, the silicon analogue **5-46** (SiIDT-IC) in thin film has a favorable face-on orientation. Also, the solar cell efficiency in a blend with PBDB-T is improved to 8.16%.^354^

The substitution of sulfur with oxygen (i.e., replacing thiophene with furan) in the structures **5-4** and **5-24** leads to the NFAs **5-47** (IDF-IC) and **5-48** (IDF-4F), with similar HOMO levels but significant lower LUMO energies consequently leading to smaller optical band gaps. Thus, solar cells with PM6 have lower *V*_OC_s and overall only moderate performances.^355^

Modification of the short axis of the IDT core by introduction of methyl groups on the central phenyl ring (**5-49**, ID-MeIC) results in a reduction of the optical band gap by 160 meV (in contrast to **5-4**), suggesting significant changes in the molecular packing. In films, **5-49** has a dominant face-on orientation, while **5-4** does not show a favorable orientation (*vide supra*). Solar cells with PBDB-T revealed better PCE values compared to the unsubstituted compound (6.46% vs 4.94% for **5-4**).^356^ The introduction of alkoxy groups on the central benzene in combination with halogenated INCN acceptor groups leads to compounds **5-50**–**5-54** with optical band gaps of about 1.55 eV. Optical and electrochemical properties are influenced minimally if chlorine-substituted (**5-50**, MO-IDIC-Cl-1, and **5-51**, MO-IDIC-Cl-2)^238^ or fluorine-substituted (**5-52**, MO-IDIC-2F)^357^ INCN end groups are used. Also, the length of the alkyl chain of the alkoxy group has no influence (**5-53**, HO-IDIC-2F, and **5-54**, DO-IDIC-2F).^357^ All compounds (**5-50**–**5-54**) have a face-on orientation in film, and solar cells with these compounds deliver similarly good efficiencies between 10.8 and 13%.

Asymmetrical NFAs are obtained if only one sulfur in the IDT unit is replaced with selenium: **5-55** (T-Se),^359^**5-56** (SePT-IN),^335^**5-57** (T-Se-4F),^359^**5-58** (T-Se-Th), all with different acceptor end groups.^359^ Their energy levels are slightly raised if compared to their sulfur-containing analogues (**5-4**, **5-16**, **5-24**, and **5-71**, respectively), but the optical band gap is smaller. Solar cells of these compounds with various donor polymers have lower *V*_OC_s but higher *J*_SC_s; thus, similar or higher PCEs are reached (values between 7.44 and 10.3%).^335^ In film, the selenium-containing compound **5-56** has a face-on orientation, just as its sulfur analogue, yet the π–π stacking appears stronger in the Se-containing compound.^335^

#### 4.1.4 Other Acceptor End Groups

End-capping of the IDT core with 3-ethylrhodanine (**5-59**, A1) yields uplifted energy levels and a higher optical band gap compared to **5-2** with the classical INCN unit (1.95 eV vs 1.64 eV). Solar cells with P3HT reached PCE values of 5.39%.^360^ A similar large optical band gap (1.90 eV) is also obtained if a TBA derived end group is used as in **5-60** (IDTA), which reached a PCE of 7.1% with PBDB-T. The same end groups when combined with an IDTT central core (**7-85**) led to a PCE of 10.8%.^170^ The NFA **5-61** (IDT-TBA/TPT-T),^293,361^ in which the octyl side chains are replaced with hexylphenyl side chains, shows similar photovoltaic performance in devices with the same donor (PCE: 6.70%). Again, the NFA with the IDTT central core (**7-86**) reached higher PCE values (7.5%).^361^ Compounds **5-63** (IDT-CT) and **5-64** (IDT-CR) containing a cyclohexene linker were designed in order to increase the (photo)chemical stability of the exocyclic double bond between the IDT core and the end group.^140^ An elongation of the π-system leads to lower optical band gaps than in **5-61** and **5-62** (IDT-R): 1.63 eV for **5-63** and 1.78 eV for **5-64** compared to 1.91 eV for **5-61** and 1.96 eV for **5-62**. Albeit the PCEs of solar cells of **5-63** and **5-64** were not high, these compounds exhibited improved stability to chemical degradation and photo-degradation. Furthermore, it is worth noting that their analogues with the IDTT central core (**7-109** and **7-110**) reached better PCE values.^140^ In these above-mentioned examples (**5-60** to **5-64**), the PCE is boosted upon the replacement of the IDT central core with the slightly larger IDTT core, which illustrates the advantages of an increased conjugation length. Acceptors **5-65**–**5-69** containing the (thio)isatylidene-based end groups have the peculiarity that they are attached to the IDT core by a C–C single bond instead of a vinylene unit. The compounds **5-65**–**5-69** have similar, relatively high lying HOMO energy levels, and the variations of the LUMO energies correspond to the electron-withdrawing strength of the acceptor unit (**5-66**, IDNO-IDT has the highest LUMO).^362,363^ All (thio)isatine-based acceptors suffer from low electron mobilities; thus, solar cells with them yield low PCEs below 2%.^363^ Structure **5-70** (IBCT) contains thiophene-based indandione analogue end groups. Combined with the donor L1, good PCEs up to 11.3% are reached.^364^ Compounds **5-71**–**5-74** comprise CPTCN end groups with slightly weaker electron-withdrawing strength compared to INCN. Compound **5-71** (IDTCN) in solar cells with PBDB-T exhibits PCE values of 6.40%,^378^ and with other donors even higher values (PTQ10, 7.4%; PBT1-EH, 8.69%).^365,366^ Similar to its INCN-based analogue **5-4**, **5-71** does not show a preferential orientation in film.^365,366,378^ Compound **5-72** (IDTPC) on the other hand has a clear face-on orientation in film, due to a replacement of the bulky hexylphenyl side chains with hexyl groups.^366^ Solar cells with PTQ10/**5-72** yielded higher PCE values (12.2%).^366,367^ A methyl substitution on the end group’s thiophene elevates the LUMO energy; thus, a higher *V*_OC_ can be achieved in solar cells with compounds **5-73** (IDTPC-Me) and **5-74** (IDTPC-DMe). At the same time, the lower electron mobility and slightly higher charge carrier recombination lead to a lower PCE. Interestingly, the solubility of **5-74** is only half as good as that of **5-72** (<35 vs >65 mg mL^–1^, respectively) due to an increased crystallinity upon the introduction of the methyl substituents.^367^

### 4.2 π-Spacers

The discussion so far clearly revealed how modifications of the IDT backbone, side chains, or end groups influence the acceptor properties and photovoltaic performance. Also, an addition of a conjugated π-spacer was already described for the dimeric compounds (**5-32**–**5-44**) linked either via an electron-donating or electron-accepting π-linker.

**Figure 16:**
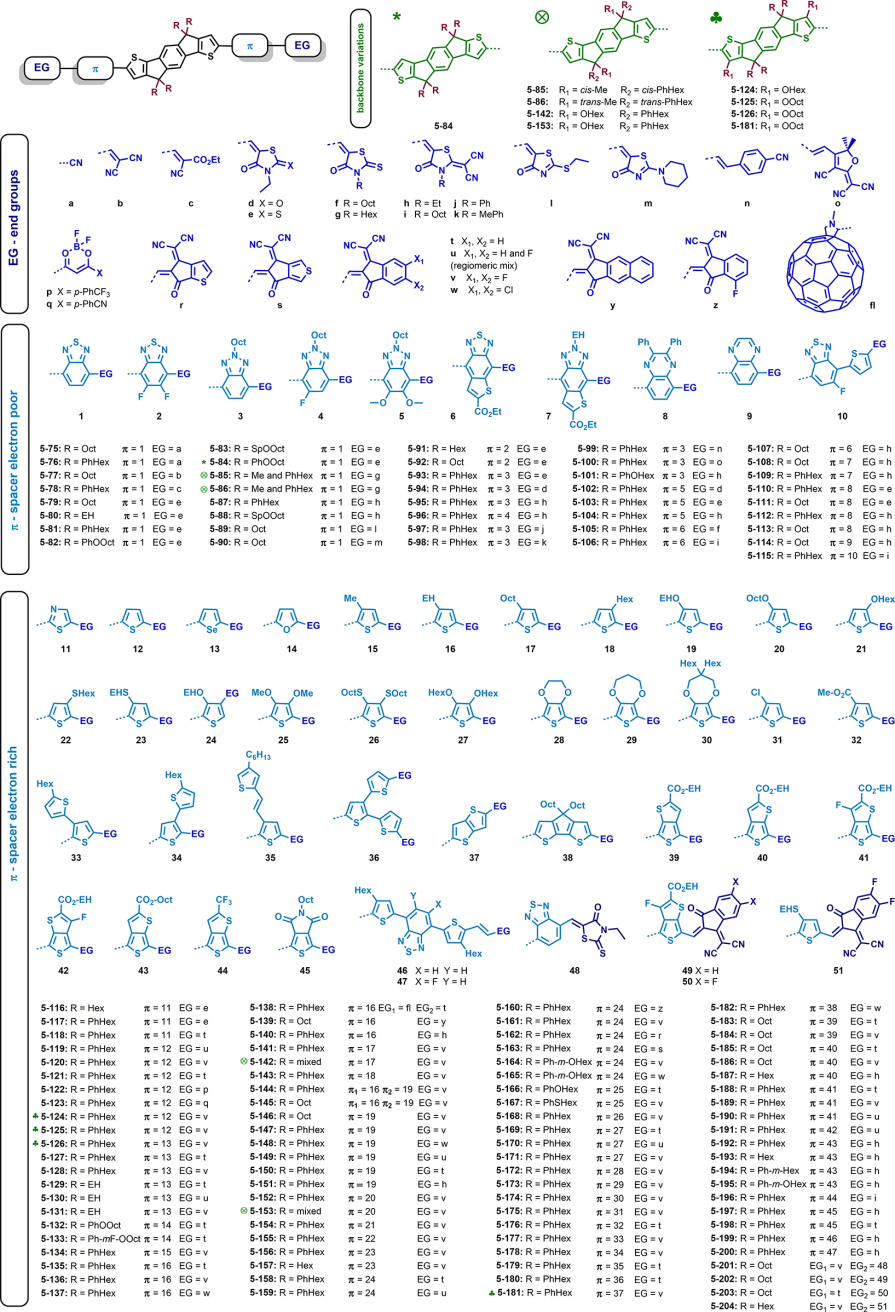
Structures of non-fullerene acceptors based on IDT core units and its derivatives (indicated by symbols next to the compound number) with different π-spacers and end groups.

Thus, in the following, we subdivided the π-spacers into two groups: electron-deficient (benzothiadiazoles, benzotriazoles, quinoxalines, and their derivates) and electron-rich (various thiophene derived compounds), as shown in Figure 16. Of course, this classification is not always unambiguous, especially in cases where in one π-spacer mixed electron-rich and electron-poor heteroatoms are used. Nevertheless, proceeding with a classification into electron-poor and electron-rich spacers gives two types of structural motifs: A′–A–D–A–A′ and A–D′–D–D′–A, respectively. Acceptors belonging in the first class have higher LUMO levels, which yields solar cells with high *V*_OC_s. Meanwhile, the increased electron-rich nature of the acceptors in the second class contributes to very low optical band gaps and thus large *J*_SC_s. π-Spacers can also be used as non-covalent conformational locks, in “like–acceptor–like–donor” strategy (incorporating similar building blocks in acceptor molecules as those used in donors) or for the preparation of asymmetric acceptors.

#### 4.2.1 Electron-Deficient π-Spacers (Benzothiadiazoles, Benzotriazole, Quinoxaline)

A considerable number of acceptors (**5-75**–**5-92**) comprises IDT-based cores with a BT π-spacer and different accepting end groups (see Figure 16). Compounds **5-75**–**5-78** are among the few examples where the end groups are not based on heterocycles. With the simple nitrile end groups in **5-76** (IDT-BC) and **5-75** (IDT-C8-BC), 6.3–7.3% PCE values were achieved (blends with PBDB-T, see also Table 12).^379^ Malononitrile derived end groups like in **5-77** (O-IDTBCN) gave a nearly doubled *J*_SC_ in solar cells (blend with PTB7-Th), leading to high PCE values of 11.1%.^380^ Solar cells of **5-78** (IDT-CA), which contains the less electron-withdrawing cyanoacetate end groups, give PCEs of 4.19% (blend with P3HT).^381^ The change to heterocyclic 3-ethylrhodanine as the end group leads to the NFA O-IDTBR (**5-79**), which exhibits higher PCE values in solar cells with P3HT (up to 7.10%).^404,405^ Solar cells of **5-79** with PTB7-Th reach PCEs between 8.8^406^ and 9.9%,^380^ while those with the donor 2TRA surpass 10%.^407^ Nevertheless, the highest PCE (10.4%) was achieved using PffBT2T-TT as a donor polymer, despite a small LUMO–LUMO offset.^382^ Further variations of this acceptor were prepared with different side chains: ethylhexyl (**5-80**, EH-IDTBR),^383,408,409^ hexylphenyl (**5-81**, IDT-BT-R),^38,384^ octyloxyphenyl (**5-82**, 1-IDTBTRh),^385^ and 2,7-bis(octyloxy)spirofluorene groups (**5-83**, DTFBT-1).^386^ However, none of the solar cells using these NFAs reached PCEs above 10%. Interestingly, if the thiophenes of the central core are swapped upside-down, to point in the same direction as the sp^3^ hybridized carbon (**5-84**, a-IDTBTRh, in Figure 16, see the upper right side for the structure of the central core), the efficiencies of the solar cells are reduced by half (2.53% vs 5.38% for **5-82** in blend with P3HT).^385^ Another pair of isomers are the compounds **5-85** (*cis*-IDT-BT-R) and **5-86** (*trans*-IDT-BT-R), both containing mixed side chains (hexylphenyl and methyl groups, located *cis* or *trans* to each other) on the sp^3^ hybridized carbon. The *trans* isomer (**5-86**) has a higher PCE (9.43% compared to 8.02%) in solar cells with PTB7-Th.^387^ NFA **5-87** (IDT-4CN) with a RCN end group does not exceed 3.5% efficiency in solar cells with P3HT,^381,410^ but devices with PBDB-T reached values over 8%.^388^ The RCN end group has a positive impact on the PCE (4.4%, blend with J71) if compounds **5-88** (DTFBT-2) and **5-83** are compared (the latter contains a rhodanine end group without cyano modification, PCE 3.35%).^386^ Introducing electron-donating substituents on the rhodanine units as in structures **5-89** (IDT-2) and **5-90** (IDT-3) causes an increase of the optical band gap and an upshift of the HOMO/LUMO energies. Solar cells with P3HT give neglible PCE values.^389^ Fluorination of the BT spacer (**5-91**, H-FFBR, and **5-92**, O-FFBR) lowers the energy levels compared to **5-79**. Solar cells with PTB7-Th have similar PCE values (up to 9.4% in both cases).^390^ The Zhou research group has developed new acceptors based on a benzotriazole spacer (BTA series). Benzotriazole is less electron-withdrawing than the BT unit and if two molecules with the same residual structure are compared; e.g., the BTA-based NFA **5-95** (BTA3) has higher frontier orbital energies and larger band gaps than the analogous BT-based **5-87**. Similar permutations in the BTA-based acceptors are undertaken for BT-based compounds. Compound **5-94** (BTA2) with the less electron-withdrawing oxygenated rhodanine end groups has higher LUMO levels (thus, a higher band gap of 2.0 eV) than its sulfur analogue **5-93** (BTA1; 1.87 eV) or the RCN derivative **5-95** (1.76 eV), showing the influence of different end groups.^391^ Solar cells with all of these molecules with J61 reached very high *V*_OC_s ≥ 1.15 V and in the case of **5-95** PCEs up to 8.25%.^391^ Solar cells with the chlorinated donor J52-Cl reached a remarkable *V*_OC_ of 1.24 V together with a PCE of 10.5%.^386^ Fluorination of BTA (**5-96**, F-BTA3) minimally lowers the HOMO/LUMO energies; thus, the *V*_OC_ for solar cells based on P2F-EHp is reduced by 40 mV, but the *J*_SC_ and PCE are nearly doubled (8.38%) compared to the non-fluorinated **5-95** (PCE 4.62%).^393^ Replacing the ethyl substituent on the end group’s nitrogen of **5-95** with phenyl (**5-97**, BTA4) or benzyl (**5-98**, BTA5) has differing outcomes. The phenyl substituent (**5-97**) elevates the HOMO/LUMO energies and the optical band gap; thus, solar cells with J52-F as a donor have a higher *V*_OC_ than those of **5-95** but lower *J*_SC_, FF, and PCE values (**5-97** gives 8.38%, while **5-95** yields 9.04%). On the other hand, the additional methylene group between the nitrogen and the phenyl ring in **5-98** results in lower HOMO/LUMO energies and a lower optical band gap. Thus, solar cells with J52-F have a lower *V*_OC_, but all other parameters are improved, giving a higher PCE (11.3%). Compared to **5-95**, **5-98** has an enhanced face-on orientation in film with J52-F, while **5-97** has an amorphous stacking morphology.^394^ Replacing rhodanine-based end groups with a 4-cyano-styryl (**5-99**, BTA701) or cyanated furane-like end groups (**5-100**, BTA703) has a negative impact on the PCE values (blends with J71 reaching 0.07% for **5-99** and 1.21% for **5-100**).^395^ Replacing the hexylphenyl side chains in **5-95** with hexyloxyphenyl side chains on the IDT core, such as in **5-101** (BTA43), has a positive impact on the PCE values in solar cells with P3HT (6.56% vs 5.64% for **5-95**).^396^

**Table 12:** Optical, Electrical, and Photovoltaic Properties of Non-Fullerene Acceptors **5-75**–**5-115**

NFA	original name	HOMO^a^ (eV)	LUMO^a^ (eV)	*E*_g_^opt^ (eV)	donor	D:A ratio	*V*_OC_ (V)	*J*_SC_(mA cm^–2^)	FF (%)	PCE (%)	μ_e_^e^(cm^2^ V^–1^ s^–1^)	ref.
**5-75**	IDT-C8-BC	–5.39	–3.50	1.83	PBDB-T	1:1.5	1.05	10.3	68	7.3	-/3.0 × 10^–4^	(379)
**5-76**	IDT-BC	–5.50	–3.57	1.92	PBDB-T	1:1.5	1.08	10.5	56	6.3	-/1.4 × 10^–4^	(379)
**5-77**	O-IDTBCN	–5.70^c^	–4.18	1.52	PTB7-Th	1:2.5	0.72	19.8	73	11.1	2 × 10^–4^/2.2 × 10^–4^	(380)
**5-78**	IDT-CA	–5.59	–3.64	1.66	P3HT	1:0.8	0.84	7.77	64	4.19		(381)
**5-79**	O-IDTBR	–5.51	–3.55	1.63	PffBT2T-TT	1:1.5	1.08	14.3	67	10.4		(382)
**5-80**	EH-IDTBR				PffBT4T-2OD	1:1.5	1.04	14.5	63	9.5	-/4.4 × 10^–5^	(383)
**5-81**	IDT-BT-R	–5.30^d^	–3.59^d^		PTB7-Th	1:1.5	1.05	14.6	60	9.39	-/1.0 × 10^–4^	(384)
**5-82**	l-IDTBTRh	–5.42	–3.68	1.65	P3HT	1:0.8	0.86	8.81	71	5.38	-/1.2 × 10^–5^	(385)
**5-83**	DTFBT-1	–5.62	–3.82		J71	1:1	1.13	6.32	47	3.35	-/1.3 × 10^–4^	(386)
**5-84**	a-IDTBTRh	–5.63	–3.59	1.89	P3HT	0.8:1	0.92	5.00	55	2.53	-/8.8 × 10^–6^	(385)
**5-85**	*cis*-IDT-BT-R	–5.44	–3.62	1.64	PTB7-Th	1:1.5	1.08	13.8	58	8.02	-/1.0 × 10^–6^	(387)
**5-86**	*trans*-IDT-BT-R	–5.43	–3.61	1.63	PTB7-Th	1:1.3	1.05	15.1	60	9.43	-/2.2 × 10^–6^	(387)
**5-87**	IDT-4CN	–5.45	–3.95		PBDB-T	1:1.2	0.91	14.7	61	8.13	-/1.9 × 10^–4^	(388)
**5-88**	DTFBT-2	–5.76	–3.93		J71	1:1	0.94	8.35	56	4.40	-/3.1 × 10^–4^	(386)
**5-89**	IDT-2	–5.34	–3.49	1.65	P3HT	1:1	0.55	0.04	22	0.01	-/5 × 10^–9^	(389)
**5-90**	IDT-3	–5.28	–3.28	1.77	P3HT	1:1	0.68	0.29	25	0.05	-/6 × 10^–8^	(389)
**5-91**	H-FFBR	–5.54	–3.93^b^	1.61	PTB7-Th	1:1.5	0.94	16.9	57	9.1	-/3.4 × 10^–5^	(390)
**5-92**	O-FFBR	–5.59	–3.95^b^	1.64	PTB7-Th	1:1.5	0.94	17.0	59	9.4	-/5.7 × 10^–5^	(390)
**5-93**	BTA1	–5.46	–3.59	1.87	J61	1:1	1.24	5.21	47	3.02	-/5.4 × 10^–5^	(391)
**5-94**	BTA2	–5.43	–3.43	2.00	J61	1:1	1.29	0.84	24	0.26	-/2.4 × 10^–5^	(391)
**5-95**	BTA3	–5.49	–3.73	1.82	J52-Cl	1:1	1.24	13.2	67	10.5	-/2.4 × 10^–4^	(392)
**5-96**	F-BTA3	–5.59	–3.82^b^	1.77	P2F-EHp	1:1	1.25	11.3	59	8.38	-/1.5 × 10^–5^	(393)
**5-97**	BTA4	–5.50	–3.65	1.79	J52-F	1:1	1.21	8.39	55	5.61	-/1.3 × 10^–5^	(394)
**5-98**	BTA5	–5.55	–3.71	1.76	J52-F	1:1	1.17	13.8	70	11.3	-/3.4 × 10^–5^	(394)
**5-99**	BTA701	–5.27	–2.83	2.10	J71	1:1	1.32	0.21	25	0.07	-/2.5 × 10^–8^	(395)
**5-100**	BTA703	–5.50	–3.90	1.50	J71	1:0.5	0.85	3.56	40	1.21	-/1.1 × 10^–7^	(395)
**5-101**	BTA43	–5.44	–3.47	1.78	P3HT	1:0.5	0.89	10.8	68	6.56	-/3.2 × 10^–6^	(396)
**5-102**	BTA100	–5.32	–3.23	2.05	P3HT	1:1	1.34	1.65	47	1.04	-/6.9 × 10^–9^	(397)
**5-103**	BTA101	–5.41	–3.55	1.88	P3HT	1:1	1.19	5.33	56	3.55	-/1.4 × 10^–7^	(397)
**5-104**	BTA103	–5.37	–3.64	1.77	P3HT	1:2	0.94	8.56	66	5.31	-/3.5 × 10^–5^	(397)
**5-105**	A1	–5.66	–3.70	1.40	PTB7-Th	1.5:1	0.89	12.5	52	5.79	3.0 × 10^–4^/6.3 × 10^–5^	(398)
**5-106**	A2	–5.70	–3.78	1.36	PTB7-Th	1:2	0.70	20.8	63	9.07	7.5 × 10^–4^/2.3 × 10^–4^	(398)
**5-107**	JC1	–5.51	–3.95	1.30	P3HT	1:0.6	0.48	10.5	56	2.80	5.0 × 10^–4^/1.5 × 10^–4^	(399)
**5-108**	JC2	–5.48	–3.73	1.48	P3HT	1:0.8	0.71	14.0	63	6.24	4.1 × 10^–4^/4.5 × 10^–5^	(399)
**5-109**	A3	–5.60	–3.71	1.59	PTB7-Th	1:1.5	0.96	17.3	66	11.0	6.1 × 10^–4^/2.0 × 10^–4^	(400)
**5-110**	Qx1	–5.42	–3.60	1.74	P3HT	1:0.6	1.00	6.02	67	4.03	-/7.5 × 10^–7^	(401)
**5-111**	Qx1b	–5.35	–3.66	1.68	P3HT	0.8:1	0.95	7.34	69	4.81	-/1.1 × 10^–5^	(401)
**5-112**	Qx3	–5.38	–3.56	1.64	P3HT	1:0.5	0.89	5.57	68	3.37	-/4.2 × 10^–6^	(402)
**5-113**	Qx3b	–5.27	–3.63	1.59	P3HT	1:0.8	0.75	12.9	66	6.37	-/2.0 × 10^–5^	(402)
**5-114**	Qx3c	–5.30	–3.59	1.60	P3HT	1:1	0.75	0.14	30	0.03	-/1.3 × 10^–4^	(402)
**5-115**	IDT-FBTR	–5.47	–3.65	1.67	PTB7-Th	1:1.5	1.02	15.2	58	9.14	-/1.5 × 10^–4^	(403)

a Obtained from the oxidation/reduction potential of the CV measurement if not otherwise stated.

b HOMO/LUMO energy levels obtained from the LUMO/HOMO levels determined by CV and the optical band gap.

c HOMO obtained via PESA.

d Other method or method not defined.

e Determined via the SCLC technique from the neat acceptor/donor:acceptor blend films if not otherwise stated.

Three NFAs with dimethoxy-substituted BTAs and different end groups were prepared: **5-102** (BTA100) with 2,4-thiazolidinedione, **5-103** (BTA101) with rhodanine, and **5-104** (BTA103) with RCN.^397^ In this order, the optical band gaps were reduced to 2.05, 1.88, and 1.77 eV. Solar cells with compound **5-102**/P3HT gave a remarkable *V*_OC_ of 1.34 V with a PCE around 1%, but **5-104** had the highest PCE values up to 5.31% but a lower *V*_OC_ of 0.94 V.^397^

The introduction of thienobenzothiadiazole and thienobenzotriazole spacer units (spacer units 6 and 7, NFAs **5-105**–**5-109**) contributes to an extended conjugation length and strengthened intramolecular charge transfer due to an increased quinoid character. Benzothiadiazole derived molecules **5-105** (A1),^398^**5-106** (A2),^398^ and **5-107** (JC1)^399^ have lower band gaps (1.40, 1.36, and 1.30 eV, respectively) than the benzotriazole-based **5-108** (JC2)^399^ and **5-109** (A3)^400^ with 1.48 and 1.59 eV due to the stronger electron-withdrawing nature of benzothiazole. Solar cells with PTB7-Th resulted in PCE values of 11.0% using **5-109** due to a *V*_OC_ of 0.96 V, a FF of 66%, and a good *J*_SC_ of 17.3 mA cm^–2^ compared to those with **5-106** with a higher *J*_SC_ of 20.8 mA cm^–2^ but an overall lower PCE of 9.07%.^398,400^ Further representatives of π-spacers encompass the quinoxaline (π-spacer 9) and 2,3-diphenylquinoxaline (π-spacer 8) moiety. A number of acceptors were designed through the combination of different side chains (hexylphenyl or octyl) and rhodanine-based end groups (**5-110**–**5-114**). OCSs from blends with the P3HT give PCEs from 0.03 to 6.37% and *V*_OC_s in the range 0.75–1.00 V.^401,402^ Compound **5-115** (IDT-FBTR) has two spacers, fluoro-benzodithiophene and thiophene, leading to an A–D′–A′–D–A′–D′–A structure.^403^ This motif yields a PCE of 9.14% (blended with PTB7-Th).^403^

#### 4.2.2 Electron-Rich π-Spacers

The thiazole spacer unit in molecules **5-116** (H-IDTzR, hexyl side chains)^411^ and **5-117** (P-IDTzR, hexylphenyl side chains) acts as a non-covalent conformational lock, thus improving the planarity (due to the interactions between the S atom of the central core and the N atom of the spacer).^411^ The **5-116**/P3HT blend reaches PCE values of 3.53%, while **5-117** gave a slightly larger PCE of 5.01% (see Table 13). Replacement of the ethylrhodanine end group in **5-117** with INCN yields the structure **5-118** (DC-IDT2Tz) which reaches a PCE of 5.81% (blend with PTB7-Th).^412^

**Table 13:** Optical, Electrical, and Photovoltaic Properties of Non-Fullerene Acceptors **5-116**–**5-204**

NFA	original name	HOMO^a^ (eV)	LUMO^a^ (eV)	*E*_g_^opt^ (eV)	donor	D:A ratio	*V*_OC_ (V)	*J*_SC_(mA cm^–2^)	FF (%)	PCE (%)	μ_e_^e^(cm^2^ V^–1^ s^–1^)	ref.
**5-116**	H-IDTzR	–5.31^b^	–3.44	1.87	P3HT	1:1	1.04	6.67	53	3.53	-/1.8 × 10^–6^	(411)
**5-117**	P-IDTzR	–5.31^b^	–3.42	1.89	P3HT	1:1	1.02	9.00	55	5.01	-/7.1 × 10^–6^	(411)
**5-118**	DC-IDT2Tz	–5.63	–4.03^b^	1.60	PTB7-Th	1:1.5	0.82	10.8	66	5.81	-/5.0 × 10^–7^	(412)
**5-119**	T-TPT-T-2F	–5.50	–3.99	1.51	PBT1-C	1:1	0.93	17.6	67	10.7	7.7 × 10^–4^/1.2 × 10^–4^	(413)
**5-120**	IDTT2F	–5.57	–4.03	1.46	PBDB-T	1:1	0.81	18.5	59	8.85	-/3.1 × 10^–6^	(414)
**5-121**	IDT-T	–5.57	–4.01	1.56	PBDB-T	1:1	0.95	11.2	60	6.36	-/8.2 × 10^–6^	(415)
**5-122**	3	–5.47	–3.67	1.69	J61	1:1	0.92	8.09	43	3.17	-/7.5 × 10^–4^	(416)
**5-123**	4	–5.47	–3.72	1.59	J61	1:1	0.89	9.27	50	4.06	-/6.4 × 10^–4^	(416)
**5-124**	IDTOT2F	–5.54	–3.94	1.44	PBDB-T	1:1	0.85	20.9	72	12.8	-/4.0 × 10^–5^	(414)
**5-125**	IDTO-T-4F	–5.49	–3.88	1.45	PBDB-T	1:1.2	0.86	20.1	73	12.6	-/6.4 × 10^–5^	(417)
**5-126**	IDTO-Se-4F	–5.48	–3.90	1.40	PBDB-T	1:1.1	0.83	18.6	69	10.7	-/4.5 × 10^–5^	(417)
**5-127**	IDT2Se	–5.41	–3.87	1.45	PBDB-T	1:1	0.89	17.5	61	9.36	-/4.8 × 10^–5^	(418)
**5-128**	IDT2Se-4F	–5.51	–4.00	1.39	PBDB-T	1:1	0.79	21.5	66	11.2	-/1.6 × 10^–4^	(418)
**5-129**	IDT2SeC2C4	–5.30	–3.91	1.44	PBDB-T	1:1	0.88	17.2	59	8.92	1.3 × 10^–4^/6.5 × 10^–5^	(419)
**5-130**	IDT2SeC2C4-2F	–5.37	–3.99	1.40	PBDB-T	1:1	0.81	19.2	66	10.2	1.8 × 10^–4^/1.5 × 10^–4^	(419)
**5-131**	IDT2SeC2C4-4F	–5.40	–4.08	1.30	PBDB-T	1:1	0.77	22.0	62	10.6	2.1 × 10^–4^/1.7 × 10^–4^	(419)
**5-132**	IFIC	–5.11	–3.53	1.58	HFQx-T	1:1	1.02	11.2	55	6.28	-/9.9 × 10^–5^	(420)
**5-133**	IFIC-F	–5.43	–3.86	1.57	HFQx-T	1:1	0.95	12.0	51	5.87	-/1.1 × 10^–4^	(420)
**5-134**	ITMIC	–5.51^b^	–4.08	1.47	PM6	1:1	0.88	12.6	54	5.95	-/1.5 × 10^–4^	(421)
**5-135**	IEIC	–5.41^c^	–3.84^c^	1.57	PBDB-T	1:1	1.02	15.1	48	7.30	-/1.1 × 10^–4^	(422)
**5-136**	IE-4F	–5.51^c^	–4.07^c^	1.44	PBDB-T	1:1	0.87	21.4	58	10.8	-/3.0 × 10^–4^	(422)
**5-137**	IE-4Cl	–5.54^c^	–4.11^c^	1.43	PBDB-T	1:1	0.86	21.5	60	11.1	-/3.7 × 10^–4^	(422)
**5-138**	A1	–5.15	–3.73	1.55	J71	1:1	0.97	5.71	30	1.63	3.5 × 10^–5^/1.2 × 10^–5^	(144)
**5-139**	IDT-TN	–5.42	–4.03	1.43	PBDB-T	1:1	0.97	13.2	45	5.89	1.2 × 10^–6^/6.8 × 10^–5^	(423)
**5-140**	ERCN	–5.50	–3.59	1.82	P3HT	1:1.3	0.90	5.87	50	2.64	-/2.7 × 10^–5^	(424)
**5-141**	IDTP-P-C	–5.37	–3.72	1.42	PTB7-Th	1:1	0.79	18.8	55	8.21	7.2 × 10^–4^/8.6 × 10^–4^	(425)
**5-142**	IDTP-O-C	–5.43	–3.75	1.44	PTB7-Th	1:1	0.77	18.1	62	8.61	1.4 × 10^–3^/9.5 × 10^–4^	(425)
**5-143**	IDTCN-C	–5.59	–3.92	1.48	PBDB-T	1:1	0.84	20.3	70	11.9	-/9.5 × 10^–5^	(426)
**5-144**	*p*-IO1	–5.46	–4.13	1.34	PTB7-Th	1:1.5	0.78	22.3	62	10.8		(427)
**5-145**	*o*-IO1	–5.44	–4.15	1.28	PTB7-Th	1:1.5	0.74	26.3	67	13.1		(427)
**5-146**	*o*-IO2	–5.41	–4.21	1.20	PTB7-Th	1:1.5	0.68	21.8	63	9.3		(427)
**5-147**	*p*-IO2	–5.44	–4.19	1.24	PTB7-Th	1:1.5	0.70	23.0	67	10.8		(427)
	IEICO-4F	–5.43^d^	–4.03^d^		Si25-H2	1:1.5	0.70	26.9	70	13.2	1.2 × 10^–4^/9.2 × 10^–4^	(428)
**5-148**	IEICO-4Cl	–5.56	–4.23		D18	1:1.6	0.80	3.45	49	1.42		(136)
**5-149**	IOTIC-2F	–5.34	–4.06	1.31	PTB7-Th	1:1.5	0.82	21.9	65	12.1	-/2.1 × 10^–5^	(181)
**5-150**	IEICO	–5.32^d^	–3.95^d^		PTB7-Th	1:1.5	0.90	12.5	59	6.7	-/3.1 × 10^–4^	(429)
**5-151**	ORCN	–5.37	–3.56	1.64	P3HT	1:1.3	0.87	11.5	62	6.40	-/1.8 × 10^–4^	(424)
**5-152**	IDTP-P-O	–5.21	–3.75	1.28	PTB7-Th	1:1	0.73	19.1	59	8.17	3.7 × 10^–3^/2.6 × 10^–3^	(425)
**5-153**	IDTP-O-O	–5.27	–3.76	1.30	PTB7-Th	1:1	0.73	19.0	60	8.40	6.5 × 10^–3^/2.9 × 10^–3^	(425)
**5-154**	IDTCN-O	–5.54	–3.80	1.53	PBDB-T	1:1	0.91	20.0	73	13.3	-/7.6 × 10^–5^	(426)
**5-155**	IDTCN-S	–5.57	–3.90	1.48	PBDB-T	1:1	0.85	19.0	66	10.6	-/4.5 × 10^–5^	(426)
**5-156**	ACS8	–5.54	–4.05	1.30	PTB7-Th	1:2	0.75	25.3	69	13.2	2.7 × 10^–4^/2.0 × 10^–4^	(430)
**5-157**	A134	–5.54	–4.05		PTB7-Th	1:2	0.75	16.7	61	7.6		(275)
**5-158**	i-IEICO	–5.31	–3.68	1.60	J52	1:1	0.96	18.8	58	10.5	-/1.1 × 10^–4^	(431)
**5-159**	i-IEICO-2F	–5.29^b^	–3.73	1.58	J52	1:1	0.91	20.9	68	12.9	-/2.0 × 10^–4^	(432)
**5-160**	i-IEICO-F3	–5.34^b^	–3.74	1.59	J52	1:1	0.90	16.2	53	7.65	-/2.8 × 10^–5^	(432)
**5-161**	i-IEICO-4F			1.56	J52	1:1	0.85	22.9	68	13.2	-/3.8 × 10^–4^	(433)
**5-162**	i-cc23	–5.34	–3.64	1.69	PBDB-T	1:1	1.10	11.2	59	7.34	-/1.8 × 10^–5^	(434)
**5-163**	i-cc34	–5.39	–3.75	1.57	PBDB-T	1:1	0.96	15.7	63	9.51	-/1.2 × 10^–4^	(434)
**5-164**	i-mO-4F	–5.50	–3.81	1.55	PBDB-T	1:1	0.92	21.6	71	14.0	-/2.2 × 10^–4^	(435)
**5-165**	i-mO-4Cl	–5.55	–3.83	1.53	PBDB-T	1:1	0.87	15.1	56	7.41	-/5.1 × 10^–5^	(435)
**5-166**	IDT-OT	–5.11	–3.46		PBDB-T	1:1.5	0.93	8.27	43	3.32	-/1.1 × 10^–4^	(436)
**5-167**	IDTS-4F	–5.54	–3.93	1.43	PM6	1:1	0.89	21.0	70	12.9	-/3.1 × 10^–4^	(437)
**5-168**	IDT2ST-4F	–5.36	–3.71	1.43	PBDB-T	1:1	0.85	19.4	69	11.4	-/3.3 × 10^–5^	(438)
**5-169**	ITOIC	–5.48	–3.75	1.55	PBDB-T	1:1	1.02	15.7	55	8.87	2.5 × 10^–5^/2.4 × 10^–4^	(439)
**5-170**	ITOIC-F	–5.52	–3.82	1.50	PBDB-T	1:1	0.95	18.6	61	10.7	6.5 × 10^–5^/4.9 × 10^–4^	(439)
**5-171**	ITOIC-2F	–5.57	–3.87	1.45	PBDB-T	1:1.5	0.90	21.0	65	12.2	1.3 × 10^–4^/6.0 × 10^–4^	(439)
**5-172**	IDT-EDOT	–5.43	–3.80	1.41	PBDB-T	1:1.2	0.86	21.3	62	11.3	-/6.6 × 10^–5^	(440)
**5-173**	IDT-PDOT	–5.39	–3.77	1.43	PBDB-T	1:0.5	0.85	5.26	49	2.18	-/3.0 × 10^–6^	(440)
**5-174**	IDT-PDOT-C6	–5.59^c^	–4.17	1.42	PBDB-T	1:1	0.91	19.5	62	11.1	-/3.1 × 10^–4^	(441)
**5-175**	ITCIC	–5.59^b^	–4.12	1.43	PM6	1:1	0.86	20.5	64	11.3	-/4.3 × 10^–4^	(421)
**5-176**	IDT-3MT	–5.68	–4.16	1.52	PBDB-T	1:1	0.95	14.4	61	8.40	-/1.0 × 10^–5^	(415)
**5-177**	IDT-T_i_FIC	–5.57	–4.05	1.41	PBDB-T	1:1	0.86	17.0	65	9.46	-/2.1 × 10^–4^	(442)
**5-178**	IDT-T_o_FIC	–5.55	–3.86	1.50	PBDB-T	1:1	0.88	17.8	71	11.1	-/3.9 × 10^–4^	(442)
**5-179**	ITVT	–5.47	–3.59	1.48	PBZ	1:2	0.96	14.2	43	5.84	1.9 × 10^–4^/1.4 × 10^–4^	(443)
**5-180**	A401	–5.43	–3.53		PBDB-T	1:1	0.93	13.0	62	7.54	-/3.1 × 10^–6^	(444)
**5-181**	IDTO-TT-4F	–5.39	–3.89	1.38	PBDB-T	1:1.1	0.86	17.2	69	10.2	-/2.2 × 10^–5^	(417)
**5-182**	IDTC-4Cl	–5.50	–3.79	1.35	PBDB-T	1:1	0.82	19.2	60	9.50	2.8 × 10^–4^/2.5 × 10^–4^	(445)
**5-183**	4TIC	–5.36	–4.11	1.26	PTB7-Th	1:1.3	0.70	14.6	49	5.26	-/4.4 × 10^–4^	(446)
**5-184**	4T4F	–5.38	–4.13	1.22	PTB7-Th	1:1.3	0.60	17.3	60	6.58	-/4.8 × 10^–4^	(446)
**5-185**	6TIC	–5.31	–3.96	1.30	PTB7-Th	1:1.3	0.74	19.2	54	8.13	-/9.8 × 10^–4^	(446)
**5-186**	6T4F	–5.45	–4.00	1.23	PTB7-Th	1:1.3	0.60	24.9	69	10.7	-/1.3 × 10^–3^	(446)
**5-187**	ATT-5	–5.41	–3.60	1.50	PBDB-T	1:1	0.93	18.9	71	12.4	2.1 × 10^–4^/2.5 × 10^–4^	(447)
**5-188**	IFIC-i-2F	–5.42	–3.91	1.34	PTB7-Th	1:1.8	0.72	21.0	65	9.82	-/2.3 × 10^–4^	(448)
**5-189**	IFIC-i-6F	–5.31	–4.00	1.27	PTB7-Th	1:1.8	0.61	22.0	70	9.43	-/7.9 × 10^–4^	(448)
**5-190**	IFIC-i-4F	–5.34	–3.96	1.30	PTB7-Th	1:1.8	0.65	24.9	67	10.9	-/5.1 × 10^–4^	(448)
**5-191**	IFIC-*o*-4F	–5.36	–4.01	1.27	PTB7-Th	1:1.8	0.61	18.6	62	7.01	-/3.4 × 10^–5^	(448)
**5-192**	ATT-1	–5.51	–3.68	1.54	PBDB-T	1:1	0.92	16.4	60	9.00	8.6 × 10^–5^/0.1 × 10^–4^	(447)
**5-193**	ATT-4	–5.41	–3.62	1.49	PBDB-T	1:1	0.93	17.3	70	11.2	3.7 × 10^–4^/1.0 × 10^–4^	(447)
**5-194**	ATT-6	–5.48	–3.66	1.53	PBDB-T	1:1	0.96	14.7	59	8.39	-/2.4 × 10^–5^	(449)
**5-195**	ATT-7	–5.45	–3.61	1.54	PBDB-T	1:1	0.97	16.0	67	10.3	-/5.4 × 10^–5^	(449)
**5-196**	ATT-8	–5.46	–3.71	1.55	PBDB-T	1:1	0.80	13.9	65	7.31	-/1.9 × 10^–5^	(450)
**5-197**	TPD3	–5.65	–3.79	1.71	J52-Cl	1:1.5	0.96	12.7	62	7.58	-/1.1 × 10^–6^	(451)
**5-198**	TPD8	–5.60	–4.10^b^	1.46	PTQ10	1:1.5	0.91	18.2	63	10.4	-/1.3 × 10^–4^	(452)
**5-199**	IDBTC	–5.25	–3.54	1.57	P3HT		0.73	7.7	45	2.5		(453)
**5-200**	IDBTCF	–5.27	–3.55	1.56	P3HT		0.75	8.93	48	3.22		(453)
**5-201**	IDTBF	–5.64	–3.80	1.58	PM6	1:1	0.94	17.0	65	10.4	-/2.5 × 10^–4^	(454)
**5-202**	2FIFIC	–5.45	–3.97	1.38	PM6	1:1.4	0.80	22.4	62	11.3	-/8.0 × 10^–4^	(455)
**5-203**	ICIF2F	–5.45	–4.02	1.36	PM6	1:1.4	0.79	19.3	61	9.60	-/5.7 × 10^–4^	(455)
**5-204**	IDST-4F	–5.59	–4.01	1.41	PM6	1:1.25	0.82	24.9	70	14.3	-/4.3 × 10^–4^	(456)

a Obtained from the oxidation/reduction potential of the CV measurement if not otherwise stated.

b HOMO/LUMO energy levels obtained from the LUMO/HOMO levels determined by CV and the optical band gap.

c Obtained via ultraviolet photoelectron spectroscopy (UPS).

d Method not defined.

e Determined via the SCLC technique from the neat acceptor/donor:acceptor blend films if not otherwise stated.

Li et al. investigated IDTT analogues by replacing the terminal condensed thiophene units of the electron-donating IDTT core in structure **7-2** with thiophene as a π-bridging unit.^413^ In the case of one thiophene unit being replaced, an asymmetric six-ring system is created (see the section “Six Fused Aromatic Ring Systems”, **6-26**), while removal of the thiophene units from both sides yields the structure **5-119** (T-TPT-T-2F).^413^ The absorption spectrum of **5-119** is more red-shifted when compared to the parent seven-ring structure **7-2**. The LUMO level is influenced minimally, while the HOMO has a higher energy than in the fully fused molecule. Solar cells built from **5-119** with donor PBT1-C reached similar *V*_OC_ values than those with the six-ring analogue **6-26** or the seven-ring structure **7-2**. The overall photovoltaic performance was similar to the devices based on **7-2** with PCE values of 10.7% vs 10.5%, but both were outperformed by those of the asymmetric **6-26** with the highest *J*_SC_ and FF values leading to PCEs of 12.7%. Solar cells of **5-120**, which has INCN-2F end groups, reached PCE values of 8.85%,^414^ while solar cells of **5**-**121** (IDT-T), with INCN groups, had a PCE of 6.36% (both in blend with PBDB-T).^415^ Li et al. developed NFAs with new electron-withdrawing groups based on difluoroboron(III)β-diketonate.^416^ Energy levels, particularly the LUMOs of compounds **5-122** (3, with a *para*-CF_3_-phenyl group)^416^ and **5-123** (4, with a *para*-CN-phenyl group)^416^ are upshifted in comparison to compounds with INCN-based end groups (**5-119**–**5-121**); thus, also the optical band gaps are larger (1.69 eV/1.59 eV). Solar cells with J61 as a donor had moderate PCEs of 3.17% (**5-122**) and 4.06% (**5-123**).^416^ Modification of the IDT central core by the introduction of an additional alkoxy substituent on the terminal thiophene rings leads to **5-124** (IDTOT2F)^414^ and **5-125** (IDTO-T-4F),^417^ which differ from each other by the length of the alkyl chain (hexyl, octyl). Both compounds have similar optical band gaps, also compared to the unsubstituted **5-120**. Compound **5-124** has a better solubility than **5-120**, and it is more crystalline (face-on orientation, just as **5-125**). Solar cells of both compounds with PBDB-T gave increased *V*_OC_, *J*_SC_, as well as FF values compared to **5-120** and PCEs above 12.5%.^414,417^ Replacing the thiophene π-spacer in **5-125** with selenophene leads to structure **5-126** (IDTO-Se-4F)^417^ with a slightly decreased band gap of 1.40 eV. However, also the PCE values for solar cells with PBDB-T are lower (10.7%).^417^ Moreover, selenophene was introduced in several other IDT-based NFAs. Selenium increases the energy levels but reduces the optical band gap due to Se having a stabilizing influence on the LUMO. This can be seen by comparing **5-127** (IDT2Se; with an INCN end group and a band gap of 1.45 eV)^418^ and **5-128** (IDT2Se-4F; with INCN-2F, 1.39 eV)^418^ to their sulfur analogues **5-121** (INCN, 1.56 eV) and **5-120** (INCN-2F, 1.46 eV), respectively. Despite an increased LUMO energy, solar cells with the same donor polymer (PBDB-T) have a smaller *V*_OC_ than their sulfur analogues. At the same time, an enhanced *J*_SC_ and FF and a higher electron mobility enable higher efficiencies. Solar cells with **5-127** reached efficiencies of 9.36%, and devices based on the difluorinated analogue **5-128** revealed PCE values up to 11.2%.^418^ Similar observations are made for compounds in which the hexylphenyl side chains of the IDT core unit are replaced with 2-ethylhexyl groups, as shown in the compounds **5-129** (IDT2SeC2C4; INCN end group),^419^**5-130** (IDT2SeC2C4-2F; INCN-F),^419^ and **5-131** (IDT2SeC2C4-4F; INCN-2F).^419^ The optical band gaps are further reduced to 1.30 eV for **5-131**, and solar cells with PBDB-T as a donor gave similar PCE values as those of the hexylphenyl derivatives. Due to the different side chains on the central core, the furan-bridged molecules **5-132** (IFIC)^420^ and **5-133** (IFIC-F)^420^ cannot be directly compared to the thiophene- and selenophene-containing acceptors. Solar cells of these compounds with the donor HFQx-T, show high *V*_OC_s of approx. 1 V; however, due to the moderate *J*_SC_s and FFs, the PCEs are 6.28% for **5-132** and 5.87% for **5-133**.^420^ Adding a methyl group on the thiophene spacer, as realized in structure **5-134** (ITMIC),^421^ significantly improves the electron mobility in comparison to the compound without the methyl group (**5-120**), but the optical properties remain the same. In a blend with PM6, **5-134** reaches a PCE of 5.95%.^421^ Hong et al. introduced a series of NFAs with 2-ethylhexylthiophene as a π-spacer and INCN end groups (**5-135**, IEIC),^422^ INCN-2F (**5-136**, IE-4F),^422^ and INCN-2Cl (**5**-**137**, IE-4Cl)^422^ with decreasing optical band gaps (1.57, 1.44, and 1.43 eV). The alkyl substituent on the thiophene spacer has a positive effect on the performance in solar cells. Despite the relatively similar optical properties, the solar cells based on the 3-(2-ethylhexyl) derivatives **5-135** and **5-136** (polymer donor: PBDB-T) deliver higher PCE values than the thiophene derivatives **5-121** and **5-120**, 7.30% vs 6.36% for **5-135** and **5-121** (INCN groups) and 10.8% vs 8.85% for **5-136** and **5-120** (INCN-2F groups) due to the much higher electron mobility values.^415,422^ Solar cells with the chlorinated compound **5-137** reached the highest PCE (11.1%, blend with PBDB-T) due to even better electron mobility values.^422^ NFA **5-139** (IDT-TN)^423^ with extended INCN groups yields a lower electron mobility and weaker solar cell performance in blend with PBDB-T (PCE 5.89%).^423^ In structure **5-140** (ERCN),^424^ RCN end groups are used; in blend with P3HT, the material is yielding a moderate performance (2.64%).^424^ Also, the asymmetric IDT with an ethylhexylthiophene linker, one fullerene, and one INCN end group (**5-138**, A1) yields low PCE values (1.63% in a blend with J71).^144^ A further altnernative π-spacer is octylthiophene, present in the molecules **5-141** (IDTP-P-C)^425^ and **5-142** (IDTP-O-C).^425^ The combination with INCN-2F end groups leads to structure **5-141**, with very similar properties as the analogue compounds with thiophene (**5-120**), methylthiophene (**5-134**), and 2-ethylhexylthiophene (**5-136**). Solar cells with PTB7-Th yield a PCE of 8.21%.^425^ Replacing one hexylphenyl side chain of the IDT core with hexyloxy groups leads to the modified structure **5-142** (for the central core modification, see the upper right corner in Figure 16) which has a slightly better PCE (8.61%).^425^ Structure **5-143** (IDTCN-C)^426^ has a (4-hexyl)-thiophene spacer and INCN-2F end groups. Compared to the (3-octyl)-thiophene derivative (**5-141**), the optical band gap is slightly larger, but the electron mobility is 1 order of magnitude lower. Nevertheless, solar cells with PBDB-T reached a relatively good PCE of almost 12% with a high *J*_SC_ (20.3 mA cm^–2^) and FF (70%).^426^ Asymmetric compounds were prepared by using two different π-spacers simultaneously, 2-ethylhexylthiophene and 2-ethylhexyloxythiophene with INCN-2F end groups and either hexylphenyl (**5-144**, *p*-IO1) or octyl (**5-145**, *o*-IO1) side chains on the IDT core.^427^ Both compounds have similar HOMO and LUMO energy levels; however, since the octyl side chains allow closer molecular packing, compound **5-145** has a lower optical band gap (1.28 eV vs 1.34 eV). As a result, solar cells of **5-145** with PTB7-Th as a donor have a better *J*_SC_, FF, and PCE (13.1%) than those of **5-144** (PCE 10.8%). Interestingly, the symmetric molecules with two 3-(2-ethylhexyloxy)-thiophene spacers **5-146** (*o*-IO2; octyl chains on the IDT core) and **5-147** (*p*-IO2; with hexylphenyl groups on IDT) both exhibit even lower optical band gaps with values of 1.20 eV for **5-146** and 1.24 eV for **5-147**.^427^ Compound **5-146**, due to its symmetric nature, has a stronger crystallization tendency than the asymmetric **5-145**, which leads to a higher degree of phase separation in the absorber layer with PTB7-Th as a donor, causing a negative influence on exciton dissociation, thus lowering the PCE (9.3%). The hexylphenyl derivative (**5-147**, IEICO-4F) shows an improved phase separation; i.e., the domain size is reduced compared to **5-146**. As a result, solar cells with PTB7-Th yield an improved performance with PCE values up to 10.8% by the same authors^427^ and up to 12.6% by Corzo et al.^457^ NFA **5-147** has a low optical band gap (1.24 eV) and a comparably good electron mobility (10^–4^ cm^2^ V^–1^ s^–1^), ensuring a good *J*_SC_ (typically over 20 mA cm^–2^) and FF (60–70% range). The highest PCE values of 13.2% were obtained in devices with Si25-H2 as donor.^428^ The INCN-2Cl analogue **5-148** (IEICO-4Cl) reached only a mediocre performance of 1.35% in solar cells with D18.^136^ Using the INCN-F group instead leads to **5-149** (IOTIC-2F) with an increased optical band gap due to the higher lying LUMO energy level. Thus, solar cells with PTB7-Th achieve higher *V*_OC_ values compared to the before discussed acceptors and PCE values went up to 12.1%.^181^ The non-halogenated INCN compound **5-150** (IEICO) has a larger optical band gap, as expected. Solar cells with various donor polymers gave only moderate PCE values, e.g., with J52 a value of 5.13%^431^ and with PTB7-Th values between 6.0 and 6.7%,^429,458^ while **5-151** (ORCN) with RCN-based end groups reached a similar PCE with P3HT (6.40%).^424^ A further, structurally similar, π-spacer is octyloxythiophene (spacer unit 20), which is used in compounds **5-152** (IDTP-P-O) and **5-153** (IDTP-O-O). Both compounds in blend with PTB7-Th have lower efficiencies (>8.4%) than those of **5-147** (ethylhexyloxythiophene spacer derivatives), with the same donor (PCE above 10%).^425^ Compound **5-154** has a 4-(hexyloxy)-thiophene spacer (spacer unit 21 in Figure 16); thus, the alkoxy group is on the far side of the IDT unit in contrast to the octyloxy group in **5-152** or the 2-ethylhexyloxy unit in **5-147**. Compared to the latter two, **5-154** (IDTCN-O) has a much larger optical band gap with a value of 1.53 eV. As expected, solar cells of **5-154** with PBDB-T exhibit a higher *V*_OC_ of 0.91 V and a PCE of 13.3%.^426^ Replacing oxygen with sulfur, i.e., using (hexylthio)thiophene, the structure **5-155** (IDTCN-S) with a slightly lower band gap (1.48 eV) is obtained. Solar cells with PBDB-T show a lower performance (PCE of 10.6%) than those of the hexyloxythiophene derivative, probably due to the lower electron mobility.^426^ Another example of a sulfur-containing π-spacer is the 3-(2-ethylhexylthio)-thiophene spacer unit 23 (see Figure 16) which is the sulphur analogue to 3-(2-ethylhexyloxy)-thiophene (spacer unit 19). The NFA structure **5-156** (ACS8) is the sulfur analogue to **5-147**, comprising INCN-2F groups, and has a slightly higher band gap of 1.30 eV. Solar cells with PTB7-Th are showing an increased *J*_SC_ without reducing other solar cell parameters (compared to **5-147**), and thus delivers a better PCE of 13.2%.^430^ The PCE values drop to 7.6% in solar cells of PTB7-Th/NFA **5-157** (A134), in which the hexylphenyl side chains are replaced with hexyl groups, mainly due to a much lower *J*_SC_.^275^ Structures **5-158** (i-IEICO),^431^**5-159** (i-IEICO-2F),^432^ and **5-161** (i-IEICO-4F)^433^ use the 2-(2-ethylhexyloxythiophene) linker with INCN, INCN-F, and INCN-2F, but these acceptor end groups are attached at the 4-position of the thiophene ring and thus these compounds are regioisomers to compounds **5-150**, **5-149**, and **5-147**, respectively. All of the 4-isomers have larger band gaps and thus larger *V*_OC_ values than the 5-isomers in solar cells. In combination with J52, **5-158** reached a *V*_OC_ of 0.96 V and a PCE of 10.5%, which is twice as high as the efficiencies of devices with the regioisomer **5-150**.^431^ Solar cells of **5-159** (INCN-F groups) showed increased PCE values from 11.3^459^ to 12.9%,^432^ and further, those of **5-161** with the INCN-2F groups get even higher up to 13.8%, also exceeding the performance achieved with the isomer **5-147**.^433^ Electrochemical and optical properties of **5-160** (i-IEICO-F3),^432^ in which the fluorine atom is located *ortho* to the carbonyl group of the INCN moiety, are nearly identical to its regioisomer **5-159**. However, solar cells with the donor J52 have a lower performance (PCE of 7.65%), which might be due to the 1 order of magnitude reduced electron mobility.^432^ Structures **5-162** (i-cc23) and **5-163** (i-cc34) comprise the thiophene modifications (end group **r** and **s** in Figure 16) of the INCN end groups. Solar cells of the 3,4-fused thiophene end group (i-cc34) outperformed its 2,3-fused isomer i-cc23 (PCE 9.51% vs 7.34%, blends with PBDB-T).^434^ Compounds **5-164** (i-mO-4F) have *meta*-hexyloxyphenyl side chains on the IDT core and INCN-2F end groups and, thus, can be seen as modifications of structures **5-161** with *para*-hexylphenyl side chains on the core. Both acceptors have nearly identical optical band gaps, and also, the solar cell parameters are very similar (albeit with different donors). Blends of **5-164** with PBDB-T show a high *J*_SC_ (21.6 mA cm^–2^) and an excellent FF (71%) and consequently reached a PCE of 14.0%.^435^ In acceptor **5-165** (i-mO-4Cl), the INCN-2F end group is replaced with INCN-2Cl units, which has a negative influence on solar cells with the same donor (PCE: 7.41%).^435^ Acceptor **5-166** (IDT-OT) contains 3,4-dimethoxythiophene spacer units, but its blends with PBDB-T did not reach high efficiencies (3.32%).^436^ Better results are obtained with **5-167** (IDTS-4F) with the same π-spacer but fluorinated end groups and oxygen replaced with sulfur in the central core side chains, i.e., *para*-hexylthiophenyl groups. Solar cells using a blend with the donor polymers PM6 or PM7 give efficiencies over 12%.^437^**5-168** (IDT2ST-4F) contains a 3,4-di(octylthio)thiophene π-spacer (spacer unit 26) and INCN-2F groups.^438^ Oxygen analogous compounds having a 3,4-di(hexyloxy)thiophene π-spacer (spacer unit 27) are represented by the structures **5-169** (ITOIC) with INCN, **5-170** (ITOIC-F) with INCN-F, and **5-171** (ITOIC-2F) with INCN-2F groups.^439^ As expected, fluorination leads to lower frontier orbital energies, especially on the LUMO and thus to lower band gaps decreasing with the fluorine content from 1.55 eV for **5-169** to 1.50 eV for **5-170** and 1.45 eV for **5-171**. Consequently, solar cells of these compounds with PBDB-T show an expected decrease in *V*_OC_ values in this order but an increase in *J*_SC_ and FF, thus making the devices with the INCN-2F groups **5-171** the most efficient ones, reaching PCEs of 12.2%.^439^ The sulfur analogue **5-168** has an even lower band gap of 1.40 eV, and thus, solar cells with PBDB-T gave even lower *V*_OC_ values, but due to a FF of 69% and a *J*_SC_ of 19.4 mA cm^–2^, they reached almost the same PCE values (11.4%).^438^ It is interesting to compare **5-171** with compounds **5-172** (IDT-EDOT), **5-173** (IDT-PDOT),^440^ and **5-174** (IDT-PDOT-C6)^441^ all having 3,4-alkoxy substituents on the thiophene unit. However, the latter three in different cyclic forms, i.e., **5-172**, contain the classical ethylenedioxythiophene (EDOT) as a spacer, **5-173** propylenedioxythiophene, and **5-174** additional exocyclic hexyl side chains on the propylenedioxy bridge. The optical band gaps of all of these compounds are nearly identical (between 1.41 and 1.45 eV), and in solar cells, also the *V*_OC_ values (with PBDB-T donor) do not differ significantly (0.88 ± 0.03 V). Whereas also the other solar cell parameters of **5-172** and **5-174** are very similar to the overall PCE values of 11.3 and 11.1%, respectively, the PCE of devices with **5-173** is significantly smaller (2.18%). The 3,4-propylenedioxythiophene derived molecule (**5-173**) has a lower molar absorption coefficient in solution than its 3,4-ethylenedioxythiophene-based counterpart **5-172**; also, the π–π stacking of **5-172** was determined to be stronger. This might be the reason for the improved electron mobility, *J*_SC_, and FF.^440^ At the same time, adding two hexyl chains as in **5-174** again increases the electron mobility (this compound has a face-on orientation in film).^441^ The π-spacers used in acceptors **5-175** (ITCIC)^421^ and **5-176** (IDT-3MT)^415^ differ from other thiophene-based spacers reviewed so far as being the only ones with an electron-withdrawing substituent. **5-175** has a 2-chlorothiophene linker, which can form non-covalent interactions with sulfur atoms of the IDT core. This interaction improves the planarity of the molecule, which has a positive impact on the electron mobility. In comparison to the methyl-substituted analogue **5-134**, which has nearly identical optoelectronic parameters, solar cells of **5-175**/PM6 have an improved *J*_SC_, FF, and PCE (11.3% vs 5.95% for **5-134**).^421^**5-176** has an ester group in the 3-position of the thiophene linker, and when compared to **5-121** (with the unsubstituted thiophene linker IDT-T), the former yields a higher PCE (8.40% vs 6.36%) in blend with PBDB-T.^415^ Ming et al. attached (5-hexylthiophen-2-yl) groups to the thiophene π-bridge, once in the 3-position, i.e., pointing toward the IDT core (“inner position”, **5-177**, IDT-T_i_FIC), and once in the 4-position, pointing toward the end group (“outer position”, **5-178**, IDT-T_o_FIC). The outer isomer (**5-178**) is slightly better with a PCE of 11.1% (blend with PBDB-T).^442^ Linking the two thiophene molecules with a double bond as in **5-179** (ITVT) leads to a lower PCE of 5.84% (PBZ as donor).^443^ Also, the acceptor **5-180** (A401) with four INCN accepting units attached to a terthiophene branching unit (see spacer unit 36 in Figure 16) did not lead to a high PCE in solar cells with PBDB-T (7.54%).^444^ Compound **5-181** (IDTO-TT-4F) comprises thienothiophene spacer units. This modification leads to a slightly reduced PCE (10.2%, in blend with PBDB-T) if compared to a simple thiophene linked molecule (**5-125**, 10.7%).^417^ The cyclopentadithiophene π-bridge present in acceptor **5-182** (IDTC-4Cl) with INCN-2Cl groups shows relatively good absorption properties expanding until 900 nm, and thus, solar cells with PBDB-T have relatively high *J*_SC_ values of 19.2 mA cm^–2^. However, the *V*_OC_ of 0.82 V and FF of 60% do not allow a PCE over 9.5%.^445^ Alternative π-spacer units are thieno[3,4-*b*]thiophene bridges (spacer units 39–44) and have been introduced in the NFA structures **5-183**–**5-196**. NFAs **5-183** (4TIC) and **5-184** (4T4F) combine an IDT core with central octyl chains with INCN and INCN-2F units, respectively, bridged with 2-ethylhexyloxycarbonyl-substituted thienothiophene spacers. Both compounds absorb into the near infrared regions with absorption maxima in thin films at 881 and 920 nm, respectively. Similar values are observed also for the thienothiophene π-spacer isomer (spacer unit 40) containing **5-185** (6TIC) and **5-186** (6T4F). Solar cells of the INCN-based compounds **5-185** and **5-183** with PBDB-T have, as expected, higher *V*_OC_ but lower *J*_SC_ values than the difluorinated compounds. Devices with **5-185** and **5-186** (comprising the spacer unit 40) exhibit higher *J*_SC_ values, with **5-186** outperforming **5-185**. The excellent electron mobility in combination with the red-shifted absorption maximum gives a high *J*_SC_ of 24.9 mA cm^–2^ and a FF of 69%, resulting in a PCE of 10.7%.^446^ In addition to the change from octyl to hexyl groups on the IDT core, the INCN group has been replaced with RCN acceptor units in compound **5-187** (ATT-5).^447^ These changes lead to a blue-shift in the absorption maximum. Solar cells with PBDB-T reached a high *V*_OC_ above 0.9 V and a good *J*_SC_ and FF, leading to an overall PCE up to 12.4%.^447^ Compounds **5-188**–**5-191** have an additional fluorine on the π-spacer and combine hexylphenyl-IDT cores with INCN (**5-188**, IFIC-i-2F), INCN-2F (**5-189**, IFIC-i-6F), as well as INCN-F (**5-190**, IFIC-i-4F). Additionally, **5-191** (IFIC-*o*-4F) is the regioisomer of **5-190** by changing the thienothiophene orientation as discussed before. These compounds have higher optical band gaps in thin films than their non-halogenated analogous thienothiophenes; however, this effect may partly arise from the bulkier hexylphenyl side chains on the IDT core hindering closer packing. Solar cells with PTB7-Th show very similar efficiency values: 9.82% for **5-188**, 9.43% for **5-189**, 10.9% for **5-190**, and 7.01% for **5-191**.^448^ By linking RCN acceptor units with IDT cores with different side chains via an octyloxycarbonyl-substituted thienothiophene spacer (43), NFA structures **5-192** (ATT-1)^447^ with *para*-hexylphenyl side chains, **5-193** (ATT-4)^447^ with hexyl side chains, **5-194** (ATT-6)^449^ with *meta*-hexylphenyl groups, and **5-195** (ATT-7)^449^ with *meta*-hexyloxyphenyl groups have been introduced. The optical band gaps are within values between 1.49 and 1.54 eV, resulting in high *V*_OC_s around 0.95 V of solar cells with PBDB-T. Replacing *p*-hexylphenyl side chains on the IDT core (**5-192**) with its *meta* isomer (**5-194**) reduced the PCE from 9.00 to 8.39%, while the corresponding *m*-hexyloxyphenyl side chains (**5-195**) improved the PCE to 10.3%.^449^ However, the derivative with hexyl side chains **5-193** leads to the highest PCE of 11.2%.^447^ These results again emphasize the impact of the side chain modification on the film morphology and thus on the solar cell performance. Acceptor **5-196** contains a trifluoromethyl substitution on the thieno[3,4-*b*]thiophene bridge (spacer unit 44) and has very similar optical properties to the aforementioned ones; however, solar cells with PBDB-T show only moderate efficiencies of 7.3% mainly due to a lower *V*_OC_.^450^

The imide functionality in 5-octyl-4*H*-thieno[3,4-*c*]pyrrole-4,6(5*H*)-dione (π-spacer 45) makes this spacer unit more electron-withdrawing. In structures **5-197** (TPD3)^451^ and **5-198** (TPD8),^452^ this π-spacer is used to combine hexylphenyl-IDT with either RCN^451^ or INCN^452^ end groups, respectively. Compound **5-198** in blend with PTQ10 reached good PCE values of 10.4%,^452^ while **5-197** in blends with J52-Cl gave smaller PCE values (7.58%).^451^ A thiophene–benzothiadiazole–thiophene bridge (spacer unit 46) and the fluorinated derivative (spacer unit 47) were incorporated between the IDT core and the RCN end group to give the structures **5-199** (IDBTC) and **5-200** (IDBTCF). However, these combinations of D–A–D building blocks in the π-bridging unit partly counteract with each other; thus, solar cells with P3HT reach moderate PCE values up to 2.5 and 3.22%, respectively.^453^

Asymmetric acceptors **5-201**–**5-204** were prepared by using only one π-spacer combined with an acceptor unit (spacer units 48-51) and using an INCN end group without a spacer on the other side. Structure **5-201** (IDTBF) was prepared by end-capping the IDT core with a INCN-2F group on one side and a BT π-spacer with a rhodanine end group (spacer unit 48) on the other side.^454^ This molecule can be roughly compared to O-IDTBR (**5-79**), the symmetric acceptor with two BT π-spacers and rhodanine end groups. The maximum PCE obtained with PM6 is 10.4%, and it is in the same performance range as the solar cells of **5-79** with different donors (see discussion above). Acceptors **5-202** (2FIFIC) and **5-203** (ICIF2F) differ in their end groups; in one case, fluorinated INCN is attached directly to the IDT core and a non-modified INCN group is attached to the 2-ethylhexyloxycarbonyl modified fluorothienothiophene spacer (**5-202**), while, in the other case, the end groups are exchanged (**5-203**). Solar cells of **5-202** with PM6 have higher PCE values than those with **5-203** (11.0% vs 9.42%).^455^

However, the best results are achieved by the molecule **5-204** (IDST-4F), which contains a 3-(2-ethylhexyl)thiothiophene π-spacer between one of the INCN-2F end groups and the IDT core. Solar cells of **5-204** with PM6 reach *J*_SC_s and FFs of almost 25 mA cm^–2^ and 70%, leading to PCE values of 14.3%.^456^

### 4.3 Miscellaneous Rings

**Figure 17:**
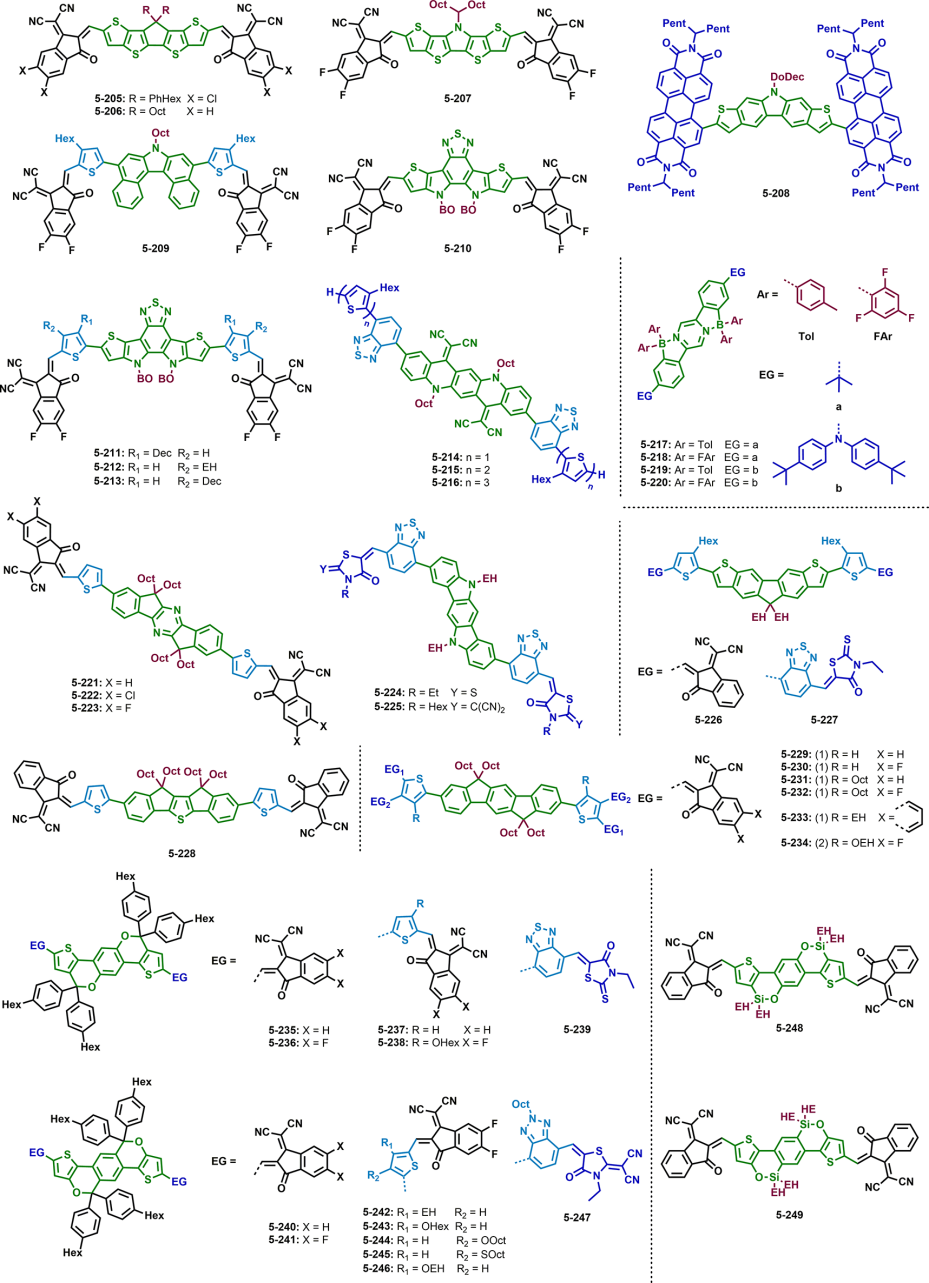
Structures of non-fullerene acceptors with miscellaneous five-ring core units and varying side chains, end groups, and π-spacers.

In the last three years, also multiple other fused-ring cores have been prepared and tested in solar cells (Figure 17, Table 14). Some are based on well-known structural motifs, while others are completely new. Many of these pioneering structures still are lacking in efficiency; however, such initial material design is essential for the development of OPVs and might bring further breakthroughs.

**Table 14:** Optical, Electrical, and Photovoltaic Properties of Non-Fullerene Acceptors **5-205**–**5-249**

NFA	original name	HOMO^a^ (eV)	LUMO^a^ (eV)	*E*_g_^opt^ (eV)	donor	D:A ratio	*V*_OC_ (V)	*J*_SC_(mA cm^–2^)	FF (%)	PCE (%)	μ_e_^d^(cm^2^ V^–1^ s^–1^)	ref.
**5-205**	CC5	–5.75	–4.09	1.37	PM6	1:0.7	0.65	18.0	60	6.91	-/5.2 × 10^–5^	(460)
**5-206**	CDTTIC	–5.62	–3.98	1.44	PFBDB-T	1:1	0.78	25.8	57	11.5	4.2 × 10^–3^/3.5 × 10^–4^	(461)
**5-207**	DTP-C_17_-4F	–5.59	–4.16	1.38	PM6	1:1	0.69	21.2	61	8.94	-/3.5 × 10^–3^	(462)
**5-208**	DTC-PDI	–5.39	–3.88	1.69	PTB7-Th	1:1.25	0.90	7.38	39	2.63	-/2.2 × 10^–5^	(463)
**5-209**	DCB-4F	–5.50	–3.86	1.55	PM6	1:1	1.00	16.4	58	9.56	-/1.3 × 10^–4^	(464)
**5-210**	BTPT-4F	–5.73	–4.00	1.45	P2F-EHp	1:1.2	0.78	3.20	44	1.09		(213)
**5-211**	H1	–5.39	–3.90	1.22	PBDB-T	1:1.2	0.78	16.8	53	6.98	1.1 × 10^–4^/1.0 × 10^–4^	(465)
**5-212**	H2	–5.38	–3.90	1.22	PBDB-T	1:1.2	0.78	24.4	69	13.2	2.9 × 10^–3^/3.5 × 10^–4^	(465)
**5-213**	H3	–5.40	–3.92	1.22	PBDB-T	1:1.2	0.76	25.8	70	13.8	3.3 × 10^–3^/4.2 × 10^–4^	(465)
**5-214**	DCNQA-BT-T1	–5.36	–3.69	1.81	P3HT	1:1	0.51	3.75	38	0.79		(466)
**5-215**	DCNQA-BT-T2	–5.31	–3.70	1.80	P3HT	1:1	0.51	1.60	28	0.31		(466)
**5-216**	DCNQA-BT-T3	–5.16	–3.65	1.79	P3HT	1:1	0.50	1.21	33	0.28		(466)
**5-217**	1-Tol	–6.01	–3.71	2.38	PTB7-Th	1:1	0.96	1.4	31	0.4		(467)
**5-218**	1-Far	–6.24	–3.88	2.27	PTB7-Th	1:1	0.87	5.4	40	1.9		(467)
**5-219**	2-Tol	–5.13		1.64	PTB7-Th	1:1	0.89	0.8	27	0.2		(467)
**5-220**	2-Far	–5.18	–3.66	1.48	PTB7-Th	1:1	0.79	1.2	31	0.3		(467)
**5-221**	IPY-T-IC	–5.69	–3.50	1.90	PTB7-Th	1:2	0.83	16.5	56	7.68	-/1.9 × 10^–4^	(468)
**5-222**	IPY-T-ICCl	–5.72	–3.56	1.75	PTB7-Th	1:1.5	0.69	14.1	64	6.20	-/1.1 × 10^–4^	(468)
**5-223**	IPY-T-ICF	–5.71	–3.53	1.82	PTB7-Th	1:1.5	0.71	13.5	60	5.81	-/1.1 × 10^–4^	(468)
**5-224**	ICz-Rd_2_	–5.59	–3.88^b^	1.71	P	1:1	1.04	14.0	54	7.88	1.2 × 10^–4^/-	(469)
**5-225**	ICz-RdCN_2_	–5.58	–3.98^b^	1.61	P	1:1	1.01	15.6	62	9.76	2.9 × 10^–4^/-	(469)
**5-226**	DICTFDT	–5.56	–3.89^b^	1.67	PTB7-Th	1:1.5	0.89	12.5	46	5.12	-/2.6 × 10^–5^	(470)
**5-227**	TFDTBR	–5.46	–3.77^b^	1.69	PTB7-Th	2:3	0.92	12.3	35	3.95	-/3.5 × 10^–6^	(470)
**5-228**	IDIDT-C8	–5.50	–3.86	1.64	PBDB-T	1:1.5	0.97	15.8	66	10.1	-/2.4 × 10^–5^	(471)
**5-229**	ICBF	–5.64	–3.80	1.80	PBDB-T	1:1	0.99	10.3	60	6.07		(472)
**5-230**	FICBF	–5.66	–3.88	1.72	PBDB-T	1:1	0.87	12.7	67	7.41		(472)
**5-231**	ICBF-O	–5.70	–3.75	1.90	PBDB-T	1:1	1.06	6.82	43	3.11		(472)
**5-232**	FICBF-O	–5.72	–3.85	1.81	PBDB-T	1:1	0.96	10.6	55	5.54		(472)
**5-233**	IF-TN	–5.80	–3.93	1.81	PBDB-T	1:1	1.01	7.02	41	3.03	4.1 × 10^–7^/1.0 × 10^–5^	(423)
**5-234**	i-IF-4F	–5.55	–3.71	1.79	PTB7-Th	1:1.5	0.87	12.5	60	6.47	-/1.3 × 10^–5^	(473)
**5-235**	PTIC	–5.67	–3.85	1.55	PBDB-T	1:1	0.84	14.2	64	7.66	-/4.9 × 10^–5^	(474)
**5-236**	CO*i*5DFIC	–5.96^c^	–4.29^c^	1.56	PTB7-Th	1:1.5	0.59	14.4	53	4.5		(307)
**5-237**	PTTIC	–5.48	–4.04	1.53	PBDB-T	1:1	0.93	14.3	55	7.35		(475)
**5-238**	*Ph*-DTDP_*i*_-OT	–5.55	–3.90	1.51	PBDB-T	1:1	0.90	18.7	68	11.4	-/8.8 × 10^–5^	(476)
**5-239**	PTBT-R	–5.59	–3.70	1.64	PBDB-T	1:1	0.97	10.4	50	5.06		(475)
**5-240**	*i*-PTIC	–5.41^b^	–3.86	1.55	PBDB-T	1:1	0.83	11.0	65	5.80		(477)
**5-241**	*i*-PTIC-F	–5.66^b^	–4.18	1.48	PBDB-T	1:1	0.63	14.6	62	5.68		(477)
**5-242**	*Ph*-DTDP_*o*_-TE	–5.63	–3.96	1.43	PBDB-T	1:1	0.82	20.8	71	12.2	-/1.0 × 10^–4^	(478)
**5-243**	*Ph*-DTDP_*o*_-OT	–5.50	–3.87	1.46	PBDB-T	1:1	0.87	14.8	59	7.60	-/1.9 × 10^–5^	(476)
**5-244**	CO5DFIC-OT	–5.51	–3.95	1.28	PTB7-Th	1:1.5	0.71	17.6	61	7.66	-/8.6 × 10^–6^	(479)
**5-245**	CO5DFIC-ST	–5.55	–3.92	1.34	PTB7-Th	1:2.5	0.74	20.7	64	9.73	-/2.0 × 10^–5^	(479)
**5-246**	*Ph*-DTDP_*o*_-OTE	–5.52	–3.88	1.46	PBDB-T	1:1	0.88	18.3	69	11.0	-/4.1 × 10^–5^	(478)
**5-247**	BTA53	–5.48	–3.58	1.68	P3HT	1:0.8	0.88	11.6	62	6.31	-/1.8 × 10^–6^	(396)
**5-248**	SiOTC	–5.60	–3.81	1.61	PM6	1:1.2	0.81	5.06	48	1.96	-/5.3 × 10^–6^	(480)
**5-249**	SiOTIC	–5.57	–3.84	1.55	PM6	1:1.2	0.92	14.5	75	10.0	-/1.3 × 10^–4^	(480)

a Obtained from the oxidation/reduction potential of the CV measurement if not otherwise stated.

b HOMO/LUMO energy levels obtained from the LUMO/HOMO levels determined by CV and the optical band gap.

c Other method or method not defined.

d Determined via the SCLC technique from the neat acceptor/donor:acceptor blend films if not otherwise stated.

Structure **5-205** (CC5)^460^ combines a di(hexylphenyl)-cyclopentadithienothiophene (CDTT) core with two INCN-2Cl units and exhibits a smaller optical band gap of 1.37 eV and lower lying HOMO and LUMO levels than the structurally similar **5-206** (CDTTIC),^461^ combining a dioctyl-CDTT core with an INCN end group exhibiting an optical band gap of 1.44 eV. The as-cast device built up from a blend of PFBDB-T/**5-206** delivered a very good PCE of 11.5% and a *J*_SC_ of 25.8 mA cm^–2^.^461^ The fusion of pyrrole and thienothiophenes leads to pentacyclic *S*,*N*-heteroacene compounds, for example, to structure **5-207** (DTP-C_17_-4F) with an optical band gap of 1.38 eV. A high *J*_SC_ of 21.2 mA cm^–2^ was obtained for solar cells with PM6 as a donor (PCE 8.94%).^462^

Structure **5-208** (DTC-PDI) consists of an electron-rich pentacyclic di(benzothiophene)carbazole core and perylene diimides as end groups, but blends with PTB7-Th lacked in *J*_SC_ and FF, yielding only a PCE of 2.60%.^463^ NFA **5-209** (DCB-4F) also comprises a carbazole as the central unit but flanked by two benzenes on the second and fourth rings of this central core. INCN-2F end groups are attached via a hexylthiophene π-spacer. The acceptor has an optical band gap of 1.55 eV, and its solar cells with PM6 reach a high *V*_OC_ (1.00 V) and an overall PCE of 9.56%.^464^ Moreover, several acceptors contain a fused (D–A′–D) core, which is formed via fusion of an electron-deficient BT to two thienopyrrole units. Consequently, a prolonged conjugation length combined with a good delocalization of π-electrons is achieved. The combination of this core with INCN-2F end groups leads to structure **5-210** (BTPT-4F) having low lying energy levels and an optical band gap of 1.45 eV.^213^ Solar cells with the donor P2F-EHp yielded only a PCE of 1.09%, which is a hard contrast to the seven-ring analogue **7-145** (BTPTT-4F/Y6) which achieved a PCE of 16.0%. The partly edge-on orientation of **5-210** in the active layer being unfavorable for charge transfer could be the reason for the limited performance of **5-210** in solar cells. The incorporation of an additional alkylthiophene π-spacer between the dithienopyrrolobenzothiadiazole core and the INCN-2F end groups leads to the structures **5-211** (H1), **5-212** (H2), and **5-213** (H3) with narrow band gaps of 1.22 eV.^465^ The three structures differ in their alkyl substitution on the thiophene linker; i.e., **5-211** has a decyl group in the 3-position (pointing toward the core), whereas **5-212** and **5-213** have 2-ethylhexyl and decyl chains in the 4-position (pointing toward INCN). All three compounds have nearly identical electrochemical and optical properties, and blends with PBDB-T have good electron mobilities (**5-213** > **5-212** > **5-211**). As expected, solar cells of these compounds with PBDB-T have nearly the same *V*_OC_; however, the *J*_SC_ values are different following the trend of the mobility data. **5-211** has the smallest *J*_SC_ with 16.8 mA cm^–2^, **5-212** reached 22.4 mA cm^–2^, while **5-213**-based devices revealed even 25.8 mA cm^–2^. Consequently, solar cells with the latter yield the highest PCE value (13.8%). Also, in this case, **5-211** had an edge-on orientation in neat film, **5-212** a mixed edge-on and face-on orientation, and the best performing NFA, **5-213**, a face-on orientation.^465^ Quinacridone-based acceptors **5-214**–**5-216** (DCNQA-BT-Tx) are interesting due to their reversed structure; e.g., the electron-rich thiophenes are used as end groups. However, solar cells with P3HT as a donor gave only PCE values below 1% in all cases.^466^

Morgan et al. introduced boron-containing NFAs by the reaction of different 2,5-diphenylpyrazine derivatives with BCl_3_ and subsequent substitution of the chlorides with further aryl groups (structures **5-217**–**5-220**).^467^ The best solar cells were assembled from **5-218** which, blended with PTB7-Th, gave a *V*_OC_ of 0.87 V and a *J*_SC_ of 5.4 mA cm^–2^, reaching a PCE of 1.9%; the solar cells of the other three compounds reached PCEs <1%.^467^ Acceptors **5-221**–**5-223** contain a 6,12-dihydrodiindeno[1,2-*b*:1′,2′-*e*]pyrazine as a core linked via a thiophene π-spacer to INCN-based end groups, i.e., INCN (**5-221**, IPY-T-IC), INCN-2Cl (**5-222**, IPY-T-ICCl), and INCN-2F (**5-223**, IPY-T-ICF).^468^ Structure **5-221** has the highest LUMO, and thus, also its solar cells with PTB7-Th as a donor yielded a *V*_OC_ (0.83 V) higher than the solar cells of its halogenated analogues. Combined with a *J*_SC_ of 16.5 mA cm^–2^, this results in the highest PCE values obtained in this series (7.68%).^468^ Suman et al. prepared acceptor structures by combining a 5,11-dihydroindolo[3,2-*b*]carbazole core with a BT spacer and the rhodanine (**5-224**, ICz-Rd_2_) or the RCN (**5-225**, ICz-RdCN_2_) end group. The latter had a better performance in solar cells, with polymer P giving a PCE of 9.76%.^469^ Fluorene fused with thiophene was introduced as an alternative electron-donating core in combination with a hexylthiophene π-spacer and INCN termini (**5-226**, DICTFDT) and with an additional benzothiadiazole π-spacer combined with a rhodanine end group (**5-227**, TFDTBR). The former yielded a better PCE (5.12%, in PTB7-Th).^470^ Structure **5-228** comprises a thiophene-based core, linked to INCN end groups by thiophene π-bridges. Rather untypical is the *cis* arrangement of the alkyl side chains of the central core. Solar cells prepared with PBDB-T revealed a PCE of 10.1%.^471^ Structures **5-229**–**5-234** contain a 6,12-dihydroindeno[1,2-*b*]fluorene core linked via different thiophene spacer units and INCN-based end groups. The lack of heteroatoms in the central core makes these compounds slightly less electron-rich; thus, the optical band gaps are rather high (between 1.72 and 1.90 eV). Structure **5-229** (ICBF) comprises the unmodified INCN and a simple thiophene linker. Solar cells in combination with PBDB-T achieved PCE values up to 6.07%, which were improved to 7.41% when INCN-2F end groups were used (**5-230**, FICBF). The introduction of an octyl substituent on the thiophene spacer leads to the derivatives **5-231** (ICBF-O) and **5-232** (FICBF-O). The planarity of the molecules is slightly reduced, whereby the optical band gaps increase and both compounds show lower performances in solar cells with the same donor.^472^ The efficiency is not improved with the compound **5-233**, which has a 2-ethylhexyl chain on the linker and extended INCN end groups (PCEs 3.03% with PBDB-T).^423^ The electron-donating strength of the thiophene spacer was increased by alkoxy substitution as in compound **5-234** (i-IF-4F). Solar cells with PTB7-Th yielded a PCE of 6.47%.^473^

A further class of NFAs is based on 4,10-dihydrothieno[2′,3′:4,5]pyrano[2,3-*g*]thieno[3,2-*c*]chromene as a central core. Here, a thiophene–benzene–thiophene structure is additionally linked by (oxymethylene) bridges. The combination of this central core with different end groups gives the structures **5-235** (PTIC) with INCN^474^ and **5-236** (CO*i*5DFIC) with INCN-2F^307^ accepting groups. Solar cells with **5-235** in blends with PBDB-T had a higher *V*_OC_ (0.94 V, consequence of higher LUMO energy), improved FF, and a better overall PCE of 7.66%^474^ compared to **5-236**-based devices. When compared to its higher analogue **7-309**,^307^**5-236** has lowered energy levels, leading also to a lower *V*_OC_ (0.59 V) and PCE (4.5%) in solar cells with the polymer donor PTB7-Th.^307^ A variation of this acceptor motif is represented in structure **5-237** (PTTIC) in which the core and the INCN group are linked via a thiophene linker, leading to a slightly lower optical band gap of 1.53 eV. Upshifted energy levels lead to a higher *V*_OC_ (0.93 V) and a PCE of 7.35% in solar cells with PBDB-T as a donor.^475^ The combination with a hexyloxythiophene π-spacer and INCN-2F groups leads to structure **5-238** (*Ph*-DTDP_*i*_-OT) with an even lower optical band gap of 1.51 eV. As expected, solar cells with PBDB-T showed a slightly lower *V*_OC_, but due to much higher values for the *J*_SC_ (18.7 mA cm^–2^) and FF (68%), the devices reached a promising PCE of 11.4%.^476^ The combination of this core with rhodanine end groups linked by a BT bridge gives molecule **5-239** (PTBT-R), with the highest optical band gap in this series. Thus, solar cells with PBDB-T have a good *V*_OC_ of 0.97 V, but due to reduced *J*_SC_, the PCE is smaller (5.06%).^475^ Changing the oxygen and carbon positions in the central core leads to the isomeric 5,11-dihydrothieno[2′,3′:5,6]pyrano[3,4-*g*]thieno[3,2-*c*]isochromene donor unit, used for building the acceptors **5-240**–**5-247**. The acceptor molecule **5-240** (*i*-PTIC) is the isomeric compound to **5-235**, but in solar cells, **5-240** has a lower PCE (5.80% vs 7.66%), which is only slightly improved if INCN-2F end groups are used (compound **5-241**, PCE 5.86% with PBDB-T).^477^ Blends of **5-241** with PTB7-Th yield a similar PCE (5.58%).^479^ The introduction of a thiophene-based π-spacer between the core and INCN-2F groups has a positive influence on the photovoltaic performance. Structure **5-242** with ethylhexylthiophene spacers has a reduced optical band gap of 1.43 eV, and solar cells with PBDB-T reached the best PCE of this compound class with a value of 12.2%.^478^ The change to ethylhexyloxythiophene, i.e., the introduction of an additional oxygen, leads to the acceptor structure **5-246** (*Ph*-DTDP_*o*_-OTE) with a slightly larger band gap of 1.46 eV, thus giving slightly higher *V*_OC_ values in solar cells with the same donor. However, due to a lower *J*_SC_, the PCE is reduced to 11.0%.^478^ The hexyloxythiophene derivative **5-243** (*Ph*-DTDP_*o*_-OT) and the octyloxythiophene derivative **5-244** (CO5DFIC-OT) showed a lower performance in solar cells, reaching PCEs of 7.6% in both cases (**5-243** with PBDB-T,^476,478^**5-244** with PTB7-Th^479^). Exchanging oxygen in compound **5-244** with sulfur using the 3-(octylthio)-thiophene spacer gives **5-245** (CO5DFIC-ST), which showed a good performance in solar cells with PTB7-Th of almost 10%.^479^ Using BTA as a π-spacer and RCN end groups yields NFA **5-247** (BTA53). Due to the electron-withdrawing character of BTA, the HOMO and LUMO energies are shifted upward, and the optical band gap is increased to 1.68 eV (in comparison to the thiophene-spacer-containing compounds). Solar cells with P3HT achieved a PCE of 6.31%.^396^ In compounds **5-248** (SiOTC) and **5-249** (SiOTIC), the sp^3^-hybridized carbons of the central core are replaced by Si(EH)_2_ groups. The inner isomer **5-248** has a slightly higher band gap than the outer isomer **5-249** (1.61 eV vs 1.55 eV), and the HOMO and LUMO energy levels are also quite similar. Nevertheless, solar cells of **5-249** with PM6 show a much better performance with a PCE reaching 10.0%, while those of **5-248** are below 2%. The better performance of **5-249** was explained by the much higher electron mobility and improved blend morphology, which had a positive impact on *J*_SC_ and FF.^480^

## 5 Six Fused Aromatic Ring Systems

**Figure 18:**
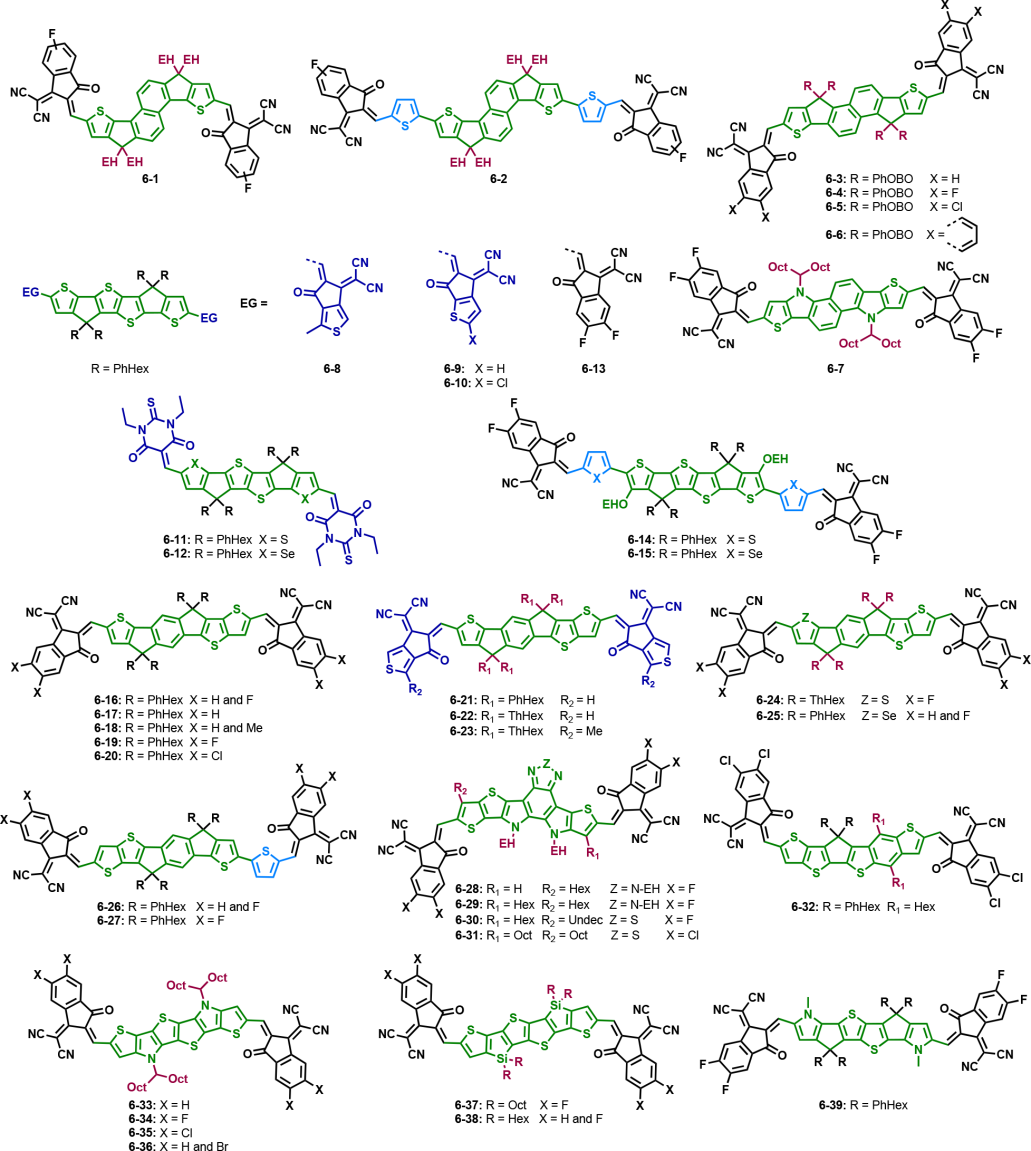
Structures of non-fullerene acceptors with six fused rings.

The hexacyclic fused-ring electron acceptors reported since 2018 can be assigned to four main groups based on the structural features they are containing. The characteristic structural unit of the first group is an angular shaped dithienonaphthalene (DTN) core. A typical representative of this group is **6-1** (DTNIF), exhibiting an optical band gap of 1.63 eV and reaching PCEs of 8.73% with PBDB-T (Figure 18, Table 15).^481^ The incorporation of a thiophene spacer into the backbone of this molecule results in **6-2** (DTNSF) and leads to a reduced optical band gap (1.47 eV). However, overall, this modification resulted in a reduced PCE (7.15%) compared to **6-1** due to a significantly decreased FF.^481^

**Table 15:** Optical, Electrical, and Photovoltaic Properties of the Non-Fullerene Acceptors Containing a Six-Fused-Ring Structural Feature

NFA	original name	HOMO^a^ (eV)	LUMO^a^ (eV)	*E*_g_^opt^ (eV)	donor	D:A ratio	*V*_OC_ (V)	*J*_SC_(mA cm^–2^)	FF (%)	PCE (%)	μ_e_^d^(cm^2^ V^–1^ s^–1^)	ref.
**6-1**	DTNIF	–5.82	–3.92	1.63	PBDB-T	1:1	0.90	13.3	73	8.73	-/1.8 × 10^–5^	(481)
**6-2**	DTNSF	–5.52	–4.00	1.47	PBDB-T	1:1	0.92	14.5	55	7.15	-/6.7 × 10^–6^	(481)
**6-3**	O-NTIC	–5.51	–3.67	1.70	PBDB-T	1:0.8	0.98	13.3	70	9.1	-/8.3 × 10^–5^	(482)
**6-4**	NT-4F	–5.88	–3.86	1.68	PM6	1:1.2	0.96	13.9	71	9.46	-/3.3 × 10^–4^	(278)
**6-5**	NT-4Cl	–5.89	–3.92	1.64	PM6	1:1.2	0.93	16.6	74	11.4	-/7.8 × 10^–4^	(278)
**6-6**	O-NTNC	–5.54	–3.78	1.64	PBDB-T	1:0.8	0.94	16.0	73	11.0	-/1.7 × 10^–4^	(482)
**6-7**	TITI-4F	–5.81^c^	–4.25^c^	1.56	PM6	1:1	0.91	16.0	75	10.9	-/3.5 × 10^–4^	(483)
**6-8**	T6Me	–5.50	–3.94	1.38	PM6	1:1	0.87	21.3	65	12.1	-/6.4 × 10^–4^	(292)
**6-9**	4TTIC	–5.46	–3.73	1.46	PBDB-ST	1:1	0.93	18.6	67	11.5	-/3.5 × 10^–4^	(484)
**6-10**	4TTIC-Cl	–5.50	–3.79	1.42	PBDB-ST	1:1	0.88	20.2	74	13.1	-/4.2 × 10^–4^	(484)
**6-11**	4TBA	–5.51	–3.70^b^	1.66	PBDT-2TC	1:1	0.96	15.4	71	10.5	-/3.9 × 10^–5^	(485)
**6-12**	STBA	–5.45	–3.60^b^	1.59								(485)
**6-13**	F6IC	–5.68	–4.04	1.36	PTB7-Th	1:1.2	0.61	18.2	63	7.00	9.1 × 10^–4^/9.1 × 10^–5^	(486)
**6-14**	4TO-T-4F	–5.30	–3.85	1.30	PTB7-Th	1:1.5	0.75	20.4	58	8.87	-/4.0 × 10^–5^	(487)
**6-15**	4TO-Se-4F	–5.29	–3.85	1.27	PTB7-Th	1:1.5	0.70	19.1	55	7.40	-/2.1 × 10^–5^	(487)
**6-16**	TPTT-2F	–5.75	–4.04	1.58	PBT1-C	1:1	0.88	15.8	73	10.2	4.5 × 10^–4^/1.6 × 10^–4^	(299)
**6-17**	A201	–5.69	–3.93	1.64	J71	1:1	0.88	13.2	67	9.36	-/5.2 × 10^–4^	(488)
	ITIC-3T	–5.54	–3.93^b^	1.61	PBDTBDD-Ph	1:1	0.92	17.5	68	11.0	-/1.6 × 10^–4^	(329)
**6-18**	IDT6CN-M	–5.62	–3.90	1.63	PBDB-T	1:1	0.92	16.0	76	11.2	9.0 × 10^–4^/5.9 × 10^–4^	(489)
**6-19**	T-TT-4F	–5.44	–3.51	1.58	PM6	1:1	0.86	18.5	66	10.5	-/4.3 × 10^–4^	(490)
**6-20**	T-TT-4Cl	–5.48	–3.59	1.53	PM6	1:1	0.81	19.0	66	10.2	-/3.7 × 10^–4^	(490)
**6-21**	IDT6CN	–5.68	–3.97	1.63	PBDB-T	1:1	0.83	15.1	74	9.27	7.7 × 10^–4^/6.3 × 10^–4^	(378)
**6-22**	IDT6CN-Th	–5.71	–4.01	1.61	PBDB-T	1:1	0.81	16.8	77	10.4	9.0 × 10^–4^/7.1 × 10^–4^	(378)
**6-23**	IDT6CN-TM	–5.70	–3.96	1.60	PM6	1:1	0.95	17.4	75	12.4	8.3 × 10^–4^/-	(491)
**6-24**	IDT6CN-4F	–5.78	–4.12	1.58	PM6	1:1	0.86	18.3	69	10.9	7.6 × 10^–4^/-	(491)
**6-25**	SePTT-2F	–5.71	–4.00	1.50	PBT1-C	1:0.9	0.83	17.5	75	10.9	5.1 × 10^–4^/2.5 × 10^–4^	(300)
**6-26**	TTPT-T-2F	–5.60	–4.00	1.54	PBT1-C	1:1.5	0.92	18.5	75	12.7	6.6 × 10^–4^/3.6 × 10^–4^	(413)
**6-27**	TTPT-T-4F	–5.63	–4.08	1.45	L2	1:1.5	0.86	22.2	74	14.0	3.6 × 10^–4^/3.4 × 10^–4^	(492)
**6-28**	Y21	–5.65	–3.90	1.35	PM6	1:1	0.83	24.9	74	15.4	-/1.3 × 10^–3^	(493)
**6-29**	Y22	–5.69	–3.94	1.36	PM6	1:1.2	0.85	24.4	74	15.4	-/1.0 × 10^–3^	(494)
**6-30**	BP-4F	–5.68	–3.88		PM7	1:1.2	0.88	21.7	76	14.6	-/3.3 × 10^–5^	(495)
**6-31**	TB-4Cl	–5.7	–4.1		PM6		0.85	23.0	75	14.7	-/5.8 × 10^–4^	(496)
**6-32**	CC10	–5.72	–4.07	1.38	PM6	1:1.2	0.77	22.7	67	11.8	-/1.0 × 10^–4^	(460)
**6-33**	SN61C	–5.38	–3.93	1.39	PBDB-T	1:1	0.88	16.5	66	9.6	-/3.0 × 10^–4^	(497)
**6-34**	SN61C-4F	–5.52	–4.11	1.32	PBDB-T	1:1	0.78	23.2	73	13.2	-/5.0 × 10^–4^	(497)
**6-35**	PTTtID-Cl	–5.53	–4.08		PTB7-Th	1:1.6	0.71	19.9	60	8.5		(498)
**6-36**	SN6-2Br	–5.39	–3.86	1.32	PBDB-T		0.73	19.4	71	10.0	-/4.9 × 10^–4^	(499)
**6-37**	NFDTS	–5.36	–3.92	1.41	PTB7-Th	1:1.2	0.67	18.8	49	6.15	-/3.9 × 10^–6^	(303)
**6-38**	Si4TIC-F	–5.58	–4.21	1.37	PBTIBDTT	1:1.5	0.78	18.8	69	10.2	2.3 × 10^–4^/1.3 × 10^–5^	(500)
**6-39**	P6IC	–5.43	–3.94	1.30	PTB7-Th	1:1	0.69	25.0	70	12.2	8.8 × 10^–4^/1.9 × 10^–4^	(501)

a Obtained from the oxidation/reduction potential of the CV measurement if not otherwise stated.

b HOMO/LUMO energy levels obtained from the LUMO/HOMO levels determined by CV and the optical band gap.

c Obtained via UPS.

d Determined via the SCLC technique from the neat acceptor/donor:acceptor blend films if not otherwise stated.

Feng et al. used non-fluorinated INCN units and solubilizing *para*-alkoxy-phenyl side chains leading to structure **6-3** (O-NTIC),^482^ which has a slightly increased band gap compared to the phenylhexyl-substituted analogue IHIC1.^502^ By combining this donor unit with INCN-2F (**6-4**, NT-4F) and INCN-2Cl (**6-5**, NT-4Cl) end groups, NFAs with lower optical band gaps and downshifted energy levels are obtained. With PM6/**6-4**-based solar cells, PCEs of 9.46% are reported; PM6/**6-5** absorber layers lead to PCEs of 11.4%.^278^ Through extending the end groups of **6-3** from INCN to the benzannulated derivative 2-(3-oxo-2,3-dihydroinden-1-ylidene)malononitrile, a more ordered packing could be achieved in **6-6** (O-NTNC), which resulted in a significantly red-shifted absorption and increased electron mobility. Furthermore, an enhancement of the PCE of the respective solar cells (blend with PBDB-T) from 9.1 to 11.0% was obtained. Exchanging the bridging atom in the donor unit of **6-2** from carbon to nitrogen leads to **6-7** (TITI-4F). This acceptor, in combination with PM6, reached PCEs up to 10.9%.^483^

A further structural modification similar to the IDT unit was implemented by exchanging the central benzene ring with a thienothiophene moiety to give the donor unit T-Cp-T-T-Cp-T.^503^ Using CPTCN-Me end groups gives **6-8** (T6Me).^292^ The extension of the backbone conjugation compared to IDT-based NFAs such as, e.g., **5-73** causes, as expected, also a red-shift of the absorption with an optical band gap of 1.38 eV and an absorption maximum in thin film of 820 nm. Based on their energy levels, **6-8** and PM6 are well suited to be combined in the absorber layer and solar cells based on this material combination revealed high PCEs up to 12.1%. Chlorination of the CPTCN end group (**6-9**, 4TTIC, and **6-10**, 4TTIC-Cl) could increase the solar cell efficiency from 11.5% (PBDB-ST/**6-9**) to 13.1% (PBDB-ST/**6-10**).^484^ The NFAs **6-11** (4TBA) and **6-12** (STBA) contain TBA derived end groups, and in **6-12**, the outer thiophene units are replaced by selenophenes. These compounds reveal an optical band gap of 1.66 and 1.59 eV, respectively. Combined with the polymer PBDT-2TC, **6-11** shows a *V*_OC_ of 0.96 V and a PCE of 10.5%.^485^**6-13** (F6IC) consists of the T-Cp-T-T-Cp-T core unit with INCN-2F end groups and reveals an optical band gap of 1.36 eV, very similar to **6-8**. For PTB7-Th/**6-13**-based solar cells, PCEs up to 7.00% are reported.^486,504^ Thiophene or selenophene π-spacers combined with additional alkoxy side chains on the peripheral thiophene rings of the central core yield very narrow band gap materials **6-14** (4TO-T-4F, 1.30 eV) and **6-15** (4TO-Se-4F, 1.27 eV).^487^ Solar cells prepared in combination with PTB7-Th had good *J*_SC_ values, but PCEs did not exceed 8.87% (for compound **6-14**).^487^

The second group of recently investigated six-fused-ring NFAs (**6-16**–**6-24**) contains the asymmetric moiety thieno[1,2-*b*]indaceno[5,6-*b*′]thienothiophene (TITT) as a donor unit (T-Cp-B-Cp-T-T). TITT in turn is obtained from a fusion of the IDT unit with an additional thiophene moiety. Asymmetric structures have the advantage of bearing higher dipole moments, which facilitates (i) intramolecular charge transfer in the A–D–A structure leading to a higher charge carrier mobility as well as (ii) the self-assembly of the molecules involving enhanced lamellar packing and π–π stacking.^488,489,505−507^ Moreover, a broader absorption range and an increased LUMO energy level are expected.^299^ A series of such molecules with different INCN acceptor units (INCN-F, **6-16** (TPTT-2F);^299^ INCN, **6-17** (A201^488^/ITIC-3T);^329^ monomethylated INCN, **6-18** (IDT6CN-M);^378,489^ INCN-2F, **6-19** (T-TT-4F);^490,508^ INCN-2Cl, **6-20** (T-TT-4Cl)^490^) are reported. Using different donor polymers, solar cells yielded relatively similar PCEs around 9–11%. These structures are also closely related to the seven-ring ITIC with the only difference that one of the two thienothiophene groups in the fused ITIC backbone is replaced by a thiophene unit, making the acceptor asymmetric and shorter. The shorter structure is discussed to possess advantages in forming beneficial morphologies in combination with certain weakly aggregated conjugated polymers, which could also be proved by an increased PCE.^329^ As a further variation of this structure, the terminal INCN was substituted by a CPTCN unit, resulting in **6-21** (IDT6CN), and an additional substitution of the hexylphenyl residue with thienyl side chains (5-hexylthiophen-2-yl) can facilitate the π–π stacking of the side chains in **6-22** (IDT6CN-Th).^378,491^ Using PBDB-T as a donor polymer, solar cells of **6-21** and **6-22** exhibit lower *V*_OC_ values and thus lower PCEs (9.27 and 10.4%) than those of **6-18** (11.2%) with the same donor polymer. The NFA **6-23** (IDT6CN-TM)^491^ bears additional methyl groups at the termini and was compared to **6-24** (IDT6CN-4F) with INCN-2F end groups. A comparison of solar cells based on **6-23** and **6-24** with PM6 reveals a change in PCE from 12.4 to 10.9%.^491^ This enhanced performance of **6-23** is ascribed to the higher lying LUMO level, the higher dipole moment, and the slightly higher electron mobility compared to **6-24**, which is beneficial for the *V*_OC_ and the FF of the solar cells (cf. Table 15).

Replacing the singular thiophene unit in the donor core of **6-16** with a selenophene unit leads to **6-25** (SePTT-2F).^300^ Compared to the sulfur analogue **6-16**,^299^ the optical band gap is reduced from 1.58 to 1.50 eV. In combination with the donor polymer PBT1-C, the PCE is improved from 10.2 to 10.9%. **6-26** (TTPT-T-2F) is a further modification of **6-16** by introducing a thiophene as the π-spacer.^413^ Solar cells of **6-26** with the donor polymer PBT1-C showed a balanced charge mobility and higher PCEs of 12.7%^413^ compared to those of **6-16** with the same donor (cf. Table 15). With **6-27** (TTPT-T-4F), the analogue of **6-26** with INCN-2F end groups, PCEs of 14.0% are obtained in combination with polymer L2.^492^

The NFAs **6-28**–**6-31** are modifications of the Y-series NFAs with one fused thiophene less on one end of the core, leading to an asymmetric molecular geometry. **6-28** (Y21) and **6-29** (Y22) contain a BTA unit instead of the commonly used BT group. Compared to **6-28**, **6-29** bears only one additional hexyl side chain hardly affecting the optical and photovoltaic properties. Both NFAs have a narrow band gap of ∼1.35 eV and exhibit a PCE of 15.4% (polymer PM6).^493,494^**6-30** (BP-4F) and **6-31** (TB-4Cl) contain INCN-2F and INCN-2Cl end groups, respectively, and can be additionally distinguished by their different alkyl side chains. In solar cells, PCEs of 14.6% (PM7/**6-30**) and 14.7% (PM6/**6-31**) are obtained.^495,496^

The asymmetric NFA **6-32** (CC10)^460^ comprises a donor core in a T-T-Cp-T-B-T motif. Compared to the symmetric five-ring structure T-T-Cp-T-T (**5-205**), this asymmetric acceptor leads to better π–π stacking, enhanced electron mobility and morphology, and also less trap assisted recombination. Overall, this is reflected in a simultaneous increase of *V*_OC_, *J*_SC_, and FF, resulting in a significant enhancement of the photovoltaic performance in solar cells with PM6 from 6.91% (PM6/**5-205**) to 11.8% (PM6:**6-32**).

The donor units of structures **6-33**–**6-36** replace the two cyclopentadiene units with *N*-alkylated pyrroles, leading to the symmetric motif T-P-T-T-P-T.^497^ The aromatic pyrrole ring can thereby enhance the conjugation and has a higher electron-donating character, leading to increased energy levels. The combination with INCN and INCN-2F acceptor units leads to the structures **6-33** (SN61C) and **6-34** (SN61C-4F), both introduced by Huang et al.,^497^ and with INCN-2Cl to **6-35** (PTTtID-Cl), published by Wang et al.^498^**6-33** and **6-34** were evaluated in solar cells with the donor PBDB-T. The lower band gap significantly increased the *J*_SC_ values of the devices based on difluorinated **6-34**. Together with a higher fill factor, PCE values reach remarkable 13.2% compared to 9.6% for those of **6-33**. In **6-35**, the fluorinated INCN are replaced by INCN-2Cl units, giving a compound with rather similar HOMO and LUMO levels (see Table 15). However, the solar cell performance cannot be fully compared, as **6-35** was tested (i) in combination with PTB7-Th, whereby a PCE of 8.5% was obtained, and (ii) in a ternary solar cell architecture in combination with the donor PM7 and IT-4F (**7-2**) as a second acceptor, resulting in a PCE of 12.0%. **6-36** (SN6-2Br) contains brominated INCN end groups and reveals the same optical band gap (1.32 eV) as the fluorinated analogue (**6-34**); the performance in solar cells is, however, lower. For PBDB-T/**6-36**-based solar cells, PCEs of 10.0% are obtained.^499^

The replacement of the pyrrole rings with silole rings leads to the bisdithienosilole-based NFAs **6-37** (NFDTS) and **6-38** (Si4TIC-F). **6-37** in solar cells with PTB7-Th gave a photovoltaic performance of 6.15%,^303^ while the devices prepared from **6-38** with the conjugated polymer PBTIBDTT reveal a PCE of 10.2%.^500^ Lu et al. compared NFA **6-13**, consisting of the donor unit T-Cp-T-T-Cp-T and INCN-2F units, with structure **6-39** (P6IC) where the two outermost thiophenes of the donor unit are replaced by *N*-alkylated pyrroles.^501^ Solar cells of the pyrrole compounds in combination with PTB7-Th reach PCE values up to 12.2% compared to only moderate 7.00% for **6-13**. This was mainly ascribed to a narrower band gap, an enhanced crystallinity and electron mobility, as well as a slightly upshifted LUMO energy level.

## 6 Eight Fused Aromatic Ring Systems

**Figure 19:**
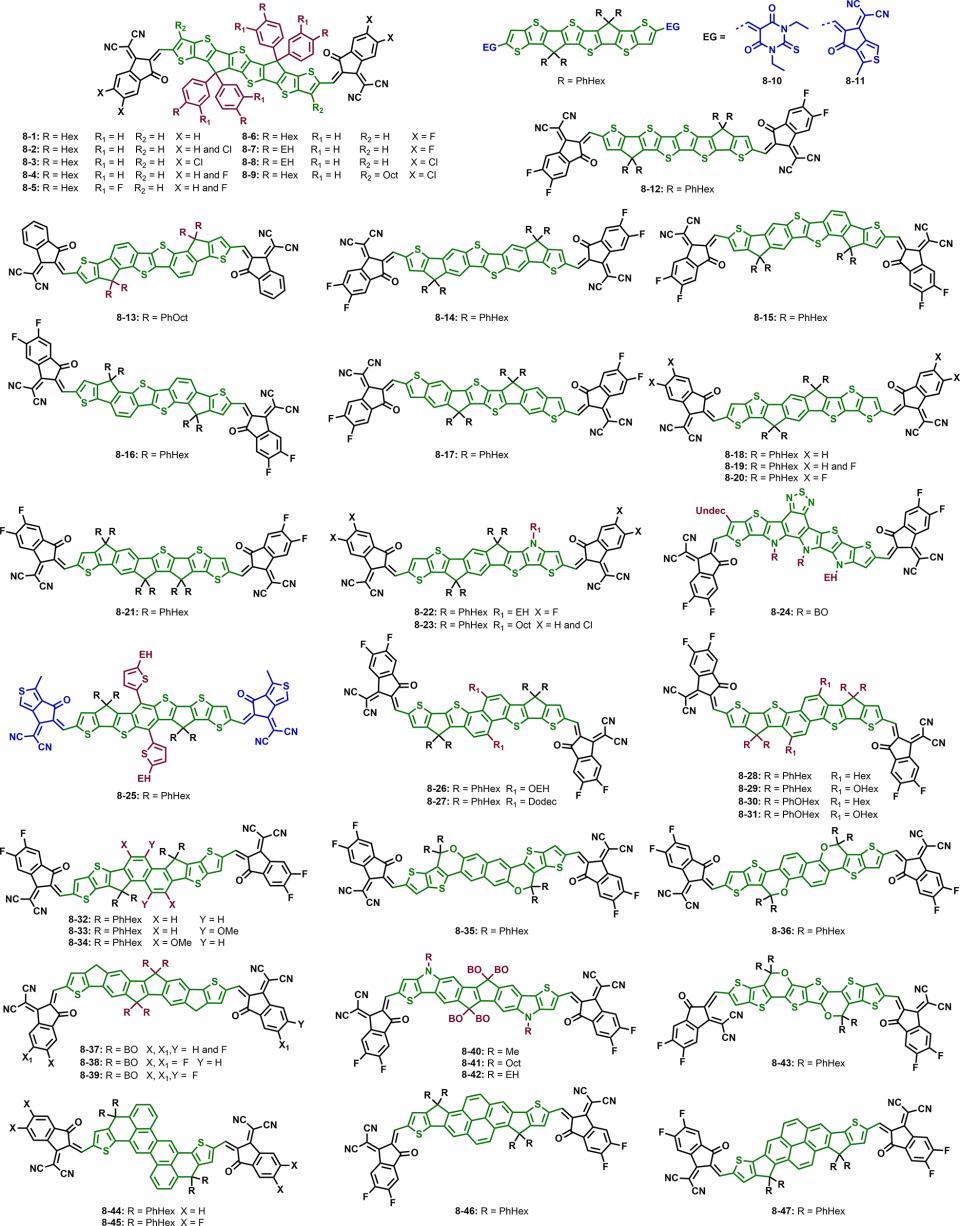
Structures of non-fullerene acceptors with eight fused rings.

A common design for the class of octocyclic acceptors is based on annulating three thieno[2,3-*b*]thiophene units via cyclopentadiene rings to the motif T-T-Cp-T-T-Cp-T-T and different acceptor end groups (see Figure 19, structures **8-1**–**8-6**). Chen et al. investigated the influence of chlorination using INCN, INCN-Cl, and INCN-2Cl acceptor units, which led to NFAs **8-1** (IXIC), **8-2** (IXIC-2Cl), and **8-3** (IXIC-4Cl), respectively.^509^

The chlorination of the end group causes a decrease of the band gap from 1.35 eV (**8-1**) to 1.30 eV (**8-2**) to 1.25 eV (**8-3**) mainly by lowering the LUMO energies (Table 16). Consequently, the *V*_OC_s of solar cells with PBDB-T also decreased in this order from 0.82 to 0.69 V. Moreover, the presence of chlorine increases the crystallinity as well as the π–π stacking, which facilitates charge carrier mobility and leads to higher FFs in the solar cells. While the PCEs of the PBDB-T/**8-1** as well as the PBDB-T/**8-3**-based solar cells were around 11.2%, the highest efficiencies of 12.2% in this series were obtained with the PBDB-T/**8-2**-based devices due to the best compromise of the *V*_OC_ and *J*_SC_ in this absorber material combination.^509^

**Table 16:** Optical, Electrical, and Photovoltaic Properties of the Non-Fullerene Acceptors Containing an Eight-Fused-Ring Structural Feature

NFA	original name	HOMO^a^ (eV)	LUMO^a^ (eV)	*E*_g_^opt^ (eV)	donor	D:A ratio	*V*_OC_ (V)	*J*_SC_(mA cm^–2^)	FF (%)	PCE (%)	μ_e_^e^(cm^2^ V^–1^ s^–1^)	ref.
**8-1**	IXIC	–5.13	–3.78^b^	1.35	PBDB-T	1:1	0.82	20.9	65	11.3	9.3 × 10^–4^/4.1 × 10^–4^	(509)
**8-2**	IXIC-2Cl	–5.20	–3.90^b^	1.30	PBDB-T	1:1	0.73	23.6	71	12.2	1.0 × 10^–3^/5.2 × 10^–4^	(509)
**8-3**	IXIC-4Cl	–5.20	–3.95^b^	1.25	PBDB-T	1:1	0.69	22.9	71	11.2	1.1 × 10^–3^/4.9 × 10^–4^	(509)
**8-4**	FOIC	–5.36	–3.92	1.32	PTB7-Th	1:1.5	0.74	24.0	67	12.0	1.2 × 10^–3^/5.1 × 10^–4^	(512)
**8-5**	FOIC1	–5.39	–3.99		PTB7-Th	1:1	0.69	23.4	69	11.0	1.8 × 10^–3^/3.9 × 10^–4^	(513)
**8-6**	F8IC	–5.43	–4.00	1.27	PTB7-Th	1:1.7	0.64	25.1	68	10.9	1.5 × 10^–3^/1.6 × 10^–4^	(504)
**8-7**	3TT-FIC	–5.42	–4.17	1.25	PTB7-Th	1:1.2	0.66	25.9	71	12.2	-/1.7 × 10^–4^	(514)
**8-8**	3TT-CIC	–5.24	–3.95	1.23	PTB7-Th	1:1.5	0.65	26.7	69	12.0	-/1.2 × 10^–4^	(515)
**8-9**	3TT-OCIC	–5.22	–3.91	1.29	PTB7-Th	1:1	0.68	26.5	69	12.4	-/1.4 × 10^–4^	(515)
**8-10**	6TBA	–5.41	–3.66	1.52	PBDT-2TC	1:1	1.0	13.9	63	8.76		(485)
**8-11**	T8Me	–5.39	–3.89	1.35	PM6	1:1	0.90	10.5	64	6.09	-/2.5 × 10^–4^	(292)
**8-12**	F8IC1	–5.43	–3.99	1.32	PTB7-Th	1:1.5	0.68	22.3	70	10.7	1.1 × 10^-3^ / 3.9 × 10^-4^	(486)
**8-13**	DBTIC	–5.45	–3.73	1.71	J52		0.94	13.8	67	8.64	-/9.3 × 10^–5^	(516)
**8-14**	Z1-aa	–5.67	–3.84	1.68	PM6	1:1	0.98	11.7	40	4.56	-/5.5 × 10^–6^	(517)
**8-15**	Z1-ab	–5.69	–3.87	1.68	PM6	1:1	1.00	14.8	61	9.60	-/1.9 × 10^–5^	(517)
**8-16**	Z1-bb	–5.70	–3.92	1.61	PM6	1:1	0.98	18.5	70	12.7	-/3.0 × 10^–4^	(517)
**8-17**	FTTBT	–5.57	–4.02	1.54	PM6	1:1	0.93	16.0	65	9.79	-/1.1 × 10^–4^	(315)
**8-18**	TTPTTT-IC	–5.64	–3.87	1.60	PBT1-C	1:1.2	1.00	12.5	64	7.91	2.8 × 10^–4^/1.7 × 10^–4^	(518)
**8-19**	TTPTTT-2F	–5.67	–4.04	1.54	PBT1-C	1:1.2	0.92	16.8	75	11.5	5.0 × 10^–4^/2.6 × 10^–4^	(518)
**8-20**	TTPTTT-4F	–5.69	–4.12	1.52	PBT1-C	1:1.2	0.86	19.4	72	12.1	6.5 × 10^–4^/3.2 × 10^–4^	(518)
**8-21**	AOIC	–5.50	–3.93	1.39	PTB7-Th	1:1.25	0.74	24.5	75	13.7	2.0 × 10^–3^/2.3 × 10^–3^	(371)
**8-22**	IDTTP-4F	–5.53	–3.97		PM7	1:1	0.91	21.2	72	13.8	6.2 × 10^–4^/3.5 × 10^–4^	(296)
**8-23**	N8IT	–5.41	–3.90	1.42	PM6	1:1	0.94	18.5	68	11.9	5.9 × 10^–4^/3.8 × 10^–4^	(294)
**8-24**	BTDTP-4F	–5.56	–3.93	1.30	PM6	1:1.2	0.87	21.3	71	13.1	7.9 × 10^–4^/4.2 × 10^–4^	(266)
**8-25**	a-BTTIC	–5.45	–3.83	1.43	PBDB-T	1:1	0.90	20.3	74	13.6	1.1 × 10^–3^/5.5 × 10^–4^	(519)
**8-26**	NTO-4F	–5.61	–3.88	1.55	PM6	1.25:1	0.99	19.1	61	11.5	-/4.0 × 10^–5^	(520)
**8-27**	NC-FIC	–5.43	–3.94	1.51	PBDB-T	1:1	0.88	15.2	56	7.52	-/7.0 × 10^–7^	(521)
**8-28**	IOIC2	–5.70^c^	–4.16^c^	1.54	PM6	1:1	0.97	16.3	66	10.5	1.0 × 10^–3^/0.5 × 10^–4^	(522)
**8-29**	IOIC3	–5.64^c^	–4.19^c^	1.45	PM6	1:1	0.92	20.0	70	12.8	1.5 × 10^–3^/0.6 × 10^–4^	(522)
**8-30**	IOIC4	–5.70^c^	–4.17^c^	1.53	PM6	1:1.2	0.96	17.3	67	11.1	1.1 × 10^–3^/0.4 × 10^–4^	(522)
**8-31**	IOIC5	–5.65^c^	–4.19^c^	1.46	PM6	1:1.2	0.92	20.9	72	13.8	1.5 × 10^–3^/1.4 × 10^–4^	(522)
**8-32**	NOIC	–5.76	–4.03	1.55	PM6	1:1	0.89	18.1	71	11.4	6.2 × 10^–4^/1.1 × 10^–4^	(523)
**8-33**	NOIC1	–5.41	–4.02	1.38	PM6	1:1	0.86	21.9	66	12.5	7.1 × 10^–4^/2.4 × 10^–4^	(523)
**8-34**	NOIC2	–5.64	–3.99	1.49	PM6	1:1	0.93	20.6	74	14.1	9.0 × 10^–4^/2.5 × 10^–4^	(523)
**8-35**	NOIC3	–5.83	–3.95	1.62	PM6	1:1	0.93	12.9	60	7.15	6.6 × 10^–5^/3.4 × 10^–5^	(523)
**8-36**	NOIC4	–5.64	–3.90	1.55	PM6	1:1	0.94	16.8	64	10.1	1.2 × 10^–4^/8.6 × 10^–5^	(523)
**8-37**	ZITI	–5.59	–3.74	1.53	J71	1:1	0.93	20.4	69	13.2	-/1.1 × 10^–4^	(524)
**8-38**	ZITI-3F	–5.64	–3.76	1.50	J71	1:1	0.90	20.7	72	13.2	-/1.6 × 10^–4^	(525)
**8-39**	ZITI-4F	–5.66	–3.81	1.47	J71	1:1	0.85	21.3	73	13.2	-/1.9 × 10^–4^	(525)
**8-40**	ZITI-N-CH_3_	–5.53	–3.82	1.41	PffBT4T-2OD	1:1	0.82	19.0	56	8.78	-/2.1 × 10^–4^	(526)
**8-41**	ZITI-N-C_8_H_17_	–5.53	–3.85	1.40	PffBT4T-2OD	1:1	0.80	21.0	72	12.1	-/2.5 × 10^–4^	(526)
**8-42**	ZITI-N-EH	–5.52	–3.86	1.40	PffBT4T-2OD	1:1	0.81	22.1	73	13.1	-/2.7 × 10^–4^	(526)
**8-43**	CO*i*8DFIC	–5.50^d^	–3.88		PTB7-Th	1:1	0.70	27.4	66	12.9	-/5.5 × 10^–5^	(527)
**8-44**	CRIC	–5.44	–3.82	1.53	J52-2F	1:1	0.94	13.4	49	6.12	-/7.8 × 10^–5^	(528)
**8-45**	CRIC-4F	–5.67	–3.90	1.60	J52-2F	1:1.5	0.89	13.1	48	5.61	-/1.9 × 10^–5^	(528)
**8-46**	Py-1	–5.79	–3.76	1.95	PTB7-Th	1:1	0.74	13.0	60	5.75	-/1.1 × 10^–4^	(529)
**8-47**	Py-2	–5.83	–3.78	1.97	PTB7-Th	1:1	0.75	9.67	47	3.47	-/1.9 × 10^–5^	(529)

a Obtained from the oxidation/reduction potential of the CV measurement if not otherwise stated.

b HOMO/LUMO energy levels obtained from the LUMO/HOMO levels determined by CV and the optical band gap.

c Obtained via UPS.

d HOMO obtained via PESA.

e Determined via the SCLC technique from the neat acceptor/donor:acceptor blend films if not otherwise stated.

Nian et al. obtained a PCE of 13.5% with solar cells based on the small molecular donor ZnP-TSEH and **8-1**.^510^ The combination of **8-3** with PM7, which has a lower HOMO level compared to PBDB-T, led to an increase of the *V*_OC_ from 0.69 V up to 0.80 V, which is also reflected in a higher PCE of 12.0%.^511^

The NFAs **8-4** (FOIC) and **8-6** (F8IC) contain INCN-F and INCN-2F end groups, respectively, and similar to the chlorinated compounds (**8-2**, **8-3**), the LUMO energy levels are lowered and the optical band gaps are decreasing compared to **8-1**.^504,512^ This is reflected in a lower *V*_OC_; however, a higher *J*_SC_ is obtained in PTB7-Th/**8-6**, compared to PTB7-Th/**8-4**-based solar cells. The introduction of fluorine atoms into the phenyl moiety of the side chains of **8-4** leads to **8-5** (FOIC1). While in a bulk heterojunction absorber layer in combination with PTB7-Th as a donor the PCE is slightly decreased compared to the **8-4**-based solar cells (11.0%), efficiencies of 12.0% were obtained by a sequentially deposited heterojunction.^513^ Changing the linear hexyl chains in **8-6** to branched ethylhexyl side chains results in **8-7** (3TT-FIC) and leaves the optical and photovoltaic properties almost unchanged; only the FF is increased, leading to a higher PCE of 12.2%.^514^

Structural variations of compound **8-3** bearing INCN-2Cl end groups were introduced by Gao et al.^515^ by replacing hexyl side chains with ethylhexyl chains, leading to structure **8-8** (3TT-CIC), or keeping the hexyl chains and introducing additional octyl side chains at the terminal thiophene units of the donor core, resulting in **8-9** (3TT-OCIC). As expected, structure **8-8** has a very similar optical band gap as **8-3**, whereas structure **8-9** leads to a higher band gap, which entailed a 30 mV increase in *V*_OC_ to 0.68 V compared to **8-8** in solar cells with PTB7-Th, resulting in a slight increase in PCE from 12.0 to 12.4%. Moreover, this terthieno[3,2-*b*]thiophene core structure was combined with a TBA end group, resulting in **8-10** (6TBA) as well as the CPTCN-Me terminal group leading to structure **8-11** (T8Me). In solar cells, **8-10** revealed a PCE of 8.76% in combination with PBDT-2TC.^485^ The NFA **8-11** was, however, tested in combination with PM6, which resulted in a lower PCE of 6.09% compared to similar hexacyclic (**6-8**) and heptacyclic structures (**7-271**); see Table 16. Although the absorption range is slightly broader, solar cells of **8-11** reached significantly lower *J*_SC_ values due to the unfavorable energy level alignment of the donor and the acceptor material in the absorber layer.^292^

Changing the position of the cyclopentadienyl rings, resulting in a T-Cp-T-T-T-T-Cp-T motif, leads to the NFA **8-12** (F8IC1).^486^ Compared to **8-6**, bearing the same side chains and end groups, no significant changes in the optoelectronic properties as well as the photovoltaic performance are revealed and PCEs of 10.7% are obtained in solar cells.

A further group of NFAs contains a central benzo[*b*]benzo[4,5]thieno[2,3-*d*]thiophene (BTTB) structural motif with annulated cyclopentathiophene units on each side (T-Cp-B-T-T-B-Cp-T) in combination with INCN units and is represented by the compounds **8-13**–**8-16**. This core unit is covalently rigidified and has a large π-conjugation, which typically causes high lying energy levels. The electron-donating ability of the donor core is comparatively low, which reduces intramolecular charge transfer, resulting in a larger optical band gap with significantly higher values between 1.61 and 1.71 eV than the NFAs **8-1**–**8-12**, discussed before with values around 1.3 eV. Solar cells of **8-13** (DBTIC, INCN end groups) with the donor J52 reached a maximum PCE of 8.64%.^516^ Using INCN-2F, the influence of different structural isomers was investigated on the structures **8-14**–**8-16**, with the same sequentially fused-ring cores but with different annulation of the terminal cyclopentathiophenes to the BTTB, as shown in Figure 19.^517^**8-14** (Z1-aa) has [2,3:*b*] and [6,7:*b*] annulation and thus a linear structure, **8-15** (Z1-ab) comprises a [1,2:*b*] and [6,7:*b*] annulation leading to a kink, whereas **8-16** (Z1-bb) has a double-kink due to a [1,2:*b*] and [5,6:*b*] annulation. Solar cells based on **8-16** and PM6 as a donor reveal clearly the highest photovoltaic performance (12.7%, see also Table 16) compared to 9.60 and 4.56% for solar cells with **8-15** and **8-14**, respectively. This originates from several facts: First, **8-16** has a reduced optical band gap (1.61 eV) compared to **8-14** and **8-15** (both 1.68 eV), which leads to a broader absorption range in combination with PM6. Second, **8-16** possesses a better phase morphology, more ordered π–π stacking, and significantly higher electron mobilities in the blend with PM6 compared to the other isomers, leading to a more balanced electron and hole mobility.^517^

Wang et al. introduced the centrosymmetric acceptor **8-17** (FTTBT) with a T-B-Cp-T-T-Cp-B-T motif, which is very similar to the heptacyclic structure **7-327**.^315^ Thereby, the central thiophene unit in **7-327** was replaced by a thienothiophene unit. Solar cells with a PM6/**8-17** blend led to a significant increase of the PCE (9.79%) compared to devices using **7-327** in the same solar cell setup, which revealed a PCE of only 3.47%. This performance increase is based on a smaller optical band gap originating from an extended π-conjugation and a phase morphology more beneficial for charge transport.^315^

Li et al. investigated asymmetric NFAs combining a dithienothiophene unit and a thienothiophene via a s-indacene unit leading to the octocyclic motif T-T-T-Cp-B-Cp-T-T.^518^ They focused on the influence of the fluorination degree of the INCN end groups comparing INCN (**8-18**, TTPTTT-IC), INCN-F (**8-19**, TTPTTT-2F), and INCN-2F (**8-20**, TTPTTT-4F) acceptor units. Fluorination caused a red-shift of the absorption along with a downshift of the HOMO/LUMO energies. Moreover, the fluorination led to strong intra- and intermolecular interactions and consequently also to a higher electron mobility. Solar cells of these acceptors with PTB1-C show—despite a decrease in *V*_OC_—an overall increase of the PCE with increasing fluorinination degree caused by higher *J*_SC_ and FF. While solar cells with the non-fluorinated analogue showed a PCE of 7.91%, the efficiency was increased to 11.5% for **8-19** and to 12.1% for the **8-20**-based solar cells.^518^ The asymmetric compound **8-21** (AOIC) is based on the five-fused-ring compound **5-24** having an extension of the fused-ring system on one side of the molecule.^371^ This change in the chemical structure results in upshifted HOMO/LUMO levels, a reduced band gap, and, in particular, a higher electron mobility (2.0 × 10^–3^ cm^2^ V^–1^ s^–1^) compared to **5-24**, which is reflected in a significantly increased photovoltaic performance (PTB7-Th/**8-21**, 13.7%; PTB7-Th/**5-24**, 5.61%).

In the NFA **8-22** (IDTTP-4F), one thiophene in the central unit is substituted by an *N*-alkylated pyrrole leading to a T-T-Cp-B-Cp-T-P-T structure.^296^**8-22** has a C-shape and is related to **7-280**, which reveals an S-shape confirmation. Solar cells using PM7 as a donor revealed higher PCEs with the S-shape (15.2%) compared to 13.8% obtained with **8-22**. This difference is due to the more pronounced aggregation of the C-shaped **8-22** in contrast to the S-shaped **7-280**.^296^**8-23** (N8IT) bears INCN-Cl end groups and an *n*-octyl alkyl chain on the pyrrole moiety, which leads to slightly elevated HOMO and LUMO levels compared to **8-22** and an optical band gap of 1.42 eV; PM6/**8-23**-based solar cells reveal PCEs of 11.9%.^294^ The importance of the optimization and tuning of the molecular packing and the phase morphology is also thoroughly elaborated by Luo et al. based on the compounds **8-24** (BTDTP-4F) and **7-222** as well as **8-22** and **7-280**.^266^ While in the latter mentioned compounds, the S-shape led to the higher PCE, in the case of **8-24** and **7-222**, the NFA with the C-shape (**7-222**) revealed the higher photovoltaic performance compared to **8-24** outlining an S-shape. This is due to the fact that, in this case, the C-shape led to the more favorable phase morphology and more pronounced face-on orientation, which were identified to be the main reasons for the difference in the photovoltaic performance.^266^

A good photovoltaic performance was obtained in solar cells using **8-25** (a-BTTIC) combined with PBDB-T, yielding a PCE of 13.6%.^519^ This asymmetric compound is based on the symmetric structure **7-251** to which one thiophene ring is added, shows a T-Cp-T-B-T-Cp-T-T motif, and bears CPTCN-Me acceptor units. Compared to **7-251**,^282^ the band gap is reduced and the LUMO level is elevated to −3.83 eV. This leads to simultaneously increased *J*_SC_ and *V*_OC_ values, and a lower energy loss was observed for PBDB-T/**8-25**-based devices.

The molecule **8-26** (NTO-4F) is based on a naphthodithiophene core bearing alkoxy side chains.^520^ In this structure, the oxygen in the side chain and the sulfur of the thiophene unit can form intramolecular non-covalent S–O interactions and can thereby increase the size of the conjugated system. **8-26** reveals an optical band gap of 1.55 eV and an elevated LUMO level (−3.88 eV) which leads to a high *V*_OC_ (0.99 V) in solar cells with PM6 as a donor. These solar cells exhibit a PCE of 11.5% and good thermal stability.^520^ The NFA **8-27** bears linear dodecyl side chains instead of the ethylhexyloxy side chains of **8-26**, and for PBDB-T/**8-27** devices, PCEs of 7.52% are reported.^521^ Zhu et al. investigated the influence of the alkoxylation position on this core unit (**8-28**–**8-31**).^522^ Alkoxylation on the naphthodithiophene core leads to a reduced optical band gap, upshifted HOMO and downshifted LUMO levels, and slightly increased electron mobility, while the alkoxylation of the Cp unit does not have significant effects. Compared to **8-28** (IOIC2, no alkoxylation), hexyloxy chains on the core (**8-29**, IOIC3) lead to an increase of the PCE from 10.5 to 12.8%, while the alkoxylation of the Cp unit (**8-30**, IOC4) only leads to an increase to 11.1%. The highest photovoltaic performance (13.8%) was obtained with the NFA **8-31** (IOIC5, alkoxylation at both sites) in combination with PM6.^522^ Using FTAZ as a conjugated polymer, **8-28** reveals PCEs up to 12.3%^530^ and PTB7-Th/**8-29**-based solar cells show PCEs of 13.1%.^531^

Li et al. investigated the influence of the methoxylation of the naphthalene unit in NFAs with the structural motif T-T-Cp-B-B-Cp-T-T combined with INCN-2F end groups (**8-32**–**8-36**). Different methoxylation positions strongly influence the material properties; i.e., methoxy substitution at the core leads to red-shifted absorption, higher crystallinity, and charge carrier mobility, while carbon–oxygen bridges (present in **8-35** and **8-36**) were found to be not beneficial regarding the material properties as well as the solar cell performance. Consequently, PM6/**8-35** solar cells reveal a PCE of 7.15%, the lowest value within this series, and PCEs of 14.1% are obtained with PM6/**8-34**-based devices.^523^

The acceptors **8-37**–**8-39** contain a fused di(thienocyclopenta)indenoindene (ZIT) core (with the T-Cp-B-Cp-Cp-B-Cp-T motif) and two INCN-F (**8-37**, ZITI), one INCN-F, and one INCN-2F on each side (**8-38**, ZITI-3F) and two INCN-2F units (**8-39**, ZITI-4F) as termini.^524,525^ These materials reveal an optical band gap of ∼1.5 eV, whereby it is slightly lowered by the fluorination. Zhang et al. synthesized **8-38** and **8-39** in a one-pot synthesis, which yielded a molar ratio of 1:1.^525^ After separation of these compounds and preparation of solar cells in combination with the donor J71, very similar PCEs of 13.15 and 13.18% were obtained, which is also similar to the PCE of J71/**8-37**-based solar cells.^524^ When the mixture of **8-38** and **8-39** (ZITI-m), as yielded in the synthesis, is directly used for the device preparation, the obtained ternary solar cells revealed an improved PCE of 13.7% which was attributed to synergy effects in the ternary blend, e.g., a broader absorption range and a more balanced charge carrier transport.^525^ In the compounds **8-40**–**8-42**, the outer Cp units in the ZIT core are replaced by pyrrole units bearing different alkyl chains (**8-40**, ZITI-N-CH_3_, methyl; **8-41**, ZITI-N-C_8_, *n*-octyl; **8-42**, ZITI-N-EH, ethylhexyl). While the different alkyl chains only hardly affect the positions of the energy levels and the optical properties, the solar cell efficiency based on these NFAs and PffBT4T-2OD increases remarkably from 8.78% (**8-40**) to 12.1% (**8-41**) and 13.1% (**8-42**), which is due to the fact that, in the PffBT4T-2OD/**8-42**-based absorber layers, the smallest domain sizes and the highest crystallinity are found within this series.^526^**8-43** (CO*i*8DFIC) contains two carbon–oxygen bridges in the fused-ring core and INCN-2F end groups and shows high crystallinity in the absorber layers. In combination with PTB7-Th, PCEs up to 12.9% are obtained.^527^ This acceptor was also used compared to its smaller sized central core analogues (five to seven fused rings). It could be seen that the PCE was increasing together with the conjugation length. Obviously, changes in the conjugation length have an impact on the optical band gap and energy levels of these acceptors; however, also significant changes in phase morphology are observed.^307^

Furthermore, fused octocyclic acceptors (**8-44**, CRIC, and **8-45**, CRIC-4F) based on a chrysene core are reported by Zhao et al. and reveal optical band gaps of 1.53 and 1.60 eV, respectively.^528^ Solar cells based on J52-2F/**8-44** showed a PCE of 6.12%, and an efficiency of 5.61% was obtained in the same device setup with the fluorinated analogue **8-45**. The higher photovoltaic performance of the J52-2F/**8-44** blend is ascribed to a higher LUMO energy level (−3.82 eV compared to −3.90 eV) and a more beneficial crystallinity and blend morphology.^528^

The molecules **8-46** (Py-1) and **8-47** (Py-2) bear a dithienocyclopentapyrene core and INCN-2F acceptor groups, whereby **8-46** is the centrosymmetric and **8-47** the axisymmetric isomer.^529^ While the optical band gap (**8-46**, 1.95 eV; **8-47**, 1.97 eV) and the energy levels show no significant difference, the centrosymmetric isomer shows a significantly higher electron mobility in the blend with PTB7-Th, which also results in an increased PCE of 5.75% (compared to 3.47% for a PTB7-Th/**8-47**-based solar cell).^529^

## 7 Nine Fused Aromatic Ring Systems

**Figure 20:**
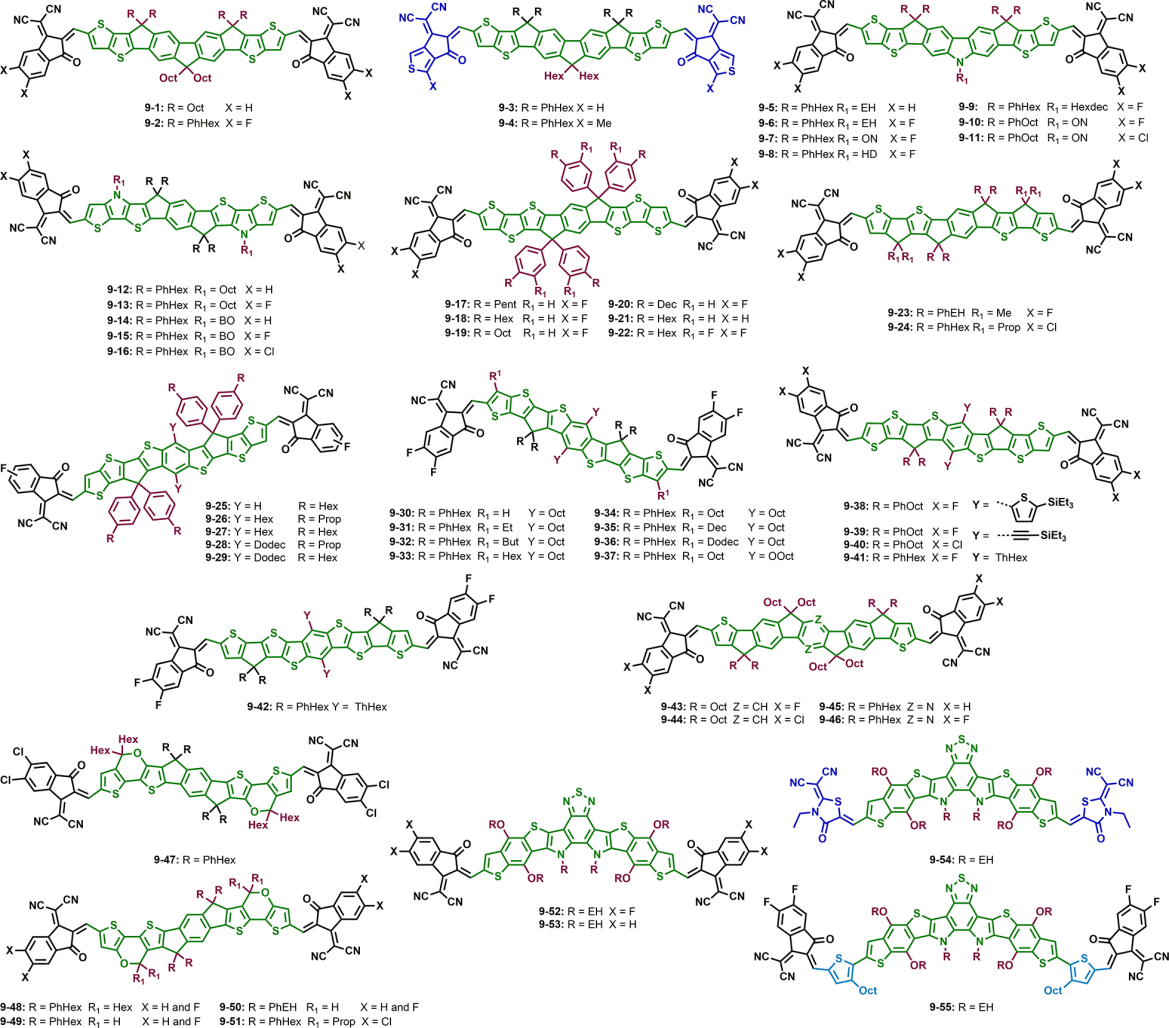
Structures of non-fullerene acceptors with nine fused rings.

One type of nonacyclic NFAs is based on combining two cyclopentathienothiophene units via annulation with a central fluorene or carbazole unit (**9-1**–**9-11**)—with a T-T-Cp-B-Cp (or Py)-B-T-T motif (see also Figure 20 and Table 17). **9-1** (BTTFIC) contains a bis(thieno[3,2-*b*]thieno)cyclopentafluorene donor core and INCN units as end groups and has an optical band gap of 1.58 eV and HOMO and LUMO energy levels of −5.56 and −3.95 eV.^532^ The carbazole-based analogue **9-5** (CZTT-IC) reveals rather similar optical properties, energy levels, and photovoltaic performance.^533^ In combination with PBDB-T, PCEs of 9.00% were obtained for the **9-1**-based photovoltaic devices, while, for solar cells with PBDB-T/**9-5**, 9.87% are reached. Also, the compounds **9-3** (FTTCN) and **9-4** (FTTCN-M) with thiophene-based end groups have nearly identical optical and electrochemical properties, but the efficiencies of solar cells from blends with PBDB-T reach PCEs above 10%.^534^ Further improvement can be achieved if INCN-2F end groups are used. To that end, compound **9-2** (4TFIC-4F, BTTFIC4F-Ar) reaches a PCE above 11%, but its carbazole-based analogue **9-7** (4TCIC-4F) even 13.0%.^535,536^ Albeit, here a different donor polymer (PM7) was used than for compound **9-1**. Fluorination of the end group results in a lower band gap and downshifted energy levels, as can be seen if acceptor **9-6** (CZTT-4F) is compared to its non-fluorinated analogue **9-5**. Thus, solar cells with PBDB-T and **9-6** show lower *V*_OC_ but higher *J*_SC_ values, leading to a very similar overall PCE of 9.8–9.9%. However, a lower miscibility of the blend with PM6 is observed for **9-6**, which leads to a beneficial polymer/NFA morphology in the bulk heterojunction film and a higher domain purity. Overall, this results in a PCE of 12.1% of PM6/**9-6**-based solar cells.^533^ Variations of the alkyl substituent on the carbazole nitrogen have a marginal influence on the optical and electrochemical properties of the molecule. This can be seen when **9-7** (4TC-4F-C8C8) is compared to **9-8** (4TC-4F-C6C8) and **9-9** (4TC-4F-C16). A linear alkyl chain (**9-9**) allows for the highest crystallinity, while the α- and β-branched chains have moderate and low crystallinities, respectively (**9-7** and **9-8**). This has a large influence on the PV performance in blends with the donor PBTIBDTT. The best PCE is achieved with α-branched **9-7** (12.1%), the lowest with the β-branched **9-8** (2.38%).^537^

**Table 17:** Optical, Electrical, and Photovoltaic Properties of the Non-Fullerene Acceptors Containing a Nine-Fused-Ring Structural Feature

NFA	original name	HOMO^a^ (eV)	LUMO^a^ (eV)	*E*_g_^opt^ (eV)	donor	D:A ratio	*V*_OC_ (V)	*J*_SC_(mA cm^–2^)	FF (%)	PCE (%)	μ_e_^c^(cm^2^ V^–1^ s^–1^)	ref.
**9-1**	BTTFIC	–5.56	–3.95	1.58	PBDB-T	1.5:1	0.94	17.7	54	9.00	-/1.4 × 10^–4^	(532)
**9-2**	4TFIC-4F	–5.66	–3.98	1.55	PM7	1:1	0.93	16.6	73	11.2	1.7 × 10^–4^/1.9 × 10^–4^	(535)
	BTTFIC4F-Ar	–5.64	–4.05	1.54	PM7	1.5:1	0.95	19.5	64	11.8	-/1.2 × 10^–4^	(536)
**9-3**	FTTCN	–5.54	–3.95	1.56	PBDB-T	1:1	0.90	15.9	74	10.6	-/6.6 × 10^–4^	(534)
**9-4**	FTTCN-M	–5.53	–3.91	1.57	PBDB-T	1:1	0.93	15.2	71	10.1	-/5.5 × 10^–4^	(534)
**9-5**	CZTT-IC	–5.54	–3.97	1.56	PBDB-T	1:1	0.97	17.3	59	9.87	-/8.6 × 10^–4^	(533)
**9-6**	CZTT-4F	–5.61	–4.11	1.48	PM6	1:1.25	0.94	19.7	65	12.1	-/1.9 × 10^–3^	(533)
**9-7**	4TCIC-4F	–5.53	–3.95	1.51	PM7	1:1	0.94	19.0	73	13.0	2.9 × 10^–4^/2.4 × 10^–4^	(535)
	4TC-4F-C8C8	–5.60	–3.96	1.51	PBTIBDTT	1:1.5	0.90	18.6	72	12.1	2.3 × 10^–4^/2.0 × 10^–4^	(537)
**9-8**	4TC-4F-C6C8	–5.59	–4.00	1.49	PBTIBDTT	1:1	0.84	6.75	42	2.38	7.2 × 10^–5^/1.5 × 10^–6^	(537)
**9-9**	4TC-4F-C16	–5.53	–3.96	1.48	PBTIBDTT	1:1.25	0.91	16.1	65	9.53	2.9 × 10^–4^/7.1 × 10^–5^	(537)
**9-10**	DTTC-4F	–5.69	–3.91	1.55	PM6	1:1.1	0.95	21.7	68	13.9	-/3.0 × 10^–4^	(538)
**9-11**	DTTC-4Cl	–5.72	–4.03	1.47	PM6	1:1	0.92	22.6	74	15.4	-/7.9 × 10^–4^	(538)
**9-12**	INPIC	–5.36	–3.82	1.46	PBDB-T	1:1	0.96	8.55	53	4.31	2.0 × 10^–4^/9.6 × 10^–5^	(539)
**9-13**	INPIC-4F	–5.42	–3.94	1.39	PBDB-T	1:1	0.85	21.6	72	13.1	1.3 × 10^–3^/5.0 × 10^–4^	(539)
**9-14**	IPIC	–5.47	–3.82	1.44	PBDB-T	1:1	0.95	7.16	59	3.98	-/2.5 × 10^–7^	(540)
**9-15**	IPIC-4F	–5.54	–3.94	1.38	PBDB-T	1:1	0.84	19.8	67	11.1	-/5.6 × 10^–5^	(540)
**9-16**	IPIC-4Cl	–5.51	–3.95	1.32	PBDB-T	1:1	0.81	22.2	74	13.4	-/1.0 × 10^–4^	(540)
**9-17**	2F-C5	–5.36	–3.95	1.49	PBDB-T	1:1	0.77	19.6	67	10.2	2.4 × 10^–4^/1.3 × 10^–4^	(541)
**9-18**	2F-C6	–5.36	–3.97	1.48	PBDB-T	1:1	0.79	21.0	68	11.3	5.8 × 10^–4^/2.2 × 10^–4^	(541)
**9-19**	2F-C8	–5.38	–3.98	1.49	PBDB-T	1:1	0.80	21.3	72	12.3	7.9 × 10^–4^/2.6 × 10^–4^	(541)
**9-20**	2F-C10	–5.37	–3.97	1.49	PBDB-T	1:1	0.80	19.9	65	10.4	1.3 × 10^–3^/8.9 × 10^–5^	(541)
**9-21**	INIC	–5.47	–3.89	1.57	PBD-SF	1:1.4	1.05	9.89	49	5.1	9.0 × 10^–5^/2.1 × 10^–5^	(542)
**9-22**	FINIC	–5.56	–4.05	1.51	PBD-SF	1:1.4	0.87	22.0	73	14.0	8.3 × 10^–4^/4.5 × 10^–4^	(542)
**9-23**	BCPT-4F	–5.36	–3.91	1.32	PBDB-T	1:1	0.78	23.0	70	12.4	-/2.9 × 10^–4^	(543)
**9-24**	CPDT-4Cl	–5.32	–3.78	1.35	PTB7-Th	1:1.5	0.74	23.2	69	11.9	3.6 × 10^–4^/3.1 × 10^–4^	(544)
**9-25**	NNFA[0,6]	–5.27	–3.86	1.38	FTAZ	1:1.4	0.84	19.8	57	9.52	-/3.9 × 10^–5^	(545)
**9-26**	NNFA[6,3]	–5.21	–3.83	1.37	FTAZ	1:1.4	0.87	14.7	59	7.56	-/7.2 × 10^–6^	(545)
**9-27**	NNFA[6,6]	–5.23	–3.84	1.37	FTAZ	1:1.4	0.87	19.9	61	10.6	-/4.9 × 10^–5^	(545)
**9-28**	NNFA[12,3]	–5.23	–3.84	1.37	FTAZ	1:1.4	0.86	19.3	65	10.8	-/5.7 × 10^–5^	(545)
**9-29**	NNFA[12,6]	–5.21	–3.83	1.37	FTAZ	1:1.4	0.86	18.9	59	9.54	-/2.8 × 10^–5^	(545)
**9-30**	TTC0-4F	–5.65^b^	–4.27^b^	1.38	PM6	1:1	0.81	19.5	67	10.6	-/2.8 × 10^–4^	(546)
**9-31**	TTC2-4F	–5.60^b^	–4.20^b^	1.40	PM6	1:1	0.84	20.3	67	11.4		(546)
**9-32**	TTC4-4F	–5.60^b^	–4.20^b^	1.40	PM6	1:1	0.88	21.0	68	12.7	-/3.5 × 10^–4^	(546)
**9-33**	TTC6-4F	–5.60^b^	–4.20^b^	1.40	PM6	1:1	0.89	21.7	69	13.2		(546)
**9-34**	TTC8-4F	–5.60^b^	–4.19^b^	1.41	PM6	1:1	0.90	22.4	69	14.0	-/3.7 × 10^–4^	(546)
**9-35**	TTC10-4F	–5.60^b^	–4.19^b^	1.41	PM6	1:1	0.90	22.6	65	13.2		(546)
**9-36**	TTC12-4F	–5.60^b^	–4.19^b^	1.41	PM6	1:1	0.91	22.3	62	12.5	-/3.7 × 10^–4^	(546)
**9-37**	TTC8-O1-4F	–5.42	–3.86	1.31	PBDB-T	1:1	0.82	23.0	70	13.2	-/2.5 × 10^–4^	(547)
**9-38**	IN-4F	–5.56	–3.99	1.38	PM6	1:1	0.87	21.8	69	13.0	0.7 × 10^–5^/6.8 × 10^–4^	(548)
**9-39**	ISI-4F	–5.65	–4.01	1.43	PM6	1:1	0.88	22.8	62	12.5	-/4.3 × 10^–4^	(549)
**9-40**	ISI-4Cl	–5.64	–4.05	1.42	PM6	1:1	0.87	20.5	68	12.1	-/3.2 × 10^–4^	(549)
**9-41**	FNIC2	–5.56	–4.00	1.38	PTB7-Th	1:1	0.74	23.9	73	13.0	1.7 × 10^–3^/1.4 × 10^–3^	(550)
**9-42**	FNIC1	–5.61	–3.92	1.48	PTB7-Th	1:1	0.77	20.0	66	10.3	1.2 × 10^–3^/6.0 × 10^–4^	(550)
**9-43**	TfIF-4F	–5.67	–3.98	1.61	PM7	1:1	0.97	18.5	78	14.0	3.7 × 10^–4^/3.5 × 10^–4^	(551)
**9-44**	TfIF-4Cl	–5.71	–4.09	1.57	PM7	1:1	0.97	18.5	76	13.7	6.0 × 10^–4^/4.6 × 10^–4^	(551)
**9-45**	IPY-T-IC	–5.64	–3.48	1.71	PTB7-Th	1:1.5	0.91	13.1	52	6.19	-/4.5 × 10^–5^	(552)
**9-46**	IPY-T-ICF	–5.67	–3.51	1.67	PTB7-Th	1:1.5	0.76	15.5	59	7.00	-/9.7 × 10^–5^	(552)
**9-47**	ITOT-4Cl	–5.49	–3.90	1.28	PBDB-T	1:1	0.78	22.9	71	12.5	-/1.8 × 10^–4^	(553)
**9-48**	IDTODT-1	–5.35	–3.90	1.32	PBDB-T	1:1	0.80	14.5	59	6.87	-/1.3 × 10^–5^	(554)
**9-49**	IDTODT-2	–5.38	–3.95	1.34	PBDB-T	1:1	0.77	15.3	59	6.99	-/6.1 × 10^–5^	(554)
**9-50**	IDTODT-3	–5.39	–3.96	1.34	PBDB-T	1:1	0.79	17.3	61	8.34	-/5.2 × 10^–5^	(554)
**9-51**	DTPR-4Cl	–5.35	–3.85	1.30	PTB7-Th	1:1.5	0.68	25.0	62	10.5	2.1 × 10^–4^/1.4 × 10^–4^	(544)
**9-52**	X94FIC	–5.58	–4.17	1.25	PBDB-T	1:1	0.73	14.7	66	7.08	-/6.1 × 10^–4^	(555)
**9-53**	X9IC	–5.53	–4.06	1.29	PBDB-T	1:1	0.86	11.4	64	6.29	-/6.2 × 10^–4^	(555)
**9-54**	X9Rd	–5.51	–3.80	1.45	PBDB-T	1:1	1.08	6.70	34	2.48	-/6.6 × 10^–4^	(555)
**9-55**	X9T4FIC	–5.51	–4.10	1.29	PBDB-T	1:1	0.85	7.01	54	3.22	-/5.2 × 10^–4^	(555)

a Obtained from the oxidation/reduction potential of the CV measurement if not otherwise stated.

b Obtained via UPS.

c Determined via the SCLC technique from the neat acceptor/donor:acceptor blend films if not otherwise stated.

Compound **9-10** (DTTC-4F) is very similar to **9-7**, with the only difference being the use of octylphenyl instead of hexylphenyl side chains on the central core.^538^ Solar cells with PM6 gave PCE values of almost 14%. **9-10** was compared to the analogous compound **9-11** (DTTC-4Cl) containing an INCN-2Cl end group. The chlorinated **9-11** reveals a smaller optical band gap as well as slightly lower energy levels, stronger π–π interaction, and a significantly increased PCE of 15.4% for PM6/**9-11**-based solar cells (cf. Table 17).^538^

The NFAs **9-12** (INPIC) and **9-13** (INPIC-4F) are based on a donor core with two dithieno[3,2-*b*:2′,3′-*d*]pyrrole units annulated via a S-indacene unit (structural motif: T-P-T-Cp-B-Cp-T-P-T) and INCN as well as INCN-2F end groups, respectively.^539^ As expected, the fluorinated analogue has a narrower band gap of 1.39 eV, downshifted energy levels (−3.94 eV compared to −3.82 eV), a higher crystallinity, as well as a higher electron mobility. While solar cells based on PBDB-T/**9-12** led to a moderate PCE of 4.31%, PBDB-T/**9-13**-based devices showed a PCE of 13.1%.^539,556^ Replacing the octyl chains on the pyrrole nitrogens in compounds **9-12** and **9-13** by 2-butyloctyl chains (compounds **9-14**, IPIC, and **9-15**, IPIC-4F) results in significantly lower electron mobilities (for blends with PBDB-T). Consequently, the solar cells yield lower PCEs. Nevertheless, replacing fluorine by chlorine in the end group as in compound **9-16** (IPIC-4Cl) restores the good electron mobility, and a remarkable PCE of 13.4% is achieved.^540^

Using dithienothiophene units instead of dithienopyrroles leads to a T-T-T-Cp-B-Cp-T-T-T motif.^101^ In a study by Zhao et al., the influence of increasing alkyl chain lengths was systematically investigated for pentyl (**9-17**, 2F-C5), hexyl (**9-18**, 2F-C6), octyl (**9-19**, 2F-C8), and decyl side chains (**9-20**, 2F-C10), as shown in Figure 20.^541^ Longer side chains increased the miscibility of the acceptor with the used polymer PBDB-T, which led to smaller domain sizes and lower domain purity. On the one hand, smaller domain sizes are beneficial for efficient exciton dissociation, but on the other hand, a too low domain purity leads to increased bimolecular recombination. Based on that, the highest PCE (12.3%) was obtained for the PBDB-T/**9-19**-based absorber layer with octyl side chains, which showed the best compromise of domain size and domain purity. Acceptor **9-21** (INIC) is structurally very similar to **9-18** but comprises unsubstituted INCN termini, leading to a higher optical band gap and a lower electron mobility. Therefore, solar cells with the donor PBD-SF yield a moderate PCE of 5.1%. Solar cells with the same donor and the difluorinated acceptor **9-22** (FINIC) exhibit a significantly increased PCE (14.0%). In comparison to devices of the structurally similar **9-18** with the PBDB-T donor, the largest improvement has been reached in *V*_OC_ despite **9-22** having a lower LUMO level than **9-18**.^542^

Exchanging the pyrrole ring in **9-13** with a cyclopentadiene ring leads to the conjugated structure of **9-23** (BCPT-4F).^543^**9-23** showed a low band gap of 1.32 eV, and solar cells based on this acceptor and PBDB-T gave PCE values of 12.4%. Structurally similar **9-24** (CPDT-4Cl) has upshifted energy levels and an increased optical band gap (1.35 eV). Solar cells with PTB7-Th yield an overall similar performance (PCE: 11.9%).^544^

The influence of alkyl chain lengths was investigated in NFAs with a benzodithiophene-based central core, flanked by two cyclopentathienothiophene units (T-T-Cp-T-B-T-Cp-T-T) and INCN-F acceptor units (**9-25**–**9-29**).^545^ Alkyl chains were introduced on the central core (*in-plane*) and on the phenyl side chains (*out-of-plane*). All molecules had very similar optical and electrochemical properties, but the solubility was varying between 23 and 226 mg mL^–1^. Solar cells with the FTAZ donor achieved the best PCE values (10.8%) using **9-28** (NNFA[12,3]), which bears dodecyl chains in-plane and propyl side chains out-of-plane. In another study, various alkyl chains were introduced on the peripheral thiophene unit, keeping all other groups the same: octyl (central benzene) and hexylphenyl (on the Cp-rings) side chains and INCN-2F groups.^546^ Alkyl chains, regardless of their length (ethyl to dodecyl, compounds **9-31**–**9-36**, TTCn-4F), slightly lower the energy levels and increase the optical band gap compared to the unsubstituted **9-30**, TTC0-4F (1.40 eV vs 1.38 eV). Nevertheless, solar cells of alkyl derivatives (blends with PM6) show an improved electron mobility and high PCEs (11–14%).^546^ Replacing the octyl chains on the core with octyloxy groups gives compound **9-37** (TTC8-O1-4F),^547^ which shows a narrower optical band gap of 1.31 eV and a similar electron mobility of 2.5 × 10^–4^ cm^2^ V^–1^ s^–1^. In the acceptors **9-38** (IN-4F) and **9-39** (ISI-4F), a silyl-based side chain is introduced via thiophene (**9-38**) or triple bond (**9-39**) spacers. Compared to the octyl-substituted compound **9-30**, the LUMO energies are higher. Thus, devices with PM6 have increased *V*_OC_ values and due to a higher electron mobility also slightly higher *J*_SC_ values, leading to overall improved PCEs of 13.0% (**9-38**)^548^ and 12.5% (**9-39**). Replacing fluorine atoms in the end group by chlorine (**9-40**, ISI-4Cl) does not improve the PCE (12.1%).^549^ Wang et al. studied the NFA **9-41** (FNIC2), bearing a hexylthiophene substituent at the central benzene unit and its isomer **9-42** (FNIC1) with the structure T-Cp-T-T-B-T-T-Cp-T.^550^**9-41** exhibits a lower band gap of 1.38 eV and a higher electron mobility (1.7 × 10^–3^ cm^2^ V^–1^ s^–1^) compared to **9-42** (1.48 eV, 1.2 × 10^–3^ cm^2^ V^–1^ s^–1^). While consequently the *V*_OC_ of PTB7-Th/**9-41**-based solar cells is 30 mV lower, the *J*_SC_ is increased from 20.0 to 23.9 mA cm^–2^. This, together with a higher FF, leads to an increase in the PCE from 10.3% (PTB7-Th/**9-42**) to 13.0% (PTB7-Th/**9-41**).

A further nonacyclic structure is based on indenofluorene flanked by cyclopenta[*b*]thiophene units (T-Cp-B-Cp-B-Cp-B-Cp-T) at both sides and either INCN-2F or INCN-2Cl acceptor groups, as realized in **9-43** (TfIF-4F) and **9-44** (TfIF-4Cl).^551^ The chlorinated NFA shows a lower band gap, deeper energy levels, and better molecular packing. However, the photovoltaic properties of PM7/**9-43**- and PM7/**9-44**-based devices were rather similar, yielding PCEs of 14.0 and 13.7%, respectively. The solar cells based on the chlorinated analogue revealed a low layer thickness dependence of the PCE due to the higher electron mobility.^551^ Exchanging the central benzene ring with a pyrazine yields **9-45** (IPY-T-IC) and its end group fluorinated analogue **9-46** (IPY-T-ICF). NFA **9-46** has a significantly higher LUMO, and due to unchanged HOMO energy, a higher optical band gap is observed compared to **9-43**. Solar cells of PTB7-Th/**9-46** blends yielded PCEs of 7.00%.^552^

The dithienopyrane moieties containing acceptor **9-47** (ITOT-4Cl) have a low optical band gap (1.28 eV) and a good electron mobility in blend with PBDB-T. Due to its low LUMO energy, solar cells are not reaching high *V*_OC_ values (0.78 V), but the low band gap and good electron mobility contribute to a high *J*_SC_ (22.9 mA cm^–2^) and thus decent PCEs (12.5%).^553^ Acceptor **9-48** (IDTODT-1) also is comprised of a dithienopyrane moiety, but the oxygen atoms are located in the *outer* direction, i.e., closer to the periphery of the central core. Since INCN-F groups are used instead of INCN-2F, one cannot draw a conclusion about how the position of the oxygen atom influences the molecular properties. Nevertheless, compound **9-48** has the same LUMO energy as **9-47**, but the optical band gap is higher (by 40 meV) and the electron mobility is one order of magnitude lower. Thus, solar cells with PBDB-T have much lower *J*_SC_s (14.5 mA cm^–2^), smaller FFs (59% vs 71%), and therefore reduced PCEs (6.87%). The morphology of the blend can be improved by side chain modifications; thus, both **9-49** (IDTODT-2) and **9-50** (DITODT-3) have an improved electron mobility and a higher PCE (6.99 and 8.34%, respectively).^554^ The acceptor **9-51** (DTPR-4Cl) has INCN-2Cl end groups and in comparison to **9-47** different side chains, leading to a slightly higher LUMO and a larger optical band gap. Good electron mobility ensures high *J*_SC_ values for its devices with PTB7-Th (25.0 mA cm^–2^), but a low *V*_OC_ (0.68 V) and FF (62%) limits the PCE (10.5%).^544^

Similar to the Y-series, the A–(DA′D)–A approach was introduced in nonacyclic NFAs, as realized in structures **9-52**–**9-55** by the insertion of additional benzene rings replacing the thienothiophenes with benzodithiophene units.^555^ These structures comprise different acceptor termini, **9-52** (X94FIC) INCN-2F (analogue to Y6, **7-145**), **9-53** (X9IC) INCN (analogue to Y5, **7-142**), and **9-54** (X9Rd) an RCN end group (analogue to TPBT-RCN, **7-200**). **9-55** (X9T4FIC) bears additional thiophene π-spacers between the core unit and the INCN-2F termini. Within this series, **9-52** showed the highest performance in solar cells (PCE: 7.08%) in combination with PBDB-T. While **9-52**, **9-53**, and **9-55** revealed rather similar optical band gap values between 1.25 and 1.30 eV, **9-54** possesses an optical band gap of 1.45 eV due to a significantly upshifted LUMO level (−3.80 eV).^555^

## 8 Aromatic Ring Systems with 10–13 Fused Rings

**Figure 21:**
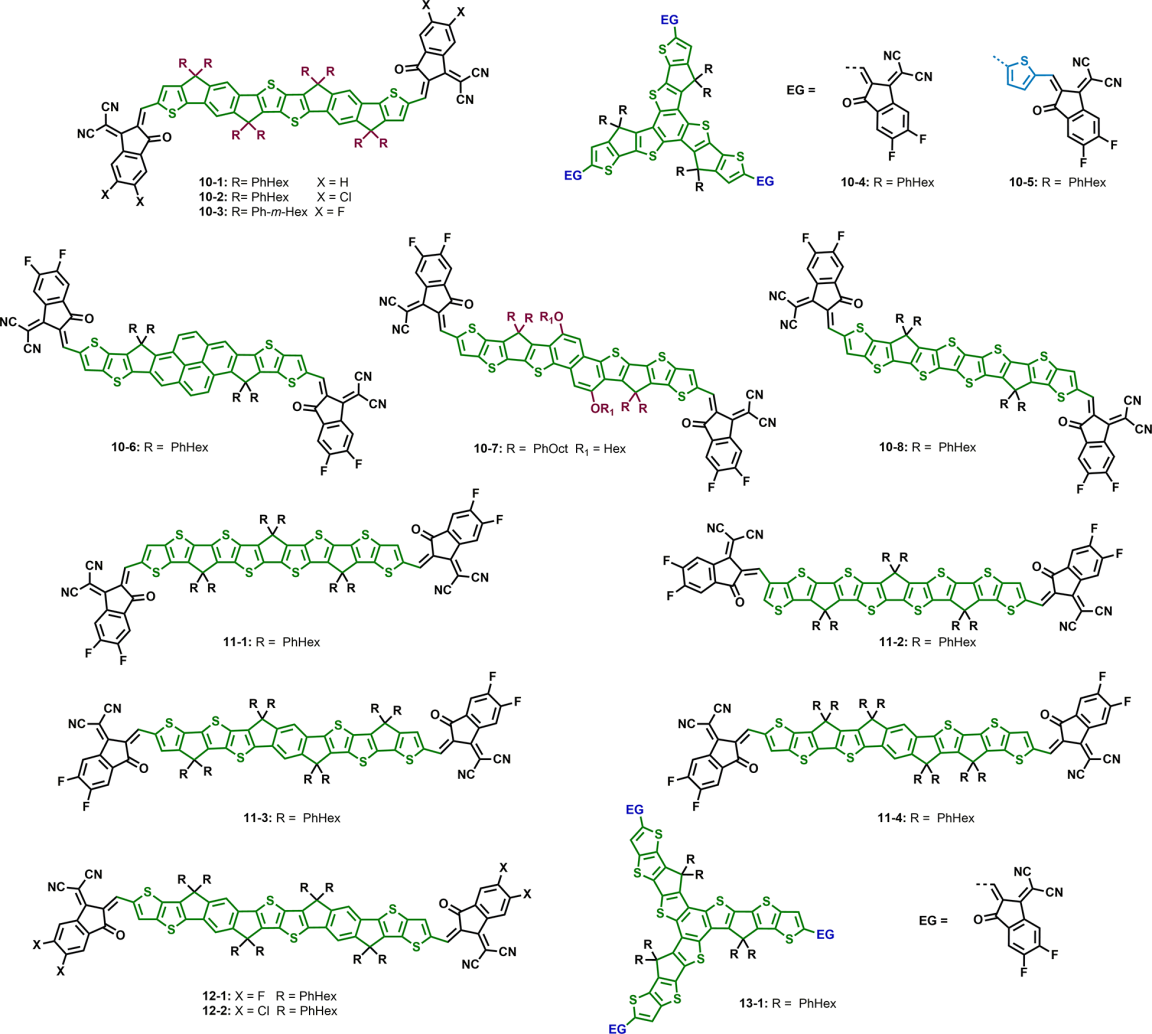
Structures of non-fullerene acceptors with 10–13 fused rings.

A majority of the reported NFAs containing fused decacyclic aromatic systems as a core unit are based on two fused IDT units (see Figure 21). In **10-1** (*p*-IDTIDT-IC) and **10-2** (R10-Cl), the double IDT unit contains *para*-hexylphenyl side chains and INCN (**10-1**) or INCN-2Cl end groups (**10-2**).^557,558^ The INCN-2Cl units lead to a reduced optical band gap (1.43 eV compared to 1.53 eV for **10-1**, see also Table 18). In combination with PTB7-Th, solar cells of **10-1** led to a PCE of 6.48%, while PBDB-T/**10-2**-based devices reached PCE values of 10.7%.^557,558^

**Table 18:** Optical, Electrical, and Photovoltaic Properties of the Non-Fullerene Acceptors Containing 10- and 12-Fused-Ring Structural Features

NFA	original name	HOMO^a^ (eV)	LUMO^a^ (eV)	*E*_g_^opt^ (eV)	donor	D:A ratio	*V*_OC_ (V)	*J*_SC_(mA cm^–2^)	FF (%)	PCE (%)	μ_e_^b^(cm^2^ V^–1^ s^–1^)	ref.
**10-1**	*p*-IDTIDT-IC	–5.42	–3.82	1.53	PTB7-Th	1:1.5	0.94	14.5	48	6.48	5.3 × 10^–6^/-	(557)
**10-2**	R10-Cl	–5.35	–3.91	1.43	PBDB-T		0.85	18.9	67	10.7	-/2.3 × 10^–5^	(558)
**10-3**	*m*-IDTIDT-FIC	–5.47	–3.94	1.51	J71	1:1.5	0.92	18.0	68	11.3	2.8 × 10^–5^/1.9 × 10^–4^	(557)
**10-4**	FBTIC	–5.70	–3.85	1.70	PM6	1:1	0.95	14.1	75	10.1	1.7 × 10^–3^/1.2 × 10^–3^	(559)
**10-5**	B3T-TT-6F	–5.42	–4.04	1.59	PBDB-T	1:1.5	0.82	18.3	66	9.94	-/1.1 × 10^–4^	(560)
**10-6**	FPIC	–5.75	–3.97	1.63	PTB7-Th	1:2	0.76	15.3	73	8.45	1.7 × 10^–3^/4.7 × 10^–4^	(561)
**10-7**	F10IC2	–5.53	–3.97	1.35	PTB7-Th	1:1	0.77	23.8	69	12.5	-/1.2 × 10^–4^	(562)
**10-8**	F10IC1	–5.35	–3.96	1.29	PTB7-Th	1:1.2	0.72	23.4	72	12.3	-/6.1 × 10^–4^	(486)
**11-1**	FUIC	–5.31	–4.06	1.22	PTB7-Th	1:1.5	0.69	22.9	71	11.2	6.8 × 10^–4^/3.0 × 10^–4^	(563)
**11-2**	*i*-FUIC	–5.31	–3.99	1.28	PTB7-Th	1:1.5	0.78	20.9	63	10.3	3.0 × 10^–4^/1.4 × 10^–4^	(563)
**11-3**	IUIC	–5.45	–3.87	1.41	PTB7-Th	1:1.5	0.80	21.7	65	11.2	1.1 × 10^–3^/5.5 × 10^–4^	(564)
**11-4**	IUIC2	–5.32	–3.86	1.25	PTB7-Th	1:1.5	0.76	11.0	53	4.48	3.9 × 10^–4^/1.4 × 10^–5^	(371)
**12-1**	LC81	–5.49	–3.97	1.45	PBT1-C	1:1.1	0.88	19.8	73	12.7	7.0 × 10^–4^/5.5 × 10^–4^	(565)
**12-2**	R12-4Cl	–5.26	–3.95	1.35	PBDB-T		0.75	18.5	67	9.3	-/2.1 × 10^–5^	(558)
**13-1**	BTTCTT-ICF	–5.64	–4.08	1.54	PM6	1:1.5	0.91	16.8	68	10.4	-/1.6 × 10^–5^	(566)
	B3T-BT-6F	–5.62	–4.03	1.69	PBDB-T	1:1.2	0.81	16.5	63	8.40	-/1.2 × 10^–4^	(560)

a Obtained from the oxidation/reduction potential of the CV measurement if not otherwise stated.

b Determined via the SCLC technique from the neat acceptor/donor:acceptor blend films if not otherwise stated.

Compared to that, **10-3** (*m*-IDTIDT-FIC) bears *meta*-hexylphenyl side chains and INCN-2F termini.^557^ The difference in the position of the alkylphenyl side chain has a significant effect on the molecular packing and thus on the electron mobility. The *meta*-alkylphenyl side chains in **10-3** lead to a smaller π–π stacking distance, an increased crystalline coherence length, and a higher mobility. This is also reflected in the PCE of the respective solar cells, which is increased from 6.48 to 8.27% due to a significantly enhanced FF when **10-3** is used instead of **10-1** in combination with PTB7-Th. By replacing PTB7-Th with J71, the PCE of solar cells using **10-3** could be further improved to 11.3%.^557^

The star shaped acceptors **10-4** (FBTIC) in solar cells with PM6 reach a good *V*_OC_ (0.95 V) and fill factor (75%). However, this blend has a narrow spectral response (<750 nm) which limits the *J*_SC_ value (14.1 mA cm^–2^). Nevertheless, a PCE of 10.1% is reached.^559^ The spectral response is improved if a thiophene linker is incorporated between the central core and the end groups as in acceptor **10-5** (B3T-TT-6F). However, devices of **10-5** with PBDB-T have a lower electron mobility and thus lower *J*_SC_ values (18.3 mA cm^–2^), resulting in lower PCEs (9.94%).^560^ A similar problem can be observed for the acceptor **10-6** (FPIC), which reaches 8.45% PCE when blended with PTB7-Th. The solar cells have reasonable *V*_OC_ and FF values (0.76 V, 73%), and thus, the PCE seems mainly limited by the lack of the blend’s absorption above 800 nm.^561^ Compound **10-7** (F10IC2) is structurally similar, but the central core is made electron rich by replacing two of the benzene rings with thiophene and adding alkyloxy substituents to the other two benzene rings. This lowers the HOMO energy, resulting in a narrower band gap (1.35 eV). Since the LUMO energies of both acceptors are the same, also blends with PTB7-Th have the same *V*_OC_. The slightly lower electron mobility and FF (69%) are compensated by the large *J*_SC_ (23.8 mA cm^–2^), yielding a good PCE of 12.5%.^562^

Acceptor **10-8** (F10IC1) combines a bis(thienothiophene) unit with two cyclopentathienothiophenes (T-T-Cp-T-T-T-T-Cp-T-T motif) and INCN-2F end groups and yields the smallest optical band gap for the fused 10-ring systems covered here. Solar cells with PTB7-Th reach good *J*_SC_ (23.4 mA cm^–2^) and PCE values (12.3%). Its analogues with eight-fused-ring (**8-12**) and six-fused-ring (**6-13**) central cores reach lower efficiencies (10.7 and 7.00%, respectively).^486^ Incorporation of one more cyclopentadiene moiety in the centers gives the 11-ring system **11-1** (FUIC).^563^ Despite the very low optical band gap (1.22 eV), devices with PTB7-Th do not reveal an improved *J*_SC_ (22.9 mA cm^–2^) and yield PCE values up to 11.2%. A reason for this could be the lower electron mobility. Linking one of the end groups to the β-carbon of the central core’s last thiophene gives the asymmetric **11-2** (*i*-FUIC). This acceptor has a higher LUMO level; thus, solar cells with PTB7-Th reach higher *V*_OC_ values than for **11-1** (0.78 V vs 0.69 V), but the PCEs were not improved (10.3%).^563^

**11-3** (IUIC) derives from **7-4** (ITIC-4F) and contains an extended conjugated core with the motif T-Cp-T-T-Cp-B-Cp-T-T-Cp-T. This leads to a smaller band gap, a higher charge carrier mobility, and an improved photovoltaic performance of 11.2% using PTB7-Th as a polymer.^564^ However, **11-4** (IUIC2), which is an extended version of **5-24** (IDIC-4F) with the structure T-T-Cp-T-Cp-B-Cp-T-Cp-T-T, led to neither increased charge carrier mobility nor improved PCEs, whereas the unsymmetrically extended compound **8-21** showed better mobility and a significantly higher PCE of 13.7%.^371^

The reported 12-fused-ring systems reveal a similarly replicated IDT structure as the aforementioned 10-fused-ring systems with additional peripheral thiophenes on both sides.^565^ Acceptor **12-1** (LC81) has INCN-2F end groups,^565^ whereas NFA **12-2** (R12-4Cl) comprises INCN-2Cl units.^558^ The chlorinated analogue **12-2** has a narrower optical band gap of 1.35 eV, while the LUMO energy levels of both acceptors are similar. Solar cells with **12-1** and PBT1-C reach efficiencies up to 12.7%, whereas those of **12-2** in combination with PBDB-T give 9.3%. The 10-fused-ring analogue **10-2** with the donor PBDB-T reached a PCE of 10.7%, serving as an example where an acceptor with a shorter conjugation length performs better than its higher analogue.^558^

The star shaped acceptor **13-1** (BTTCTT-ICF/B3T-BT-6F) is based on a truxene core and consists of 13 fused rings. The truxene core is not a strong electron donor; thus, despite the large π-system, this molecule has a rather high optical band gap. Blends with PBDB-T yield 8.40% PCE. This result is slightly outperformed by **10-5** (PCE 9.94%). Both molecules have the same number of conjugated rings, but the terminal thiophene unit is not fused to the central core, which is beneficial to the phase morphology.^560^ At the same time, blends of **13-1** with PM6 reach a higher PCE (10.4%) with the main improvement being found in the higher *V*_OC_ (0.91 V vs 0.81 V for the blend with PBDB-T).^566^

## 9 Conclusion and Outlook

Summarizing the large number of fused-ring molecules reported in the last three years enables the possibility of making more general conclusions about non-fullerene acceptor design. Some of the design strategies are universal to all of the acceptors discussed in this Review. For example, the length and sterical bulk of the side chains allows tuning of the material processability and blend morphology. Here, linear alkyl chains are to be favored for an increased crystallinity, while bulky (such as phenylhexyl) or branched units (ethylhexyl) are preferable for a decreased crystallinity. Longer alkyl chains often facilitate the processability. However, when choosing the side chains, also the properties of the desired donor material need to be taken into account, i.e., to have appropriate miscibility and phase separation of both materials in the absorber layer blend.

**Figure 22:**
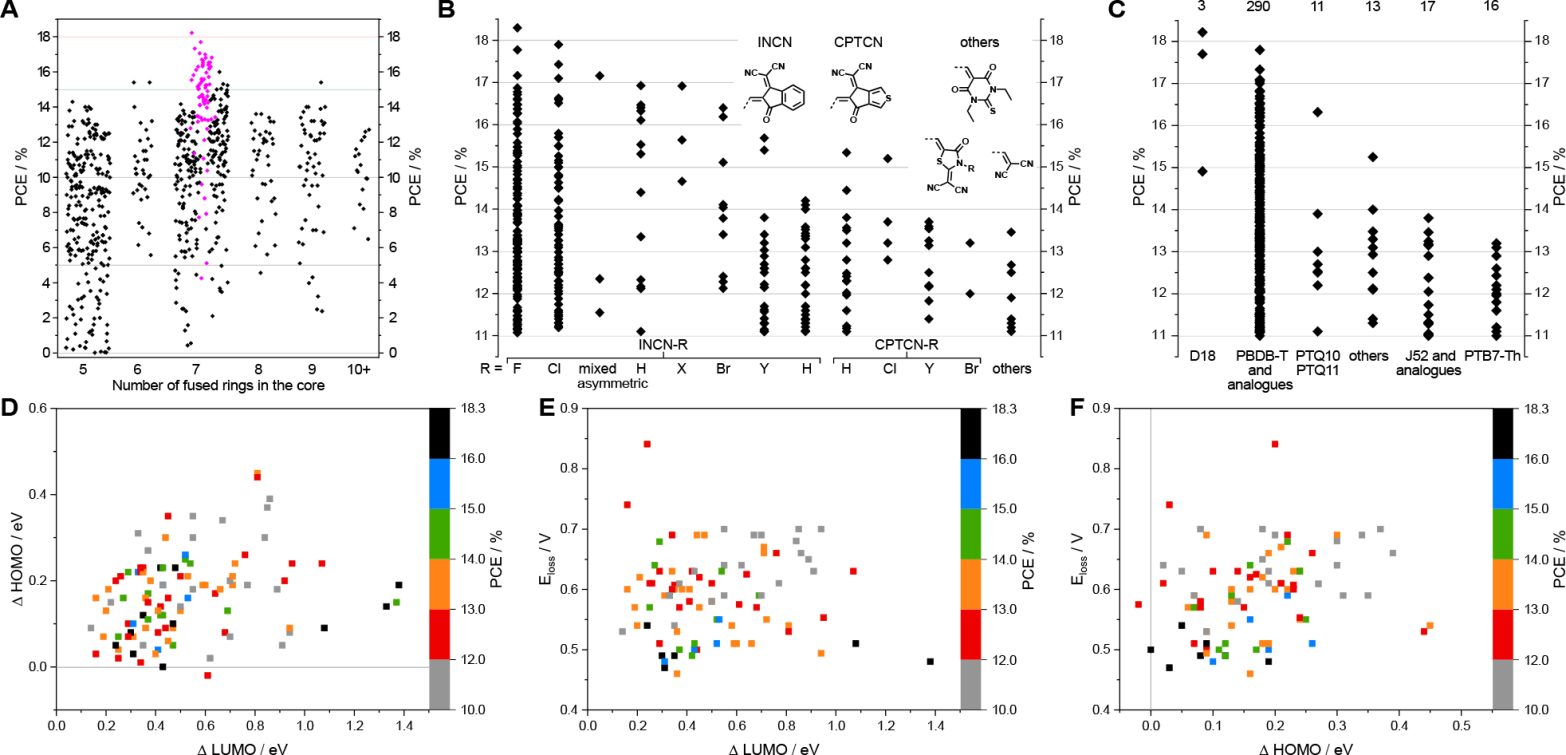
(A) PCEs obtained with the NFAs covered in this Review grouped by the number of fused rings in their donor core (entries of the Y-series are marked in magenta), (B) PCEs obtained with NFAs bearing different commonly used end groups, and (C) conjugated polymers applied in non-fullerene OSCs with PCEs exceeding 11%. (D) ΔHOMO versus ΔLUMO levels of donor and acceptor; the data of representative donor:acceptor combinations from each core size revealing PCEs above 11% are shown. (E) *E*_loss_ versus ΔLUMO levels and (F) *E*_loss_ versus ΔHOMO levels of donor and acceptor. The respective PCE ranges of the solar cells are indicated by the color scale.

The data covered in this Review reveal the trend that NFAs with larger fused-ring systems (≥6 rings), as can be seen in Figure 22A, show a higher fraction of PCE values above 10% compared to solar cells based on five-fused-ring NFAs, but they also reveal that PCEs above 15% are mainly reached with seven-fused-ring systems, in particular with NFAs related to the Y-series (highlighted in magenta), whereas only two compounds containing a six-fused-ring core and one with a nine-fused-ring core show PCEs above 15%. The photovoltaic performance of all NFAs belonging to the groups of 5-, 8-, and 10+-fused-ring systems stays below this threshold.

This is an interesting fact, as generally an increased conjugation length of the fused-ring core leads to narrower band gaps, upshifted HOMO and LUMO levels, as well as increased charge carrier mobility due to enhanced molecular packing, which is beneficial for the performance in solar cells. However, the length of the fused-ring core also significantly influences the solubility of the compounds and the phase morphology of the donor and the acceptor. Therefore, an extension of the conjugation length does not automatically lead to increased PCE values. In this regard, some studies reveal that it is more difficult to obtain sufficient solubility or ideal phase morphology with the NFAs containing larger conjugated fused-ring cores.^371,372^

Moreover, often the preparation of these larger conjugated fused-ring systems is synthetically more demanding compared to smaller ones and the synthesis routes of many five- and seven-fused-ring acceptors are well established, with many building blocks being commercially available. As, in addition, the majority of the reported seven-ring systems combined with a suitable donor polymer can reach PCEs above 10% (65% of the seven-ring compounds in this Review) and the current record efficiencies are obtained with NFAs of this core size, it comes as no surprise that in the last years seven-fused-ring systems have been studied and applied in solar cells very frequently.

The herein discussed acceptor systems usually comprise an A–D–A-type structure. By analyzing the solar cell data of devices with PCE values of more than 11%, there is a rather large variety of different electron-donating cores with the A–DA′D–A structure of the Y-series as the most promising motifs. However, also other seven-ring-based cores, in particular, asymmetric structures with the motif T-Cp-B-Cp-T-P-T, as well as lower- and higher-numbered fused-ring molecules gave attractive values, whereby IDT-based compounds with thiophene π-spacers, asymmetric derivatives of Y-series structures, or some carbon–oxygen-bridged NFAs can be highlighted in this context.

A further important aspect to note is that the high photovoltaic performance of certain NFAs often cannot only be ascribed to their well suited optical and electronic properties, but also photophysical and structural peculiarities play crucial roles. Regarding Y6, one of the currently most efficient NFAs, major reasons for the low energy losses in solar cells comprising the Y6 acceptor have been found to be the conformational rigidity and uniformity. The alkyl chains at the outer thiophene units hinder the rotation of the end groups, leading to lower charge carrier trapping into electronic intra-bandgap tail states.^567^ Moreover, exciton delocalization, charge transfer, and transport in Y6 are facilitated due to a particular crystal packing of the Y6 molecules.^568,569^ This example clearly shows the intricate correlations of device physics with the molecular design as well as the crystalline structures of the NFAs and the importance of understanding these correlations for the further development of this field.

Furthermore, a large amount of research in this field in the recent years has been devoted to modifications of the end groups and a huge variety of different structures have been reported. In almost all highly efficient solar cells, which reveal PCE values above 11% (see Figure 22B), the used acceptors comprise INCN-based accepting groups, especially halogenated INCN units. Alternatively, acceptors with the thiophene analogue, the CPTCN motif, are much less investigated but also give promising solar cell efficiencies up to 15%. There are only three other electron-accepting units (a simple malonitrile-based end group, as well as RCN and TBA derivatives, summarized as “others” in Figure 22B) in this category, which reached PCEs up to 13.5% in solar cells.

Although not being the focus of this Review, the donor materials, in most cases a conjugated polymer, are of the same importance and a large number of materials have been introduced during the last years. By analyzing the solar cell data discussed in this Review, it is obvious that most of the solar cell optimization was carried out using only a few polymers. Figure 22C shows all donor materials used in solar cells with PCEs >11%. Within this category, PBDB-T-type polymers (such as PBDB-T, PM6, and PM7) are the most often used donor materials. Out of about 350 reported non-fullerene acceptors leading to solar cell efficiencies above 11%, approximately 280 contained a PBDB-T-type polymer in the absorber layer. Moreover, several of these solar cells showed PCEs above 17%. A higher PCE (18.2%) could only be otained with the recently designed donor material D18. Alternatively, polymers of the J-series and PTB7-Th have been used reaching PCEs up to 14%. Furthermore, it should be mentioned that PTQ10 is a very promising donor, especially due to its simple structure (synthetic considerations) and solar cells have revealed already very good performance.

As discussed above, it is generally accepted that both the LUMO and HOMO energies of the acceptor should be bigger than those of the donor to facilitate exciton separation. Generally, a value of 0.3 eV was postulated and this empirical driving energy has been regularly questioned for non-fullerene solar cells.^570^ Thus, we have extracted the LUMO–LUMO and HOMO–HOMO offsets of various NFA-conjugated polymer combinations from each of the fused-ring core sizes discussed in this Review (PCE values above 10%, representative examples from each chapter, the HOMO and LUMO values of both the donor and the acceptor have been taken from the respective articles) and have compared these data in regard to the power conversion efficiency and the energy loss (band gap of the solar cell - *V*_OC_). Figure 22D correlates the donor/acceptor HOMO and LUMO offsets of these highly efficient solar cells. The entries are grouped and color-coded regarding their efficiency. In general, high energy level offsets lead to lower energy losses within the charge separation process; however, a high offset in the HOMO values deteriorates the maximum possible *V*_OC_. Thus, by looking at the data, the differences in the HOMO offsets are generally small and exhibit values below 0.25 eV for the most efficient solar cells (PCEs above 15%). There are even data showing a negligible offset; however, as there are certain uncertainties in the determination of the exact orbital energies, this has to still be verified by further investigations. In contrast, the LUMO offset values vary in a larger range from 0.15 up to 1.4 eV. This supports the picture that the HOMO offset values are more crucial to reach higher PCE values due to the correlation with the *V*_OC_. Parts E and F of Figure 22 contain the energy loss data (optical band gap minus *V*_OC_) plotted against HOMO and LUMO orbital offsets, respectively. The approach of using the optical band gap of the component in the absorber layer with the lowest band gap for the calculation was chosen as an approximation for using the inflection point of the onset of the EQE spectrum.^571^ As expected, the best solar cells have a low energy loss (<0.6 eV). The distribution of orbital offsets is much smaller for the HOMO, up to 0.5 eV, with the best solar cells having values below 0.3 eV. Regarding the LUMO, the orbital offset distribution is much broader (up to 1.5 eV) and highly efficient cells can be found over the entire range.

**Figure 23:**
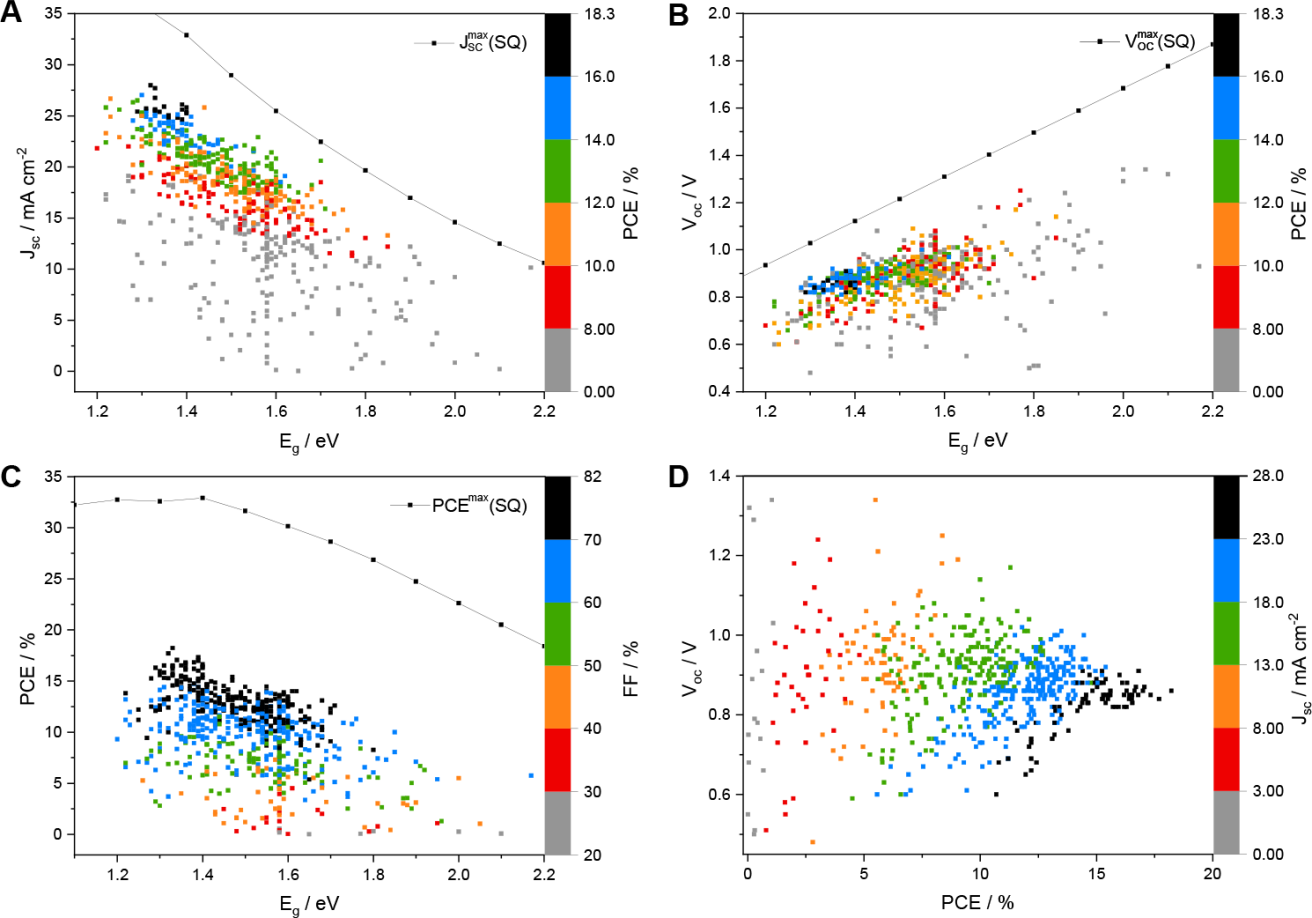
(A) *J*_SC_, (B) *V*_OC_, and (C) PCE of the solar cells covered in this Review plotted vs the optical band gap of the component with the lower band gap in the absorber layer. While in parts A and B the data points are color coded by the PCE of the solar cells, in part C, they are color coded according to the respective FF value. The line represents the theoretical Shockley–Queisser limit. (D) *V*_OC_ plotted against the PCE with the *J*_SC_ value in the color code information.

Finally, we analyzed the dependence of the characteristic solar cell parameters on the band gap of the absorber layer. Parts A–C of Figure 23 show the *J*_SC_, *V*_OC_, and PCE plotted against the optical band gap of the longest wavelength absorbing component of the devices. Moreover, the respective data points are color coded to additionally show the PCE (Figure 23A,B) and the FF (Figure 23C) of the respective solar cell. It can be seen from Figure 23A that only absorber layer blends with band gaps below 1.7 eV yield solar cells with efficiencies above 12% and particularly high *J*_SC_s (>25 mA cm^–2^) have been obtained for active layers made of acceptors with band gaps around 1.45 eV. The maximal open circuit voltage the solar cell can reach is reduced (Figure 23B) when lower band gap absorbers are used. Therefore, highly efficient solar cells with *V*_OC_s above 1 V are hardly obtained. As is shown in Figure 23D, all solar cells with PCEs above 15% exhibit very similar photovoltages ranging between 0.80 and 0.90 V.

In the graphs in Figure 23A and B, also the maximum theoretical *J*_SC_ and *V*_OC_ values according to the Shockley–Queisser (SQ) limit are included to indicate the distance of the already obtained values in organic solar cells compared to the maximum achievable ones.^3,572,573^ From both graphs, it becomes clear that the currently most efficient solar cells have band gaps between 1.30 and 1.40 eV. In both values, *V*_OC_ and *J*_SC_, OPV devices are reaching up to 80% of the theoretical limit. These differences to the SQ limits in *V*_OC_ and *J*_SC_ are currently larger than in other PV technologies such as GaAs and Si but also perovskite solar cells.^3^ This might be caused by the intrinsic donor–acceptor heterojunction of OPVs with expected higher *V*_OC_ and recombination losses compared to other technologies. The FF values of the best OPV devices are also reaching approx. 80% of the theoretical limit, and thus, overall, the best efficiencies of organic solar cells are currently slightly above 50% of the SQ limit (see Figure 23C).

Despite the significant progress in the last decade, organic photovoltaics have yet to reach and to exceed the milestone efficiency of 20%. An even more detailed understanding of the materials, processes, and mechanisms involved in non-fullerene organic solar cells will be very beneficial to guide material design toward this ambitious target. Besides, the power conversion efficiency, aspects such as device stability, and the scalability of materials synthesis and coating processes become increasingly important in view of the applicability of OPV and its commercialization. In particular, some issues have to be addressed in the acceptor molecule design and synthesis, such as the research toward simple, large scale, and high yield synthesis methods for the most efficient non-fullerene acceptor molecules. Regarding the stability of NFAs, the majority of compounds reported in this Review have a good thermal stability: however, this is not always associated with good (photo)chemical stability. Thus, improving the latter is another crucial hurdle, which has to be overcome in NFA design. Finally, a facile and reproducible processability and also the use of green solvents in the coating processes are crucial aspects to be taken into account in future research on non-fullerene acceptors.
